# Supplementary material for: The functioning of different beetle (Coleoptera) sampling methods across altitudinal gradients in Peninsular Malaysia
Source: PLoS One. 2022 Mar 31;17(3):e0266076. doi: 10.1371/journal.pone.0266076 (PMC8970512; doi:10.1371/journal.pone.0266076)
Supplement: S1 Table — (DOCX) [file pone.0266076.s001.docx]

**S1 Table. GLMM outputs for data collected using different sampling methods; number of species, rarefaction standardized species richness to 5, 10 and 20 individuals, and coverage-based asymptotic richness.** Statistics shown for random and fixed effects, followed by ANOVA table. Var = variance, SD = standard deviation, Estimate = estimated difference from baseline (Intercept; set as 500 m a.s.l.), SE = standard error for Estimate; df = degrees of freedom (estimates for GLMM), t = GLMM test statistic, p = probability for no difference, SS = sum-of-squares, MS = mean squares, F = ANOVA test statistic. Sample sizes were 15 for light, 14 for Malaise and 40 for pitfall trap data. For model fits, see S1 Fig.

| **LIGHT TRAPS** | |  |  |  |  |
| --- | --- | --- | --- | --- | --- |
| *Number of species* | |  |  |  |  |
| **Random** | **Var** | **SD** |  |  |  |
| Mountain | 4.67 | 2.16 |  |  |  |
| Residual | 95.74 | 9.79 |  |  |  |
| **Fixed** | **Estimate** | **SE** | **df** | **t** | **p** |
| (Intercept) | 27.07 | 5.87 | 7.85 | 4.61 | 0.0018 |
| 1,000 | -9.07 | 7.49 | 10.18 | -1.21 | 0.2529 |
| 1,500 | -10.07 | 7.49 | 10.18 | -1.35 | 0.2077 |
| 1,800 | -20.32 | 7.49 | 10.18 | -2.72 | 0.0214 |
| **AOV** | **df** | **SS** | **MS** | **F** | **p** |
| Altitude | 3 | 742.20 | 247.39 | 2.58 | 0.1120 |
| Mountain | 1 | 130.00 | 130.01 | 1.36 | 0.2710 |
| Residuals | 10 | 957.40 | 95.74 |  |  |
| *Rarefied richness to 5 individuals* | | | |  |  |
| **Random** | **Var** | **SD** |  |  |  |
| Mountain | 0.00 | 0.04 |  |  |  |
| Residual | 0.11 | 0.33 |  |  |  |
| **Fixed** | **Estimate** | **SE** | **df** | **t** | **p** |
| (Intercept) | 4.32 | 0.19 | 8.61 | 22.54 | 0.0000 |
| 1,000 | 0.00 | 0.25 | 10.22 | -0.01 | 0.9952 |
| 1,500 | -0.12 | 0.25 | 10.22 | -0.46 | 0.6539 |
| 1,800 | -0.48 | 0.25 | 10.22 | -1.93 | 0.0822 |
| **AOV** | **df** | **SS** | **MS** | **F** | **p** |
| Altitude | 3 | 0.60 | 0.20 | 1.86 | 0.2010 |
| Mountain | 1 | 0.12 | 0.12 | 1.12 | 0.3150 |
| Residuals | 10 | 1.07 | 0.11 |  |  |
| *Rarefied richness to 10 individuals* | | | |  |  |
| **Random** | **Var** | **SD** |  |  |  |
| Mountain | 0.27 | 0.52 |  |  |  |
| Residual | 0.72 | 0.85 |  |  |  |
| **Fixed** | **Estimate** | **SE** | **df** | **t** | **p** |
| (Intercept) | 8.23 | 0.74 | 4.62 | 11.15 | 0.0002 |
| 1,000 | -0.83 | 0.77 | 8.54 | -1.08 | 0.3092 |
| 1,500 | -1.04 | 0.77 | 8.54 | -1.35 | 0.2117 |
| 1,800 | -2.29 | 0.79 | 8.25 | -2.89 | 0.0194 |
| **AOV** | **df** | **SS** | **MS** | **F** | **p** |
| Altitude | 3 | 6.49 | 2.16 | 2.98 | 0.0962 |
| Mountain | 1 | 2.18 | 2.18 | 3.01 | 0.1209 |
| Residuals | 8 | 5.80 | 0.72 |  |  |
| *Rarefied richness to 20 individuals* | | | |  |  |
| **Random** | **Var** | **SD** |  |  |  |
| Mountain | 0.00 | 0.00 |  |  |  |
| Residual | 3.49 | 1.87 |  |  |  |
| **Fixed** | **Estimate** | **SE** | **df** | **t** | **p** |
| (Intercept) | 13.07 | 1.32 | 8.00 | 9.90 | 0.0000 |
| 1,000 | -1.49 | 1.62 | 8.00 | -0.92 | 0.3840 |
| 1,500 | -1.50 | 1.62 | 8.00 | -0.93 | 0.3802 |
| 1,800 | -6.07 | 1.87 | 8.00 | -3.25 | 0.0117 |
| **AOV** | **df** | **SS** | **MS** | **F** | **p** |
| Altitude | 3 | 43.10 | 14.37 | 4.03 | 0.0586 |
| Mountain | 1 | 2.94 | 2.94 | 0.83 | 0.3935 |
| Residuals | 7 | 24.94 | 3.56 |  |  |
| *Coverage-based asymptotic richness* | | | |  |  |
| **Random** | **Var** | **SD** |  |  |  |
| Mountain | 308.2 | 17.56 |  |  |  |
| Residual | 773.10 | 27.81 |  |  |  |
| **Fixed** | **Estimate** | **SE** | **df** | **t** | **p** |
| (Intercept) | 61.67 | 20.40 | 3.63 | 3.02 | 0.0443 |
| 1,000 | -23.25 | 21.34 | 10.06 | -1.09 | 0.3013 |
| 1,500 | -33.33 | 21.34 | 10.06 | -1.56 | 0.1492 |
| 1,800 | -60.93 | 21.34 | 10.06 | -2.86 | 0.0170 |
| **AOV** | **df** | **SS** | **MS** | **F** | **p** |
| Altitude | 3 | 7428.00 | 2476.00 | 3.20 | 0.0707 |
| Mountain | 1 | 3034.00 | 3033.50 | 3.92 | 0.0758 |
| Residuals | 10 | 7731.00 | 773.10 |  |  |
| **MALAISE TRAPS** | |  |  |  |  |
| *Number of species* | |  |  |  |  |
| **Random** | **Var** | **SD** |  |  |  |
| Mountain | 2.83 | 1.68 |  |  |  |
| Residual | 19.78 | 4.45 |  |  |  |
| **Fixed** | **Estimate** | **SE** | **df** | **t** | **p** |
| (Intercept) | 36.69 | 3.47 | 6.35 | 10.56 | 0.0000 |
| 1,000 | -15.94 | 3.95 | 9.80 | -4.04 | 0.0025 |
| 1,500 | -19.94 | 3.95 | 9.80 | -5.05 | 0.0005 |
| 1,800 | -21.44 | 3.95 | 9.80 | -5.43 | 0.0003 |
| **AOV** | **df** | **SS** | **MS** | **F** | **p** |
| Altitude | 3 | 744.70 | 248.23 | 12.55 | 0.0014 |
| Mountain | 1 | 36.70 | 36.75 | 1.86 | 0.2060 |
| Residuals | 9 | 178.00 | 19.78 |  |  |
| *Rarefied richness to 5 individuals* | | | |  |  |
| **Random** | **Var** | **SD** |  |  |  |
| Mountain | 0.01 | 0.10 |  |  |  |
| Residual | 0.04 | 0.20 |  |  |  |
| **Fixed** | **Estimate** | **SE** | **df** | **t** | **p** |
| (Intercept) | 4.57 | 0.16 | 5.90 | 27.94 | 0.0000 |
| 1,000 | -0.20 | 0.18 | 9.67 | -1.12 | 0.2893 |
| 1,500 | -0.24 | 0.18 | 9.67 | -1.36 | 0.2060 |
| 1,800 | -0.79 | 0.18 | 9.67 | -4.40 | 0.0014 |
| **AOV** | **df** | **SS** | **MS** | **F** | **p** |
| Altitude | 3 | 1.09 | 0.36 | 8.96 | 0.0046 |
| Mountain | 1 | 0.10 | 0.10 | 2.35 | 0.1601 |
| Residuals | 9 | 0.37 | 0.04 |  |  |
| *Rarefied richness to 10 individuals* | | | |  |  |
| **Random** | **Var** | **SD** |  |  |  |
| Mountain | 0.08 | 0.28 |  |  |  |
| Residual | 0.26 | 0.51 |  |  |  |
| **Fixed** | **Estimate** | **SE** | **df** | **t** | **p** |
| (Intercept) | 8.24 | 0.43 | 5.43 | 19.31 | 0.0000 |
| 1,000 | -0.70 | 0.46 | 9.57 | -1.53 | 0.1573 |
| 1,500 | -0.82 | 0.46 | 9.57 | -1.79 | 0.1044 |
| 1,800 | -2.24 | 0.46 | 9.57 | -4.92 | 0.0007 |
| **AOV** | **df** | **SS** | **MS** | **F** | **p** |
| Altitude | 3 | 7.93 | 2.64 | 10.24 | 0.0029 |
| Mountain | 1 | 0.73 | 0.73 | 2.83 | 0.1271 |
| Residuals | 9 | 2.32 | 0.26 |  |  |
| *Rarefied richness to 20 individuals* | | | |  |  |
| **Random** | **Var** | **SD** |  |  |  |
| Mountain | 0.34 | 0.59 |  |  |  |
| Residual | 1.00 | 1.00 |  |  |  |
| **Fixed** | **Estimate** | **SE** | **df** | **t** | **p** |
| (Intercept) | 13.80 | 0.85 | 5.21 | 16.17 | 0.0000 |
| 1,000 | -1.91 | 0.90 | 9.54 | -2.13 | 0.0604 |
| 1,500 | -2.23 | 0.90 | 9.54 | -2.49 | 0.0333 |
| 1,800 | -4.73 | 0.90 | 9.54 | -5.27 | 0.0004 |
| **AOV** | **df** | **SS** | **MS** | **F** | **p** |
| Altitude | 3 | 30.82 | 10.27 | 10.26 | 0.0029 |
| Mountain | 1 | 3.06 | 3.06 | 3.06 | 0.1142 |
| Residuals | 9 | 9.01 | 1.00 |  |  |
| *Coverage-based asymptotic richness* | | | |  |  |
| **Random** | **Var** | **SD** |  |  |  |
| Mountain | 361.10 | 19.00 |  |  |  |
| Residual | 195.20 | 13.97 |  |  |  |
| **Fixed** | **Estimate** | **SE** | **df** | **t** | **p** |
| (Intercept) | 69.68 | 17.12 | 2.13 | 4.07 | 0.0496 |
| 1,000 | -38.88 | 12.70 | 9.15 | -3.06 | 0.0133 |
| 1,500 | -48.97 | 12.70 | 9.15 | -3.86 | 0.0038 |
| 1,800 | -26.51 | 12.70 | 9.15 | -2.09 | 0.0659 |
| **AOV** | **df** | **SS** | **MS** | **F** | **p** |
| Altitude | 3 | 5469.00 | 1823.1 | 9.342 | 0.00398 |
| Mountain | 1 | 2362.00 | 2361.50 | 12.10 | 0.00695 |
| Residuals | 9 | 1756.00 | 195.20 |  |  |
| **PITFALL TRAPS** | |  |  |  |  |
| *Number of species* | |  |  |  |  |
| **Random** | **Var** | **SD** |  |  |  |
| Mountain | 26.50 | 5.15 |  |  |  |
| Residual | 10.17 | 3.19 |  |  |  |
| **Fixed** | **Estimate** | **SE** | **df** | **t** | **p** |
| (Intercept) | 11.40 | 3.78 | 1.12 | 3.02 | 0.1824 |
| 1,000 | 2.70 | 1.43 | 35.00 | 1.89 | 0.0666 |
| 1,500 | -1.00 | 1.43 | 35.00 | -0.70 | 0.4878 |
| 1,800 | -1.80 | 1.43 | 35.00 | -1.26 | 0.2152 |
| **AOV** | **df** | **SS** | **MS** | **F** | **p** |
| Altitude | 3 | 115.30 | 38.40 | 3.78 | 0.0189 |
| Mountain | 1 | 540.20 | 540.20 | 53.13 | 0.0000 |
| Residuals | 35 | 355.90 | 10.20 |  |  |
| *Rarefied richness to 5 individuals* | | | |  |  |
| **Random** | **Var** | **SD** |  |  |  |
| Mountain | 0.06 | 0.25 |  |  |  |
| Residual | 0.16 | 0.40 |  |  |  |
| **Fixed** | **Estimate** | **SE** | **df** | **t** | **p** |
| (Intercept) | 3.72 | 0.22 | 1.78 | 17.14 | 0.0055 |
| 1,000 | 0.37 | 0.18 | 35.00 | 2.07 | 0.0459 |
| 1,500 | -0.11 | 0.18 | 35.00 | -0.59 | 0.5562 |
| 1,800 | 0.17 | 0.18 | 35.00 | 0.94 | 0.3524 |
| **AOV** | **df** | **SS** | **MS** | **F** | **p** |
| Altitude | 3 | 1.28 | 0.43 | 2.71 | 0.0600 |
| Mountain | 1 | 1.41 | 1.41 | 8.92 | 0.0051 |
| Residuals | 35 | 5.53 | 0.16 |  |  |
| *Rarefied richness to 10 individuals* | | | |  |  |
| **Random** | **Var** | **SD** |  |  |  |
| Mountain | 0.45 | 0.67 |  |  |  |
| Residual | 0.66 | 0.81 |  |  |  |
| **Fixed** | **Estimate** | **SE** | **df** | **t** | **p** |
| (Intercept) | 5.88 | 0.54 | 1.44 | 10.92 | 0.0235 |
| 1,000 | 0.79 | 0.36 | 32.00 | 2.16 | 0.0387 |
| 1,500 | 0.02 | 0.39 | 32.04 | 0.06 | 0.9525 |
| 1,800 | 0.08 | 0.37 | 32.01 | 0.21 | 0.8326 |
| **AOV** | **df** | **SS** | **MS** | **F** | **p** |
| Altitude | 3 | 3.70 | 1.23 | 1.86 | 0.1562 |
| Mountain | 1 | 8.79 | 8.79 | 13.25 | 0.0010 |
| Residuals | 32 | 21.23 | 0.66 |  |  |
| *Rarefied richness to 20 individuals* | | | |  |  |
| **Random** | **Var** | **SD** |  |  |  |
| Mountain | 2.00 | 1.41 |  |  |  |
| Residual | 2.22 | 1.49 |  |  |  |
| **Fixed** | **Estimate** | **SE** | **df** | **t** | **p** |
| (Intercept) | 8.34 | 1.12 | 1.32 | 7.47 | 0.0489 |
| 1,000 | 1.46 | 0.69 | 26.01 | 2.12 | 0.0434 |
| 1,500 | 0.38 | 0.82 | 26.30 | 0.47 | 0.6410 |
| 1,800 | 0.24 | 0.82 | 26.30 | 0.29 | 0.7749 |
| **AOV** | **df** | **SS** | **MS** | **F** | **p** |
| Altitude | 3 | 12.02 | 4.01 | 1.81 | 0.1709 |
| Mountain | 1 | 27.78 | 27.78 | 12.52 | 0.0015 |
| Residuals | 26 | 57.69 | 2.22 |  |  |
| *Coverage-based asymptotic richness* | | | |  |  |
| **Random** | **Var** | **SD** |  |  |  |
| Mountain | 339.40 | 18.42 |  |  |  |
| Residual | 361.10 | 19.00 |  |  |  |
| **Fixed** | **Estimate** | **SE** | **df** | **t** | **p** |
| (Intercept) | 20.84 | 14.35 | 1.33 | 1.45 | 0.3390 |
| 1,000 | -4.24 | 8.50 | 35.00 | -0.50 | 0.6210 |
| 1,500 | -4.57 | 8.50 | 35.00 | -0.54 | 0.5940 |
| 1,800 | -11.68 | 8.50 | 35.00 | -1.38 | 0.1780 |
| **AOV** | **df** | **SS** | **MS** | **F** | **p** |
| Altitude | 3 | 703.00 | 234.00 | 0.65 | 0.5890 |
| Mountain | 1 | 7150.00 | 7150.00 | 19.80 | 0.0001 |
| Residuals | 35 | 12637.00 | 361.00 |  |  |
