# Supplementary material for: The functioning of different beetle (Coleoptera) sampling methods across altitudinal gradients in Peninsular Malaysia
Source: PLoS One. 2022 Mar 31;17(3):e0266076. doi: 10.1371/journal.pone.0266076 (PMC8970512; doi:10.1371/journal.pone.0266076)
Supplement: S2 Table — (DOCX) [file pone.0266076.s002.docx]

**S2 Table. Wilk-Shapiro test for GLMM residuals plotted in S1 Fig.**

| **Data subset** | **Light traps** | **Malaise traps** | **Pitfall traps** |
| --- | --- | --- | --- |
| No. species | W = 0.9168, p = 0.1722 | W = 0.9620, p = 0.7565 | W = 0.9730, p = 0.4465 |
| Rarefied to 5 ind. | W = 0.9101, p = 0.1358 | W = 0.9396, p = 0.4132 | W = 0.8520, p = 0.0001 |
| Rarefied to 10 ind. | W = 0.8756, p = 0.0621 | W = 0.9684, p = 0.8552 | W = 0.9334, p = 0.0285 |
| Rarefied to 20 ind. | W = 0.9423, p = 0.5283 | W = 0.9756, p = 0.9411 | W = 0.9713, p = 0.5556 |
| CB asymptotic richness | W = 0.9262, p = 0.2390 | W = 0.8730, p = 0.0462 | W = 0.8416, p = 0.0001 |
