# Supplementary material for: The functioning of different beetle (Coleoptera) sampling methods across altitudinal gradients in Peninsular Malaysia
Source: PLoS One. 2022 Mar 31;17(3):e0266076. doi: 10.1371/journal.pone.0266076 (PMC8970512; doi:10.1371/journal.pone.0266076)
Supplement: S3 Table — (DOCX) [file pone.0266076.s003.docx]

**S3 Table. Wilk-Shapiro (W) and Breusch-Pagan (BP; df = 4) tests for ANOVA residuals; model Mountain + Altitude.**

| **Variable** | **Normality** | **Heteroskedasticity** |
| --- | --- | --- |
| **Light traps** |  |  |
| No. species | W = 0.9325, p = 0.2968 | BP = 9.5530, p = 0.0487 |
| Rarefied to 5 ind. | W = 0.8490, p = 0.01683 | BP = 0.7878, p = 0.9401 |
| Rarefied to 10 ind. | W = 0.9194, p = 0.2459 | BP = 3.2842, p = 0.5114 |
| Rarefied to 20 ind. | W = 0.8991, p = 0.1544 | BP = 2.2721, p = 0.6858 |
| CB asymptotic richness | W = 0.9001, p = 0.0954 | BP = 4.0895, p = 0.3940 |
| **Malaise traps** |  |  |
| No. species | W = 0.9762, p = 0.9463 | BP = 10.1420, p = 0.0381 |
| Rarefied to 5 ind. | W = 0.9585, p = 0.6988 | BP = 5.9081, p = 0.2061 |
| Rarefied to 10 ind. | W = 0.9425, p = 0.4517 | BP = 3.1458, p = 0.5337 |
| Rarefied to 20 ind. | W = 0.9573, p = 0.6790 | BP = 4.0056, p = 0.4052 |
| CB asymptotic richness | W = 0.9012, p = 0.1175 | BP = 5.7338, p = 0.2199 |
| **Pitfall traps** |  |  |
| No. species | W = 0.9717, p = 0.4061 | BP = 5.8278, p = 0.2124 |
| Rarefied to 5 ind. | W = 0.8613, p = 0.0001 | BP = 2.0305, p = 0.7301 |
| Rarefied to 10 ind. | W = 0.9356, p = 0.0334 | BP = 2.7825, p = 0.5949 |
| Rarefied to 20 ind. | W = 0.9688, p = 0.4870 | BP = 2.2930, p = 0.6820 |
| CB asymptotic richness | W = 0.8480, p = 0.0001 | BP = 9.2110, p = 0.0560 |
