# Supplementary material for: The functioning of different beetle (Coleoptera) sampling methods across altitudinal gradients in Peninsular Malaysia
Source: PLoS One. 2022 Mar 31;17(3):e0266076. doi: 10.1371/journal.pone.0266076 (PMC8970512; doi:10.1371/journal.pone.0266076)
Supplement: S4 Table — (DOCX) [file pone.0266076.s004.docx]

**S4 Table. Robust LMM output for the number of species, rarefied richness standardized to 5, 10 or 20 individuals and coverage-based asymptotic richness for three trapping methods.** The column ESTINIT shows model estimates of the initial GLMM (Table 2) for quick comparisons.

| **Light traps** |  |  |  |  |
| --- | --- | --- | --- | --- |
| *Number of species* |  |  |  |  |
| Scaled residuals: |  |  |  |  |
| Min | 1Q | Median | 3Q | Max |
| -4.32 | -0.64 | 0.04 | 0.67 | 1.14 |
| Random effects: | Variance | SD |  |  |
| Mountain (intercept) | 0.00 | 0.00 |  |  |
| Residuals | 49.08 | 7.01 |  |  |
| Fixed effects: | Estimate | SE | t | **ESTINIT** |
| (Intercept) | 34.29 | 4.15 | 8.27 | **27.07** |
| Altitude 1000 | -16.29 | 5.49 | -2.97 | **-9.07** |
| Altitude 1500 | -17.29 | 5.49 | -3.15 | **-10.07** |
| Altitude 1800 | -27.54 | 5.49 | -5.02 | **-20.32** |
| *Rarefied richness to 5 individuals* | | | | |
| Scaled residuals: |  |  |  |  |
| Min | 1Q | Median | 3Q | Max |
| -1.39 | -0.78 | 0.20 | 0.49 | 1.05 |
| Random effects: | Variance | SD |  |  |
| Mountain (intercept) | 0.00 | 0.00 |  |  |
| Residuals | 0.15 | 0.38 |  |  |
| Fixed effects: | Estimate | SE | t | **ESTINIT** |
| (Intercept) | 4.30 | 0.23 | 18.99 | **4.32** |
| Altitude 1000 | 0.03 | 0.30 | 0.08 | **0.00** |
| Altitude 1500 | -0.10 | 0.30 | -0.33 | **-0.12** |
| Altitude 1800 | -0.47 | 0.30 | -1.56 | **-0.48** |
| *Rarefied richness to 10 individuals* | | | | |
| Scaled residuals: |  |  |  |  |
| Min | 1Q | Median | 3Q | Max |
| -1.19 | -0.21 | 0.20 | 0.62 | 0.79 |
| Random effects: | Variance | SD |  |  |
| Mountain (intercept) | 0.34 | 0.58 |  |  |
| Residuals | 0.99 | 1.00 |  |  |
| Fixed effects: | Estimate | SE | t | **ESTINIT** |
| (Intercept) | 8.19 | 0.87 | 9.38 | **8.23** |
| Altitude 1000 | -0.79 | 0.92 | -0.86 | **-0.83** |
| Altitude 1500 | -0.99 | 0.92 | -1.07 | **-1.04** |
| Altitude 1800 | -2.26 | 0.95 | -2.38 | **-2.29** |
| *Rarefied richness to 20 individuals* | | | | |
| Scaled residuals: |  |  |  |  |
| Min | 1Q | Median | 3Q | Max |
| -2.19 | -0.44 | 0.21 | 0.58 | 0.99 |
| Random effects: | Variance | SD |  |  |
| Mountain (intercept) | 0.00 | 0.00 |  |  |
| Residuals | 3.22 | 1.79 |  |  |
| Fixed effects: | Estimate | SE | t | **ESTINIT** |
| (Intercept) | 13.10 | 1.30 | 10.07 | **13.07** |
| Altitude 1000 | -1.55 | 1.59 | -0.97 | **-1.49** |
| Altitude 1500 | -1.17 | 1.59 | -0.74 | **-1.50** |
| Altitude 1800 | -6.10 | 1.84 | -3.32 | **-6.07** |
| *Coverage-based asymptotic richness* | | | | |
| Scaled residuals: |  |  |  |  |
| Min | 1Q | Median | 3Q | Max |
| -2.36 | -0.38 | 0.37 | 0.45 | 1.22 |
| Random effects: | Variance | SD |  |  |
| Mountain (intercept) | 319.80 | 17.88 |  |  |
| Residuals | 654.10 | 25.57 |  |  |
| Fixed effects: | Estimate | SE | t | **ESTINIT** |
| (Intercept) | 70.68 | 20.04 | 3.527 | **61.67** |
| Altitude 1000 | -32.26 | 20.14 | -1.602 | **-23.25** |
| Altitude 1500 | -41.26 | 20.14 | -2.049 | **-33.33** |
| Altitude 1800 | -69.94 | 20.14 | -3.473 | **-60.93** |
|  |  |  |  |  |
| **Malaise traps** |  |  |  |  |
| *Number of species* | | | | |
| Scaled residuals: |  |  |  |  |
| Min | 1Q | Median | 3Q | Max |
| -1.80 | -0.32 | -0.06 | 0.64 | 1.61 |
| Random effects: | Variance | SD |  |  |
| Mountain (intercept) | 4.01 | 2.00 |  |  |
| Residuals | 16.21 | 4.03 |  |  |
| Fixed effects: | Estimate | SE | t | **ESTINIT** |
| (Intercept) | 36.55 | 3.39 | 10.78 | **36.69** |
| Altitude 1000 | -15.33 | 3.69 | -4.15 | **-15.94** |
| Altitude 1500 | -19.80 | 3.69 | -5.36 | **-19.94** |
| Altitude 1800 | -21.30 | 3.69 | -5.77 | **-21.44** |
| *Rarefied richness to 5 individuals* | | | | |
| Scaled residuals: |  |  |  |  |
| Min | 1Q | Median | 3Q | Max |
| -1.53 | -0.42 | 0.02 | 0.52 | 1.00 |
| Random effects: | Variance | SD |  |  |
| Mountain (intercept) | 0.01 | 0.10 |  |  |
| Residuals | 0.05 | 0.22 |  |  |
| Fixed effects: | Estimate | SE | t | **ESTINIT** |
| (Intercept) | 4.57 | 0.19 | 24.64 | **4.57** |
| Altitude 1000 | -0.20 | 0.20 | -0.97 | **-0.20** |
| Altitude 1500 | -0.23 | 0.20 | -1.12 | **-0.24** |
| Altitude 1800 | -0.79 | 0.20 | -3.86 | **-0.79** |
| *Rarefied richness to 10 individuals* | | | | |
| Scaled residuals: |  |  |  |  |
| Min | 1Q | Median | 3Q | Max |
| -2.03 | -0.45 | 0.00 | 0.59 | 1.26 |
| Random effects: | Variance | SD |  |  |
| Mountain (intercept) | 0.08 | 0.29 |  |  |
| Residuals | 0.26 | 0.51 |  |  |
| Fixed effects: | Estimate | SE | t | **ESTINIT** |
| (Intercept) | 8.23 | 0.44 | 18.69 | **8.24** |
| Altitude 1000 | -0.68 | 0.47 | -1.46 | **-0.70** |
| Altitude 1500 | -0.71 | 0.47 | -1.53 | **-0.82** |
| Altitude 1800 | -2.22 | 0.47 | -4.76 | **-2.24** |
| *Rarefied richness to 20 individuals* | | | | |
| Scaled residuals: |  |  |  |  |
| Min | 1Q | Median | 3Q | Max |
| -1.79 | -0.50 | 0.11 | 0.53 | 1.78 |
| Random effects: | Variance | SD |  |  |
| Mountain (intercept) | 0.35 | 0.59 |  |  |
| Residuals | 0.93 | 0.96 |  |  |
| Fixed effects: | Estimate | SE | t | **ESTINIT** |
| (Intercept) | 13.76 | 0.85 | 16.13 | **13.80** |
| Altitude 1000 | -1.87 | 0.89 | -2.11 | **-1.91** |
| Altitude 1500 | -2.19 | 0.89 | -2.47 | **-2.23** |
| Altitude 1800 | -4.69 | 0.89 | -5.29 | **-4.73** |
| *Coverage-based asymptotic richness* | | | | |
| Scaled residuals: |  |  |  |  |
| Min | 1Q | Median | 3Q | Max |
| -1.07 | -0.46 | -0.21 | 0.47 | 1.60 |
| Random effects: | Variance | SD |  |  |
| Mountain (intercept) | 457.20 | 21.38 |  |  |
| Residuals | 205.70 | 14.34 |  |  |
| Fixed effects: | Estimate | SE | t | **ESTINIT** |
| (Intercept) | 70.02 | 19.12 | 3.66 | **69.68** |
| Altitude 1000 | -39.22 | 13.38 | -2.93 | **-38.88** |
| Altitude 1500 | -50.05 | 13.38 | -3.74 | **-48.97** |
| Altitude 1800 | -27.78 | 13.38 | -2.08 | **-26.51** |
|  |  |  |  |  |
| **Pitfall traps** |  |  |  |  |
| *Number of species* |  |  |  |  |
| Scaled residuals: |  |  |  |  |
| Min | 1Q | Median | 3Q | Max |
| -2.48 | -0.34 | 0.01 | 0.61 | 1.95 |
| Random effects: | Variance | SD |  |  |
| Mountain (intercept) | 40.08 | 6.33 |  |  |
| Residuals | 9.35 | 3.06 |  |  |
| Fixed effects: | Estimate | SE | t | **ESTINIT** |
| (Intercept) | 11.75 | 4.70 | 2.50 | **11.40** |
| Altitude 1000 | 2.37 | 1.40 | 1.69 | **2.70** |
| Altitude 1500 | -1.53 | 1.40 | -1.09 | **-1.00** |
| Altitude 1800 | -1.89 | 1.40 | -1.35 | **-1.80** |
| *Rarefied richness to 5 individuals* | |  |  |  |
| Scaled residuals: |  |  |  |  |
| Min | 1Q | Median | 3Q | Max |
| -3.57 | -0.38 | 0.18 | 0.58 | 1.34 |
| Random effects: | Variance | SD |  |  |
| Mountain (intercept) | 0.06 | 0.25 |  |  |
| Residuals | 0.09 | 0.30 |  |  |
| Fixed effects: | Estimate | SE | t | **ESTINIT** |
| (Intercept) | 3.82 | 0.20 | 18.71 | **3.72** |
| Altitude 1000 | 0.27 | 0.14 | 1.94 | **0.37** |
| Altitude 1500 | -0.11 | 0.14 | -0.76 | **-0.11** |
| Altitude 1800 | 0.14 | 0.14 | 0.98 | **0.17** |
| *Rarefied richness to 10 individuals* | | | | |
| Scaled residuals: |  |  |  |  |
| Min | 1Q | Median | 3Q | Max |
| -2.99 | -0.59 | 0.06 | 0.58 | 1.64 |
| Random effects: | Variance | SD |  |  |
| Mountain (intercept) | 0.59 | 0.77 |  |  |
| Residuals | 0.53 | 0.73 |  |  |
| Fixed effects: | Estimate | SE | t | **ESTINIT** |
| (Intercept) | 6.06 | 0.60 | 10.02 | **5.88** |
| Altitude 1000 | 0.59 | 0.33 | 1.76 | **0.79** |
| Altitude 1500 | -0.16 | 0.35 | -0.45 | **0.02** |
| Altitude 1800 | 0.03 | 0.34 | 0.09 | **0.08** |
| *Rarefied richness to 20 individuals* | | | | |
| Scaled residuals: |  |  |  |  |
| Min | 1Q | Median | 3Q | Max |
| -2.36 | -0.51 | -0.01 | 0.62 | 1.52 |
| Random effects: | Variance | SD |  |  |
| Mountain (intercept) | 2.52 | 1.59 |  |  |
| Residuals | 2.25 | 1.50 |  |  |
| Fixed effects: | Estimate | SE | t | **ESTINIT** |
| (Intercept) | 8.48 | 1.26 | 6.72 | **8.34** |
| Altitude 1000 | 1.30 | 0.71 | 1.83 | **1.46** |
| Altitude 1500 | 0.27 | 0.84 | 0.32 | **0.38** |
| Altitude 1800 | 0.27 | 0.84 | 0.32 | **0.24** |
| *Coverage-based asymptotic richness* | | | | |
| Scaled residuals: |  |  |  |  |
| Min | 1Q | Median | 3Q | Max |
| -1.39 | -0.56 | 0.16 | 0.39 | 6.48 |
| Random effects: | Variance | SD |  |  |
| Mountain (intercept) | 300.20 | 17.33 |  |  |
| Residuals | 170.90 | 13.07 |  |  |
| Fixed effects: | Estimate | SE | t | **ESTINIT** |
| (Intercept) | 18.67 | 13.26 | 1.41 | **20.84** |
| Altitude 1000 | -2.10 | 6.00 | -0.350 | **-4.24** |
| Altitude 1500 | -10.73 | 6.00 | -1.789 | **-4.57** |
| Altitude 1800 | -9.85 | 6.00 | -1.643 | **-11.68** |
|  |  |  |  |  |
