# Supplementary material for: The functioning of different beetle (Coleoptera) sampling methods across altitudinal gradients in Peninsular Malaysia
Source: PLoS One. 2022 Mar 31;17(3):e0266076. doi: 10.1371/journal.pone.0266076 (PMC8970512; doi:10.1371/journal.pone.0266076)
Supplement: S5 Table — (DOCX) [file pone.0266076.s005.docx]

**S5 Table. Species captured using three collecting methods at the two study mountains; numbers are total catches.**

|  | Fraser's Hill | | |  | Genting Highland | | |
| --- | --- | --- | --- | --- | --- | --- | --- |
| Taxon | Light | Malaise | Pitfall |  | Light | Malaise | Pitfall |
| Abax_sp1 | 0 | 0 | 0 |  | 0 | 1 | 0 |
| Abax_sp2 | 0 | 0 | 0 |  | 0 | 1 | 0 |
| Abelater_sp1 | 0 | 0 | 0 |  | 0 | 1 | 0 |
| Abelater_sp2 | 0 | 0 | 0 |  | 0 | 1 | 0 |
| Acorynus_sp1 | 0 | 0 | 0 |  | 0 | 3 | 0 |
| Acorynus_sp2 | 0 | 1 | 0 |  | 2 | 1 | 0 |
| Actiastes_sp1 | 0 | 0 | 13 |  | 0 | 0 | 0 |
| Aegus_sp | 0 | 1 | 0 |  | 0 | 0 | 0 |
| Aleocharinae_sp1 | 0 | 10 | 0 |  | 0 | 78 | 0 |
| Aleocharinae_sp2 | 0 | 0 | 0 |  | 0 | 6 | 0 |
| Aleocharinae_sp3 | 0 | 2 | 32 |  | 0 | 5 | 0 |
| Altica_sp1 | 0 | 0 | 0 |  | 15 | 0 | 0 |
| Alticinae_sp1 | 3 | 13 | 0 |  | 0 | 0 | 0 |
| Alticinae_sp2 | 6 | 10 | 0 |  | 0 | 0 | 0 |
| Anacentrinus_sp1 | 0 | 0 | 0 |  | 1 | 1 | 0 |
| Anadastus_sp1 | 0 | 0 | 0 |  | 0 | 1 | 0 |
| Anisandrus_sp1 | 3 | 6 | 2 |  | 3 | 0 | 0 |
| Anomala_sp1 | 24 | 14 | 3 |  | 29 | 0 | 0 |
| Anomala_sp2 | 1 | 1 | 0 |  | 17 | 6 | 0 |
| Anomala_sp3 | 0 | 4 | 0 |  | 21 | 12 | 5 |
| Anomala_sp4 | 1 | 0 | 0 |  | 15 | 6 | 0 |
| Anomala_sp5 | 0 | 3 | 0 |  | 8 | 0 | 0 |
| Anomala_sp6 | 0 | 0 | 0 |  | 5 | 0 | 0 |
| Anomala_sp7 | 0 | 2 | 0 |  | 0 | 0 | 0 |
| Anomala_sp8 | 0 | 0 | 0 |  | 2 | 0 | 0 |
| Anotylus_sp1 | 0 | 0 | 0 |  | 1 | 10 | 10 |
| Anotylus_sp2 | 3 | 0 | 27 |  | 0 | 3 | 21 |
| Aphthona_sp1 | 0 | 0 | 0 |  | 0 | 10 | 0 |
| Aphthona_sp2 | 0 | 0 | 0 |  | 1 | 2 | 0 |
| Aphthona_sp3 | 0 | 0 | 0 |  | 0 | 2 | 0 |
| Apogonia_sp1 | 10 | 13 | 1 |  | 36 | 0 | 0 |
| Apogonia_sp2 | 13 | 13 | 3 |  | 35 | 0 | 0 |
| Apogonia_sp3 | 10 | 1 | 0 |  | 12 | 9 | 1 |
| Apogonia_sp4 | 2 | 23 | 0 |  | 13 | 8 | 0 |
| Apogonia_sp5 | 0 | 2 | 0 |  | 23 | 3 | 0 |
| Arrenodes_sp | 1 | 0 | 0 |  | 0 | 0 | 0 |
| Balistica_sp1 | 0 | 0 | 0 |  | 0 | 1 | 0 |
| Bledius_sp1 | 0 | 0 | 14 |  | 0 | 0 | 66 |
| Brachypeplus_sp1 | 7 | 5 | 31 |  | 0 | 25 | 0 |
| Brachypeplus_sp2 | 4 | 4 | 1 |  | 7 | 0 | 4 |
| Brachypeplus_sp3 | 0 | 0 | 0 |  | 13 | 31 | 0 |
| Bradymerus_sp2 | 0 | 0 | 0 |  | 0 | 40 | 0 |
| Byrrhodes_sp1 | 0 | 0 | 0 |  | 0 | 2 | 0 |
| Byrrhodes_sp2 | 0 | 0 | 0 |  | 0 | 2 | 0 |
| Byrrhodes_sp3 | 0 | 0 | 0 |  | 0 | 2 | 0 |
| Byrrhodes_sp4 | 0 | 0 | 0 |  | 0 | 1 | 0 |
| Camptorhinus_scrobicollis | 0 | 0 | 0 |  | 0 | 2 | 0 |
| Carpelimus_sp1 | 0 | 0 | 0 |  | 0 | 1 | 0 |
| Ceram_C | 0 | 0 | 0 |  | 2 | 0 | 0 |
| Cero_A | 0 | 0 | 0 |  | 0 | 0 | 2 |
| Cero_B | 0 | 0 | 0 |  | 0 | 0 | 1 |
| Cerobates_sp1 | 0 | 0 | 0 |  | 1 | 0 | 0 |
| Chelonarium_sp1 | 0 | 0 | 0 |  | 0 | 2 | 0 |
| Chelonarium_sp2 | 0 | 0 | 0 |  | 0 | 3 | 0 |
| Chelonarium_sp3 | 0 | 0 | 0 |  | 0 | 1 | 0 |
| Cicindela_sp1 | 3 | 0 | 0 |  | 4 | 0 | 0 |
| Cicindela_sp2 | 1 | 0 | 0 |  | 10 | 1 | 2 |
| Cicindela_sp3 | 0 | 0 | 0 |  | 2 | 0 | 1 |
| Cleorina_sp | 0 | 0 | 0 |  | 6 | 0 | 0 |
| Cnodalonini | 0 | 1 | 0 |  | 0 | 0 | 0 |
| Colaspoma_sp2 | 19 | 3 | 0 |  | 0 | 0 | 0 |
| Colaspoma_sp4 | 0 | 0 | 0 |  | 2 | 0 | 0 |
| Conoderinae_sp1 | 0 | 0 | 0 |  | 1 | 0 | 0 |
| Conoderinae_sp2 | 0 | 0 | 0 |  | 0 | 1 | 0 |
| Cryptalaus_sp1 | 0 | 0 | 0 |  | 1 | 0 | 0 |
| Cryptalaus_sp2 | 0 | 0 | 0 |  | 0 | 1 | 0 |
| Curcu_A | 8 | 5 | 0 |  | 0 | 0 | 0 |
| Curcu_G | 0 | 0 | 0 |  | 0 | 1 | 0 |
| Curcu_I | 0 | 0 | 0 |  | 0 | 1 | 0 |
| Dryo_A | 0 | 0 | 0 |  | 1 | 1 | 0 |
| Elacatis_sp1 | 0 | 0 | 0 |  | 2 | 0 | 0 |
| Endelus_sp1 | 0 | 0 | 0 |  | 1 | 0 | 0 |
| Endo_B | 0 | 0 | 0 |  | 1 | 0 | 0 |
| Epepeotes_lateralis | 0 | 1 | 0 |  | 4 | 0 | 0 |
| Epicauta_sp2 | 0 | 0 | 0 |  | 1 | 0 | 0 |
| Epuraea_sp1 | 0 | 0 | 0 |  | 0 | 7 | 0 |
| Fornax_sp1 | 0 | 0 | 0 |  | 1 | 0 | 0 |
| Fornax_sp2 | 0 | 0 | 0 |  | 1 | 0 | 0 |
| Galerucinae_sp1 | 5 | 6 | 0 |  | 3 | 16 | 0 |
| Haptonchus_sp1 | 1 | 0 | 0 |  | 0 | 0 | 0 |
| Harpalus_sp1 | 2 | 1 | 92 |  | 2 | 0 | 12 |
| Harpalus_sp2 | 0 | 0 | 24 |  | 0 | 0 | 54 |
| Helluonidius_sp1 | 0 | 0 | 0 |  | 2 | 0 | 0 |
| Helluonidius_sp2 | 0 | 0 | 0 |  | 1 | 0 | 0 |
| Hiletus_sp1 | 0 | 1 | 0 |  | 0 | 0 | 9 |
| Hiletus_sp2 | 0 | 0 | 0 |  | 1 | 1 | 22 |
| Hist_A | 1 | 0 | 0 |  | 0 | 0 | 0 |
| Hopliini_sp2 | 0 | 0 | 0 |  | 2 | 0 | 0 |
| Hoplocerambyx_spinicornis | 6 | 0 | 0 |  | 3 | 0 | 0 |
| Hydrovatus_enigmaticus | 4 | 0 | 13 |  | 7 | 0 | 0 |
| Idiophyes_sp1 | 0 | 0 | 0 |  | 0 | 1 | 0 |
| Illeis_sp2 | 0 | 0 | 2 |  | 14 | 0 | 0 |
| Inopeplus_sp1 | 0 | 0 | 0 |  | 1 | 0 | 70 |
| Ischnosoma_sp1 | 0 | 9 | 0 |  | 0 | 53 | 0 |
| Lampy_B | 0 | 0 | 0 |  | 1 | 0 | 0 |
| Lampy_C | 0 | 0 | 0 |  | 1 | 0 | 0 |
| Lampy_D | 0 | 1 | 0 |  | 0 | 0 | 0 |
| Lebia_sp1 | 0 | 0 | 0 |  | 0 | 0 | 17 |
| Lebia_sp2 | 0 | 0 | 0 |  | 0 | 0 | 30 |
| Lempy_E | 4 | 2 | 0 |  | 0 | 0 | 0 |
| Lispinus_sp1 | 0 | 0 | 7 |  | 0 | 0 | 35 |
| Longitarsus_sp1 | 0 | 0 | 0 |  | 0 | 1 | 0 |
| Longitarsus_sp2 | 0 | 0 | 0 |  | 0 | 4 | 0 |
| Luca_A | 0 | 0 | 0 |  | 1 | 0 | 0 |
| Luca_B | 0 | 1 | 0 |  | 0 | 0 | 0 |
| Luciola_sp1 | 6 | 0 | 0 |  | 0 | 0 | 0 |
| Luciolinae_sp1 | 0 | 0 | 0 |  | 2 | 0 | 0 |
| Luciolinae_sp3 | 3 | 0 | 0 |  | 0 | 0 | 0 |
| Lupropini_sp1 | 1 | 0 | 0 |  | 0 | 0 | 0 |
| Lupropini_sp2 | 0 | 1 | 0 |  | 0 | 0 | 0 |
| Lyci_A | 0 | 0 | 0 |  | 1 | 0 | 0 |
| Lymantor_sp1 | 0 | 3 | 0 |  | 1 | 12 | 0 |
| Lymantor_sp2 | 0 | 11 | 0 |  | 22 | 47 | 0 |
| Lymantor_sp3 | 0 | 5 | 0 |  | 27 | 33 | 0 |
| Lymantor_sp4 | 0 | 3 | 0 |  | 21 | 16 | 0 |
| Macratria_sp1 | 0 | 0 | 0 |  | 1 | 0 | 0 |
| Macratria_sp2 | 0 | 1 | 0 |  | 0 | 0 | 0 |
| Macrotomoderus_sp2 | 0 | 1 | 0 |  | 2 | 0 | 0 |
| Megalodacne_sp1 | 0 | 0 | 0 |  | 1 | 0 | 0 |
| Meloid_A | 4 | 4 | 0 |  | 0 | 0 | 0 |
| Metialma_sp1 | 0 | 0 | 0 |  | 0 | 1 | 0 |
| Metialma_sp2 | 0 | 0 | 0 |  | 0 | 1 | 0 |
| Metriorrhynchus_sp1 | 0 | 0 | 0 |  | 0 | 1 | 0 |
| Microrhagus_sp1 | 0 | 0 | 0 |  | 3 | 0 | 0 |
| Monolepta_sp1 | 0 | 0 | 0 |  | 0 | 2 | 0 |
| Monolepta_sp2 | 0 | 0 | 0 |  | 0 | 2 | 0 |
| Monolepta_sp3 | 0 | 0 | 0 |  | 0 | 2 | 0 |
| Monolepta_sp4 | 0 | 0 | 0 |  | 0 | 2 | 0 |
| Monolepta_sp5 | 0 | 0 | 0 |  | 0 | 1 | 0 |
| Monomma_sp1 | 0 | 0 | 0 |  | 1 | 0 | 0 |
| Monomma_sp2 | 0 | 0 | 0 |  | 1 | 0 | 0 |
| Mordali_B | 0 | 0 | 0 |  | 0 | 1 | 0 |
| Mordali_C | 0 | 0 | 0 |  | 0 | 1 | 0 |
| Mordeli_A | 0 | 0 | 0 |  | 3 | 3 | 0 |
| Mordeli_D | 0 | 0 | 0 |  | 0 | 1 | 0 |
| Mordeli_E | 0 | 0 | 0 |  | 0 | 2 | 0 |
| Mulsanteus_sp1 | 3 | 1 | 0 |  | 2 | 1 | 0 |
| Mulsanteus_sp2 | 0 | 1 | 0 |  | 0 | 0 | 0 |
| Mulsanteus_sp3 | 1 | 1 | 0 |  | 0 | 0 | 0 |
| Myrmex_sp1 | 0 | 0 | 0 |  | 0 | 1 | 0 |
| Neocerambyx_gigas | 1 | 0 | 0 |  | 0 | 0 | 0 |
| Nipponoelater_sp1 | 1 | 0 | 0 |  | 0 | 0 | 0 |
| Nisotra_sp2 | 4 | 5 | 0 |  | 0 | 0 | 0 |
| Niti_C | 0 | 0 | 0 |  | 0 | 2 | 0 |
| Omonadus_sp1 | 0 | 0 | 0 |  | 0 | 0 | 11 |
| Orphnebius_sp1 | 0 | 0 | 28 |  | 0 | 0 | 93 |
| Orphnebius_sp2 | 0 | 0 | 25 |  | 0 | 0 | 31 |
| Orphnebius_sp3 | 0 | 0 | 0 |  | 1 | 0 | 0 |
| Orphninae | 0 | 0 | 0 |  | 0 | 4 | 0 |
| Oxylatus_sp1 | 0 | 0 | 0 |  | 0 | 0 | 59 |
| Paedarus_sp1 | 0 | 0 | 9 |  | 0 | 0 | 90 |
| Paederinae_sp2 | 0 | 0 | 14 |  | 0 | 11 | 0 |
| Paederinae_sp3 | 2 | 0 | 23 |  | 4 | 30 | 0 |
| Passa_A | 1 | 0 | 4 |  | 0 | 0 | 0 |
| Pentagonica_sp1 | 0 | 0 | 0 |  | 1 | 0 | 45 |
| Philonthus_terminipennis | 0 | 0 | 0 |  | 2 | 0 | 0 |
| Phlacaridae_A | 0 | 0 | 0 |  | 3 | 0 | 0 |
| Pityogenes_sp1 | 6 | 10 | 50 |  | 7 | 1 | 3 |
| Pityogenes_sp2 | 4 | 2 | 2 |  | 1 | 0 | 0 |
| Platy_A | 0 | 1 | 0 |  | 0 | 0 | 0 |
| Platypus_sp1 | 0 | 0 | 0 |  | 1 | 0 | 0 |
| Poecilips_variabilis | 0 | 0 | 0 |  | 0 | 2 | 0 |
| Pterostichus_sp1 | 0 | 1 | 12 |  | 0 | 0 | 62 |
| Pterostichus_sp2 | 0 | 0 | 23 |  | 0 | 0 | 84 |
| Pterostichus_sp3 | 3 | 6 | 18 |  | 0 | 0 | 55 |
| Ptilo_A | 0 | 0 | 0 |  | 1 | 0 | 0 |
| Rhipi_B | 0 | 3 | 0 |  | 0 | 0 | 0 |
| Sarmydus_antennatus | 2 | 0 | 0 |  | 0 | 0 | 0 |
| Sarmydus_sp1 | 4 | 3 | 0 |  | 0 | 0 | 0 |
| Scara_M | 0 | 5 | 0 |  | 6 | 0 | 0 |
| Scarab_O | 0 | 0 | 0 |  | 3 | 0 | 0 |
| Scarab_P | 1 | 0 | 0 |  | 5 | 0 | 0 |
| Scoly_E | 1 | 0 | 0 |  | 0 | 0 | 0 |
| Scyd_A | 0 | 0 | 0 |  | 0 | 2 | 0 |
| Scymnus_sp1 | 0 | 0 | 0 |  | 0 | 2 | 0 |
| Scymnus_sp2 | 0 | 0 | 0 |  | 0 | 1 | 0 |
| Sepedophilus_sp1 | 0 | 0 | 0 |  | 0 | 1 | 0 |
| Silvanus_sp1 | 0 | 0 | 0 |  | 0 | 2 | 0 |
| Sinoxylon_sp1 | 0 | 0 | 0 |  | 0 | 43 | 0 |
| Spinolyprops_A | 0 | 1 | 0 |  | 1 | 0 | 25 |
| Staphy_C | 0 | 0 | 0 |  | 0 | 0 | 41 |
| Staphy_M | 0 | 0 | 0 |  | 0 | 0 | 30 |
| Stenus_sp1 | 0 | 0 | 0 |  | 1 | 1 | 0 |
| Stenus_sp2 | 0 | 0 | 0 |  | 0 | 3 | 0 |
| Stigmatium_sp1 | 0 | 0 | 0 |  | 0 | 3 | 0 |
| Strotocera_sp1 | 11 | 9 | 0 |  | 0 | 0 | 0 |
| Strotocera_sp2 | 0 | 0 | 0 |  | 1 | 0 | 0 |
| Sunius_sp1 | 0 | 0 | 18 |  | 0 | 0 | 0 |
| Theopea_impressa | 0 | 0 | 0 |  | 1 | 2 | 0 |
| Tolidopalpus_sp1 | 0 | 0 | 0 |  | 0 | 2 | 0 |
| Tomoderus_sp1 | 0 | 0 | 0 |  | 2 | 1 | 0 |
| Tomoderus_sp2 | 0 | 1 | 0 |  | 1 | 0 | 0 |
| Trichochrysea_sp1 | 1 | 1 | 0 |  | 0 | 0 | 0 |
| Xyleborus_sp1 | 0 | 0 | 0 |  | 0 | 24 | 0 |
| Xyleborus_sp2 | 0 | 0 | 0 |  | 0 | 6 | 0 |
| Xylocleptes_sp2 | 0 | 0 | 0 |  | 0 | 1 | 0 |
| Xylocleptes_sp3 | 0 | 0 | 0 |  | 0 | 1 | 0 |
| Xylothrips_sp1 | 0 | 0 | 0 |  | 0 | 5 | 0 |
