# Supplementary material for: The functioning of different beetle (Coleoptera) sampling methods across altitudinal gradients in Peninsular Malaysia
Source: PLoS One. 2022 Mar 31;17(3):e0266076. doi: 10.1371/journal.pone.0266076 (PMC8970512; doi:10.1371/journal.pone.0266076)
Supplement: S6 Table — Light gray: species shared between light and Malaise traps; dark gray: species shared with pitfall, light and Malaise traps. (DOCX) [file pone.0266076.s006.docx]

**S6 Table. Twenty most abundantly captured species in our data, sorted according to trap-type specific sample sizes; n indicates number of individuals in the data.** Light gray: species shared between light and Malaise traps; dark gray: species shared with pitfall, light and Malaise traps.

| Light | n |  | Malaise | n |  | Pitfall | n |
| --- | --- | --- | --- | --- | --- | --- | --- |
| Anomala_sp1 | 53 |  | Aleocharinae_sp1 | 88 |  | Orphnebius_sp1 | 121 |
| Apogonia_sp2 | 48 |  | Ischnosoma_sp1 | 62 |  | Pterostichus_sp2 | 107 |
| Apogonia_sp1 | 46 |  | Lymantor_sp2 | 58 |  | Harpalus_sp1 | 104 |
| Lymantor_sp3 | 27 |  | Sinoxylon_sp1 | 43 |  | Paedarus_sp1 | 99 |
| Apogonia_sp5 | 23 |  | Bradymerus_sp2 | 40 |  | Bledius_sp1 | 80 |
| Apogonia_sp3 | 22 |  | Lymantor_sp3 | 38 |  | Harpalus_sp2 | 78 |
| Lymantor_sp2 | 22 |  | Apogonia_sp4 | 31 |  | Pterostichus_sp1 | 74 |
| Anomala_sp3 | 21 |  | Brachypeplus_sp3 | 31 |  | Pterostichus_sp3 | 73 |
| Lymantor_sp4 | 21 |  | Brachypeplus_sp1 | 30 |  | Inopeplus_sp1 | 70 |
| Colaspoma_sp2 | 19 |  | Paederinae_sp3 | 30 |  | Oxylatus_sp1 | 59 |
| Anomala_sp2 | 18 |  | Xyleborus_sp1 | 24 |  | Orphnebius_sp2 | 56 |
| Anomala_sp4 | 16 |  | Galerucinae_sp1 | 22 |  | Pityogenes_sp1 | 53 |
| Altica_sp1 | 15 |  | Lymantor_sp4 | 19 |  | Anotylus_sp2 | 48 |
| Apogonia_sp4 | 15 |  | Anomala_sp3 | 16 |  | Pentagonica_sp1 | 45 |
| Illeis_sp2 | 14 |  | Lymantor_sp1 | 15 |  | Lispinus_sp1 | 42 |
| Brachypeplus_sp3 | 13 |  | Anomala_sp1 | 14 |  | Staphy_C | 41 |
| Pityogenes_sp1 | 13 |  | Apogonia_sp2 | 13 |  | Aleocharinae_sp3 | 32 |
| Brachypeplus_sp2 | 11 |  | Apogonia_sp1 | 13 |  | Brachypeplus_sp1 | 31 |
| Cicindela_sp2 | 11 |  | Alticinae_sp1 | 13 |  | Lebia_sp2 | 30 |
| Hydrovatus_enigmaticus | 11 |  | Pityogenes_sp1 | 11 |  | Staphy_M | 30 |
