# Supplementary material for: The functioning of different beetle (Coleoptera) sampling methods across altitudinal gradients in Peninsular Malaysia
Source: PLoS One. 2022 Mar 31;17(3):e0266076. doi: 10.1371/journal.pone.0266076 (PMC8970512; doi:10.1371/journal.pone.0266076)
Supplement: S1 Fig — Different rows show plots for the number of species, rarefaction standardized richness based on 5, 10 or 20 individuals, and coverage-based (CB) rarefied asymptotic richness. Solid line shows perfect fit, dash lines show 95% confidence intervals. Ideally, all sample residuals should fall between confidence intervals. (DOCX) [file pone.0266076.s007.docx]

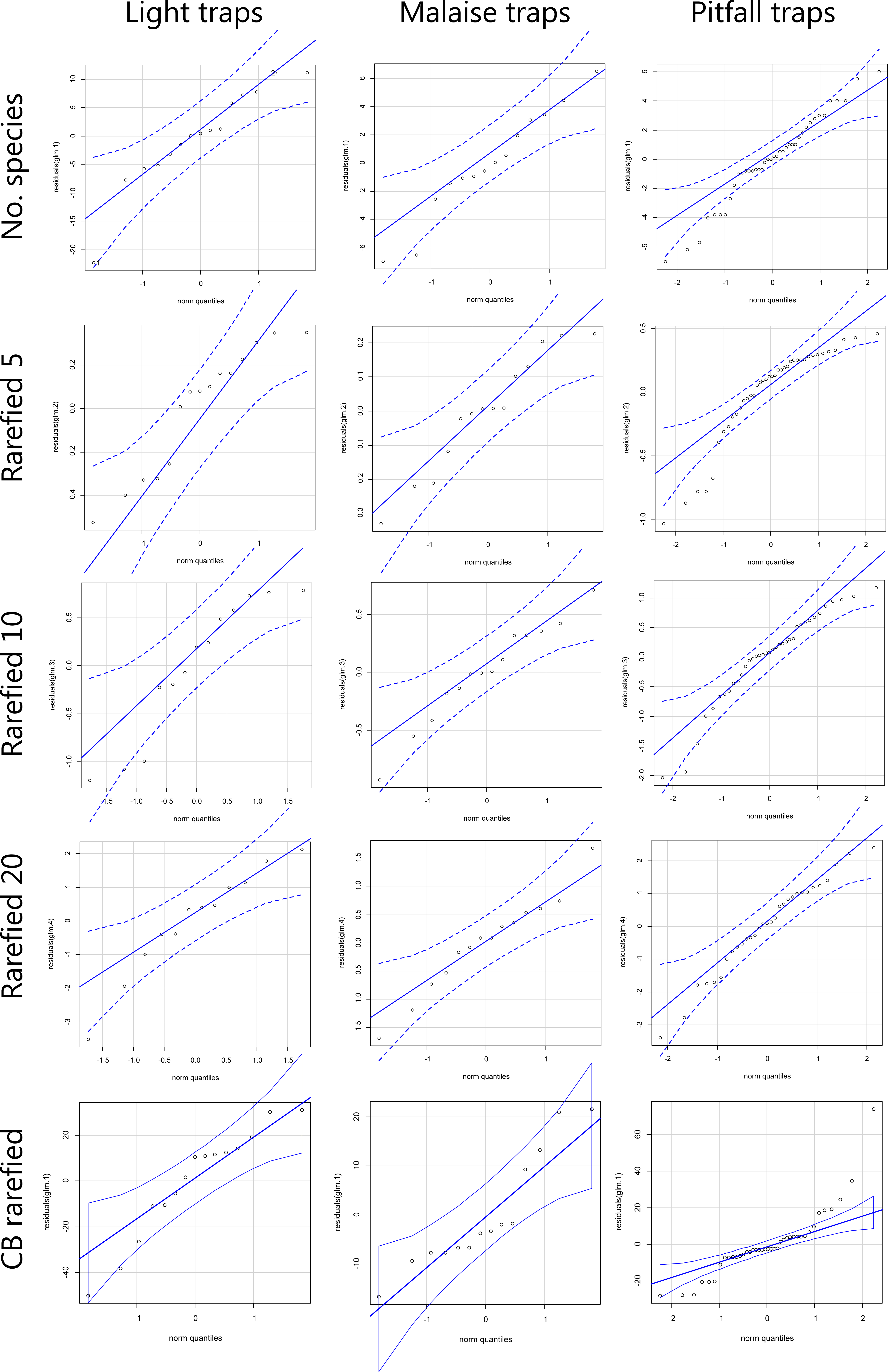


**S1 Fig. Q-Q plots for GLMM residuals, shown separately for three sampling methods (compare S1 Table).** Different rows show plots for the number of species, rarefaction standardized richness based on 5, 10 or 20 individuals, and coverage-based (CB) rarefied asymptotic richness. Solid line shows perfect fit, dash lines show 95% confidence intervals. Ideally, all sample residuals should fall between confidence intervals.
