# Supplementary material for: The functioning of different beetle (Coleoptera) sampling methods across altitudinal gradients in Peninsular Malaysia
Source: PLoS One. 2022 Mar 31;17(3):e0266076. doi: 10.1371/journal.pone.0266076 (PMC8970512; doi:10.1371/journal.pone.0266076)
Supplement: Attachment — Submitted filename: PONE-D-21-03456_reviewer.pdf [file pone.0266076.s008.pdf]

PLOS ONE

Community composition and indicator functioning of beetles across environmental gradients in Peninsular Malaysia

--Manuscript Draft--

**Manuscript Number:**	PONE-D-21-03456
**Article Type:**	Research Article
**Full Title:**	Community composition and indicator functioning of beetles across environmental gradients in Peninsular Malaysia
**Short Title:**	Beetle Diversity across elevations in Malaysia
**Corresponding Author:**	Muneeb M. No Middle Name Musthafa, PhD<br>South Eastern University of Sri Lanka<br>Oluvil, Ampara SRI LANKA
**Keywords:**	Altitude; biodiversity; forest; mountain; tropical
**Abstract:**	Biodiversity research rely largely on knowledge about species responses to environmental gradients, assessed using some commonly applied sampling method. However, the consistency of detected responses using different sampling methods, and thus the generality of findings, has seldom been assessed in tropical ecosystems. We hypothesize that the beetles can be good indicators in tropical montane ecosystems. We studied the response consistency and indicator functioning of beetle assemblages to altitudinal gradients in Malaysia, using different traps. The beetle data were analyzed using PERMANOVA, generalized linear mixed-effects models (GLMM), non-metric multidimensional scaling (NMDS), multivariate regression trees (MRT) and indicator species analysis (IndVal). The collection was resulted in 198 morpho-species of beetles representing 32 families with the total number of 3052 individual beetles. PERMANOVA indicated a significant correlation between beetle species composition and the tested elevations. GLMM showed that the two mountains differed considerably in pitfall but less so in light or Malaise traps. NMDS and MRT showed light traps is distinguished at 1,800 m from lower altitudes. IndVal showed low altitudes had about twice as many indicators as middle or high altitudes. These results suggest that the detection of community variation in the environment depends on sampling methods, but also on analytical approach.
**Order of Authors:**	Muneeb M. No Middle Name Musthafa, PhD
	Fauziah Abdullah
	Matti J. Koivula
**Additional Information:**	
**Question**	**Response**
**Financial Disclosure**<br><br>Enter a financial disclosure statement that describes the sources of funding for the work included in this submission. Review the submission guidelines for detailed requirements. View published research articles from *PLOS ONE* for specific examples.<br><br>This statement is required for submission and **will appear in the published article** if the submission is accepted. Please make sure it is accurate.	We thank the University of Malaya for financing this study (grant numbers RP004E/13SUS and PG059/2014B).

Powered by Editorial Manager® and ProduXion Manager® from Aries Systems Corporation

**Unfunded studies**<br>Enter: *The author(s) received no specific funding for this work.*<br><br>**Funded studies**<br>Enter a statement with the following details:<br>• Initials of the authors who received each award<br>• Grant numbers awarded to each author<br>• The full name of each funder<br>• URL of each funder website<br>• Did the sponsors or funders play any role in the study design, data collection and analysis, decision to publish, or preparation of the manuscript?<br>• **NO** - Include this sentence at the end of your statement: *The funders had no role in study design, data collection and analysis, decision to publish, or preparation of the manuscript.*<br>• **YES** - Specify the role(s) played.<br><br>* typeset	
**Competing Interests**<br><br>Use the instructions below to enter a competing interest statement for this submission. On behalf of all authors, disclose any competing interests that could be perceived to bias this work—acknowledging all financial support and any other relevant financial or non-financial competing interests.<br><br>This statement is required for submission and **will appear in the published article** if the submission is accepted. Please make sure it is accurate and that any funding sources listed in your Funding Information later in the submission form are also declared in your Financial Disclosure statement.<br><br>View published research articles from *PLOS ONE* for specific examples.	NO authors have competing interests

**NO authors have competing interests**<br><br>Enter: *The authors have declared that no competing interests exist.*<br><br>**Authors with competing interests**<br><br>Enter competing interest details beginning with this statement:<br><br>*I have read the journal's policy and the authors of this manuscript have the following competing interests: [insert competing interests here]*<br><br>* typeset	
**Ethics Statement**<br><br>Enter an ethics statement for this submission. This statement is required if the study involved:<br><br>• Human participants<br>• Human specimens or tissue<br>• Vertebrate animals or cephalopods<br>• Vertebrate embryos or tissues<br>• Field research<br><br>Write "N/A" if the submission does not require an ethics statement.<br><br>General guidance is provided below. Consult the submission guidelines for detailed instructions. **Make sure that all information entered here is included in the Methods section of the manuscript.**	N/A

**Format for specific study types**<br><br>**Human Subject Research (involving human participants and/or tissue)**<br>• Give the name of the institutional review board or ethics committee that approved the study<br>• Include the approval number and/or a statement indicating approval of this research<br>• Indicate the form of consent obtained (written/oral) or the reason that consent was not obtained (e.g. the data were analyzed anonymously)<br><br>**Animal Research (involving vertebrate animals, embryos or tissues)**<br>• Provide the name of the Institutional Animal Care and Use Committee (IACUC) or other relevant ethics board that reviewed the study protocol, and indicate whether they approved this research or granted a formal waiver of ethical approval<br>• Include an approval number if one was obtained<br>• If the study involved *non-human primates*, add *additional details* about animal welfare and steps taken to ameliorate suffering<br>• If anesthesia, euthanasia, or any kind of animal sacrifice is part of the study, include briefly which substances and/or methods were applied<br><br>**Field Research**<br><br>Include the following details if this study involves the collection of plant, animal, or other materials from a natural setting:<br>• Field permit number<br>• Name of the institution or relevant body that granted permission	
**Data Availability**<br><br>Authors are required to make all data underlying the findings described fully available, without restriction, and from the time of publication. PLOS allows rare exceptions to address legal and ethical concerns. See the PLOS Data Policy and FAQ for detailed information.	No - some restrictions will apply

A Data Availability Statement describing where the data can be found is required at submission. Your answers to this question constitute the Data Availability Statement and **will be published in the article**, if accepted.<br><br>**Important:** Stating 'data available on request from the author' is not sufficient. If your data are only available upon request, select 'No' for the first question and explain your exceptional situation in the text box.<br><br>Do the authors confirm that all data underlying the findings described in their manuscript are fully available without restriction?	
**Describe where the data may be found in full sentences. If you are copying our sample text, replace any instances of XXX with the appropriate details.**<br><br>• If the data are **held or will be held in a public repository**, include URLs, accession numbers or DOIs. If this information will only be available after acceptance, indicate this by ticking the box below. For example: *All XXX files are available from the XXX database (accession number(s) XXX, XXX.).*<br>• If the data are all contained **within the manuscript and/or Supporting Information files**, enter the following: *All relevant data are within the manuscript and its Supporting Information files.*<br>• If neither of these applies but you are able to provide **details of access elsewhere**, with or without limitations, please do so. For example:<br><br>*Data cannot be shared publicly because of [XXX]. Data are available from the XXX Institutional Data Access / Ethics Committee (contact via XXX) for researchers who meet the criteria for access to confidential data.*<br><br>*The data underlying the results presented in the study are available from (include the name of the third party*	N/A

and contact information or URL).<br>• This text is appropriate if the data are owned by a third party and authors do not have permission to share the data.<br><br>* typeset	
Additional data availability information:	Tick here if the URLs/accession numbers/DOIs will be available only after acceptance of the manuscript for publication so that we can ensure their inclusion before publication.

Powered by Editorial Manager® and ProduXion Manager® from Aries Systems Corporation

Manuscript

Click here to access/download;Manuscript;Community structure of beetles for PLOS ONE.docx

Full Title:

Community composition and indicator functioning of beetles across environmental gradients in Peninsular Malaysia

Short Title: Beetle Diversity across elevations in Malaysia

Muneeb M. Musthafa[1,2,3 *], Fauziah Abdullah[2,3,4] and Matti J. Koivula[5]

[1]Department of Biosystems Technology, Faculty of Technology, South Eastern University of Sri Lanka, University Park, Oluvil, #32360, Sri Lanka.

[2]Institute of Biological Science, Faculty of Science, University of Malaya, 50603, Kuala Lumpur, Malaysia.

[3]B513, Toxicology Lab, Institute of Postgraduate Studies, University Malaya, 50603, Kuala Lumpur, Malaysia.

[4]Center of Biotechnology in Agriculture, University Malaya, 50603, Kuala Lumpur, Malaysia

[5]School of Forest Sciences, University of Eastern Finland, P. O. Box 111, FI-80101 Joensuu, Finland.

* Corresponding author: muneeb@seu.ac.lk

Author contributions

Muneeb M. Musthafa: Field sample collection, data curation and drafting the manuscript.

Fauziah Abdullah: Conceptualization of the research and designed the methodology.

Matti J. Koivula: Application of statistical analysis and finalize the manuscript.

Abstract

Biodiversity research rely largely on knowledge about species responses to environmental gradients, assessed using some commonly applied sampling method. However, the consistency of detected responses using different sampling methods, and thus the generality of findings, has seldom been assessed in tropical ecosystems. We hypothesize that the beetles can be good indicators in tropical montane ecosystems. We studied the response consistency and indicator functioning of beetle assemblages ~~to~~ in altitudinal gradients ~~in~~ from Malaysia, using different traps. The beetle data were analyzed using PERMANOVA, generalized linear mixed-effects models (GLMM), non-metric multidimensional scaling (NMDS), multivariate regression trees (MRT) and indicator species analysis (IndVal). The collection was resulted in 198 morpho-species of beetles representing 32 families with the total number of 3052 individual beetles. PERMANOVA indicated a significant correlation between beetle species composition and the tested elevations. GLMM showed that the two mountains differed considerably in pitfall but less so in light or Malaise traps. NMDS and MRT showed light traps is distinguished at 1,800 m from lower altitudes. IndVal showed low altitudes had about twice as many indicators as middle or high altitudes. These results suggest that the detection of community variation in the environment depends on sampling methods, but also on analytical approach.

Key words: Altitude, biodiversity, forest, mountain, tropical.

Introduction

Most global biodiversity hotspots and the highest overall species can be found in tropical regions (Mittermeier et al. 2011) and are thus of central importance for conservation. Tropical species often have limited distributions, particularly those that occupy higher altitudes at mountain slopes

(Cox et al. 2016); these species are also sometimes such that may not be able to move elsewhere if conditions turn unfavorable for them due to, for example, habitat destruction or climate change (Escobar et al., 2005; Elsen & Tingley 2015). Hence, studies on mountain species communities provide crucial information for global conservation efforts, in addition to these environments being particularly vulnerable to habitat degradation and alterations in landscape structure (Körner 2003; Rangwala & Miller 2012). Empirical information about species responses to climate change remains scarce, but studies comparing different altitudes may provide vital cues on their responses to climate change (Chen et al. 2009; Ashton et al. 2015, 2016; Musthafa et al. 2018; Musthafa & Abdullah 2019).

Community composition – species, their relative shares, and interactions (biotic responses)– largely determines biodiversity responses to environmental variation and alterations. Communities also vary according to, among other aspects, latitude and altitude (Hooper et al. 2005; Ashton et al. 2015; Bouzan et al. 2015). Our understanding on this variation relies on various taxonomic groups and associated sampling methods; insects, for instance, are commonly sampled using Flight Intercept Traps (FIT), light, Malaise or pitfall traps (e.g., Grootaert et al. 2010). Land-use or conservation planning may sometimes be based on only a handful of taxa or sampling methods. However, earlier research indicates that the three mentioned trap types differ in their efficiency in capturing specimens and species (e.g., Hansen 1988; Ulyshen et al. 2005; Devigne & De Biseau 2014) though this could vary with the composition of the local species community determined by, for example, habitat type and geographic region. A[illegible]ifferent methods capture partly different species (e.g., Missa et al. 2009), their use may, at least theoretically, result in different conclusions and hence applications in land use or conservation. However, sampling methods have seldom been compared from this viewpoint, which is central in the present paper.

The location of the present study, Malaysia, belongs to the Sundaland global biodiversity hotspot (Myers et al. 2000) in South-Eastern Asia. Little is known about invertebrate diversity in Peninsular Malaysian mountains that contain the largest cloud forests in the region (Doll et al. 2014). Our beetle data have earlier been used to study variation in beetle richness at mountain slopes (Musthafa et al. 2018; Musthafa & Abdullah 2019). The objective of the present study is however, is methodological: we assess the consistency of light, Malaise and pitfall trap samples in reflecting a simple environmental gradient, altitude for biodiversity monitoring purposes. More specifically, we compare the three sampling methods across the altitudinal gradient from three viewpoints:

(1) Assemblage dominance structure and species turnover

(2) Assemblage composition

(3) Species associated with different combinations of sampling method and altitude

Materials and Methods

Study sites

The Titiwangsa mountain range dominates the landscape of Peninsular Malaysia. Within this range, we sampled beetles at Fraser's Hill (3°43’ N, 101°45’ E) and Genting Highland (3°25' N, 101°47' E) that are 95 km apart (Fig. 1). Fraser’s Hill mountain tops peak at between 1,000 and 1,800 m a.s.l., whereas the Genting Highland peaks at about 1,800 m a.s.l. In this region, wet and dry seasons cannot be differentiated, as the annual rainfall of 1,800–3,500 mm is distributed throughout the year (Cheong 2013; Ya’cob et al. 2016).

well preserved forest fragment by

Fraser’s Hill, locally known as Bukit Fraser, is a pristine, permanently protected nature reserve located at the Raub district of Pahang state. This mountain was formed 250 million years ago by the molten magma that extruded upon the earth crust as a result of tectonic activities (Cheong,

2013). Fraser’s Hill has been developed as a hill station dating back in 1919 (Freeman 1999), where 90% of the 2,800-ha land area is covered by forests (Kiew, 1998; Cheong, 2013). Fraser’s Hill comprises residential areas, commercial complexes, community services and recreational facilities. The forests mainly consist of tropical montane cloud forests. Lower montane forests –, about 500–1,200 m a.s.l. – are dominated by Fagaceae and Lauraceae trees, whereas upper montane forests – from about 1,200 to 1,800 m a.s.l. – are dominated by Coniferae, Ericaceae and Myrtaceae trees (Perumal & Lo, 1998).

Genting Highland is perhaps the most disturbed cloud forest in the Malaysian mountains. The summit is covered by amusement parks, casinos and hotels (Peh et al. 2011). The highest peaks at Genting Highland reach 1,800 m a.s.l, where 96% of the total of 3,965 ha of land is still covered with mostly primary forests virgin forests (Cheong 2013). Before Genting Highland became an entertainment site, it was undisturbed forest that could be reached only via jungle trekking (Piggott 1977; Stone 1981).

Sampling Methods

We sampled beetles at 500 m, 1,000 m, 1,500 m and 1,800 m a.s.l. using two Malaise traps, two light traps and five groups of five pitfall traps at each altitude at both mountain slopes in October 2014, March 2015, June 2015 and September 2015 at each time the sampling was replicated six times at each elevation. All the traps were fixed at least 150 m apart at a given altitude and were placed at least 150 m apart and from the nearest main road, where all sampling methods were used at all elevations. These roads and a river split the sampled forests into a total of twenty distinctive stands (Fig. 1).

Malaise traps consisted of a nylon net connected to a collection jar, half filled with 70% ethanol and attached to a tree branch about one meter above the ground. Pitfall traps were plastic cups

(diameter 65 mm, depth 95 mm) partly filled with 70% ethanol and dug into the ground with the rim flush with soil surface. We placed large dry leaves above each trap to protect them from litter and rain. At each collecting date, Malaise and pitfall traps were set for 24 hours, starting at 08:00 AM. Light traps were made of mosquito netting with a 160-watt mercury bulb connected to a portable Honda EU10i portable generator. At each collecting date, the light traps operated from 18:00 to 23:30, and beetles were collected manually from the traps using collection bottles and aspirators. We occasionally continued to use the light traps until the next morning until 06:00, but no beetles were captured after midnight.

Identification of specimens

We sorted, summed and cross-checked beetle samples using available keys (Saito 2003; Mohamedsaid 2004; Naomi 2006; Schawaller 2006, 2012; Schimmel & Tarnawski 2007; Assing 2008; Hlaváč et al. 2011; Prathapan & Viraktamath 2011; Ryvkin 2011; Shi et al. 2013; Filippini et al. 2016; Anzaldo 2017; Makranczy 2017; Sasakawa et al. 2017). We confirmed the identification of difficult specimens at collections of the Wildlife Department of Malaysia, University of Malaya, National University of Malaysia, and Forestry Department of Malaysia. The identification of beetles to species is hampered by the lack of experts and species compilations. In the present study, the samples contained at least five species new to science, which will be described in later papers. Clearly, the often-broad taxonomic levels and the shortage of knowledge about their ecological traits must be acknowledged while interpreting results, as similar-looking species might be different in terms of, for example, life cycles, diets, abilities to disperse, and habitat requirements.

Statistical Analysis

We had initially 72 samples (16 for light, 16 for Malaise and 40 for pitfall traps). Three samples, however, produced only 1-2 species and were excluded due to difficulty in calculating pair-wise dissimilarities and richness estimates (one light and two Malaise trap samples). Thus, we ran all analyses with 69 samples (Table 1). We ran the following analyses for the four viewpoints as proposed above.

general comment for analysis

To assess sampling-method dependent variation in species, we subjected the beetle data to a generalized linear mixed-effects model (GLMM; Zuur et al. 2009) to quantify variation according to Altitude (500, 1,000, 1,500 or 1,800 m a.s.l.). We ran the model separately for each sampling method (light, Malaise or pitfall traps; hereafter "Method" for brevity) to assess their similarity in responses to Altitude. We subjected the observed species (number of species) and richness estimates based on rarefaction standardization to 5, 10 and 20 individuals, to a GLMM with Altitude as a fixed factor and Mountain (Fraser's Hill or Genting Highland; Fig. 1, Table 1) as a random factor (t[illegible]count for possible spatial autocorrelation), and assumed a Gaussian error distribution. We checked the normality of residuals using Q-Q plots (Supplementary Figure 1). These plots did not indicate major departures from normality, except in the pitfall trap data for the number of species and rarefied richness to 5 individuals. We attempted to solve these by square-root and log transforming the data, and by applying a quasi-Poisson error distribution on untransformed data (Supplementary Figure 2). The quasi-Poisson error distribution improved the model fit for the number of species but not for the rarefied richness. Nevertheless, as the rarefied richness to 10 and 20 individuals produced broadly similar results, we are confident that our initial analyses are robust.

To examine beetle dominance structure and possible turnover according to Altitude and Method, we calculated averages and standard errors for each Altitude for 20 most abundant species per

Method. We plotted these values according to Altitude, using the rank order of the total Method-specific data. As a simple measure of turnover between Methods, we indicated in these plots species that were shared with 1–2 other Methods.

PERMANOVA analysis for Fraser's Hill and Genting Highland was conducted to assess the differences in species composition between each pairwise comparisons. Moreover, to examine beetle assemblage composition across Methods and Altitudes, we used two analyses. Firstly, we used non-metric multidimensional scaling (NMDS; Borg & Groenen 2005) to assess variation in community composition. We used Method-specific data sets by applying a Bray-Curtis dissimilarity matrix. We evaluated the result by comparing the sample scatters based on Altitude, using permutational multivariate ANOVA (the adonis function in R package vegan, with Mountain [Fraser's Hill or Genting Highland] as strata; Oksanen et al. 2018). Secondly, we subjected the Method-specific beetle data sets to multivariate regression tree analyses (MRT; De'ath 2002) based on the Bray-Curtis dissimilarity matrix. We used Altitude and, as our earlier research has indicated differences in beetle faunas between Fraser's Hill and Genting Highland (Musthafa et al. 2018), Mountain as explanatory variables. MRT identifies groups of samples as defined by explanatory variables (Mountain and Altitude) and is not restricted by non-linearities (De'ath 2002). We present the result as a tree of dichotomies, where each dichotomy is based on minimizing the dissimilarity of samples within each tree branch. We report the tree with the lowest cross-validated relative error, following the 1-SE rule (Breiman et al. 2017). Cross-validated relative error provides a better estimate than relative error for the predictive accuracy of the MRT for a new dataset (De'ath 2002).

To detect species characteristic to particular combinations of Method and Altitude, we calculated an indicator value (IndVal; Dufrêne & Legendre 1997, De Cáceres et al. 2010) for each species,

based on all logical combinations of Method and Altitude. Here, we allowed each Method to appear singly or jointly with 1–2 other Methods, whereas for Altitude we only considered single, or combinations of subsequent, Altitudes. Thus, for example a combination 500 + 1000 m was considered but not 500 + 1800 m. We restricted the IndVal to species with the total sample of at least five individuals.

We ran the analyses using R 3.6.1 software (R Core Team, 2019) with lme4 (Bates et al. 2015), vegan (Oksanen et al. 2018), mvpart (Therneau et al. 2014), indicspecies (De Cáceres & Jansen 2016) and car (Fox & Weisberg 2018) packages.

Results

The collection was resulted in 198 morpho-species of beetles representing 32 families with the total number of 3052 individual beetles. Nine were identified to species, 143 to genus, and 43 to higher taxonomic levels. We refer to all these as "species" below for convenience. Regarding Method, we collected 107 species using light traps, 127 using Malaise and 45 using pitfall traps. A total of 135 species were collected with only one Method, whereas 45 were captured with two and 18 with all three sampling Methods. Altogether 98 species were singletons or doubletons, whereas 84 were represented by at least five indiv[illegible]ls (Appendix Table 1). Species accumulation curves at both localities showed a progressive asymptote, suggesting the sample collection was satisfactory as suggested by Chao & Jost (2012).

The GLMM performed rather similarly in terms of the four measures of richness, but the three Methods produced partly different results (Table 2, Fig. 2; Supplementary materials, Table 1). The Mountains differed little in terms of light and Malaise trap data but remarkably in pitfall-trap data. The richness measures based on light traps declined with Altitude but only 1,800 m differed

significantly from 500 m. Decline with Altitude occurred also in Malaise traps so that all higher Altitudes differed significantly from 500 m. Richness measures based on pitfall traps, on the other hand, peaked at 1,000 m (Table 2, Fig. 2).

Assemblage structure and species turnover

The rank-abundance plots of twenty most abundant species (Fig. 3) reflected relatively high similarity between light and Malaise traps, with nine of the 20 most numerous species being shared (grey columns), whereas only one of the 20 dominant species in pitfall-trap samples was shared with light- and none with Malaise-trap samples (white columns). Moreover, the plots suggested more abundance or occurrence changes with Altitude in Malaise and particularly in light traps, whereas the plots varied less and more erratically in pitfall-trap samples (Fig. 3). Another striking pattern in light-trap samples (Fig. 3, left) was the lack of the 12 most abundant species at 1,800 m. Although the three Methods are difficult to compare due to different sampling efforts, species pools and perhaps also capturing efficiencies, only 9% of light-trap species and 17% of Malaise-trap species were found in both 500 m and 1,800 m, whereas 63% of species in pitfall-trap samples were found in both of these Altitudes.

Assemblage composition

The PERMANOVA pairwise tests indicated that there is a significant difference among the compositions between the elevations at Fraser's Hill (SS_{total}=210.75; $SS_{within\text{-}group}$=189.6; F=16.86, p<0.0001). Likewise, the beetle composition at Genting Highland between gradient was significant (SS_{total}=210.2; $SS_{within\text{-}group}$=194.9; F=12.05, p=0.0001) and similarly, all the pairwise comparisons were significantly different for both localities (Table 3).

According to NMDS, samples in light and Malaise traps distinguished 1,800 m from the three lower Altitudes, whereas pitfall trap samples formed two sample clusters, of which only the other distinguished clearly between Altitudes (Fig. 4). Thus, in pitfall-trap samples, Altitude was reflected in Fraser's Hill but not in Genting Highland (on the left and right, respectively, in Fig. 4). Permutational ANOVA, after accounting for variation between the Mountains, supported this visual inspection (Table 4). In light traps, only 1,800 m differed significantly from the other Altitudes; however, all except one combination of 2–3 A[illegible]udes also differed significantly from the rest of the Altitudes. The patterns were similar for Malaise traps, except that different combinations of 2–3 Altitudes differed less commonly from the rest of the Altitudes. Regarding pitfall traps, then, all Altitudes and their combinations (except for 1,000–1,500 m) had distinctive assemblages (Table 4).

MRT for light-trap data constantly resulted in a one-dichotomy tree (Fig. 4). The only division, where 1,800 m was split from the rest of the Altitudes, explained 47% of variation in light-trap data, the former being species poor according to branch-specific rank-abundance plots (Fig. 4). Malaise-trap data, on the other hand, suggested that the same dichotomy, based on Altitude, was apparent in Fraser's Hill but not in Genting Highland; again, 1,800 m had fewer species though this was based on only two samples. This two-dichotomy tree explained 81% of variation in the Malaise trap data (Fig. 4). Also, pitfall trap data suggested that Altitudes could be distinguished at Fraser's Hill but not at Genting highland; here, 1,500 and 1,800 m diverged from the two lower Altitudes that, in turn, were split in the third dichotomy (Fig. 4).

Species associated with different combinations of sampling method and altitude

We found 83 indicator species for different combinations of Method and Altitude (Table 5). Thirteen species were commonly caught with two sampling methods (12 for light and Malaise

traps, and one with Malaise and pitfall traps). Moreover, eight species indicated pitfall traps across the full Altitudinal range (500-1,800 m), whereas we did not detect such altitude-independent indicators for the two other sampling methods. Light traps produced 12 indicators of low (500-1,000 m), six of middle (1,000-1,500 m) and seven of high (1,500-1,800 m) Altitudes, whereas the respective numbers were 16, four and six for Malaise and ten, eleven and five for pitfall traps (Table 5). Moreover, three species occurred in pitfall traps across a wider Altitudinal range: two species at 500-1,500 m and one at 1,000-1,800 m.

Discussion

Our rank-abundance plots for the most abundant species across the altitudinal gradient (Fig. 3) demonstrated two important aspects. Light and Malaise trap samples shared about half of the abundant species, whereas pitfall trap samples were distinctive in this respect. Light and Malaise traps may have attracted a shared pool of species that flies actively, whereas pitfall traps capture mostly ground dwellers that seldom fall into the two other types of trap (e.g., Missa et al. 2009). Secondly, at 1,800 m light (but not Malaise or pitfall) trap samples lost all 12 most abundant species of the total light-trap sample. This loss might have resulted from the captured species pool being more sensitive to altitude-associated changes in abiotic or biotic conditions. However, some of these species were still captured at 1,800 m using Malaise traps, so another explanation may be trap functioning. Perhaps light or wind conditions were different at the highest altitudes, which might in turn have impacted the visibility of light traps or flight activity of many species. The influence of light, temperature, wind direction, and wind direction during the light trapping for insect sampling had been discussed (McGeachie 1989; Sheikh et al. 2016; Nowinszky & Puskás 2017). Moreover, the Malaise trap collection is also influenced by several environmental factors (Mathews & Mathews 1971; Skvarla & Dowling 2017).

The relative similarity of rank-abundance plots at different altitudes, particularly for pitfall-trap samples, may be that many dominant species are adapted to a wide range of altitudes (e.g., Nunes et al. 2016) and/or associated temperature or moisture conditions (Gilchrist, 1995; Saavedra et al., 2013). Climatic factors such as temperature, rainfall, relative humidity, wind velocity and the amount of light may not have changed uniformly with increasing altitude in our study sites, but the possible altitudinal effect might have been compensated by, for example, habitat type or topography (Adam & Enning 1996; Adam et al. 2011). These factors might decrease variation in micro-climate (McCain & Grytnes 2001), perhaps through the sheltering effect of forest trees (Barnes et al. 1997).

According to NMDS and MRT, light traps again distinguished 1,800 m from lower altitudes, whereas Malaise and pitfall traps reflected primarily differences between the two mountains and only secondarily altitudinal variation. Moreover, and only at Fraser's Hill, Malaise traps distinguished 1,800 m from the lower altitudes, and pitfall traps distinguished 500, 1,000 and 1,500-1,800 m. These findings suggest that the overall beetle community varied remarkably according to altitude, but the magnitude of this response depended on geographic location (the two mountains) and sampling method. Also, altitude might partly be a proxy of other environmental variables, such that were not assessed in this study. For instance, the lower (500 and 1,000 m) and higher (1,500 and 1,800 m) altitudes represented different forest types, the former being mixed deciduous and the latter being mixed conifer-heath-myrtle forests.

Pitfall traps produced eight indicator species that were common at all altitudes, whereas the other sampling methods did not produce such altitude generalists. This finding is in line with our rank-abundance plots (Fig. 3). Moreover, all sampling methods produced at least some indicators of low (500–1,000 m), intermediate (1,000–1,500 m) and/or high altitudes (1,500–1,800 m),

suggesting potential for each method to reflect altitude and thus potentially global changes in climate or land use. Important from climate perspective, we found nine significant indicators of 1,800 m (four using light, four using Malaise and one using pitfall traps). Species adapted to these highest mountain tops may face difficulty in survival with predicted climate warming (e.g., Lenoir et al. 2008; Pickering et al. 2008; Dirnböck et al. 2011) unless they can adapt or disperse elsewhere. For most Malaysian beetle species, the level of knowledge on such traits is low. The precautionary principle nevertheless calls for mitigation of climate change, and avoidance of intensive land use at high altitudes (Gallou et al. 2017).

The studied three sampling methods probably differ in capturing effici you can move all this paragraph clo [illegible] captured species pool (present results). These all make the methods difficult to compare. The differences, reported here, nevertheless suggest that the use of more than one location and sampling method is desirable in environmental assessments (Lamarre et al. 2012; Devigne & De Biseau 2014; Abdullah & Musthafa 2019). Here, pitfall traps tended to produce samples distinctive from light and Malaise traps that were more similar in many measures. Hence, a combination of pitfall and light traps might produce richer samples than other trap combinations in Malaysian mountain environments.

Taxonomic constraints may have affected our results to some extent, as most taxa had been identified to family or genus levels only. Thus, two individuals of a given taxon being captured using two methods or at different altitudes might in reality be two different species. However, this possibility rather masks than exaggerates the true community responses. We are therefore confident that the sampling methods and altitudes truly differed in beetle communities, but the differences would have been more pronounced had we been able to identify everything to species.

Land use in the two studied mountains was rather different (see Material and methods). Accordingly, it was not surprising to find out that the mountains hosted structurally different beetle assemblages (Figs. 4-5).

Beetle community assemblage across elevations at both mountains differ significantly, which is reported by other authors from different parts of the world too (Escobar et al. 2005; Wardhaugh et al. 2018; Hiramatsu & Usio 2018; Şenyüz et al. 2019). Moreover, Gallou et al. (2017) showed for butterflies that both altitude and habitat features shape the community structure, human impact having an additional, often negative effect. Ant assemblage differences were also reported by few more authors (Assis et al. 2018; Flores et al. 2018). We did not address this aspect here, however, as we only had one near-pristine (Fraser's Hill) and one partly managed mountain (Genting Highland) to study; also, ecological information for most of the studied taxa is insufficient for detailed assessments.

This research found 83 indicator species for different combinations of Method and Altitude. Moreover, light and Malaise trap samples shared about half of the abundant species, whereas pitfall trap samples were distinctive in this respect. Our results are applicable to tropical species conservation, as they provide evidence for many species to be adapted to particular altitudes and, more importantly, differences in beetle samples between collecting methods, which in turn calls caution in analysis interpretations. We recommend use multiple collecting methods in environmental impact assessments on biodiversity whenever possible. Also, land use at mountain tops should strictly follow the precautionary principle as species specialized to these conditions may be at high risk for local extinctions with changing climate.

Acknowledgements

We also thank Mohd Shukri Mohd Sabri and Davindram A/L Rajendram for the instrumental assistance in beetle sampling. We also acknowledge the Universiti Kebangsaan Malaysia and Forestry Department of Malaysia for supporting this study in many ways.

References

Abdullah F, Musthafa MM. Beetle abundance, diversity and species richness of Pulau Pangkor, Perak, Malaysia. Malaysia Forester 2019; 82: 247-270.

Adam JH, Enning FL. Altitudinal zonation of tropical rain forest at Danum Valley Research Centre, Sabah, Malaysia. Malaysian J Sci 1996; 17A: 25-35.

Adam JH, Ming LK, Juhari MAA, Jaluluddin MA, Idris WMR, Othman AR, Tarmiddzi SNA. Cluster Analysis of Submontane Forest along Western Slope of Frasers' Hill Research Centre in Raub District, Malaysia. Bangladesh J Bot 2011; 40: 121-132.

Anzaldo SS. Review of the genera of Conoderinae (Coleoptera, Curculionidae) from North America, Central America, and the Caribbean. ZooKeys 2017; 683: 51-138. https://doi.org/10.3897/zookeys.683.12080.

Ashton LA, Nakamura A, Basset Y, Burwell CJ, Cao M, Eastwood R, et al. Vertical stratification of moths across elevation and latitude. J. Biogeogr 2016; 43: 59-69. https://doi.org/10.1111/jbi.12616

Ashton LA, Odell EH, Burwell CJ, Maunsell SC, Nakamura A, McDonald WJF, Kitching RL. Altitudinal patterns of moth diversity in tropical and subtropical Australian rainforests. *Austral Ecol* 2015; 41: 197-208. https://doi.org/10.1111/aec.12309.

Assing V. Four new species and additional records of Palaearctic *Sunius*, with two new synonymies (Coleoptera: Staphylinidae: Paederinae). *Beiträge zur èntomologie* 2008; 58: 455-470. https://doi.org/10.21248/contrib.entomol.58.2.455-470.

Baniya CB, Solhøy T, Gauslaa Y, Palmer MW. The elevation gradient of lichen species richness in Nepal. The Lichenologist 2010; 42: 83-96. https://doi.org/10.1017/S0024282909008627

Barnes BV, Zak DR, Denton SR, Spurr SH. Forest ecology. 4th ed. John Wiley & Sons. 1999.

Barzani GM, Sahibin AR, Adam JH, Zulfahmi AR. The physical environment. Bukit Fraser crown of the Titiwangsa Range. In: Latiff, A., Maimon, A., Norhayati, A. & Adam, J.H., editors. Bukit Fraser Research Centre, Universiti Kebangsaan Malaysia & Fraser's Hill Development Corporation, Fraser's Hill Development Corporation, Raub, Malaysia; 2009. pp 9-16.

Bouzan AM, Flinte V, Macedo MV, Monteiro RF. Elevation and temporal distributions of Chrysomelidae in southeast Brazil with emphasis on the Galerucinae. ZooKeys 2015; 547: 103-117. https://doi.org/10.3897/zookeys.547.9723

Brehm G, Colwell RK. Kluge J. () The role of environment and mid-domain effect on moth species richness along a tropical elevational gradient. Glob Ecol Biogeogr 2007; 16: 205-219. https://doi.org/10.1111/j.1466-8238.2006.00281.x

Bates D, Maechler M, Bolker B, Walker S. Fitting linear mixed effects models using lme4. J Stat Softw 2015; 67: 1-48. http://dx.doi.org/10.18637/jss.v067.i01

Borg I, Groenen P. Modern multidimensional scaling: theory and applications. 2nd ed. Springer-Verlag, New York. 2005

Breiman L, Friedman JH, Olshen RA, Stone CG. Classification and regression trees. CRC Press, Boca Raton, Florida. 358 pp. 1998.

Chao A, Jost, L. Coverage-based rarefaction and extrapolation: standardizing samples by completeness rather than size. Ecol 2012; 93(12): 2533–2547.

Chen IC, Shiu HJ, Benedick S, Holloway JD, Chey VK, Barlow HS, Hill JK, Thomas CD. Elevation increases in moth assemblages over 42 years on a tropical mountain. PNAS 2009; 106: 1479-1483. https://doi.org/10.1073/pnas.0809320106

Cheong C. Fraser's Hill - A Lush Highland Hideaway. WWF Malaysia, Petaling Jaya. 2013.

Cox CB, Moore PD, Ladle R. Biogeography: an ecological and evolutionary approach. Wiley Blackwell. 2016.

De Cáceres M, Legendre P, Moretti M. Improving indicator species analysis by combining groups of sites. Oikos 2010; 119: 1674-1684. DOI: 10.1111/j.1600-0706.2010.18334.x

De Cáceres M, Jansen F. Package Indicspecies. 2016. URL https://CRAN.R-project.org/package=indicspecies.

De'ath G. Multivariate regression trees: a new technique for modeling species–environment relationships. Ecol 2002; 83(4): 1105-1117. https://doi.org/10.2307/3071917

De'ath G, Therneau TM, Atkinson B, Ripley B, Oksanen J. mvpart: Multivariate partitioning. 2014. URL https://mran.microsoft.com/snapshot/2014-12-11/web/packages/mvpart/index.html

Devigne C, De Biseau J-C. Urban ecology: comparison of the effectiveness of five traps commonly used to study the biodiversity of flying insects. Biodivers J 2014; 5: 165-174.

Doll HM, Butod E, Harrison RD, Fletcher C, Kassim AR, Ibrahim S, Potts MD. Environmental and geographic factors driving dung beetle (Coleoptera: Scarabaeidae: Scarabaeinae) diversity in the dipterocarp forests of Peninsular Malaysia. Raffles Bull Zool 2014; 62: 549-560.

Dufrêne M, Legendre P. Species assemblages and indicator species: the need for a flexible asymmetrical approach. Ecol Monogr 1997; 67(3): 345-366. https://doi.org/10.1890/0012-9615(1997)067[0345:SAAIST]2.0.CO;2

Dirnböck T, Essl F, Rabitsch W. Disproportional risk for habitat loss of high-altitude endemic species under climate change. Glob Chang Biol 2011; 17: 990-996. https://doi.org/10.1111/j.1365-2486.2010.02266.x

Elsen PR, Tingley MW. Global mountain topography and the fate of montane species under climate change. Nat Clim Chang 2015; 5: 772-776. https://doi.org/10.1038/nclimate2656

Escobar F, Lobo JM, Halffter G. Altitudinal variation of dung beetle assemblages in the Colombian Andes. Glob Ecol Biogeogr 2005; 14: 337-347. https://doi.org/10.1111/j.1466-822X.2005.00161.x

Filippini V, Micó E, Galante E. Checklist and identification key of Anomalini (Coleoptera, Scarabaeidae, Rutelinae) of Costa Rica. ZooKeys 2016; 621: 63-136. https://doi.org/10.3897/zookeys.621.7565

Fox J, Weisberg S. An R companion to applied regression. 3rd edition. Sage Publications Inc., California. 2019.

Gallou A, Baillet Y, Ficetola GF, Despres L. Elevational gradient and human effects on butterfly species richness in the French Alps. Ecol Evol 2017; 7(11): 1-10. https://doi.org/10.1002/ece3.2803

Gilchrist GW. () Specialists and generalists in changing environments. I. Fitness landscapes of thermal sensitivity. Am Nat 1995; 146: 252-270.

Hlaváč P, Newton AF, Maruyama M. World catalogue of the species of the tribe Lomechusini (Staphylinidae: Aleocharinae). Zootaxa 2011; 3075: 1-151.

Hooper DU, Chapin FS III, Ewel JJ, Hector A, Inchausti P, et al. Effects of biodiversity on ecosystem functioning: a consensus of current knowledge. Ecol Monogr 2005; 75: 3-35. https://doi.org/10.1890/04-0922.

Kessler M. Elevational gradients in species richness and endemism of selected plant groups in the central Bolivian Andes. Plant Ecol 2000; 149: 181-193.

Kiew R. The seed plant flora of Fraser's Hill, Peninsular Malaysia: With special reference to its conservation status. Research pamphlet No. 21. Forest Research Institute of Malaysia (FRIM), Kuala Lumpur, Malaysia. 1998.

Kohler T, Wehrli A, Jurek M. Mountains and climate change: A global concern. Sustainable Mountain Development Series. Bern, Switzerland, Centre for Development and Environment (CDE), Swiss Agency for Development and Cooperation (SDC) and Geographica Bernensia. 2014.

Koivula MJ, Chmaberlain DE, et al. Breeding bird species diversity across gradients of land use from forest to agriculture in Europe. Ecogeg 2018; 41: 1331-1344. https://doi.org/10.1111/ecog.03295

Körner C. Alpine plant life. Functional plant ecology of high mountain ecosystems. 2nd edition. Springer, Berlin. 2003.

Kumar A, Longino JT, Colwell RK, O'Donnell S. Elevational patterns of diversity and abundance of eusocial paper wasps (Vespidae) in Costa Rica. Biotropica 2009; 41, 338-346. https://doi.org/10.1111/j.1744-7429.2008.00483.x

Laliberte E, Wells JA, DeClerck,F… Mayfield, MM. Land-use intensification reduces functional redundancy and response diversity in plant communities. Ecol Lett 2009; 13: 76-86. https://doi.org/10.1111/j.1461-0248.2009.01403.x

Lamarre GPA, Molto Q, Fine PVA, Baraloto C. A comparison of two common flight interception traps to survey tropical arthropods. ZooKeys 2012; 216: 43-55. https://doi.org/10.3897/zookeys.216.3332

Lenoir J, Gegout JC, Marquet PA, de Ruffray P, Brisse H. A significant upward shift in plant species optimum elevation during the 20th century. Science 2008; 320: 1768-1771. DOI: 10.1126/science.1156831.

Makranczy G. Review of the *Anotylus* cimicoides species group (Coleoptera: Staphylinidae: Oxytelinae). Acta Zoolog Academiae Scientiarum Hung 2017; 63: 143-262. DOI: 10.17109/AZH.63.2.143.2017

Mathews RW., Mathews JR. The Malaise Trap: Its Utility and Potential for Sampling Insect Populations. *The Great Lakes Entomologist* 1971; 4(4): Article 4.

McCain CM, Grytnes JA. Elevation gradients in species richness. In Encyclopedia of Life Sciences (ELS). John Wiley & Sons Ltd., Chichester. 2000.

McGeachie WJ.. The effects of moonlight illuminance, temperature and wind speed on light-trap catches of moths. Bull Entomol Res 1989; 79: 185-192.

Mittermeier RA, Turner WR, Larsen FW, Brooks TM, Gascon C. Global Biodiversity Conservation: The Critical Role of Hotspots. In: Zachos, F.E. & Habel, J.C. editors, Biodiversity Hotspots. Springer-Verlag, Berlin-Heidelberg. 2011. Pp. 3-22.

Mohamedsaid MS. Catalogue of the Malaysian Chrysomelidae (Insecta: Coleoptera). Pensoft Publishers, Bulgaria. 2004.

Musthafa MM, Abdullah F. Coleoptera of Genting Highland, Malaysia: Species richness and diversity along the elevations. Arx de Misc Zoology 2019; 17: 45-66. DOI: 10.32800/amz.2019.17.0045.

Musthafa MM, Abdullah F, Sánchez‐Reyes UJ. Comparative study of spatial patterns and ecological niches of beetles in two Malaysian mountains elevation gradients. J Insect Conserv 2018; 22(5-6): 757-769. https://doi.org/10.1007/s10841-018-0099-z

Myers N, Mittermeier RA, Mittermeier CG, da Fonseca GAB, Kent J. Biodiversity hotspots for conservation priorities. Nature 2000; 403: 853-858.

Naomi S. Taxonomic revision of the genus *Stenus* Latreille, 1797 (Coleoptera, Staphylinidae, Steninae) of Japan: Species group of S. indubius Sharp. Jpn J Syst Entomol 2006; 12: 39-120.

Newbold T, et al. Global effects of land use on local terrestrial biodiversity. Nature 2015; 520: 45-50. https://doi.org/10.1038/nature14324

Nowinszky L, Puskás J. Light-Trap Catch of Insects in Connection with Environmental Factors. In: Vonnie D.C. Shields, editors, *Biological Control of Pest and Vector Insects*, InTech Publishers. 2017. pp. 97-117.

Nunes CA, Braga RF, Figueira JEC, Neves FS, Fernandes GW. Dung beetles along a tropical altitudinal gradient: environmental filtering on taxonomic and functional diversity. PLoS ONE 2016; 11: e0157442. https://doi.org/10.1371/journal.pone.0157442

Oksanen J, Blanchet FG, Friendly M, Kindt R, Legendre P, McGlinn D, et al. Package vegan. Community Ecology Package. 2018. URL https://cran.r-project.org, https://github.com/vegandevs/vegan

Peh KS-H, Soh MCK, Sodhi NS, Laurance WF, Ong DF, Clements R. Up in the clouds: Is sustainable use of tropical montane cloud forests possible in Malaysia? Bioscience 2011; 61: 27–38. https://doi.org/10.1525/bio.2011.61.1.8

Perumal B, Lo FSJ. Recorded mountain trees of Peninsular Malaysia - a checklist. World Wildlife Fund Malaysia, Kuala Lumpur, Malaysia. 1998.

Pickering C, Hill W, Green K. Vascular plant diversity and climate change in the alpine zone of the Snowy Mountains, Australia. Biodivers Conserv 2008; 17: 1627-1644. https://doi.org/10.1007/s10531-008-9371-y

Piggott AG. The ferns of Gunung Ulu Kali. Garden Bulletin of Singapore 1977; 30: 31-43.

Prathapan KD, Viraktamath CA. A new species of *Longitarsus* Latreille, 1829 (Coleoptera, Chrysomelidae, Galerucinae) pupating inside stem aerenchyma of the hydrophyte host from the Oriental Region. ZooKeys 2011; 87: 1-10. doi: 10.3897/zookeys.87.1294

R Core Team. R: A language and environment for statistical computing. R Foundation for Statistical Computing, Vienna, Austria. 2019. URL https://www.R-project.org/

Rahbek C. The elevational gradient of species richness: a uniform pattern? Ecography 1995; 18: 200-205. DOI: 10.1111/j.1600-0587.1995.tb00341.x

Rangwala I, Miller JR. Climate change in mountains: a review of elevation-dependent warming and its possible causes. Clim Change 2012; 114: 527-547. https://doi.org/10.1007/s10584-012-0419-3

Ryvkin AB. Contributions to the knowledge of *Stenus* (Nestus) species of the *Crassus* group (Insecta: Coleoptera: Staphylinidae: Steninae). Four new species from the Russian Far East with taxonomic notes. Balt J Coleopterol 2011; 11: 57-72.

Saavedra S, Rohr RP, Dakos V, Bascompte J. Estimating the tolerance of species to the effects of global environmental change. Nat Commun 2013; 4: 2350. https://doi.org/10.1038/ncomms3350

Saito M. A New Species of the genus *Tomoderus* (Coleoptera, Anthicidae) from the Ryukyu Islands, Southwest Japan. *Elytra* 2003; 31: 321-323.

Sasakawa K, Kim J-K, Kim J-K, Kubota K. Morphological phylogeny and biogeography of the *Pterostichus raptor* species group (Coleoptera: Carabidae) of ground beetles, endemic to the Korean Peninsula and adjacent islands. J Asia Pac Entomol 2017; 20: 7-12. https://doi.org/10.1016/j.aspen.2016.11.001

Schawaller W. Revision of the Oriental species of the genus Bradymerus Perroud, with descriptions of 29 new species (Coleoptera: Tenebrionidae). Stuttgarter Beiträge zur Naturkunde 2006; 4: 1-64.

Schawaller W. Two new species and new records of the genus *Spinolyprops* Pic, 1917 from the Oriental Region (Coleoptera, Tenebrionidae, Lupropini). ZooKeys 2012; 243: 83-94. DOI: 10.3897/zookeys.243.3879

Schimmel R, Tarnawski D. The species of the genus *Mulsanteus* Gozis, 1875 of Southeast Asia (Insecta: Coleoptera: Elateridae). Genus 2007; 14, 89-172.

Sheikh AH, Thomas M, Bhandari R, Bunkar K. Light trap and insect sampling: an overview. Int J Curr Res 2016; 8(11): 40868-40873.

Shi H, Zhou H, Liang H. Taxonomic synopsis of the subtribe *Physoderina* (Coleoptera, Carabidae, Lebiini), with species revisions of eight genera. Zookeys 2013; 284: 1-129. https://doi.org/10.3897/zookeys.284.3983

Skvarla MJ, Dowling APG. A comparison of trapping techniques (Coleoptera: Carabidae, Buprestidae, Cerambycidae, and Curculionoidea excluding Scolytinae). J Insect Sci 2017; 17(1): 7-20. https://doi.org/10.1093/jisesa/iew098

Staunton KM, Nakamura A, Burwell CJ, Robson SKA, Williams SE. Elevational distribution of flightless ground beetles in the tropical rainforests of North-Eastern Australia. PLoS ONE 2016; 11: e0155826. https://doi.org/10.1371/journal.pone.0155826

Stone BC. The summit flora of Gunung Ulu Kali (Pahang, Malaysia). Federation of Museum Journal 1981; 26(1): 157-162.

Wardhaugh CW, Stone MJ, Stork NE. Seasonal variation in a diverse beetle assemblage along two elevational gradients in the Australian Wet Tropics. Sci Rep 2018; 8: 8559. https://doi.org/10.1038/s41598-018-26216-8

Ya'cob Z, Takaoka H, Pramual P, Low VL, Sofian-Azirun M. Breeding habitat preference of preimaginal black flies (Diptera: Simuliidae) in Peninsular Malaysia. Acta Tropica 2016; 153: 57-63. doi: 10.1016/j.actatropica.2015.10.007.

Zuur AF, Ieno EN, Walker NJ, Saveliev AA, Smith GM. Mixed effects models and extensions in ecology with R. Springer Science. 2009.

Table 1 Sampling effort at Fraser's Hill (FH) and Genting Highland (GH) mountains. In both mountains, traps were set at four Altitudes: 500, 1,000, 1,500 and 1,800 m a.s.l., in ten stands in both mountains; stands are indicated with letters A-J (compare Fig. 1). The three right-hand columns show the number of traps (or groups of five pitfall traps) in each stand.

Mountain	Altitude	Stand ID	Light	Malaise	Pitfall
FH	500	A	-	-	1
FH	500	B	-	-	4
FH	500	C	1	-	-
FH	1,000	D	1	2	5
FH	1,000	E	1	-	-
FH	1,500	F	1	-	2
FH	1,500	G	-	1	1
FH	1,500	H	1	1	2
FH	1,800	I	1	2	1
FH	1,800	J	1	-	4
GH	500	A	-	1	-
GH	500	B	-	1	-
GH	500	C	2	-	5
GH	1,000	D	-	-	2
GH	1,000	E	2	2	3
GH	1,500	F	-	2	4
GH	1,500	G	-	-	1
GH	1,500	H	2	-	-
GH	1,800	I	2	1	4
GH	1,800	J	-	1	1

Table 2 . GLMM output for Altitude, using Mountain as a random factor, for the number of species and rarefaction standardized number of species for 5, 10 or 20 individuals. Results are shown for light, Malaise and pitfall trap data. Numbers in “%var” columns are percentages explained by a given variable; letters in #Effect” columns show whether a given Altitude differed significantly (p < 0.05) and positively (pos) or negatively (neg) from the lowest Altitude (500 m a.s.l.) (in parentheses if marginally significant, i.e., p < 0.1) or whether this comparison was non-significant (ns; p >0.1). For details, see text; for full output, see Appendix Table 2.

	**LIGHT TRAPS**			**MALAISE TRAPS**			**PITFALL TRAPS**	
**Variable/Category**	**%var**	**Effect**		**%var**	**Effect**		**%var**	**Effect**
Number of species								
Mountain	7			4			53	
Altitude	41			78			11	
* 1,000 m a.s.l.		ns			neg			(pos)
* 1,500 m a.s.l.		ns			neg			ns
* 1,800 m a.s.l.		neg			neg			ns
Residuals	52			19			35	
Rarefied richness to 5 individuals								
Mountain	7			6			17	
Altitude	33			70			16	
* 1,000 m a.s.l.		ns			ns			pos
* 1,500 m a.s.l.		ns			ns			ns
* 1,800 m a.s.l.		(neg)			neg			ns
Residuals	60			24			67	
Rarefied richness to 10 individuals								
Mountain	15			7			26	
Altitude	45			72			11	
* 1,000 m a.s.l.		ns			ns			pos
* 1,500 m a.s.l.		ns			ns			ns
* 1,800 m a.s.l.		neg			neg			ns
Residuals	40			21			63	
Rarefied richness to 20 individuals								
Mountain	4			7			28	
Altitude	61			72			12	
* 1,000 m a.s.l.		ns			(neg)			pos
* 1,500 m a.s.l.		ns			neg			ns
* 1,800 m a.s.l.		neg			neg			ns
Residuals	35			21			59	

Table 3 PERMANOVA pairwise comparisons of beetle composition between elevation sites in Genting Highland (Upper diagonal = F values. Lower diagonal = p values)

	500 m asl	1000 m asl	1500 m asl	1800 m asl
Fraser's Hill				
500 m asl	-	15.13	28.58	15.78
1000 m asl	0.0001	-	19.86	15.06
1500 m asl	0.0001	0.0001	-	7.563
1800 m asl	0.0001	0.0001	0.0001	-
Genting Highland				
500 m asl	-	6.969	17.35	20.29
1000 m asl	0.0001	-	9.099	10.75
1500 m asl	0.0001	0.0001	-	8.028
1800 m asl	0.0001	0.0001	0.0001	-

Table 4 Permutational multivariate ANOVA for NMDS to assess the distinctiveness of different Altitudes (Alt 500, 1,000, 1,500 or 1,800 m a.s.l., or their logical combinations) as reflected by using three sampling methods (compare Fig. 2).

	**LIGHT TRAPS**							**MALAISE**							**PITFALL**					
**Variable**	**df**	**SS**	**MS**	**F**	**R^2**	**p**		**df**	**SS**	**MS**	**F**	**R^2**	**p**		**df**	**SS**	**MS**	**F**	**R^2**	**p**
Alt 500	1	0.47	0.47	1.28	0.09	0.0659		1	0.40	0.40	1.17	0.09	0.2717		1	0.48	0.48	1.58	0.04	0.0300
Residuals	13	4.75	0.37	0.91				12	4.12	0.34	0.91				38	11.55	0.30	0.96		
Alt 1000	1	0.47	0.47	1.29	0.09	0.1548		1	0.42	0.42	1.23	0.09	0.1528		1	0.97	0.97	3.34	0.08	0.0010
Residuals	13	4.75	0.37	0.91				12	4.10	0.34	0.91				38	11.06	0.29	0.92		
Alt 1500	1	0.53	0.53	1.47	0.10	0.0939		1	0.42	0.42	1.24	0.09	0.1299		1	0.46	0.46	1.51	0.04	0.0390
Residuals	13	4.69	0.36	0.90				12	4.10	0.34	0.91				38	11.57	0.30	0.96		
Total	14	5.22	1.00					13	4.52	1.00					39	12.03	1.00			
Alt 1800	1	1.36	1.36	4.56	0.26	0.0020		1	0.70	0.70	2.21	0.16	0.0030		1	0.76	0.76	2.56	0.06	0.0020
Residuals	13	3.87	0.30	0.74				12	3.82	0.32	0.84				38	11.27	0.30	0.94		
Alt 500-1000	1	0.67	0.67	1.93	0.13	0.0290		1	0.47	0.47	1.40	0.10	0.0699		1	0.97	0.97	3.33	0.08	0.0010
Residuals	13	4.55	0.35	0.87				12	4.05	0.34	0.90				38	11.06	0.29	0.92		
Alt 1000-1500	1	0.98	0.98	3.01	0.19	0.0010		1	0.68	0.68	2.13	0.15	0.0420		1	0.55	0.55	1.82	0.05	0.0070
Residuals	13	4.24	0.33	0.81				12	3.84	0.32	0.85				38	11.48	0.30	0.95		
Alt 1500-1800	1	0.67	0.67	1.93	0.13	0.0380		1	0.47	0.47	1.40	0.10	0.0619		1	0.97	0.97	3.33	0.08	0.0010
Residuals	13	4.55	0.35	0.87				12	4.05	0.34	0.90				38	11.06	0.29	0.92		
Alt 500-1500	1	1.36	1.36	4.56	0.26	0.0020		1	0.70	0.70	2.21	0.16	0.0050		1	0.76	0.76	2.56	0.06	0.0020
Residuals	13	3.87	0.30	0.74				12	3.82	0.32	0.84				38	11.27	0.30	0.94		
Alt 1000-1800	1	0.47	0.47	1.28	0.09	0.0699		1	0.40	0.40	1.17	0.09	0.2408		1	0.48	0.48	1.58	0.04	0.0210
Residuals	13	4.75	0.37	0.91				12	4.12	0.34	0.91				38	11.55	0.30	0.96		

Table 5 Significant ($p < 0.05$) indicators with $n > 4$ in the beetle data. LT = indicator of light traps; MA = indicator of Malaise traps; PT = indicator of pitfall traps; different Altitudes or ranges are shown with numbers (500, 1,000, 1,500, 1,800 m a. s. l.). Column IndVal shows indicator value for each taxon.

**Category**	**Species**	**IndVal**		**Category**	**Species**	**IndVal**
**Single-method indicators**				**Single-method indicators continued**		
LT 500	*Anomala* sp2	0.81		PT	*Harpalus* sp1	0.84
LT 500	*Anomala* sp4	0.76		PT	*Harpalus* sp2	0.91
LT 500	*Anomala* sp6	0.57		PT	*Hiletus* sp1	0.44
LT 500	*Apogonia* sp3	0.79		PT	*Inopeplus* sp1	0.63
LT 500	*Apogonia* sp5	0.79		PT	*Lebia* sp1	0.57
LT 500	*Cicindela* sp2	0.79		PT	*Omonadus* sp1	0.52
LT 500	Mordellidae A	0.80		PT	*Pentagonica* sp1	0.54
LT 500	Scarabeidae M	0.80		PT	Staphylinidae M	0.63
LT 500	Scarabeidae P	0.58		PT 500	*Pterostichus* sp2	0.87
LT 500-1000	*Epepeotes lateralis*	0.52		PT 500-1500	*Spinolyprops* A	0.53
LT 1000	*Apogonia* sp1	0.90		PT 500-1500	Staphylinidae C	0.61
LT 1000	*Cicindela* sp1	0.69		PT 1000	*Actiastes* sp1	0.71
LT 1000-1500	*Anomala* sp1	0.94		PT 1000	*Anotylus* sp2	0.86
LT 1000-1500	*Apogonia* sp2	0.96		PT 1000	*Bledius* sp1	0.78
LT 1500	Lampyridae E	0.70		PT 1000	*Lispinus* sp1	0.84
LT 1500	*Luciola* sp1	0.71		PT 1000	*Orphnebius* sp1	0.84
LT 1500-1800	*Altica* sp1	0.69		PT 1000	*Orphnebius* sp2	0.92
LT 1800	*Cleorina* sp	0.71		PT 1000	Passalidae A	0.44
LT 1800	*Hoplocerambyx spinicornis*	0.87		PT 1000	*Pityogenes* sp1	0.74
LT 1800	*Hydrovatus enigmaticus*	0.97		PT 1000	*Sunius* sp1	0.71
LT 1800	*Illeis* sp2	0.70		PT 1000-1800	*Oxylatus* sp1	0.57
MA 500	Aleocharinae sp1	0.84		PT 1500	*Hiletus* sp2	0.58
MA 500	*Anotylus* sp1	0.94		PT 1500	*Pterostichus* sp1	0.79
MA 500	*Aphthona* sp1	0.98		PT 1500-1800	*Paedarus* sp1	0.69
MA 500	*Brachypeplus* sp1	0.92		PT 1800	*Lebia* sp2	0.57
MA 500	*Brachypeplus* sp3	0.98		PT 1800	*Pterostichus* sp3	0.79
MA 500	*Bradymerus* sp2	0.99				
MA 500	*Epuraea* sp1	0.70		**Multiple-method indicators**		
MA 500	Galerucinae sp1	0.97		MA+LT 500	*Mulsanteus* sp1	0.60
MA 500	*Ischnosoma* sp1	0.95		MA+LT 500	Paederinae sp3	0.68
MA 500	*Lymantor* sp1	0.94		MA+LT 1000	*Sarmydus* sp1	0.71

MA 500	*Lymantor* sp2	0.91		MA+LT 1000-1500	*Strotocera* sp1	0.71
MA 500	*Lymantor* sp3	0.94		MA+LT 1500	Alticinae sp2	0.69
MA 500	*Sinoxylon* sp1	0.99		MA+LT 1500	*Anisandrus* sp1	0.68
MA 500	*Xyleborus* sp1	0.98		MA+LT 1500	*Brachypeplus* sp2	0.65
MA 500-1000	Aleocharinae sp2	0.58		MA+LT 1500	*Colaspoma* sp2	0.71
MA 1000	*Anomala* sp3	0.82		MA+LT 1500	Curculionidae A	0.70
MA 1000-1500	Alticinae sp1	0.70		MA+LT 1500	Meloidae A	0.71
MA 1500	Paederinae sp2	0.66		MA+LT 1500	*Nisotra* sp2	0.71
MA 1500-1800	*Xyleborus* sp2	0.71		MA+LT 1500	*Pityogenes* sp2	0.48
MA 1800	*Anomala* sp5	0.66		MA+PT 500	Aleocharinae sp3	0.62
MA 1800	*Apogonia* sp4	0.69				
MA 1800	*Lymantor* sp4	0.90				
MA 1800	*Xylothrips* sp1	0.68				

Figure captions

Fig. 1. Study location in Malaysia. Left: map of Malaysia, with major mountain regions shown with gray; the two squares show locations of Fraser's Hill (top right) and Genting Highland (bottom right) mountains. Right-hand graphs show locations of ten study stands in each study mountain (denoted with letters A-J); black lines show major highways; Altitudes are indicated with numbers (500, 1,000, 1,500 or 1,800 m a.s.l.). In the bottom right-hand graph, the gray patch shows a forest reserve.

Fig. 2. Average samples + SE of four measures of species richness for three sampling methods according to Altitude. Number of species, and rarefaction standardized richness based on 5, 10 and 20 individuals are shown.

Fig. 3. Dominance (mean + SE) of twenty most abundant species in each trap type at different Altitudes (500, 1,000, 1,500 and 1,800 m a.s.l.). Note that species are mostly different for each trap type, each sorted according to the rank order of the total number of individuals in that trap type. Grey columns show species that were shared between light and Malaise traps, and white columns show the shared species between light and pitfall traps (none were shared between Malaise and pitfall traps).

Fig. 4. NMDS plots for Malaysian beetle data, separate runs for three sampling methods (light, Malaise or pitfall traps) at different Altitudes (500 m = black squares, 1,000 m = red circles, 1,500 m = green up-triangles and 1,800 m = blue down-triangles). For statistical comparisons between Altitudes, see Table 3.

Fig. 5. MRT for sampling-method specific beetle data, using mountain (Fraser's Hill or Genting Highland) and Altitude (500, 1,000, 1,500 or 1,800 m a.s.l.) as explanatory variables. The

column plots show relative abundance of each species captured using a given trap type, sorted according to the rank order of abundance of the total trap-type specific sample. Numbers in parentheses below each end branch show the number of samples falling into that branch.

Figure 1

Click here to access/download;Figure;Fig 1.tif

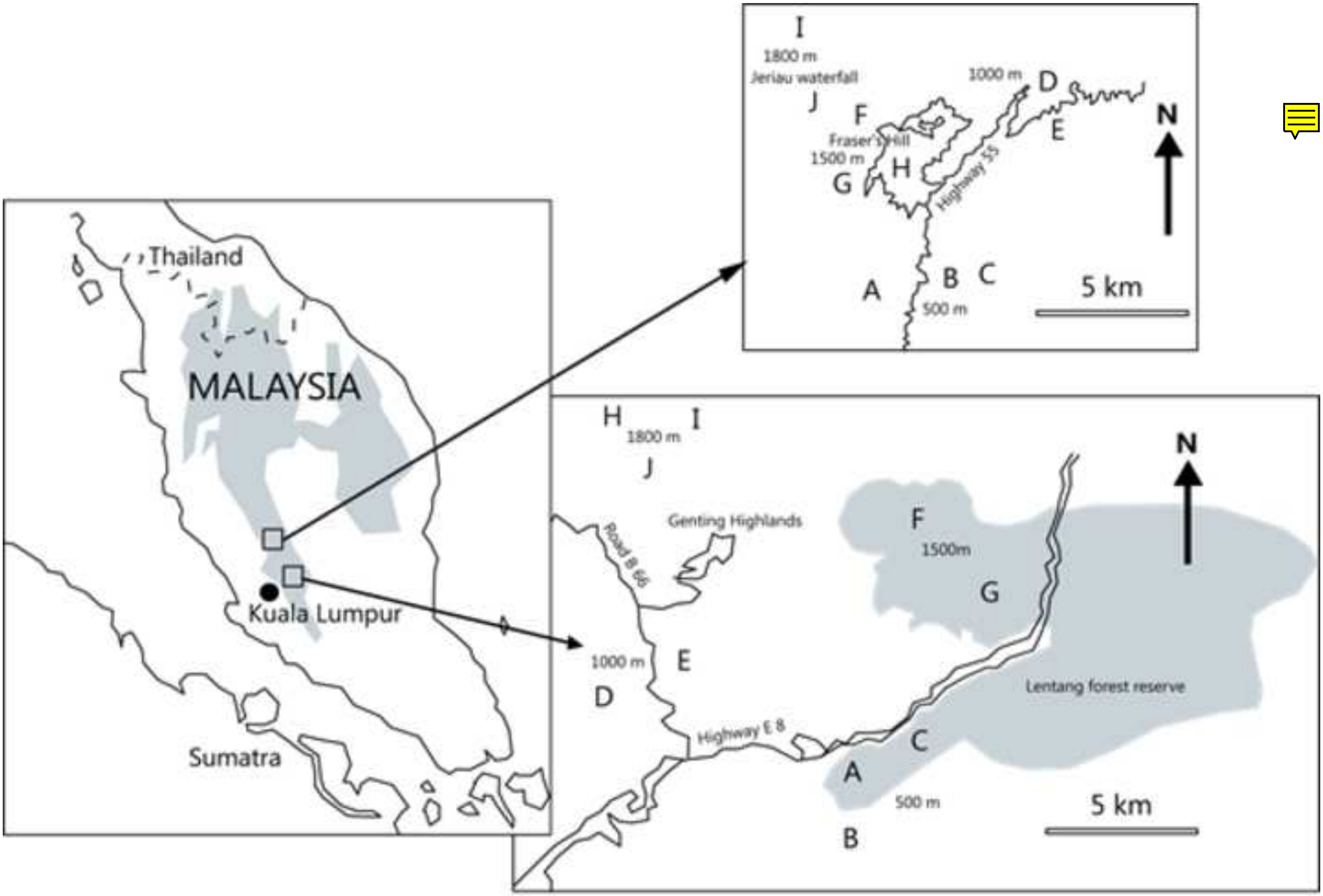

Figure 2

Click here to access/download;Figure;Fig2.tif

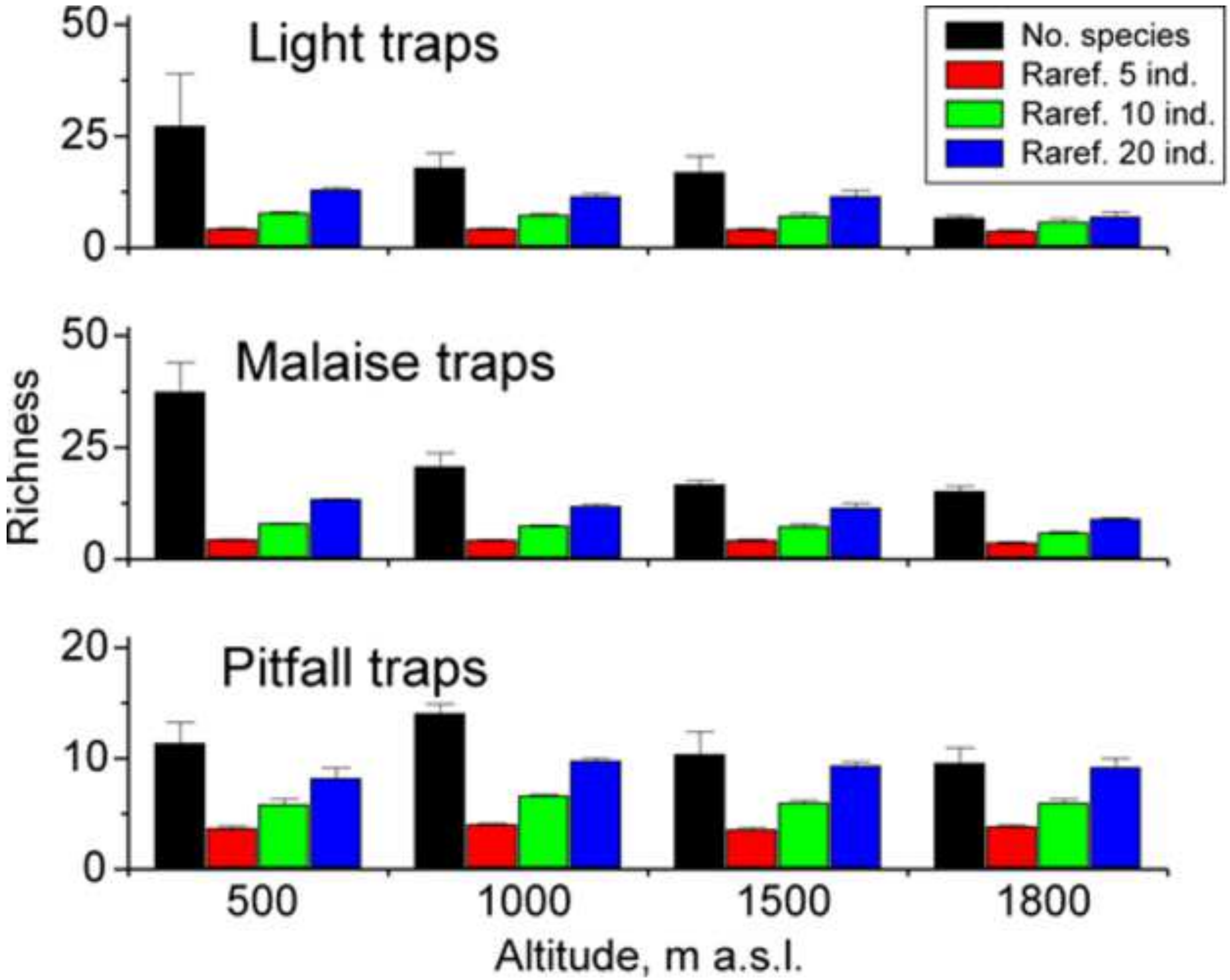

Figure 3

Click here to access/download;Figure;Fig3.tif

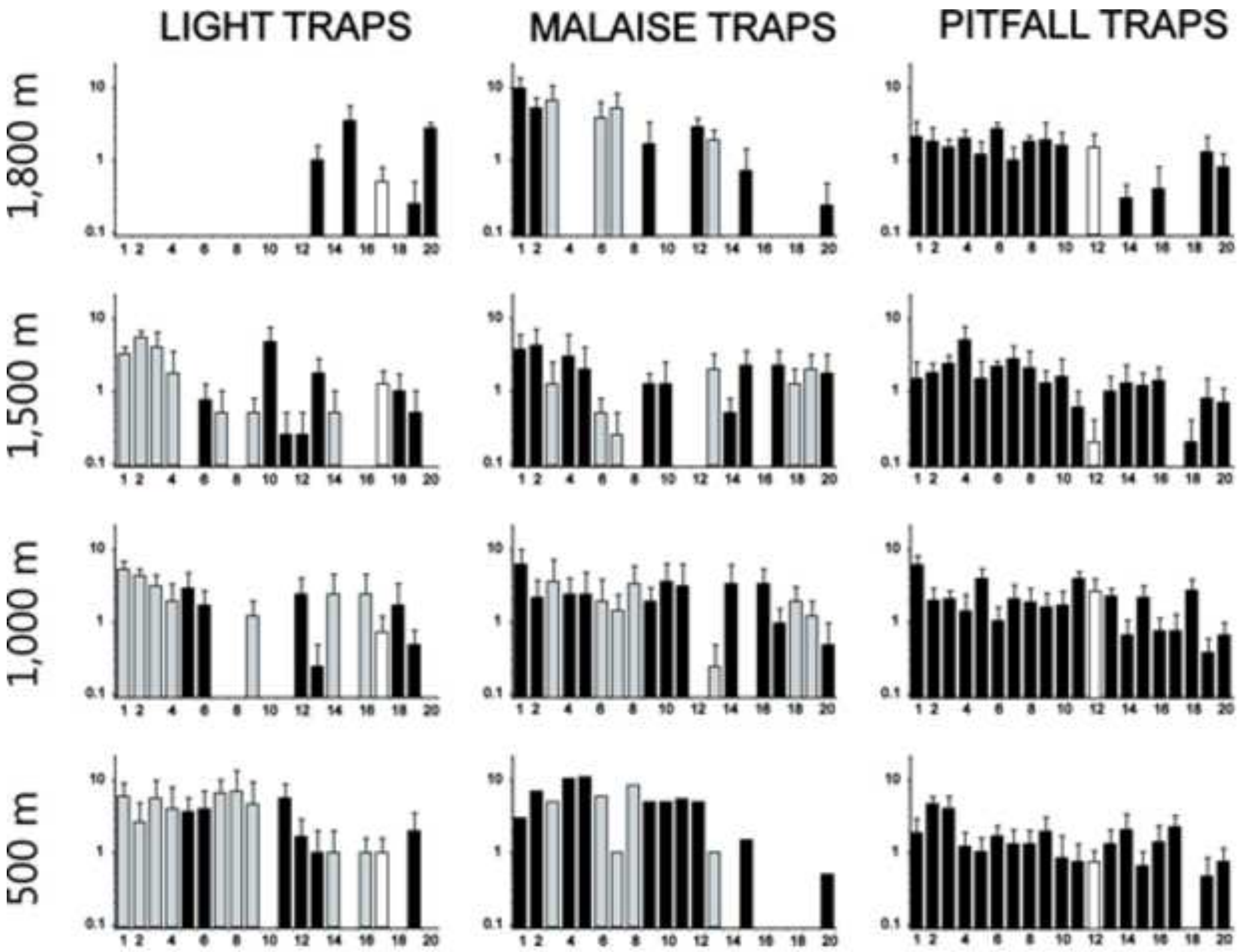

Figure 4

Click here to access/download;Figure;Fig4.tif

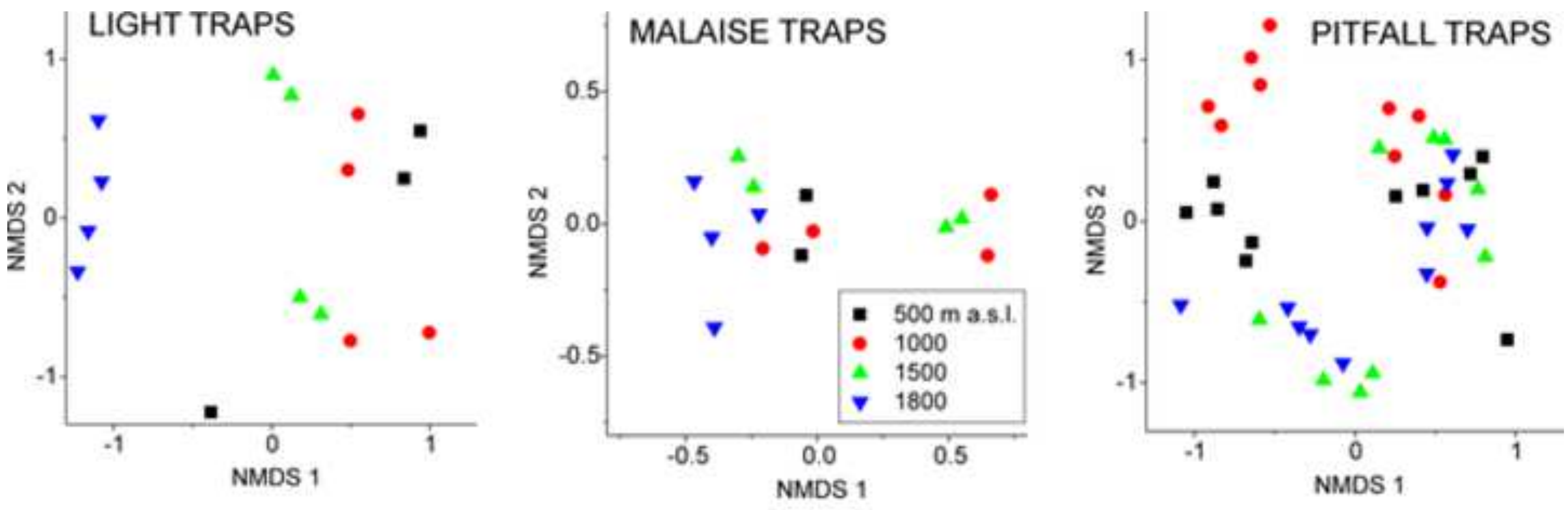

Figure 5

Click here to access/download;Figure;Fig5.tif

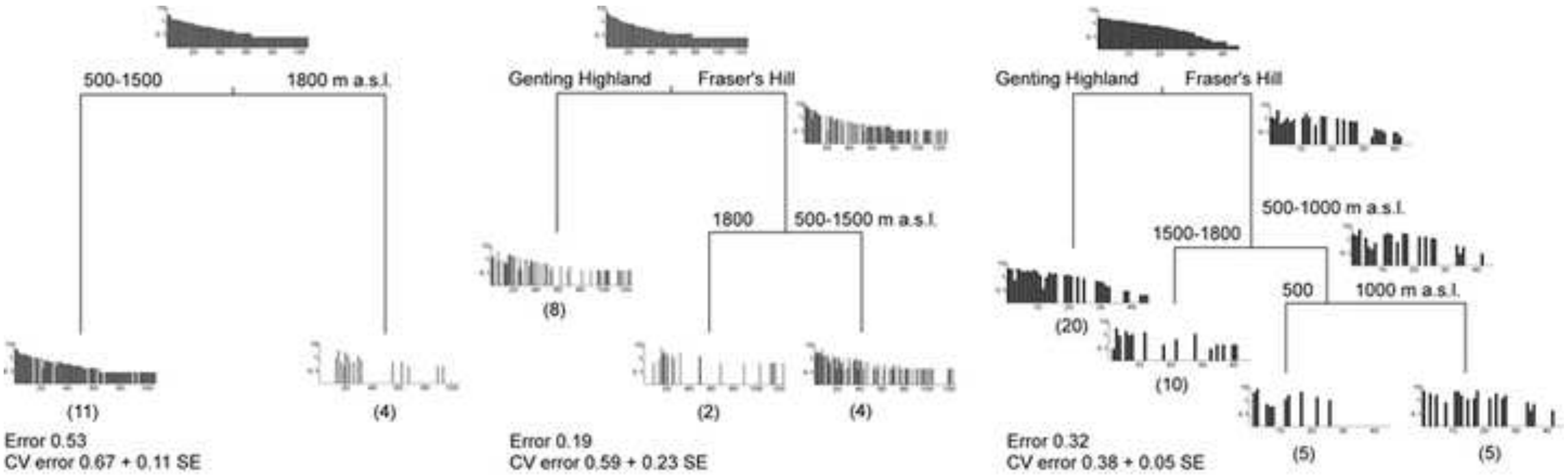

Supporting Information 1

Click here to access/download
Supporting Information
Musthafa et al_supplementary_figure_1.tif

Supporting Information 2

Click here to access/download
Supporting Information
Musthafa et al_supplementary_figure_2.tif
