# Supplementary material for: The functioning of different beetle (Coleoptera) sampling methods across altitudinal gradients in Peninsular Malaysia
Source: PLoS One. 2022 Mar 31;17(3):e0266076. doi: 10.1371/journal.pone.0266076 (PMC8970512; doi:10.1371/journal.pone.0266076)
Supplement: Attachment — Submitted filename: PONE-D-21-03456_R1_reviewed.pdf [file pone.0266076.s009.pdf]

PLOS ONE

The functioning of different beetle (Coleoptera) sampling methods across altitudinal gradients in Peninsular Malaysia

--Manuscript Draft--

**Manuscript Number:**	PONE-D-21-03456R1
**Article Type:**	Research Article
**Full Title:**	The functioning of different beetle (Coleoptera) sampling methods across altitudinal gradients in Peninsular Malaysia
**Short Title:**	Beetle sampling across altitudes in Malaysia
**Corresponding Author:**	Muneeb M. No Middle Name Musthafa, PhD<br>South Eastern University of Sri Lanka<br>Oluvil, Ampara SRI LANKA
**Keywords:**	biodiversity; altitude; forest; light trap; Malaise trap; pitfall trap; tropical
**Abstract:**	Biodiversity research relies largely on knowledge about species responses to environmental gradients, assessed using some commonly applied sampling method. However, the consistency of detected responses using different sampling methods, and thus the generality of findings, has seldom been assessed in tropical ecosystems. Hence, we studied the response consistency and indicator functioning of beetle assemblages in altitudinal gradients from two mountains in Malaysia, using Malaise, light, and pitfall traps. The data were analyzed using generalized linear mixed-effects models (GLMM), non-metric multidimensional scaling (NMDS), multivariate regression trees (MRT), and indicator species analysis (IndVal). We collected 198 morpho-species of beetles representing 32 families, with a total number of 3,052 individual beetles. We found (1) considerable differences in sample richness and composition between two mountains in pitfall but less so in light or Malaise traps; (2) light traps (but not the other trap types) distinguished high from middle or low altitudes in terms of beetle richness and assemblage composition; and (3) lower altitudes hosted about twice as many indicators as middle or high altitudes, and many species were trap-type specific in our data. These results suggest that the detection of community variation in the environment depends on the sampling method, but also on the analytical approach, suggesting the need to use multiple methods in environmental assessments.
**Order of Authors:**	Muneeb M. No Middle Name Musthafa, PhD
	Fauziah Abdullah
	Matti J. Koivula
**Response to Reviewers:**	• TITLE<br>o The title does not reflect the main idea of the research. In my view, the main objective is to test different trap methodologies.<br>o The functioning and environmental gradient do not add up to the work. The study is only with altitudes gradients.<br>Response: this is true. We rewrote the title accordingly.<br><br>• INTRODUCTION<br>o I missed in the introduction a better explanation about the methods. Why do you expect that the traps will have different outcomes? How beetle biology may affect these differences?<br>o The Climate change sentence is lost in the text, and don’t add up to your objective, you can use it in the discussion and conclusion part, but it does not make any difference in the introduction.<br>o need to make it clearer in the introduction that the goal in the introduction is to compare the methods.<br>Response:<br>1. We dedicated one paragraph to method outcomes. Beetle biology indeed impacts this, but as with many tropical regions, including the studied one, their fauna, species and ecologies are extremely poorly understood. Hence we can mostly only speculate about species biology here.

Powered by Editorial Manager® and ProduXion Manager® from Aries Systems Corporation

2. We see the reviewer's point here, and moved climate texts to Discussion.
3. We clarified the end of introduction to underscore that the aim is to compare methods.

• METHODS
o Could you show the species accumulation curve as supplemental information, with the hole composition as well.
Response:
1. We added rarefaction curves for each trap type and pooled sample (new Fig. 2). This shows that only pitfall trap catches saturated, whereas light and Malaise traps continued to trap new species, which was reflected in the total sample as well. We omitted the initial accumulation analysis as it contained a calculus error.
2. We also omitted the PERMANOVA approach as it was done with the pooled sample and intended to compare elevations only, whereas our aim was, following the reviewers' advice, to compare methods (and elevation is a "side product"). Additionally, this analysis did not produce anything new as compared to the other analyses: elevations are compared in much more detailed ways using GLMM, NMDS, MRT and IndVal. So we prefer to leave out also this whole-data curve.
o What is the letters A, B, C, D, E, F, G, H? => If they are Stands you have to explain better.
Response: Thanks for pointing out this lack of clarity. These are indeed study stands, as we now say in the figure text (stands denoted with letters A-J), and cite Table 1 where these are shown.
o Get the GLMM model more explicit.
Response: The full GLMM output is shown in the Supplementary materials. We believe that an average reader will appreciate a simple, at-glance presentation of initially quite diverse analysis outputs. However we are ready to present the big GLMM tables in the manuscript (and not in Supplementary) if the reviewer and editors require that.
o Figure 2 has some problems. Standardize the axes so you can better compare the results. It would be good to add a table with richness values by the mountain, altitude, and trap.
Response:
1. While axis standardization is generally a valid point, in our case it would lead to many of the pitfall-trap columns to become too short to be distinguishable. Therefore, we did not change the figure but added a note to figure legend that the X axis scales are different.
2. The suggested table would be our initial data set, which we will provide in the journal's open access database if our manuscript gets accepted.
o Figure 3 identifies the species, by name in the legends.
Response: we assume that the reviewer would like us to show the species identities for each column. We added an Appendix table that shows these species in rank order per method, with shared species highlighted.

• RESULTS
o NMDS- The 1800 altitude separation becomes evident only in the permanova.
Response: it is indeed common that differences are not that clear in a 2-dimensional graph, whereas permanova considers also other dimensions in the data. The separation is very clear in Table 3, and particularly regarding light traps, the result is extremely clear even without these, as the 1800-m and the other scores do not overlap (Fig. 4). We believe that, after rearranging (and partly rewriting) the methods and statistical outputs, the impact of elevation is now considerably more clearly demonstrated.

• DISCUSSION
o Discuss more the implications of the differences of the methods.
Response: We added notes on practical aspects of using these methods to Discussion, and organized the text better to highlight the use. However we need to underscore that quite little is known about the captured species, which limits conservation implications. We highlight this too in Discussion.
o What is the bias towards decision-making using only a method and possible vantages to use more than one method?
Response: this is an important point. We clarify the end of Discussion as follows: "This is an important aspect as land-use or political decision making should ideally be based on as holistic picture as possible about biodiversity to avoid species losses or

Powered by Editorial Manager® and ProduXion Manager® from Aries Systems Corporation

unwanted changes in ecosystem functioning."
o If you are talking about trap efficiency, since pitfall with a lesser richness capture differences in all elevations, and combinations I would assume this is the most efficient method (if you emphasize more the different biologies related to each trap you can sell better the idea of complementary survey methods).
Response: this may indeed be the case, and/or many species captured using pitfall traps may be too generalistic in their life histories that they may not be that relevant for conservation, after all, which would make pitfall traps less optimal for studying elevational or climate gradients. But of course, to really determine conservation relevance would require knowledge about species identities, and population sizes, locations and long-term trends. But the main reason for recommending the use of multiple methods is based on complementarity, as explained above.
Few sentences on species ecology has been added with the details of generalists and specialist species.
o Line 263: wind direction is duplicated.
Response: thanks for pointing this out. The other was deleted.

• CONCLUSION
o The recommendation part needs to be better, talk about the biology of beetles and how this would affect the methodology choice of your research. Remember that methodology should adequate to the question made.
Response: this is a valid criticism. We added discussion about links between species life histories and collecting methods (see Conclusions in the end of Discussion).
o Improve the text so it is not a resume of the paper, you can make a more thorough recommendation, a guide for decision making. As it is now the recommendation is too subtle.
Response: this is valid criticism. We tried to improve this with a stronger statement. See our proposed addition to your comment regarding decision making.

• FIGURES
o Figura 1 – Put the names of the mountains in the first image. Identify the images A, B e C.
Done accordingly and new figure has been added.
o Figure 4: Use different colors for elevation and different symbols for mountains.
Response: we prefer not to add more information to this already quite complex graph, plus mountains were not really of interest as our focus was on methods and how they reflect altitudes.

• TABLES
o Why only 500m in this table? Explain this better in the methods.
This shows a distinction between 500 m and the other Altitudes. The other altitudes are compared to the rest below the 500 row. We clarify this in Methods.

• GENERAL
o Text organization of methods and results is really good and with easy understanding.
Response: thank you for this comment.

Reviewer #2

The paper titled “Community composition and indicator functioning of beetles across environmental gradients in Peninsular Malaysia” has important contributions for studies on environmental gradients. The comparison of methods is interesting and this is the main objective of the work. The authors did a huge sampling effort and made a good analysis proposal. However, the paper has some issues and some improvements are needed. Most issues are related to the organization of ideas. Also, the paper has some writing issues. The English is good, but they need to improve the writing. I made several comments along the text trying to highlight some critical points, but I recommend a full text revision. Two specific things that were hard to understand were the sampling design and the analysis descriptions, for which they need to provide more information. Moreover, they need to improve the model diagnostics. I made some suggestions in the Material and Methods. The figures, specially the map, need improvements. Therefore, I recommend major revision and suggest that the authors incorporate the suggestions made throughout the text.
Response: thank you for these comments, which are all important. We attempted to

clarify the text and reorganize the structure according to both referees' comments (see also responses to referee 1). We went through all comments placed in the manuscript pdf, thank you for such thorough job. Specifically, we made the following major changes (linguistic or clarity-related suggestions were mostly accepted as such).

Abstract
The indicator hypothesis was removed while strengthening the focus on methods (see reviewer #1). We now use altitude, not elevation, throughout the text, and name the compared traps in Abstract. PERMANOVA was removed (see reviewer #1). From results in Abstract, we removed analysis names and clarified the tested characteristics of beetle assemblages.

Introduction
Thank you for pointing out Rahbek et al. 2019, which we now cite in Introduction.
We rewrote the global hotspot sentence as suggested.
Request for clarifying altitude and climate: this part was moved to Discussion, where we rewrote it to suggest that changes in temperature, etc. with altitude might have some similar effects as global climate change, giving a reason to study mountain slopes.
The messy paragraph 2: we agree in that we had packed too much scattered information into this. We therefore rewrote the paragraph to be better linked with our aim, which is methodological comparison, whereas altitude is a side effect.
We kept the word "largely" as the determination concerns most but not all responses. However, we went through the whole text to omit similar, unwarranted words. We too think they should be avoided.
The "three mentioned trap types": we now mention only the three studied trap types in the previous paragraph. For some reason the initial text included flight intercept traps, which is now deleted.
We moved the sentences that the reviewer indicated to appropriate sections.
Our intention is not to assess sampling efficiency but rather the consistency of methods in reflecting altitude. Hence, we did not change the initial sentence.
With these edits we believe that the text logic is now much clearer.

Methods
It is an excellent idea to reorganize this section according to study questions. So we rearranged Methods, Results and Discussion. Also, as we noted that the analyses actually describe four (instead of three) viewpoints, we adjusted the research questions accordingly.
We adopted all linguistic suggestions by the reviewer.
Regarding criticism on the trap section containing messy details, we reorganized the presentation as follows: (1) general design (mountains, altitudes), (2) trap types and their numbers, (3) how traps were placed per altitude, and (4) what were the trapping periods. We clarified the trapping-period length (one day per month), samples from different periods were pooled, and that each trap, at least 150 m from the nearest other trap, is considered a replicate in analysis. We also clarified that we had two mountains, each with four altitudes, and each such combination had 2 light, 2 Malaise and 5 (groups of 5) pitfall traps, making up initially 72 samples.
We restructured and clarified the trapping protocol to follow the reviewer's advice.
We ran all statistical analyses using 69 samples without any exceptions, as we say in the beginning of statistics descriptions. We also refer to Table 1 where these samples are linked with the ten stands shown in Fig. 1. Rarefaction is a standard method nowadays, and was calculated for each of the 69 samples (note: no exceptions).
GLMM: we see the reviewer's point in adding more explanatory variables. It is a common problem in mountain research that many things change in concert. Regrettably we do not have additional data, as no forest inventory, for example, was done. It is also worth noting that more complex models result in poorer generalizations. Nevertheless, we clearly acknowledge this shortcoming and propose alternative or, rather, complementary explanations in Discussion.
Thank you for suggesting an alternative GLMM tool. However, our approach appeared robust with alternative approaches, and we also used a well-established assessment of data normality and model robustness, so we did not change this analysis.
Turnover: The important thing is that light and Malaise traps shared about half of their abundant species, whereas pitfall traps produced rather unique data. It also captures clear changes with elevation, and is easy to understand. However, statistical power for

	this aspect comes from permanova for methods and elevations. We reorganized the text to better underline these results, and link them clearer to the turnover analysis.<br><br>Results:<br>The initial accumulation calculi had an error, plus our intention is really to compare trap types, so we recalculated accumulation curves for each trap type for each of the two mountains. These are now shown as a figure, Appendix 1.<br>Assemblage structure and turnover: we have now clarified and restructured Methods and Results as there was apparently some lack of clarity. The statements regarding similarity are partly based on the rank-abundance plots, but partly also on permutational multivariate ANOVA (see rewritten Results and Table 3).<br><br>Discussion:<br>Abiotic versus biotic factors affecting species: we do not have data for these; therefore we say "might". At a general level, of ecology, biotic and abiotic factors impact species. The referee has often commented our speculations and suggested us to provide data. We would be happy to do so, had we some. So neither do we have such data nor large libraries on the ecology of the sampled species, which we highlight in Discussion. For these reasons we express us cautiously. But we believe that even speculative sections may function as seeds for future research.<br>We did not use the general forest type in analyses because technically it would be the same thing as using altitude as a factor. But of course, as we say clearly in Discussion and in our above comments, altitude probably reflects many things and not just height from sea level; one is habitat type for sure, others are temperature, windiness, etc.<br>We now refer to Table 4 to show which species indicated what, according to the referee's suggestion.<br>We moved the statement about altitudinal variation related to mountain and method to the beginning of the paragraph. Thanks for pointing out one of the key findings!<br>We see the point that our discussion regarding taxonomic level may sound repeating. However, we need to make a point here: specimen of genus A at two different locations may in reality be two different species, whereas if we consider them to be the same (as technically we do so in our analysis), we will probably miss some true location effects.
**Additional Information:**	
**Question**	**Response**
**Financial Disclosure**<br><br>Enter a financial disclosure statement that describes the sources of funding for the work included in this submission. Review the submission guidelines for detailed requirements. View published research articles from *PLOS ONE* for specific examples.<br><br>This statement is required for submission and **will appear in the published article** if the submission is accepted. Please make sure it is accurate.	We thank the University of Malaya for financing this study (grant numbers RP004E/13SUS and PG059/2014B).

**Unfunded studies**<br>Enter: *The author(s) received no specific funding for this work.*<br><br>**Funded studies**<br>Enter a statement with the following details:<br>• Initials of the authors who received each award<br>• Grant numbers awarded to each author<br>• The full name of each funder<br>• URL of each funder website<br>• Did the sponsors or funders play any role in the study design, data collection and analysis, decision to publish, or preparation of the manuscript?<br>• **NO** - Include this sentence at the end of your statement: *The funders had no role in study design, data collection and analysis, decision to publish, or preparation of the manuscript.*<br>• **YES** - Specify the role(s) played.<br><br>* typeset	
**Competing Interests**<br><br>Use the instructions below to enter a competing interest statement for this submission. On behalf of all authors, disclose any competing interests that could be perceived to bias this work—acknowledging all financial support and any other relevant financial or non-financial competing interests.<br><br>This statement is required for submission and **will appear in the published article** if the submission is accepted. Please make sure it is accurate and that any funding sources listed in your Funding Information later in the submission form are also declared in your Financial Disclosure statement.<br><br>View published research articles from *PLOS ONE* for specific examples.	NO authors have competing interests

**NO authors have competing interests**<br><br>Enter: *The authors have declared that no competing interests exist.*<br><br>**Authors with competing interests**<br><br>Enter competing interest details beginning with this statement:<br><br>*I have read the journal's policy and the authors of this manuscript have the following competing interests: [insert competing interests here]*<br><br>* typeset	
**Ethics Statement**<br><br>Enter an ethics statement for this submission. This statement is required if the study involved:<br><br>• Human participants<br>• Human specimens or tissue<br>• Vertebrate animals or cephalopods<br>• Vertebrate embryos or tissues<br>• Field research<br><br>Write "N/A" if the submission does not require an ethics statement.<br><br>General guidance is provided below. Consult the submission guidelines for detailed instructions. **Make sure that all information entered here is included in the Methods section of the manuscript.**	The collection permits for Fraser's Hill and Genting Highlands were obtained from Forest Department of Malaysia.

**Format for specific study types**<br><br>**Human Subject Research (involving human participants and/or tissue)**<br>• Give the name of the institutional review board or ethics committee that approved the study<br>• Include the approval number and/or a statement indicating approval of this research<br>• Indicate the form of consent obtained (written/oral) or the reason that consent was not obtained (e.g. the data were analyzed anonymously)<br><br>**Animal Research (involving vertebrate animals, embryos or tissues)**<br>• Provide the name of the Institutional Animal Care and Use Committee (IACUC) or other relevant ethics board that reviewed the study protocol, and indicate whether they approved this research or granted a formal waiver of ethical approval<br>• Include an approval number if one was obtained<br>• If the study involved *non-human primates*, add *additional details* about animal welfare and steps taken to ameliorate suffering<br>• If anesthesia, euthanasia, or any kind of animal sacrifice is part of the study, include briefly which substances and/or methods were applied<br><br>**Field Research**<br><br>Include the following details if this study involves the collection of plant, animal, or other materials from a natural setting:<br>• Field permit number<br>• Name of the institution or relevant body that granted permission	
**Data Availability**<br><br>Authors are required to make all data underlying the findings described fully available, without restriction, and from the time of publication. PLOS allows rare exceptions to address legal and ethical concerns. See the PLOS Data Policy and FAQ for detailed information.	Yes - all data are fully available without restriction

A Data Availability Statement describing where the data can be found is required at submission. Your answers to this question constitute the Data Availability Statement and **will be published in the article**, if accepted.<br><br>**Important:** Stating 'data available on request from the author' is not sufficient. If your data are only available upon request, select 'No' for the first question and explain your exceptional situation in the text box.<br><br>Do the authors confirm that all data underlying the findings described in their manuscript are fully available without restriction?	
**Describe where the data may be found in full sentences. If you are copying our sample text, replace any instances of XXX with the appropriate details.**<br><br>• If the data are **held or will be held in a public repository**, include URLs, accession numbers or DOIs. If this information will only be available after acceptance, indicate this by ticking the box below. For example: *All XXX files are available from the XXX database (accession number(s) XXX, XXX.*).<br>• If the data are all contained **within the manuscript and/or Supporting Information files**, enter the following: *All relevant data are within the manuscript and its Supporting Information files.*<br>• If neither of these applies but you are able to provide **details of access elsewhere**, with or without limitations, please do so. For example:<br><br>*Data cannot be shared publicly because of [XXX]. Data are available from the XXX Institutional Data Access / Ethics Committee (contact via XXX) for researchers who meet the criteria for access to confidential data.*<br><br>*The data underlying the results presented in the study are available from (include the name of the third party*	Data is attached as supplementary information along with the manuscript.

and contact information or URL).<br>• This text is appropriate if the data are owned by a third party and authors do not have permission to share the data.<br><br>* typeset	
Additional data availability information:	Tick here if the URLs/accession numbers/DOIs will be available only after acceptance of the manuscript for publication so that we can ensure their inclusion before publication.

Powered by Editorial Manager® and ProduXion Manager® from Aries Systems Corporation

Manuscript Click here to access/download;Manuscript;Manuscript.docx

Full Title:

The functioning of different beetle (Coleoptera) sampling methods across altitudinal gradients in Peninsular Malaysia

Short Title: Beetle sampling across altitudes in Malaysia

Muneeb M. Musthafa[1,2,3 *], Fauziah Abdullah[2,3,4] and Matti J. Koivula[5]

[1]Department of Biosystems Technology, Faculty of Technology, South Eastern University of Sri Lanka, University Park, Oluvil, #32360, Sri Lanka.

[2]Institute of Biological Science, Faculty of Science, University of Malaya, 50603, Kuala Lumpur, Malaysia.

[3]B513, Toxicology Lab, Institute of Postgraduate Studies, University Malaya, 50603, Kuala Lumpur, Malaysia.

[4]Center of Biotechnology in Agriculture, University Malaya, 50603, Kuala Lumpur, Malaysia

[5] Natural Resources Institute Finland (LUKE), P. O. Box 2, FI-00791 Helsinki, Finland.

* Corresponding author: muneeb@seu.ac.lk

Author contributions

Muneeb M. Musthafa: Initial research idea, fieldwork, data curation, manuscript writing.

Fauziah Abdullah: Conceptualization of the research and methodological design, manuscript writing.

Matti J. Koivula: Statistical analysis, manuscript writing, and technical editing.

Abstract

Biodiversity research relies largely on knowledge about species responses to environmental gradients, assessed using some commonly applied sampling method. However, the consistency of detected responses using different sampling methods, and thus the generality of findings, has seldom been assessed in tropical ecosystems. Hence, we studied the response consistency and indicator functioning of beetle assemblages in altitudinal gradients from two mountains in Malaysia, using Malaise, light, and pitfall traps. The data were analyzed using generalized linear mixed-effects models (GLMM), non-metric multidimensional scaling (NMDS), multivariate regression trees (MRT), and indicator species analysis (IndVal). We collected 198 morpho-species of beetles representing 32 families, with a total number of 3,052 individual beetles. We found (1) considerable differences in sample richness and composition between two mountains in pitfall but less so in light or Malaise traps; (2) light traps (but not the other trap types) distinguished high from middle or low altitudes in terms of beetle richness and assemblage composition; and (3) lower altitudes hosted about twice as many indicators as middle or high altitudes, and many species were trap-type specific in our data. These results suggest that the detection of community variation in the environment depends on the sampling method, but also on the analytical approach, suggesting the need to use multiple methods in environmental assessments.

Keywords: biodiversity, altitude, forest, light trap, Malaise trap, pitfall trap, tropical.

Introduction

Most hotspots for the global biodiversity can be found in tropical regions (Mittermeier et al. 2011) which are thus of central importance for conservation. Tropical species often have limited

distributions, particularly those species that occupy higher altitudes at mountain slopes (Cox et al. 2016). The majority of the world's vertebrates, for instance, occupy mountain regions that cover only one-fourth of terrestrial landmass (excluding Antarctica; Rahbek et al. 2019). Such mountain species are often endemic to these regions, and also sometimes such that may not be able to move elsewhere if conditions turn unfavorable for them due to, for example, habitat destruction or climate change (Escobar et al., 2005; Elsen & Tingley 2015). Hence, studies on mountain species communities provide crucial information for global conservation efforts, in addition to these environments being particularly vulnerable to habitat degradation and alterations in landscape structure (Körner 2003; Rangwala & Miller 2012).

Community composition – species, their relative shares, and interactions (biotic responses) – largely determines the general biodiversity response to environmental variation. Communities vary according to, among other aspects, latitude and altitude (Hooper et al. 2005; Ashton et al. 2015; Bouzan et al. 2015). Our understanding regarding this variation relies on some commonly assessed taxonomic groups and associated sampling methods. Similarly, land-use planning or conservation decisions may be based on only a handful of well-known species or a single sampling method. Insects, for instance, are often sampled using light, Malaise, or pitfall traps (e.g., Grootaert et al. 2010). Environmental assessments, however, are often based on just one sampling method, which is assumed to produce a general biodiversity response, but this assumption has seldom been challenged.

Earlier research indicates that the three mentioned trap types differ in their efficiency in capturing specimens and species (e.g., Hansen 1988; Ulyshen et al. 2005; Devigne & De Biseau 2014) though the efficiency could vary with the composition of the local species community determined by, for example, altitude or habitat type. As different methods are known to capture partly different

species (e.g., Missa et al. 2009), their use may, at least theoretically, result in different conclusions and hence applications in land use or conservation.

The objective of the present study is methodological: we assess the consistency of light, Malaise, and pitfall trap samples in reflecting a simple environmental gradient, altitude. More specifically, we compare the three sampling methods across the altitudinal gradient from four viewpoints:

(1) Assemblage species richness;

(2) Assemblage species-compositional turnover;

(3) Assemblage composition; and

(4) Species associated with different combinations of sampling method and altitude

Materials and Methods

Study sites

The Titiwangsa mountain range dominates the landscape of Peninsular Malaysia. Within this range, we sampled beetles at Fraser's Hill (3°43’ N, 101°45’ E) and Genting Highland (3°25' N, 101°47' E) that are 95 km apart (Fig. 1). Fraser’s Hill mountain tops peak at between 1,000 and 1,800 m a.s.l., whereas the Genting Highland peaks at about 1,800 m a.s.l. In this region, wet and dry seasons cannot be differentiated, as the annual rainfall of 1,800–3,500 mm is distributed throughout the year (Cheong 2013; Ya’cob et al. 2016). The collection permits for Fraser’s Hill and Genting Highlands were obtained from Forest Department of Malaysia.

exclude this comma

Fraser’s Hill, locally known as Bukit Fraser, is a well-preserved, permanently protected nature reserve located at the Raub district of Pahang state. Fraser’s Hill has been developed as a hill

station dating back in 1919 (Freeman 1999), where 90% of the 2,800-ha land area is covered by forests (Kiew 1998; Cheong 2013). Fraser's Hill comprises residential areas, commercial complexes, community services, and recreational facilities. The forests mainly consist of tropical montane cloud forests. Lower montane forests – at about 500–1,200 m a.s.l. – are dominated by Fagaceae and Lauraceae trees, whereas upper montane forests – from about 1,200 to 1,800 m a.s.l. – are dominated by Coniferae, Ericaceae, and Myrtaceae trees (Perumal & Lo 1998).

Genting Highland is perhaps the most disturbed cloud forest in the Malaysian mountains. The summit is covered by amusement parks, casinos, and hotels (Peh et al. 2011). The highest peaks at Genting Highland reach 1,800 m a.s.l, where 96% of the total of 3,965 ha of land is still covered with mostly primary forests (Cheong 2013). Before Genting Highland became an entertainment site, it was an undisturbed forest that could be reached only via jungle trekking (Piggott 1977; Stone 1981).

Sampling methods

We sampled beetles using two Malaise traps, two light traps, and 25 (5 groups of 5) pitfall traps at each altitudes (500 m, 1,000 m, 1,500 m, and 1,800 m a.s.l.) at both mountain slopes. All the traps (or trap groups for pitfall traps) were set at least 150 m apart at a given altitude and were placed at least 150 m from the nearest main road. These roads and a river split the sampled forests into a total of twenty distinctive stands; each of these had at least one type of trap (Fig. 1, Table 1).

Malaise traps consisted of a nylon net connected to a collection jar, half filled with 70% ethanol and attached to a tree branch about one meter above the ground. Light traps had a mosquito net with attached 160-watt mercury bulb connected to a portable Honda EU10i generator. Pitfall traps

were plastic cups (diameter 65 mm, depth 95 mm) partly filled with 70% ethanol and dug into the ground with the rim flush with the soil surface. We placed large dry leaves above each pitfall trap to protect them from litter and rain.

We sampled beetles once per month in October 2014, and March, June, and September 2015. At each collecting date, Malaise and pitfall traps were set for 24 hours, starting at 08:00 AM, and light traps operated from 18:00 to 23:30. In the latter, beetles were obtained manually from the traps using collection bottles and aspirators. We occasionally continued to use the light traps until the next morning at 06:00 AM but did not capture additional beetles.

In statistical analyses, we pooled the four periods for each trap (or trap group for pitfall traps) and consider each trap (or trap group for pitfall traps) as a replicate (initially 72; 4 altitudes and 2 mountains, each with 2 light and 2 Malaise, and 5 groups of pitfall traps).

Identification of specimens

We sorted, counted, and cross-checked all beetle specimens using available keys (Saito 2003; Mohamedsaid 2004; Naomi 2006; Schawaller 2006, 2012; Schimmel & Tarnawski 2007; Assing 2008; Hlaváč et al. 2011; Prathapan & Viraktamath 2011; Ryvkin 2011; Shi et al. 2013; Filippini et al. 2016; Anzaldo 2017; Makranczy 2017; Sasakawa et al. 2017). We confirmed the identification of difficult specimens at collections of the Wildlife Department of Malaysia, University of Malaya, National University of Malaysia, and Forestry Department of Malaysia. The identification of beetles to species is hampered by the lack of experts and species compilations. In the present study, the samples contained at least five species new to science, which will be described in later papers. Clearly, the often-broad taxonomic levels and the shortage of knowledge

about the ecological traits of species must be acknowledged while interpreting results, as similar-looking species might be different in terms of, for example, life cycles, diets, abilities to disperse, and habitat requirements.

Statistical analysis

We had initially 72 samples (16 for light, 16 for Malaise, and 40 for pitfall traps). Three samples, however, produced only 1-2 species and were excluded due to difficulty in calculating pair-wise dissimilarities and richness estimates (one light and two Malaise trap samples). Thus, we ran all analyses with 69 samples (Table 1). We ran the following analyses for the four viewpoints proposed above.

Assemblage richness:

To assess sampling-method dependent variation in species, we subjected the beetle data to a generalized linear mixed-effects model (GLMM; Zuur et al. 2009) to quantify variation according to altitude (500, 1,000, 1,500, or 1,800 m a.s.l.). We ran the model separately for each sampling method (light, Malaise, or pitfall traps; hereafter "Method" for brevity) to assess their similarity in responses to altitude. We subjected the observed species (number of species) and richness estimates based on rarefaction standardization to 5, 10, and 20 individuals, to a GLMM with altitude as a fixed factor and Mountain (Fraser's Hill or Genting Highland; Fig. 1, Table 1) as a random factor (to account for possible spatial autocorrelation) and assumed a Gaussian error distribution. We checked the normality of residuals using Q-Q plots (Supplementary Figure 1). These plots did not indicate major departures from normality, except in the pitfall trap data for the number of species and rarefied richness to 5 individuals. We attempted to solve these by square-

root and log transforming the data, and by applying a quasi-Poisson error distribution on untransformed data (Supplementary Figure 2). The quasi-Poisson error distribution improved the model fit for the number of species but not for the rarefied richness. Nevertheless, as the rarefied richness to 10 and 20 individuals produced broadly similar results, we are confident that our initial analyses are robust.

Assemblage turnover:

To examine possible species turnover according to altitude and Method, we calculated averages and standard errors for 20 most abundant species, separately for each Altitude and Method. We plotted these values according to Altitude, using the rank order of the abundances of species captured using each Method. We evaluated the community turnover, or distinctiveness of samples, between Altitudes using permutational multivariate ANOVA (the adonis function in R package vegan, with Mountain [Fraser's Hill or Genting Highland] as strata; Oksanen et al. 2018). Here, we only considered single, or combinations of subsequent, Altitudes. Thus, for example, a combination 500 + 1000 m was considered but not 500 + 1800 m. As a simple measure of turnover between Methods, we indicated in these plots species that were unique for a given Method, and those species that were shared with 1–2 other Methods. We confirmed this comparison of Methods using permutational multivariate ANOVA, as described above.

.

Assemblage composition:

To examine beetle assemblage composition across Altitudes and Methods, we used two analyses. Firstly, we used non-metric multidimensional scaling (NMDS; Borg & Groenen 2005) to assess variation in community composition. We used Method-specific data sets by applying a Bray-Curtis

dissimilarity matrix. We used the above-described permutational multivariate ANOVA for Altitudes as a confirmation of the NMDS result. Secondly, we subjected the Method-specific beetle data sets to multivariate regression tree analysis (MRT; De'ath 2002) based on the Bray-Curtis dissimilarity matrix. We used altitude and, as our earlier research has indicated differences in beetle faunas between Fraser's Hill and Genting Highland (Musthafa et al. 2018), Mountain as explanatory variables. MRT identifies groups of samples as defined by explanatory variables (Mountain and Altitude) and is not restricted by non-linearities (De'ath 2002). We present the result as a tree of dichotomies, where each dichotomy is based on minimizing the dissimilarity of samples within each tree branch. We report the tree with the lowest cross-validated relative error, following the 1-SE rule (Breiman et al. 2017). Cross-validated relative error provides a better estimate than relative error for the predictive accuracy of the MRT for a new dataset (De'ath 2002).

Species associations with Altitudes and Methods:

To detect species characteristic to particular combinations of Method and Altitudes, we calculated an indicator value (IndVal; Dufrêne & Legendre 1997, De Cáceres et al. 2010) for each species, based on all logical combinations of Method and Altitude. Here, we allowed each Method to appear singly or jointly with 1–2 other Methods, whereas for Altitude we only considered single, or combinations of subsequent, Altitudes, as described above. We restricted the IndVal to species with a total sample of at least five individuals.

Analysis software:

We ran the analyses using R 3.6.1 software (R Core Team, 2019) with lme4 (Bates et al. 2015), vegan (Oksanen et al. 2018), mvpart (Therneau et al. 2014), indicspecies (De Cáceres & Jansen 2016), and car (Fox & Weisberg 2018) packages.

Results

Beetle richness according to Mountain and Altitude

We collected 198 morpho-species of beetles representing 32 families with a total number of 3052 individual beetles (Supporting Information). Nine were identified to species, 143 to genus, and 43 to higher taxonomic levels. We refer to all these as “species” below for convenience. Regarding Method, we collected 107 species using light traps, 127 using Malaise, and 45 using pitfall traps. A total of 135 species were represented by only one Method, whereas 45 had been captured with two and 18 with all three sampling methods. Altogether 98 species were singletons or doubletons, whereas 84 were represented by at least five individuals (Appendix Supplementary materials Table S1). Species accumulation curves based on rarefaction suggested that pitfall traps had captured nearly all potential species at about 200 individuals at both Mountains, whereas the accumulation curves for light and Malaise traps were still very steep at about 400-500 individuals (Fig. 2).

The GLMM performed rather similarly in terms of the four measures of richness, but the three Methods produced partly different results (Table 2, Fig. 2; Supplementary materials, Table S1). The Mountains differed little in terms of light and Malaise trap data but remarkably in pitfall-trap data. The richness measures based on light traps declined with Altitude but only 1,800 m differed significantly from 500 m. The decline with Altitude occurred also in Malaise traps so that all higher Altitudes differed significantly from 500 m. Richness measures based on pitfall traps, on the other hand, peaked at 1,000 m (Table 2, Fig. 2).

Species turnover

The rank-abundance plots of twenty most abundant species (Fig. 4) reflected relatively high similarity between light and Malaise traps, with nine of the 20 most numerous species being shared (grey columns), whereas only one of the 20 dominant species – *Pityogenes* sp1 – in pitfall-trap samples was shared with light- and Malaise-trap samples (white columns). For species identities in this graph, see Appendix Fig. S1. Permutational multivariate ANOVA indicated that also light and Malaise traps were compositionally different; for light vs. Malaise traps, F = 3.21, R2 = 0.11, p = 0.0010; for light vs. pitfall traps F = 9.41, R2 = 0.15, p = 0.0010; and for Malaise vs. pitfall traps F = 10.48, R2 = 0.17, p = 0.0010. Moreover, the plots suggested more abundance or occurrence changes with Altitude in Malaise and light traps, whereas the plots varied less and more erratically in pitfall-trap samples (Fig. 4). Another striking pattern in light-trap samples (Fig. 5, left) was the lack of the 12 most abundant species at 1,800 m. Only 9% of light-trap species and 17% of Malaise-trap species were found in both 500 m and 1,800 m, whereas the percentage was 63 for pitfall-trap samples.

Permutational multivariate ANOVA confirmed compositional changes with Altitude, particularly at 1,800 m, but the three Methods showed different patterns in this respect (Table 3). In light traps, only 1,800 m differed significantly from the other Altitudes; however, all except one combination of 2–3 Altitudes also differed significantly from the rest of the Altitudes. The patterns were similar for Malaise traps, except that different combinations of 2–3 Altitudes differed less commonly from the rest of the Altitudes. Regarding pitfall traps, then, all Altitudes and their combinations (except for 1,000–1,500 m) had distinctive assemblages (Table 3).

Assemblage composition according to Method and Altitude

According to NMDS, samples in light and Malaise traps distinguished 1,800 m from the three lower altitudes, whereas pitfall trap samples formed two sample clusters, of which only the other

distinguished clearly between Altitudes (Fig. 6). Thus, in pitfall-trap samples, Altitude was reflected in Fraser's Hill but not in Genting Highland (on the left and right, respectively, in Fig. 5).

MRT for light-trap data consistently resulted in a one-dichotomy tree (Fig. 6). The only division, where 1,800 m was split from the rest of the Altitudes, explained 47% of the variation in light-trap data, the former being species poor according to branch-specific rank-abundance plots (Fig. 6). Malaise-trap data, on the other hand, suggested that the same dichotomy, based on Altitude, was apparent in Fraser's Hill but not in Genting Highland; again, 1,800 m had fewer species though this was based on only two samples. This two-dichotomy tree explained 81% of the variation in the Malaise trap data (Fig. 6). Also, pitfall trap data suggested that Altitudes could be distinguished at Fraser's Hill but not at Genting highland; here, 1,500 and 1,800 m diverged from the two lower Altitudes that, in turn, were split in the third dichotomy (Fig. 6).

Species associated with different combinations of Method and Altitude

We found 83 indicator species for different combinations of Method and Altitude (Table 4). Thirteen species were commonly caught with two sampling methods (12 for light and Malaise traps, and one with Malaise and pitfall traps). Moreover, eight species indicated pitfall traps across the full altitudinal range (500-1,800 m), whereas we did not detect such altitude-independent indicators for the two other sampling methods. Light traps produced 12 indicators of low (500-1,000 m), six of the middle (1,000-1,500 m) and seven of high (1,500-1,800 m) Altitudes, whereas the respective numbers were 16, four, and six for Malaise and ten, eleven and five for pitfall traps (Table 4). Moreover, three species occurred in pitfall traps across a wider Altitudinal range: two species were found at 500-1,500 m and one at 1,000-1,800 m.

Discussion

Beetle species turnover between methods and across the Altitudinal gradient

We found that light and Malaise trap samples shared about half of the abundant species, whereas pitfall trap samples were distinctive in this respect. Light and Malaise traps may have attracted a shared pool of species that flies actively, whereas pitfall traps capture mostly ground dwellers that seldom fall into the two other types of traps (e.g., Missa et al. 2009). This finding suggests that, at least in the study region, the variation in beetle communities caused by altitude or climatic factors may be better captured if pitfall traps are used together with either light or Malaise traps, whereas a combination of light and Malaise traps may not be equally efficient.

At 1,800 m light (but not Malaise or pitfall) trap samples lost all 12 most abundant species of the total light-trap sample. This loss might have resulted from the captured species pool being more sensitive to altitude-associated changes in abiotic or biotic conditions. Studies comparing climatic conditions associated with altitudinal gradients may provide cues on species responses to the global climate change (Chen et al. 2009; Ashton et al. 2015, 2016; Musthafa et al. 2018; Musthafa & Abdullah 2019); perhaps many of the lower- altitudinal species eventually become more common at 1,800 m following climate change. However, some of the 12 “lost” species were still captured at 1,800 m using Malaise traps, so another explanation may be trap functioning. Perhaps light or wind conditions – or some other factors related to, for example, vegetation or moisture – were different at the highest altitudes, which might, in turn, have impacted the visibility of light traps or flight activity of many species (Mathews & Mathews 1971; McGeachie 1989; Sheikh et al. 2016; Nowinszky & Puskás 2017; Skvarla & Dowling 2017). It would be important to continue beetle monitoring at these mountain tops to see whether the altitudinal distributions of species indeed change with climate and whether the currently dominant mountain-top species persist or disappear.

The relative similarity of rank-abundance plots at different altitudes, particularly for pitfall-trap samples, may have occurred because many dominant species are adapted to a wide range of altitudes (e.g., Nunes et al. 2016) and/or associated temperature or moisture conditions (Gilchrist, 1995; Saavedra et al., 2013). Pitfall traps capture mostly ground dwellers, and field-layer vegetation or soil conditions may thus have kept conditions relatively constant across the altitudinal gradient for these species. Also, climatic factors such as temperature, rainfall, relative humidity, wind velocity, and the amount of light may not have changed uniformly with increasing altitude in our study sites, but the possible altitudinal effect (changes in temperature, atmospheric oxygen, etc.) might have been compensated by, for example, habitat type or topography (Adam & Enning 1996; Adam et al. 2011). These factors might decrease variation in micro-climate (McCain & Grytnes 2001), perhaps through the sheltering effect of forest trees (Barnes et al. 1997).

Beetle community structure across the altitudinal gradient using different methods

Our community analyses suggest that the overall beetle community varied remarkably according to altitude, but the magnitude of this response depended on geographic location (the two mountains) and captured species subset (sampling method). Thus, according to NMDS and MRT, light traps distinguished 1,800 m from lower altitudes, whereas Malaise and pitfall traps reflected primarily differences between the two mountains and only secondarily altitudinal variation. Moreover, and only at Fraser’s Hill, Malaise traps distinguished 1,800 m from the lower altitudes, and pitfall traps distinguished 500, 1,000, and 1,500-1,800 m. However, altitude might partly be a proxy of other environmental variables, such that were not assessed in this study. F[illegible]nstance, the lower (500 and 1,000 m) and higher (1,500 and 1,800 m) altitudes represented different forest types, the former being mixed deciduous and the latter being mixed conifer-heath-myrtle forests.

Indicator species for sampling methods and altitude

Pitfall traps produced eight indicator species that were common at all altitudes, whereas the other sampling methods did not produce such altitude generalists (for identities of these species, see Table 4). This finding is in line with our rank-abundance plots (Fig. 4). Moreover, all sampling methods produced at least some indicators of low (500–1,000 m), intermediate (1,000–1,500 m), and/or high altitudes (1,500–1,800 m), suggesting potential for each method to reflect altitude and thus potentially global changes in climate or land use. Important from a climate perspective, we found nine significant indicators of 1,800 m (four using light, four using Malaise, and one using pitfall traps). Species adapted to these highest mountain tops may face difficulty in survival with predicted climate warming (e.g., Lenoir et al. 2008; Pickering et al. 2008; Dirnböck et al. 2011; Elsen et al., 2020) unless they can adapt or disperse elsewhere. Good dispersal ability of beetles allows them to rapidly move towards higher altitudes with warmer temperature (Bale et al., 2002; Williams & Liebhold, 2002). Moreover, beetles may lengthen their flight-activity period and increase the number of generations produced per year to compensate with changing climate, most notably temperature (Menéndez, 2007; Altermatt, 2010). Moreover, thermal adaptability of species could regulate the distributions and local adaptations of beetles across altitudinal gradients, since temperature intensely influences their flight activity (Calosi et al., 2010; Sunday et al., 2012; García-Robledo et al., 2016). For most Malaysian beetle species, the level of knowledge about such traits is low. Though *Onthophagus* beetles (Coleoptera: Scarabaeidae) in South Korea have been predicted to move to higher altitudes with increasing ambient temperature (Kwon et al. 2015). European studies suggest that the dispersal ability is high for Curculionidae (Bouget et al., 2015) and dung beetles (Gómez et al., 2020). Moreover, some of the species in the present data can be categorized as specialists of narrow temperature ranges, such as *Orphnebius* species (Staphylinidae), and *Rhadinomerus* species (Curculionidae), whereas *Brachypeplus* species may

be considered a generalist in terms of temperature conditions of the studied mountain slopes (Musthafa, 2018). On the other hand, *Inopeplus* (Salpingidae), *Macrotomoderus* (Anthicidae), *Paederinae* (Staphylinidae) and *Pentagonica* species (Carabidae) are considered temperature specialists and only *Stenus* species (Staphylinidae) can be considered as a temperature-range generalist (Musthafa, 2018).

The precautionary principle calls for mitigation of climatic or land-use alterations at high altitudes (Gallou et al. 2017). In our study area, for instance, the two mountains hosted very different beetle communities, which perhaps partly reflects different intensities of land use. The studied sampling methods probably differ in required labor, relative capturing efficiency, and captured species pool. The differences, reported here, nevertheless suggest that the use of more than one location and several sampling methods are desirable in environmental assessments (Lamarre et al. 2012; Devigne & De Biseau 2014; Abdullah & Musthafa 2019).

Research caveats

Taxonomic constraints may have affected our results to some extent, as most taxa had been identified to family or genus levels only. Thus, two individuals of a given taxon being captured using two methods or at different altitudes might in reality be two different species. However, this possibility rather masks than exaggerates the true community responses. We are therefore confident that the sampling methods and altitudes truly differed in beetle communities, but the differences would have been more pronounced had we been able to identify everything to species.

Conclusions

The location of the present study, Malaysia, belongs to the global biodiversity hotspot of Sundaland, yet little is known about the invertebrate diversity of its mountains (Myers et al. 2000,

Doll et al. 2014). Our data contribute to fulfilling this knowledge gap. However, our results are also applicable to tropical species conservation: they provide evidence for adaptations of many species to particular altitudes and, more importantly, differences in beetle samples between collecting methods. The three sampling methods reflected the altitudinal gradient in different ways. This, in turn, calls for caution in interpretations of data based on only one method and thus on a narrow range of species life histories. We, therefore, recommend the use of multiple collecting methods in environmental impact assessments on biodiversity, whenever possible. This is particularly important in land-use or political decision making, which should ideally be based on a holistic picture of biodiversity to avoid unwanted species losses or changes in ecosystem functioning.

Acknowledgments

We thank Mohd Shukri Mohd Sabri and Davindram A/L Rajendram for assistance in beetle sampling. We also acknowledge the Universiti Kebangsaan Malaysia and Forestry Department of Malaysia for supporting this study, and Marcelo Bruno Pessôa and an anonymous reviewer for constructive comments.

References

Abdullah F, Musthafa MM. Beetle abundance, diversity and species richness of Pulau Pangkor, Perak, Malaysia. Malaysia Forester 2019; 82: 247-270.

Adam JH, Enning FL. Altitudinal zonation of tropical rain forest at Danum Valley Research Centre, Sabah, Malaysia. Malaysian J Sci 1996; 17A: 25-35.

Adam JH, Ming LK, Juhari MAA, Jaluluddin MA, Idris WMR, Othman AR, Tarmiddzi SNA. Cluster Analysis of Submontane Forest along Western Slope of Frasers' Hill Research Centre in Raub District, Malaysia. Bangladesh J Bot 2011; 40: 121-132.

Altermatt F. Climatic warming increases voltinism in European butterflies and moths. Proc Biol Sci. 2010; 277(1685):1281-1287. doi:10.1098/rspb.2009.1910.

Anzaldo SS. Review of the genera of Conoderinae (Coleoptera, Curculionidae) from North America, Central America, and the Caribbean. ZooKeys 2017; 683: 51-138. https://doi.org/10.3897/zookeys.683.12080.

Ashton LA, Nakamura A, Basset Y, Burwell CJ, Cao M, Eastwood R, et al. Vertical stratification of moths across elevation and latitude. J Biogeogr 2016; 43: 59-69. https://doi.org/10.1111/jbi.12616

Ashton LA, Odell EH, Burwell CJ, Maunsell SC, Nakamura A, McDonald WJF, Kitching RL. Altitudinal patterns of moth diversity in tropical and subtropical Australian rainforests. *Austral Ecol* 2015; 41: 197-208. https://doi.org/10.1111/aec.12309.

Assing V. Four new species and additional records of Palaearctic *Sunius*, with two new synonymies (Coleoptera: Staphylinidae: Paederinae). *Beiträge zur èntomologie* 2008; 58: 455-470. https://doi.org/10.21248/contrib.entomol.58.2.455-470.

Bale JS, Masters GJ, Hodkinson ID, Caroline A, Martijn BT, Valerie KB, … John BW. Herbivory in global climate change research: Direct effects of rising temperature on insect herbivores. Global Change Biology 2002; 8: 1-16. https://doi.org/10.1046/j.1365-2486.2002.00451.x

Baniya CB, Solhøy T, Gauslaa Y, Palmer MW. The elevation gradient of lichen species richness in Nepal. The Lichenologist 2010; 42: 83-96. https://doi.org/10.1017/S0024282909008627

Barnes BV, Zak DR, Denton SR, Spurr SH. Forest ecology. 4th ed. John Wiley & Sons. 1999.

Barzani GM, Sahibin AR, Adam JH, Zulfahmi AR. The physical environment. Bukit Fraser crown of the Titiwangsa Range. In: Latiff, A., Maimon, A., Norhayati, A. & Adam, J.H., editors. Bukit Fraser Research Centre, Universiti Kebangsaan Malaysia & Fraser's Hill Development Corporation, Fraser's Hill Development Corporation, Raub, Malaysia; 2009. pp 9-16.

Bouget, C., Brin, A., Tellez, D. et al. Intraspecific variations in dispersal ability of saproxylic beetles in fragmented forest patches. Oecologia 2015; 177: 911–920. https://doi.org/10.1007/s00442-014-3162-9

Bouzan AM, Flinte V, Macedo MV, Monteiro RF. Elevation and temporal distributions of Chrysomelidae in southeast Brazil with emphasis on the Galerucinae. ZooKeys 2015; 547: 103-117. https://doi.org/10.3897/zookeys.547.9723

Brehm G, Colwell RK. Kluge J. () The role of environment and mid-domain effect on moth species richness along a tropical elevational gradient. Glob Ecol Biogeogr 2007; 16: 205-219. https://doi.org/10.1111/j.1466-8238.2006.00281.x

Bates D, Maechler M, Bolker B, Walker S. Fitting linear mixed effects models using lme4. J Stat Softw 2015; 67: 1-48. http://dx.doi.org/10.18637/jss.v067.i01

Borg I, Groenen P. Modern multidimensional scaling: theory and applications. 2nd ed. Springer-Verlag, New York. 2005

Breiman L, Friedman JH, Olshen RA, Stone CG. Classification and regression trees. CRC Press, Boca Raton, Florida. 358 pp. 1998.

Calosi P, Bilton DT, Spicer JI, Votier SC, Atfield A. What determines a species' geographical range? Thermal biology and latitudinal range size relationships in European diving beetles (Coleoptera: Dytiscidae). J Anim Ecol 2010; 79: 194–204. DOI: 10.1111/j.1365-2656.2009.01611.x.

Chao A, Jost, L. Coverage-based rarefaction and extrapolation: standardizing samples by completeness rather than size. Ecol 2012; 93(12): 2533–2547.

Chen IC, Shiu HJ, Benedick S, Holloway JD, Chey VK, Barlow HS, Hill JK, Thomas CD. Elevation increases in moth assemblages over 42 years on a tropical mountain. PNAS 2009; 106: 1479-1483. https://doi.org/10.1073/pnas.0809320106

Cheong C. Fraser's Hill - A Lush Highland Hideaway. WWF Malaysia, Petaling Jaya. 2013.

Cox CB, Moore PD, Ladle R. Biogeography: an ecological and evolutionary approach. Wiley Blackwell. 2016.

De Cáceres M, Legendre P, Moretti M. Improving indicator species analysis by combining groups of sites. Oikos 2010; 119: 1674-1684. DOI: 10.1111/j.1600-0706.2010.18334.x

De Cáceres M, Jansen F. Package Indicspecies. 2016. URL https://CRAN.R-project.org/package=indicspecies.

De'ath G. Multivariate regression trees: a new technique for modeling species–environment relationships. Ecol 2002; 83(4): 1105-1117. https://doi.org/10.2307/3071917

De'ath G, Therneau TM, Atkinson B, Ripley B, Oksanen J. mvpart: Multivariate partitioning. 2014. URL https://mran.microsoft.com/snapshot/2014-12-11/web/packages/mvpart/index.html

Devigne C, De Biseau J-C. Urban ecology: comparison of the effectiveness of five traps commonly used to study the biodiversity of flying insects. Biodivers J 2014; 5: 165-174.

Doll HM, Butod E, Harrison RD, Fletcher C, Kassim AR, Ibrahim S, Potts MD. Environmental and geographic factors driving dung beetle (Coleoptera: Scarabaeidae: Scarabaeinae) diversity in the dipterocarp forests of Peninsular Malaysia. Raffles Bull Zool 2014; 62: 549-560.

Dufrêne M, Legendre P. Species assemblages and indicator species: the need for a flexible asymmetrical approach. Ecol Monogr 1997; 67(3): 345-366. https://doi.org/10.1890/0012-9615(1997)067[0345:SAAIST]2.0.CO;2

Dirnböck T, Essl F, Rabitsch W. Disproportional risk for habitat loss of high-altitude endemic species under climate change. Glob Chang Biol 2011; 17: 990-996. https://doi.org/10.1111/j.1365-2486.2010.02266.x

Elsen PR, Tingley MW. Global mountain topography and the fate of montane species under climate change. Nat Clim Chang 2015; 5: 772-776. https://doi.org/10.1038/nclimate2656

Elsen, P.R., Monahan, W.B. & Merenlender, A.M. Topography and human pressure in mountain ranges alter expected species responses to climate change. Nat Commun 2020; 11: 1974. https://doi.org/10.1038/s41467-020-15881-x

Escobar F, Lobo JM, Halffter G. Altitudinal variation of dung beetle assemblages in the Colombian Andes. Glob Ecol Biogeogr 2005; 14: 337-347. https://doi.org/10.1111/j.1466-822X.2005.00161.x

Filippini V, Micó E, Galante E. Checklist and identification key of Anomalini (Coleoptera, Scarabaeidae, Rutelinae) of Costa Rica. ZooKeys 2016; 621: 63-136. https://doi.org/10.3897/zookeys.621.7565

Fox J, Weisberg S. An R companion to applied regression. 3rd edition. Sage Publications Inc., California. 2019.

Gallou A, Baillet Y, Ficetola GF, Despres L. Elevational gradient and human effects on butterfly species richness in the French Alps. Ecol Evol 2017; 7(11): 1-10. https://doi.org/10.1002/ece3.2803.

García-Robledo C, Kuprewicz EK, Staines CL, Erwin TL, Kress WJ. Limited tolerance by insects to high temperatures across tropical elevational gradients and the implications of global warming for extinction. PNAS 2016: 113; 680-685. doi: 10.1073/pnas.1507681113

Gilchrist GW. Specialists and generalists in changing environments. I. Fitness landscapes of thermal sensitivity. Am Nat 1995; 146: 252-270.

Gómez VCG, Verdú JR, Zurita GA. Thermal niche helps to explain the ability of dung beetles to exploit disturbed habitats. Sci Rep 2020; 10: 13364. https://doi.org/10.1038/s41598-020-70284-8

Hlaváč P, Newton AF, Maruyama M. World catalogue of the species of the tribe Lomechusini (Staphylinidae: Aleocharinae). Zootaxa 2011; 3075: 1-151.

Hooper DU, Chapin FS III, Ewel JJ, Hector A, Inchausti P, et al. Effects of biodiversity on ecosystem functioning: a consensus of current knowledge. Ecol Monogr 2005; 75: 3-35. https://doi.org/10.1890/04-0922.

Kessler M. Elevational gradients in species richness and endemism of selected plant groups in the central Bolivian Andes. Plant Ecol 2000; 149: 181-193.

Kiew R. The seed plant flora of Fraser's Hill, Peninsular Malaysia: With special reference to its conservation status. Research pamphlet No. 21. Forest Research Institute of Malaysia (FRIM), Kuala Lumpur, Malaysia. 1998.

Kohler T, Wehrli A, Jurek M. Mountains and climate change: A global concern. Sustainable Mountain Development Series. Bern, Switzerland, Centre for Development and Environment (CDE), Swiss Agency for Development and Cooperation (SDC) and Geographica Bernensia. 2014.

Koivula MJ, Chmaberlain DE, et al. Breeding bird species diversity across gradients of land use from forest to agriculture in Europe. Ecogeg 2018; 41: 1331-1344. https://doi.org/10.1111/ecog.03295

Körner C. Alpine plant life. Functional plant ecology of high mountain ecosystems. 2nd edition. Springer, Berlin. 2003.

Kumar A, Longino JT, Colwell RK, O'Donnell S. Elevational patterns of diversity and abundance of eusocial paper wasps (Vespidae) in Costa Rica. Biotropica 2009; 41, 338-346. https://doi.org/10.1111/j.1744-7429.2008.00483.x.

Kwon T-S, Lee CM, Kim S-S. Prediction of abundance of beetles according to climate warming in South Korea. J Asia Pac Biodivers 2015; 8: 7-30. https://doi.org/10.1016/j.japb.2015.01.001.

Laliberte E, Wells JA, DeClerck,F… Mayfield, MM. Land-use intensification reduces functional redundancy and response diversity in plant communities. Ecol Lett 2009; 13: 76-86. https://doi.org/10.1111/j.1461-0248.2009.01403.x

Lamarre GPA, Molto Q, Fine PVA, Baraloto C. A comparison of two common flight interception traps to survey tropical arthropods. ZooKeys 2012; 216: 43-55. https://doi.org/10.3897/zookeys.216.3332

Lenoir J, Gegout JC, Marquet PA, de Ruffray P, Brisse H. A significant upward shift in plant species optimum elevation during the 20th century. Science 2008; 320: 1768-1771. DOI: 10.1126/science.1156831.

Makranczy G. Review of the *Anotylus* cimicoides species group (Coleoptera: Staphylinidae: Oxytelinae). Acta Zoolog Academiae Scientiarum Hung 2017; 63: 143-262. DOI: 10.17109/AZH.63.2.143.2017

Mathews RW., Mathews JR. The Malaise Trap: Its Utility and Potential for Sampling Insect Populations. *The Great Lakes Entomologist* 1971; 4(4): Article 4.

McCain CM, Grytnes JA. Elevation gradients in species richness. In Encyclopedia of Life Sciences (ELS). John Wiley & Sons Ltd., Chichester. 2000.

McGeachie WJ.. The effects of moonlight illuminance, temperature and wind speed on light-trap catches of moths. Bull Entomol Res 1989; 79: 185-192.

Menéndez R. How are insects responding to global warming? Tijdschrift voor Entomologie 2007; 150: 355-365.

Mittermeier RA, Turner WR, Larsen FW, Brooks TM, Gascon C. Global Biodiversity Conservation: The Critical Role of Hotspots. In: Zachos, F.E. & Habel, J.C. editors, Biodiversity Hotspots. Springer-Verlag, Berlin-Heidelberg. 2011. Pp. 3-22.

Mohamedsaid MS. Catalogue of the Malaysian Chrysomelidae (Insecta: Coleoptera). Pensoft Publishers, Bulgaria. 2004.

Musthafa, MM. A comparative study of beetle diversity at different elevations at Fraser's hill and Genting Highland. Ph.D. Thesis. University of Malaya. 2018.

Musthafa MM, Abdullah F. Coleoptera of Genting Highland, Malaysia: Species richness and diversity along the elevations. Arx de Misc Zoology 2019; 17: 45-66. DOI: 10.32800/amz.2019.17.0045.

Musthafa MM, Abdullah F, Sánchez‐Reyes UJ. Comparative study of spatial patterns and ecological niches of beetles in two Malaysian mountains elevation gradients. J Insect Conserv 2018; 22(5-6): 757-769. https://doi.org/10.1007/s10841-018-0099-z

Myers N, Mittermeier RA, Mittermeier CG, da Fonseca GAB, Kent J. Biodiversity hotspots for conservation priorities. Nature 2000; 403: 853-858.

Naomi S. Taxonomic revision of the genus *Stenus* Latreille, 1797 (Coleoptera, Staphylinidae, Steninae) of Japan: Species group of S. indubius Sharp. Jpn J Syst Entomol 2006; 12: 39-120.

Newbold T, et al. Global effects of land use on local terrestrial biodiversity. Nature 2015; 520: 45-50. https://doi.org/10.1038/nature14324

Nowinszky L, Puskás J. Light-Trap Catch of Insects in Connection with Environmental Factors. In: Vonnie D.C. Shields, editors, *Biological Control of Pest and Vector Insects*, InTech Publishers. 2017. pp. 97-117.

Nunes CA, Braga RF, Figueira JEC, Neves FS, Fernandes GW. Dung beetles along a tropical altitudinal gradient: environmental filtering on taxonomic and functional diversity. PLoS ONE 2016; 11: e0157442. https://doi.org/10.1371/journal.pone.0157442

Oksanen J, Blanchet FG, Friendly M, Kindt R, Legendre P, McGlinn D, et al. Package vegan. Community Ecology Package. 2018. URL https://cran.r-project.org, https://github.com/vegandevs/vegan

Peh KS-H, Soh MCK, Sodhi NS, Laurance WF, Ong DF, Clements R. Up in the clouds: Is sustainable use of tropical montane cloud forests possible in Malaysia? Bioscience 2011; 61: 27–38. https://doi.org/10.1525/bio.2011.61.1.8

Perumal B, Lo FSJ. Recorded mountain trees of Peninsular Malaysia - a checklist. World Wildlife Fund Malaysia, Kuala Lumpur, Malaysia. 1998.

Pickering C, Hill W, Green K. Vascular plant diversity and climate change in the alpine zone of the Snowy Mountains, Australia. Biodivers Conserv 2008; 17: 1627-1644. https://doi.org/10.1007/s10531-008-9371-y

Piggott AG. The ferns of Gunung Ulu Kali. Garden Bulletin of Singapore 1977; 30: 31-43.

Prathapan KD, Viraktamath CA. A new species of *Longitarsus* Latreille, 1829 (Coleoptera, Chrysomelidae, Galerucinae) pupating inside stem aerenchyma of the hydrophyte host from the Oriental Region. ZooKeys 2011; 87: 1-10. doi: 10.3897/zookeys.87.1294

R Core Team. R: A language and environment for statistical computing. R Foundation for Statistical Computing, Vienna, Austria. 2019. URL https://www.R-project.org/

Rahbek C, Borregaard MK, Colwell RK, Dalsgaard B, Holt BG, Morueta-Holme N, ... Fjeldsa J. Humboldt's enigma: What causes global patterns of mountain biodiversity? Science 2019; 365(6458): 1108-1113. https://doi.org/10.1126/science.aax0149.

Rangwala I, Miller JR. Climate change in mountains: a review of elevation-dependent warming and its possible causes. Clim Change 2012; 114: 527-547. https://doi.org/10.1007/s10584-012-0419-3

Ryvkin AB. Contributions to the knowledge of *Stenus* (Nestus) species of the *Crassus* group (Insecta: Coleoptera: Staphylinidae: Steninae). Four new species from the Russian Far East with taxonomic notes. Balt J Coleopterol 2011; 11: 57-72.

Saavedra S, Rohr RP, Dakos V, Bascompte J. Estimating the tolerance of species to the effects of global environmental change. Nat Commun 2013; 4: 2350. https://doi.org/10.1038/ncomms3350

Saito M. A New Species of the genus *Tomoderus* (Coleoptera, Anthicidae) from the Ryukyu Islands, Southwest Japan. *Elytra* 2003; 31: 321-323.

Sasakawa K, Kim J-K, Kim J-K, Kubota K. Morphological phylogeny and biogeography of the *Pterostichus raptor* species group (Coleoptera: Carabidae) of ground beetles, endemic to the Korean Peninsula and adjacent islands. J Asia Pac Entomol 2017; 20: 7-12. https://doi.org/10.1016/j.aspen.2016.11.001

Schawaller W. Revision of the Oriental species of the genus Bradymerus Perroud, with descriptions of 29 new species (Coleoptera: Tenebrionidae). Stuttgarter Beiträge zur Naturkunde 2006; 4: 1-64.

Schawaller W. Two new species and new records of the genus *Spinolyprops* Pic, 1917 from the Oriental Region (Coleoptera, Tenebrionidae, Lupropini). ZooKeys 2012; 243: 83-94. DOI: 10.3897/zookeys.243.3879

Schimmel R, Tarnawski D. The species of the genus *Mulsanteus* Gozis, 1875 of Southeast Asia (Insecta: Coleoptera: Elateridae). Genus 2007; 14, 89-172.

Sheikh AH, Thomas M, Bhandari R, Bunkar K. Light trap and insect sampling: an overview. Int J Curr Res 2016; 8(11): 40868-40873.

Shi H, Zhou H, Liang H. Taxonomic synopsis of the subtribe *Physoderina* (Coleoptera, Carabidae, Lebiini), with species revisions of eight genera. Zookeys 2013; 284: 1-129. https://doi.org/10.3897/zookeys.284.3983

Skvarla MJ, Dowling APG. A comparison of trapping techniques (Coleoptera: Carabidae, Buprestidae, Cerambycidae, and Curculionoidea excluding Scolytinae). J Insect Sci 2017; 17(1): 7-20. https://doi.org/10.1093/jisesa/iew098

Staunton KM, Nakamura A, Burwell CJ, Robson SKA, Williams SE. Elevational distribution of flightless ground beetles in the tropical rainforests of North-Eastern Australia. PLoS ONE 2016; 11: e0155826. https://doi.org/10.1371/journal.pone.0155826

Stone BC. The summit flora of Gunung Ulu Kali (Pahang, Malaysia). Federation of Museum Journal 1981; 26(1): 157-162.

Sunday, JM, Bates AE, Dulvy NK. Thermal tolerance and the global redistribution of animals. Nature Clim Change 2002: 2(9); 686–690. https://doi.org/10.1038/nclimate1539.

Williams DW, Liebhold AM. Climate change and the outbreak of two North American bark beetles. Agr Forest Entomol 2002; 4: 87-99.

Ya'cob Z, Takaoka H, Pramual P, Low VL, Sofian-Azirun M. Breeding habitat preference of preimaginal black flies (Diptera: Simuliidae) in Peninsular Malaysia. Acta Tropica 2016; 153: 57-63. doi: 10.1016/j.actatropica.2015.10.007.

Zuur AF, Ieno EN, Walker NJ, Saveliev AA, Smith GM. Mixed effects models and extensions in ecology with R. Springer Science. 2009.

Table 1 Sampling effort at Fraser's Hill (FH) and Genting Highland (GH) mountains. In both mountains, traps were set at four Altitudes: 500, 1,000, 1,500 and 1,800 m a.s.l., in ten stands in both mountains; stands are indicated with letters A-J (compare Fig. 1). The three right-hand columns show the number of traps (or groups of five pitfall traps) in each stand.

Mountain	Altitude	Stand ID	Light	Malaise	Pitfall
FH	500	A	-	-	1
FH	500	B	-	-	4
FH	500	C	1	-	-
FH	1,000	D	1	2	5
FH	1,000	E	1	-	-
FH	1,500	F	1	-	2
FH	1,500	G	-	1	1
FH	1,500	H	1	1	2
FH	1,800	I	1	2	1
FH	1,800	J	1	-	4
GH	500	A	-	1	-
GH	500	B	-	1	-
GH	500	C	2	-	5
GH	1,000	D	-	-	2
GH	1,000	E	2	2	3
GH	1,500	F	-	2	4
GH	1,500	G	-	-	1
GH	1,500	H	2	-	-
GH	1,800	I	2	1	4
GH	1,800	J	-	1	1

Table 2 GLMM summary for Altitude, using Mountain as a random factor, for the number of species and rarefaction standardized number of species for 5, 10 or 20 individuals. Results are shown for light, Malaise and pitfall trap data. Numbers in "%var" columns are percentages explained by a given variable; letters in "Effect" columns show whether a given Altitude differed significantly ($p < 0.05$) and positively (pos) or negatively (neg) from the lowest Altitude (500 m a.s.l.) (in parentheses if marginally significant, i.e., $p < 0.1$) or whether this comparison was non-significant (ns; $p > 0.1$). For full output, see Appendix, Table 1.

	**LIGHT TRAPS**			**MALAISE TRAPS**			**PITFALL TRAPS**	
**Variable/Category**	**%var**	**Effect**		**%var**	**Effect**		**%var**	**Effect**
Number of species								
Mountain	7			4			53	
Altitude	41			78			11	
* 1,000 m a.s.l.		ns			neg			(pos)
* 1,500 m a.s.l.		ns			neg			ns
* 1,800 m a.s.l.		neg			neg			ns
Residuals	52			19			35	
Rarefied richness to 5 individuals								
Mountain	7			6			17	
Altitude	33			70			16	
* 1,000 m a.s.l.		ns			ns			pos
* 1,500 m a.s.l.		ns			ns			ns
* 1,800 m a.s.l.		(neg)			neg			ns
Residuals	60			24			67	
Rarefied richness to 10 individuals								
Mountain	15			7			26	
Altitude	45			72			11	
* 1,000 m a.s.l.		ns			ns			pos
* 1,500 m a.s.l.		ns			ns			ns
* 1,800 m a.s.l.		neg			neg			ns
Residuals	40			21			63	
Rarefied richness to 20 individuals								
Mountain	4			7			28	
Altitude	61			72			12	
* 1,000 m a.s.l.		ns			(neg)			pos
* 1,500 m a.s.l.		ns			neg			ns
* 1,800 m a.s.l.		neg			neg			ns
Residuals	35			21			59	

Table 3 Permutational multivariate ANOVA to assess the distinctiveness of beetle communities at different Altitudes (Alt 500, 1,000, 1,500 or 1,800 m a.s.l., or their logical combinations) as reflected by using three sampling methods (compare Fig. 2).

	**LIGHT TRAPS**							**MALAISE**							**PITFALL**					
**Variable**	**df**	**SS**	**MS**	**F**	**R^2**	**p**		**df**	**SS**	**MS**	**F**	**R^2**	**p**		**df**	**SS**	**MS**	**F**	**R^2**	**p**
Alt 500	1	0.47	0.47	1.28	0.09	0.0659		1	0.40	0.40	1.17	0.09	0.2717		1	0.48	0.48	1.58	0.04	0.0300
Residuals	13	4.75	0.37	0.91				12	4.12	0.34	0.91				38	11.55	0.30	0.96		
Alt 1000	1	0.47	0.47	1.29	0.09	0.1548		1	0.42	0.42	1.23	0.09	0.1528		1	0.97	0.97	3.34	0.08	0.0010
Residuals	13	4.75	0.37	0.91				12	4.10	0.34	0.91				38	11.06	0.29	0.92		
Alt 1500	1	0.53	0.53	1.47	0.10	0.0939		1	0.42	0.42	1.24	0.09	0.1299		1	0.46	0.46	1.51	0.04	0.0390
Residuals	13	4.69	0.36	0.90				12	4.10	0.34	0.91				38	11.57	0.30	0.96		
Total	14	5.22	1.00					13	4.52	1.00					39	12.03	1.00			
Alt 1800	1	1.36	1.36	4.56	0.26	0.0020		1	0.70	0.70	2.21	0.16	0.0030		1	0.76	0.76	2.56	0.06	0.0020
Residuals	13	3.87	0.30	0.74				12	3.82	0.32	0.84				38	11.27	0.30	0.94		
Alt 500-1000	1	0.67	0.67	1.93	0.13	0.0290		1	0.47	0.47	1.40	0.10	0.0699		1	0.97	0.97	3.33	0.08	0.0010
Residuals	13	4.55	0.35	0.87				12	4.05	0.34	0.90				38	11.06	0.29	0.92		
Alt 1000-1500	1	0.98	0.98	3.01	0.19	0.0010		1	0.68	0.68	2.13	0.15	0.0420		1	0.55	0.55	1.82	0.05	0.0070
Residuals	13	4.24	0.33	0.81				12	3.84	0.32	0.85				38	11.48	0.30	0.95		
Alt 1500-1800	1	0.67	0.67	1.93	0.13	0.0380		1	0.47	0.47	1.40	0.10	0.0619		1	0.97	0.97	3.33	0.08	0.0010
Residuals	13	4.55	0.35	0.87				12	4.05	0.34	0.90				38	11.06	0.29	0.92		
Alt 500-1500	1	1.36	1.36	4.56	0.26	0.0020		1	0.70	0.70	2.21	0.16	0.0050		1	0.76	0.76	2.56	0.06	0.0020
Residuals	13	3.87	0.30	0.74				12	3.82	0.32	0.84				38	11.27	0.30	0.94		
Alt 1000-1800	1	0.47	0.47	1.28	0.09	0.0699		1	0.40	0.40	1.17	0.09	0.2408		1	0.48	0.48	1.58	0.04	0.0210
Residuals	13	4.75	0.37	0.91				12	4.12	0.34	0.91				38	11.55	0.30	0.96		

Table 4 Significant ($p < 0.05$) indicators with $n > 4$ in the beetle data. LT = indicator of light traps; MA = indicator of Malaise traps; PT = indicator of pitfall traps; different Altitudes or ranges are shown with numbers (500, 1,000, 1,500, 1,800 m a. s. l.). Column IndVal shows indicator value for each taxon.

Category	Species	IndVal		Category	Species	IndVal
**Single-method indicators**				**Single-method indicators continued**		
LT 500	*Anomala* sp2	0.81		PT	*Harpalus* sp1	0.84
LT 500	*Anomala* sp4	0.76		PT	*Harpalus* sp2	0.91
LT 500	*Anomala* sp6	0.57		PT	*Hiletus* sp1	0.44
LT 500	*Apogonia* sp3	0.79		PT	*Inopeplus* sp1	0.63
LT 500	*Apogonia* sp5	0.79		PT	*Lebia* sp1	0.57
LT 500	*Cicindela* sp2	0.79		PT	*Omonadus* sp1	0.52
LT 500	Mordellidae A	0.80		PT	*Pentagonica* sp1	0.54
LT 500	Scarabeidae M	0.80		PT	Staphylinidae M	0.63
LT 500	Scarabeidae P	0.58		PT 500	*Pterostichus* sp2	0.87
LT 500-1000	*Epepeotes lateralis*	0.52		PT 500-1500	*Spinolyprops* A	0.53
LT 1000	*Apogonia* sp1	0.90		PT 500-1500	Staphylinidae C	0.61
LT 1000	*Cicindela* sp1	0.69		PT 1000	*Actiastes* sp1	0.71
LT 1000-1500	*Anomala* sp1	0.94		PT 1000	*Anotylus* sp2	0.86
LT 1000-1500	*Apogonia* sp2	0.96		PT 1000	*Bledius* sp1	0.78
LT 1500	Lampyridae E	0.70		PT 1000	*Lispinus* sp1	0.84
LT 1500	*Luciola* sp1	0.71		PT 1000	*Orphnebius* sp1	0.84
LT 1500-1800	*Altica* sp1	0.69		PT 1000	*Orphnebius* sp2	0.92
LT 1800	*Cleorina* sp	0.71		PT 1000	Passalidae A	0.44
LT 1800	*Hoplocerambyx spinicornis*	0.87		PT 1000	*Pityogenes* sp1	0.74
LT 1800	*Hydrovatus enigmaticus*	0.97		PT 1000	*Sunius* sp1	0.71
LT 1800	*Illeis* sp2	0.70		PT 1000-1800	*Oxylatus* sp1	0.57
MA 500	Aleocharinae sp1	0.84		PT 1500	*Hiletus* sp2	0.58
MA 500	*Anotylus* sp1	0.94		PT 1500	*Pterostichus* sp1	0.79
MA 500	*Aphthona* sp1	0.98		PT 1500-1800	*Paedarus* sp1	0.69
MA 500	*Brachypeplus* sp1	0.92		PT 1800	*Lebia* sp2	0.57
MA 500	*Brachypeplus* sp3	0.98		PT 1800	*Pterostichus* sp3	0.79
MA 500	*Bradymerus* sp2	0.99				
MA 500	*Epuraea* sp1	0.70		**Multiple-method indicators**		
MA 500	Galerucinae sp1	0.97		MA+LT 500	*Mulsanteus* sp1	0.60
MA 500	*Ischnosoma* sp1	0.95		MA+LT 500	Paederinae sp3	0.68
MA 500	*Lymantor* sp1	0.94		MA+LT 1000	*Sarmydus* sp1	0.71

MA 500	*Lymantor* sp2	0.91		MA+LT 1000-1500	*Strotocera* sp1	0.71
MA 500	*Lymantor* sp3	0.94		MA+LT 1500	Alticinae sp2	0.69
MA 500	*Sinoxylon* sp1	0.99		MA+LT 1500	*Anisandrus* sp1	0.68
MA 500	*Xyleborus* sp1	0.98		MA+LT 1500	*Brachypeplus* sp2	0.65
MA 500-1000	Aleocharinae sp2	0.58		MA+LT 1500	*Colaspoma* sp2	0.71
MA 1000	*Anomala* sp3	0.82		MA+LT 1500	Curculionidae A	0.70
MA 1000-1500	Alticinae sp1	0.70		MA+LT 1500	Meloidae A	0.71
MA 1500	Paederinae sp2	0.66		MA+LT 1500	*Nisotra* sp2	0.71
MA 1500-1800	*Xyleborus* sp2	0.71		MA+LT 1500	*Pityogenes* sp2	0.48
MA 1800	*Anomala* sp5	0.66		MA+PT 500	Aleocharinae sp3	0.62
MA 1800	*Apogonia* sp4	0.69				
MA 1800	*Lymantor* sp4	0.90				
MA 1800	*Xylothrips* sp1	0.68				

Appendix 1. Twenty most abundantly captured species in our data, sorted according to trap-type specific sample sizes; n indicates number of individuals in the data. Light gray: species shared between light and Malaise traps; dark gray: species shared with pitfall, light and Malaise traps.

Light	n	Malaise	n	Pitfall	n
Anomala_sp1	53	Aleocharinae_sp1	88	Orphnebius_sp1	121
Apogonia_sp2	48	Ischnosoma_sp1	62	Pterostichus_sp2	107
Apogonia_sp1	46	Lymantor_sp2	58	Harpalus_sp1	104
Lymantor_sp3	27	Sinoxylon_sp1	43	Paedarus_sp1	99
Apogonia_sp5	23	Bradymerus_sp2	40	Bledius_sp1	80
Apogonia_sp3	22	Lymantor_sp3	38	Harpalus_sp2	78
Lymantor_sp2	22	Apogonia_sp4	31	Pterostichus_sp1	74
Anomala_sp3	21	Brachypeplus_sp3	31	Pterostichus_sp3	73
Lymantor_sp4	21	Brachypeplus_sp1	30	Inopeplus_sp1	70
Colaspoma_sp2	19	Paederinae_sp3	30	Oxylatus_sp1	59
Anomala_sp2	18	Xyleborus_sp1	24	Orphnebius_sp2	56
Anomala_sp4	16	Galerucinae_sp1	22	Pityogenes_sp1	53
Altica_sp1	15	Lymantor_sp4	19	Anotylus_sp2	48
Apogonia_sp4	15	Anomala_sp3	16	Pentagonica_sp1	45
Illeis_sp2	14	Lymantor_sp1	15	Lispinus_sp1	42
Brachypeplus_sp3	13	Anomala_sp1	14	Staphy_C	41
Pityogenes_sp1	13	Apogonia_sp2	13	Aleocharinae_sp3	32
Brachypeplus_sp2	11	Apogonia_sp1	13	Brachypeplus_sp1	31
Cicindela_sp2	11	Alticinae_sp1	13	Lebia_sp2	30
Hydrovatus_enigmaticus	11	Pityogenes_sp1	11	Staphy_M	30

Figure captions

Fig. 1. Study location in Malaysia. Left: map of Malaysia, with major mountain regions shown with gray; the two squares show locations of Fraser's Hill (top right) and Genting Highland (bottom right) mountains. Right-hand graphs show locations of ten study stands in each study mountain (denoted with letters A-J; compare Table 1); black lines show major highways; Altitudes are indicated with numbers (500, 1,000, 1,500 or 1,800 m a.s.l.). In the bottom right-hand graph, the gray patch shows a forest reserve. (The figure is similar but not identical to the original image and is therefore for illustrative purposes only).

Fig. 2. Rarefaction curves for the three trap types and the pooled sample. The end point of each curve indicates the trap-type specific or pooled (total) number of individuals.

Fig. 3. Average samples + SE of four measures of species richness for three sampling methods according to Altitude. Number of species, and rarefaction standardized richness based on 5, 10 and 20 individuals are shown. Note that the X axis length for pitfall trap samples is different.

Fig. 4. Rank abundance (mean + SE) of twenty most abundant species in each trap type at different Altitudes (500, 1,000, 1,500 and 1,800 m a.s.l.). Note that species are mostly different for each trap type, each sorted according to the rank order of the total number of individuals in that trap type. Grey columns show species that were shared between light and Malaise traps, and white columns show the shared species between pitfall, light and pitfall traps (one species). For species identities, see Appendix.

Fig. 5. NMDS plots for Malaysian beetle data, separate runs for three sampling methods (light, Malaise or pitfall traps) at different Altitudes (500 m = black squares, 1,000 m = red circles, 1,500

m = green up-triangles and 1,800 m = blue down-triangles). For statistical comparisons between Altitudes, see Table 3.

Fig. 6. MRT for sampling-method specific beetle data, using mountain (Fraser's Hill or Genting Highland) and Altitude (500, 1,000, 1,500 or 1,800 m a.s.l.) as explanatory variables. The column plots show relative abundance of each species captured using a given trap type, sorted according to the rank order of abundance of the total trap-type specific sample. Numbers in parentheses below each end branch show the number of samples falling into that branch.

Figure 1

Click here to access/download;Figure;Fig 1.jpg

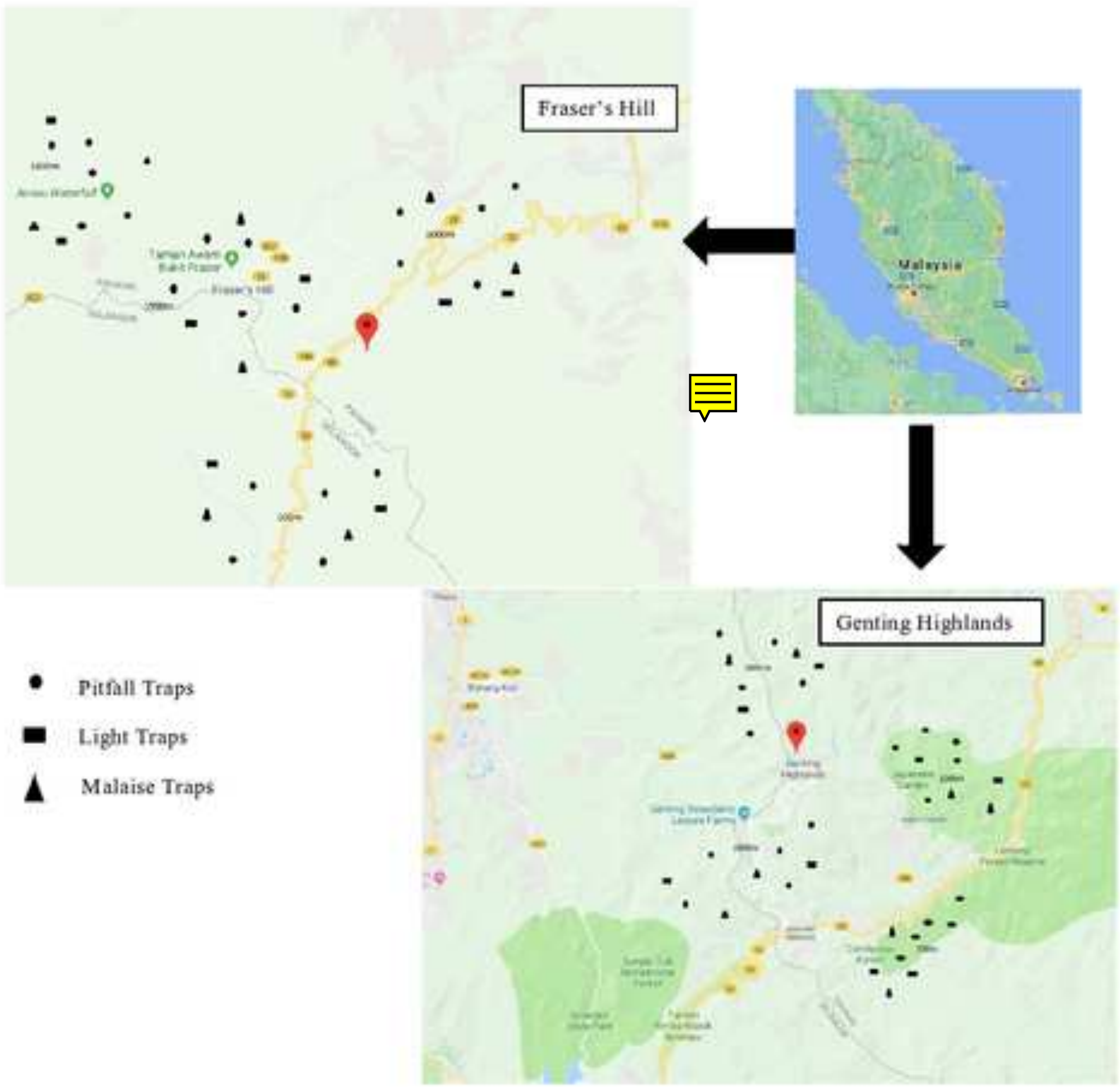

Figure 2

Click here to access/download;Figure;Fig 2.jpg

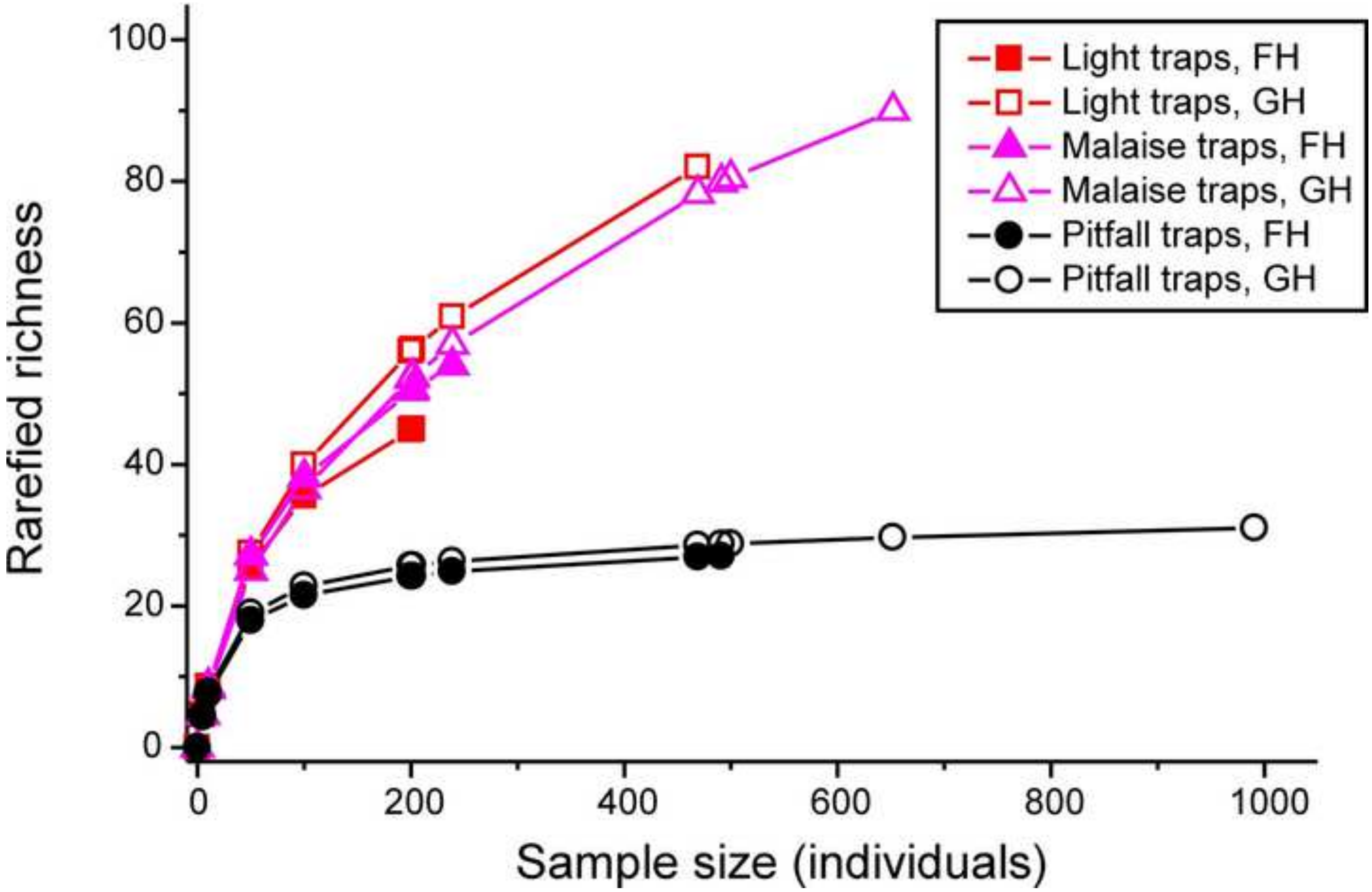

Figure 3

Click here to access/download;Figure;Fig 3.tif

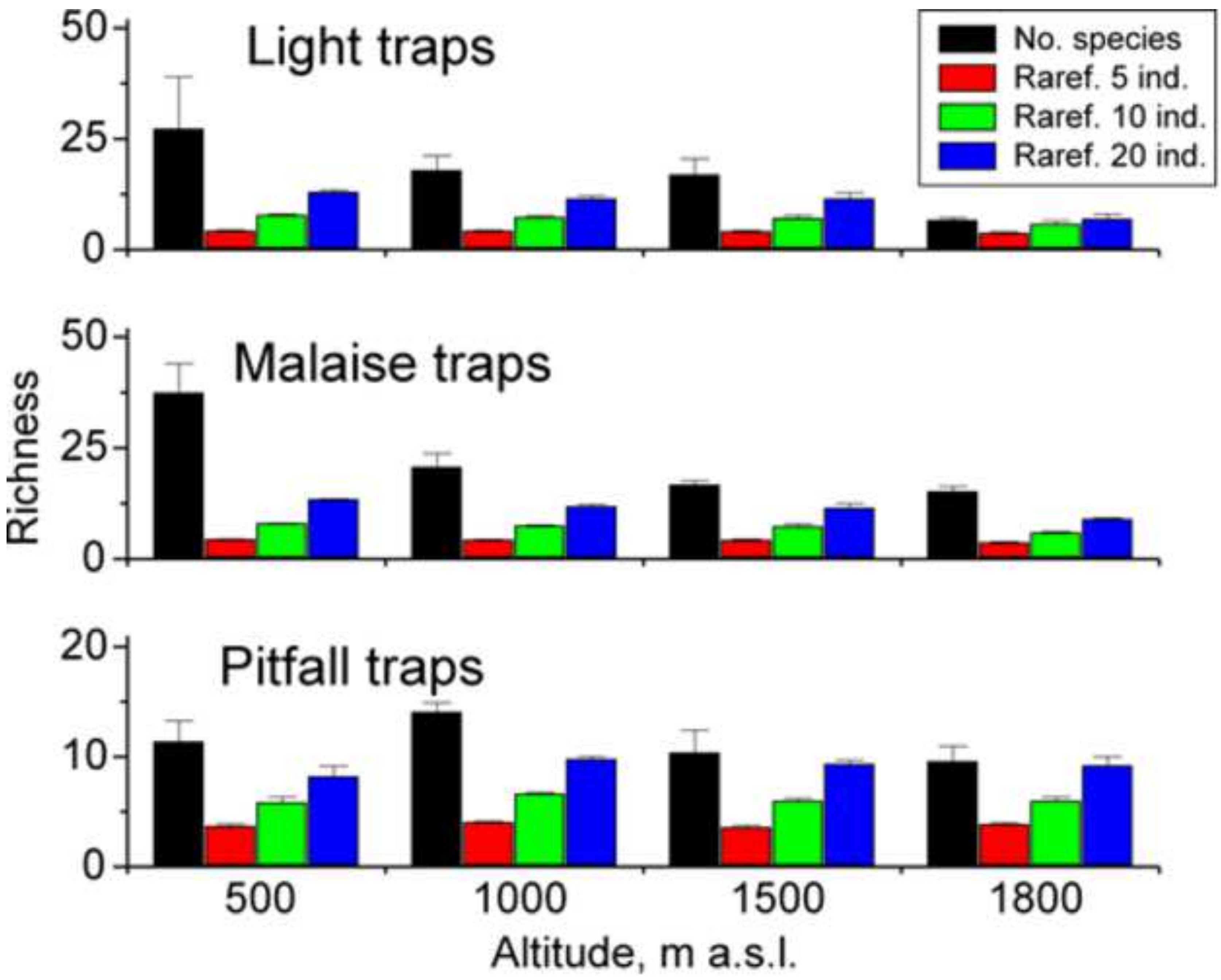

Figure 4

Click here to access/download;Figure;Fig 4.tif

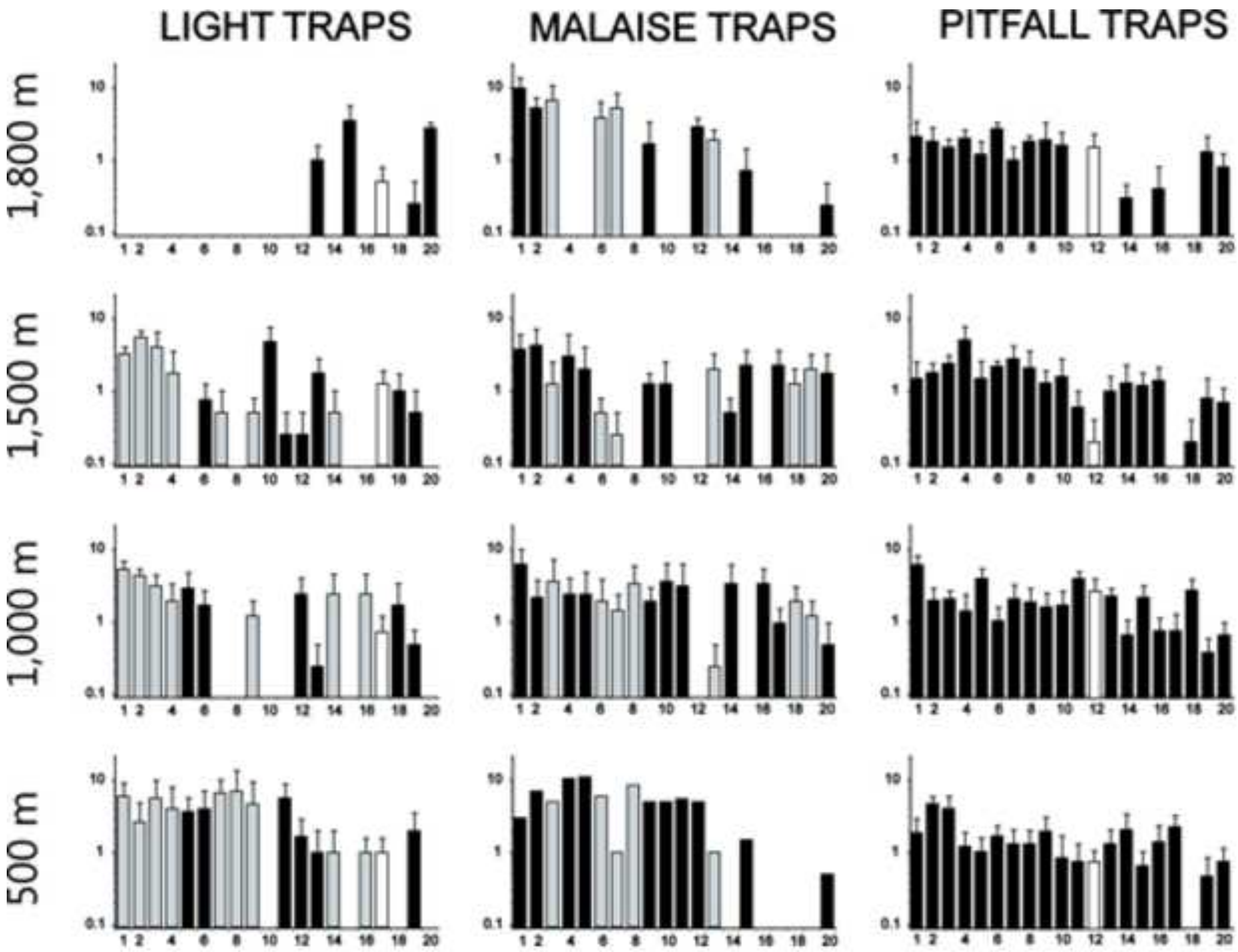

Figure 5

Click here to access/download;Figure;Fig 5.tif

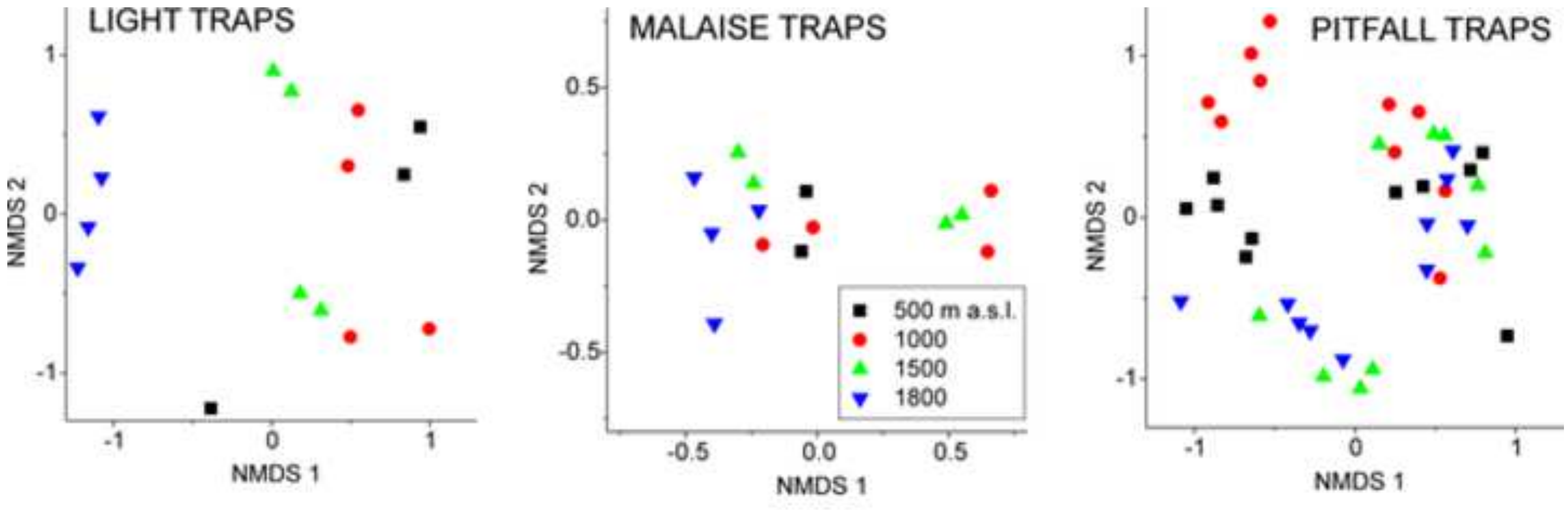

Figure 6

Click here to access/download;Figure;Fig 6.tif

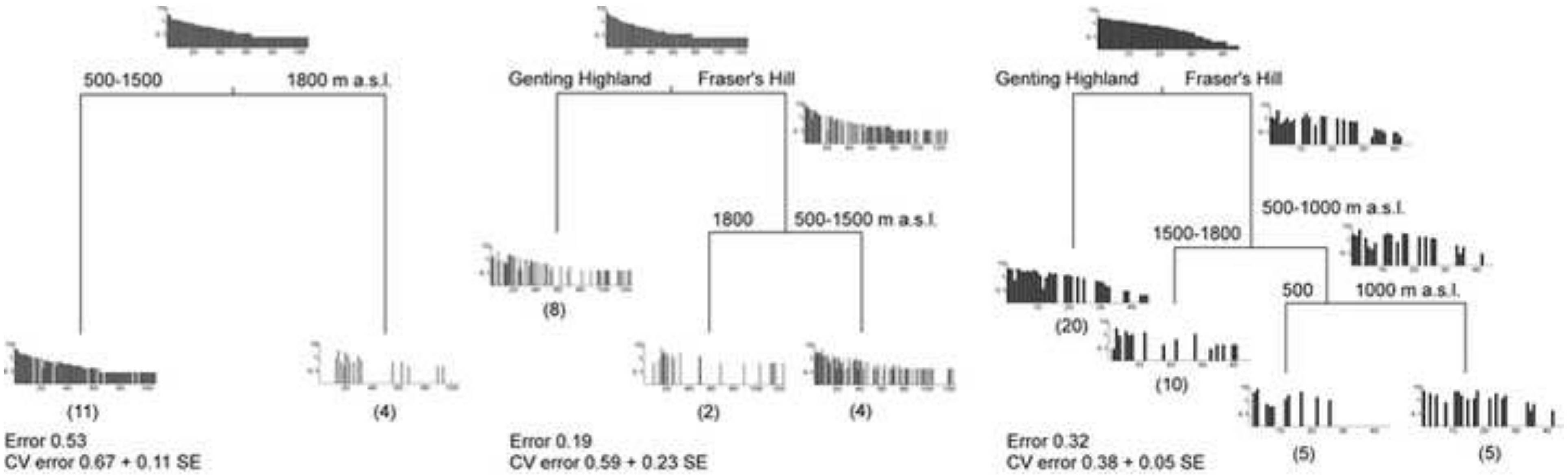

Click here to access/download
Supporting Information
PLOS ONE R1 Online Supplementary Materials.docx

Beetle Data

Click here to access/download

Supporting Information

Supporting Information Musthafa et al..xlsx

Full Title:

The functioning of different beetle (Coleoptera) sampling methods across altitudinal gradients in Peninsular Malaysia

Short Title: Beetle sampling across altitudes in Malaysia

Muneeb M. Musthafa[1,2,3 *], Fauziah Abdullah[2,3,4] and Matti J. Koivula[5]

[1]Department of Biosystems Technology, Faculty of Technology, South Eastern University of Sri Lanka, University Park, Oluvil, #32360, Sri Lanka.

[2]Institute of Biological Science, Faculty of Science, University of Malaya, 50603, Kuala Lumpur, Malaysia.

[3]B513, Toxicology Lab, Institute of Postgraduate Studies, University Malaya, 50603, Kuala Lumpur, Malaysia.

[4]Center of Biotechnology in Agriculture, University Malaya, 50603, Kuala Lumpur, Malaysia

[5] Natural Resources Institute Finland (LUKE), P. O. Box 2, FI-00791 Helsinki, Finland.

* Corresponding author: muneeb@seu.ac.lk

Author contributions

Muneeb M. Musthafa: Initial research idea, fieldwork, data curation, manuscript writing.

Fauziah Abdullah: Conceptualization of the research and methodological design, manuscript writing.

Matti J. Koivula: Statistical analysis, manuscript writing, and technical editing.

Abstract

Biodiversity research relies largely on knowledge about species responses to environmental gradients, assessed using some commonly applied sampling method. However, the consistency of detected responses using different sampling methods, and thus the generality of findings, has seldom been assessed in tropical ecosystems. H~~h~~ence, we studied the response consistency and indicator functioning of beetle assemblages ~~to~~ in altitudinal gradients ~~in~~ from two mountains in Malaysia, using Malaise, light, and pitfall traps. The data were analyzed using generalized linear mixed-effects models (GLMM), non-metric multidimensional scaling (NMDS), multivariate regression trees (MRT), and indicator species analysis (IndVal). We collected 198 morpho-species of beetles representing 32 families, with a total number of 3,052 individual beetles. We found (1) considerable differences in sample richness and composition between two mountains in pitfall but less so in light or Malaise traps; (2) light traps (but not the other trap types) distinguished high from middle or low altitudes in terms of beetle richness and assemblage composition; and (3) lower altitudes hosted about twice as many indicators as middle or high altitudes, and many species were trap-type specific in our data. These results suggest that the detection of community variation in the environment depends on the sampling method, but also on the analytical approach, suggesting the need to use multiple methods in environmental assessments. ~~These results suggest that the~~

~~detection of community variation in the environment depends on the sampling method, but also on the analytical approach.~~

Keywords: biodiversity, ~~elevation~~altitude, forest, light trap, Malaise trap, pitfall trap, tropical.

Introduction

Most hotspots for the global biodiversity can be found in tropical regions (Mittermeier et al. 2011) which are thus of central importance for conservation. Tropical species often have limited distributions, particularly those species that occupy higher altitudes at mountain slopes (Cox et al. 2016). The majority of the world's vertebrates, for instance, occupy mountain regions that cover only one-fourth of terrestrial landmass (excluding Antarctica; ~~) (~~Rahbek et al. 2019). Such mountain species are often endemic to these regions, and also sometimes such that may not be able to move elsewhere if conditions turn unfavorable for them due to, for example, habitat destruction or climate change (Escobar et al., 2005; Elsen & Tingley 2015). Hence, studies on mountain species communities provide crucial information for global conservation efforts, in addition to these environments being particularly vulnerable to habitat degradation and alterations in landscape structure (Körner 2003; Rangwala & Miller 2012).

Community composition – species, their relative shares, and interactions (biotic responses) – largely determines the general biodiversity response to environmental variation. Communities vary according to, among other aspects, latitude and altitude (Hooper et al. 2005; Ashton et al. 2015; Bouzan et al. 2015). Our understanding regarding this variation relies on some commonly assessed taxonomic groups and associated sampling methods. Similarly, land-use planning or conservation decisions may be based on only a handful of well-known species or a single sampling

method. Insects, for instance, are often sampled using light, Malaise, or pitfall traps (e.g., Grootaert et al. 2010). Environmental assessments, however, are often based on just one sampling method, which is assumed to produce a general biodiversity response, but this assumption has seldom been challenged.

Earlier research indicates that the three mentioned trap types differ in their efficiency in capturing specimens and species (e.g., Hansen 1988; Ulyshen et al. 2005; Devigne & De Biseau 2014) though the efficiency could vary with the composition of the local species community determined by, for example, altitude or habitat type. As different methods are known to capture partly different species (e.g., Missa et al. 2009), their use may, at least theoretically, result in different conclusions and hence applications in land use or conservation.

The objective of the present study is methodological: we assess the consistency of light, Malaise, and pitfall trap samples in reflecting a simple environmental gradient, altitude. More specifically, we compare the three sampling methods across the altitudinal gradient from four viewpoints:

(1) Assemblage species richness;

(2) Assemblage species-compositional turnover;

(3) Assemblage composition; and

(4) Species associated with different combinations of sampling method and altitude

Materials and Methods

Study sites

The Titiwangsa mountain range dominates the landscape of Peninsular Malaysia. Within this range, we sampled beetles at Fraser's Hill (3°43' N, 101°45' E) and Genting Highland (3°25' N, 101°47' E) that are 95 km apart (Fig. 1). Fraser's Hill mountain tops peak at between 1,000 and 1,800 m a.s.l., whereas the Genting Highland peaks at about 1,800 m a.s.l. In this region, wet and dry seasons cannot be differentiated, as the annual rainfall of 1,800–3,500 mm is distributed throughout the year (Cheong 2013; Ya'cob et al. 2016). The collection permits for Fraser's Hill and Genting Highlands were obtained from Forest Department of Malaysia.

Fraser's Hill, locally known as Bukit Fraser, is a well-preserved, permanently protected nature reserve located at the Raub district of Pahang state. Fraser's Hill has been developed as a hill station dating back in 1919 (Freeman 1999), where 90% of the 2,800-ha land area is covered by forests (Kiew 1998; Cheong 2013). Fraser's Hill comprises residential areas, commercial complexes, community services, and recreational facilities. The forests mainly consist of tropical montane cloud forests. Lower montane forests – at about 500–1,200 m a.s.l. – are dominated by Fagaceae and Lauraceae trees, whereas upper montane forests – from about 1,200 to 1,800 m a.s.l. – are dominated by Coniferae, Ericaceae, and Myrtaceae trees (Perumal & Lo 1998).

Genting Highland is perhaps the most disturbed cloud forest in the Malaysian mountains. The summit is covered by amusement parks, casinos, and hotels (Peh et al. 2011). The highest peaks at Genting Highland reach 1,800 m a.s.l, where 96% of the total of 3,965 ha of land is still covered with mostly primary forests (Cheong 2013). Before Genting Highland became an entertainment site, it was an undisturbed forest that could be reached only via jungle trekking (Piggott 1977; Stone 1981).

Sampling methods

We sampled beetles using two Malaise traps, two light traps, and 25 (5 groups of 5) pitfall traps at each altitude (500 m, 1,000 m, 1,500 m, and 1,800 m a.s.l.) at both mountain slopes. All the traps (or trap groups for pitfall traps) were set at least 150 m apart at a given altitude and were placed at least 150 m from the nearest main road. These roads and a river split the sampled forests into a total of twenty distinctive stands; each of these had at least one type of trap (Fig. 1, Table 1).

Malaise traps consisted of a nylon net connected to a collection jar, half filled with 70% ethanol and attached to a tree branch about one meter above the ground. Light traps had a mosquito net with attached 160-watt mercury bulb connected to a portable Honda EU10i generator. Pitfall traps were plastic cups (diameter 65 mm, depth 95 mm) partly filled with 70% ethanol and dug into the ground with the rim flush with the soil surface. We placed large dry leaves above each pitfall trap to protect them from litter and rain.

We sampled beetles once per month in October 2014, and March, June, and September 2015. At each collecting date, Malaise and pitfall traps were set for 24 hours, starting at 08:00 AM, and light traps operated from 18:00 to 23:30. In the latter, beetles were obtained manually from the traps using collection bottles and aspirators. We occasionally continued to use the light traps until the next morning at 06:00 AM but did not capture additional beetles.

In statistical analyses, we pooled the four periods for each trap (or trap group for pitfall traps) and consider each trap (or trap group for pitfall traps) as a replicate (initially 72; 4 altitudes and 2 mountains, each with 2 light and 2 Malaise, and 5 groups of pitfall traps).

Identification of specimens

We sorted, counted, and cross-checked all beetle specimens using available keys (Saito 2003; Mohamedsaid 2004; Naomi 2006; Schawaller 2006, 2012; Schimmel & Tarnawski 2007; Assing 2008; Hlaváč et al. 2011; Prathapan & Viraktamath 2011; Ryvkin 2011; Shi et al. 2013; Filippini et al. 2016; Anzaldo 2017; Makranczy 2017; Sasakawa et al. 2017). We confirmed the identification of difficult specimens at collections of the Wildlife Department of Malaysia, University of Malaya, National University of Malaysia, and Forestry Department of Malaysia. The identification of beetles to species is hampered by the lack of experts and species compilations. In the present study, the samples contained at least five species new to science, which will be described in later papers. Clearly, the often-broad taxonomic levels and the shortage of knowledge about the ecological traits of species must be acknowledged while interpreting results, as similar-looking species might be different in terms of, for example, life cycles, diets, abilities to disperse, and habitat requirements.

Statistical analysis

We had initially 72 samples (16 for light, 16 for Malaise, and 40 for pitfall traps). Three samples, however, produced only 1-2 species and were excluded due to difficulty in calculating pair-wise dissimilarities and richness estimates (one light and two Malaise trap samples). Thus, we ran all analyses with 69 samples (Table 1). We ran the following analyses for the four viewpoints proposed above.

Assemblage richness:

To assess sampling-method dependent variation in species, we subjected the beetle data to a generalized linear mixed-effects model (GLMM; Zuur et al. 2009) to quantify variation according

to Altitude (500, 1,000, 1,500, or 1,800 m a.s.l.). We ran the model separately for each sampling method (light, Malaise, or pitfall traps; hereafter "Method" for brevity) to assess their similarity in responses to Altitude. We subjected the observed species (number of species) and richness estimates based on rarefaction standardization to 5, 10, and 20 individuals, to a GLMM with Altitude as a fixed factor and Mountain (Fraser's Hill or Genting Highland; Fig. 1, Table 1) as a random factor (to account for possible spatial autocorrelation) and assumed a Gaussian error distribution. We checked the normality of residuals using Q-Q plots (Supplementary Figure 1). These plots did not indicate major departures from normality, except in the pitfall trap data for the number of species and rarefied richness to 5 individuals. We attempted to solve these by square-root and log transforming the data, and by applying a quasi-Poisson error distribution on untransformed data (Supplementary Figure 2). The quasi-Poisson error distribution improved the model fit for the number of species but not for the rarefied richness. Nevertheless, as the rarefied richness to 10 and 20 individuals produced broadly similar results, we are confident that our initial analyses are robust.

Assemblage turnover:

To examine possible species turnover according to Altitude and Method, we calculated averages and standard errors for 20 most abundant species, separately for each Altitude and Method. We plotted these values according to Altitude, using the rank order of the abundances of species captured using each Method. We evaluated the community turnover, or distinctiveness of samples, between Altitudes using permutational multivariate ANOVA (the adonis function in R package vegan, with Mountain [Fraser's Hill or Genting Highland] as strata; Oksanen et al. 2018). Here, we only considered single, or combinations of subsequent, Altitudes. Thus, for example, a combination 500 + 1000 m was considered but not 500 + 1800 m. As a simple measure of turnover

between Methods, we indicated in these plots species that were unique for a given Method, and those species that were shared with 1–2 other Methods. We confirmed this comparison of Methods using permutational multivariate ANOVA, as described above.

.

Assemblage composition:

To examine beetle assemblage composition across Altitudes and Methods, we used two analyses. Firstly, we used non-metric multidimensional scaling (NMDS; Borg & Groenen 2005) to assess variation in community composition. We used Method-specific data sets by applying a Bray-Curtis dissimilarity matrix. We used the above-described permutational multivariate ANOVA for Altitudes as a confirmation of the NMDS result. Secondly, we subjected the Method-specific beetle data sets to multivariate regression tree analysis (MRT; De'ath 2002) based on the Bray-Curtis dissimilarity matrix. We used Altitude and, as our earlier research has indicated differences in beetle faunas between Fraser's Hill and Genting Highland (Musthafa et al. 2018), Mountain as explanatory variables. MRT identifies groups of samples as defined by explanatory variables (Mountain and Altitude) and is not restricted by non-linearities (De'ath 2002). We present the result as a tree of dichotomies, where each dichotomy is based on minimizing the dissimilarity of samples within each tree branch. We report the tree with the lowest cross-validated relative error, following the 1-SE rule (Breiman et al. 2017). Cross-validated relative error provides a better estimate than relative error for the predictive accuracy of the MRT for a new dataset (De'ath 2002).

Species associations with Altitudes and Methods:

To detect species characteristic to particular combinations of Method and Altitude, we calculated an indicator value (IndVal; Dufrêne & Legendre 1997, De Cáceres et al. 2010) for each species,

based on all logical combinations of Method and Altitude. Here, we allowed each Method to appear singly or jointly with 1–2 other Methods, whereas for Altitude we only considered single, or combinations of subsequent, Altitudes, as described above. We restricted the IndVal to species with a total sample of at least five individuals.

Analysis software:

We ran the analyses using R 3.6.1 software (R Core Team, 2019) with lme4 (Bates et al. 2015), vegan (Oksanen et al. 2018), mvpart (Therneau et al. 2014), indicspecies (De Cáceres & Jansen 2016), and car (Fox & Weisberg 2018) packages.

Results

Beetle richness according to Mountain and Altitude

We collected 198 morpho-species of beetles representing 32 families with a total number of 3052 individual beetles (Supporting Information). Nine were identified to species, 143 to genus, and 43 to higher taxonomic levels. We refer to all these as "species" below for convenience. Regarding Method, we collected 107 species using light traps, 127 using Malaise, and 45 using pitfall traps. A total of 135 species were represented by only one Method, whereas 45 had been captured with two and 18 with all three sampling methods. Altogether 98 species were singletons or doubletons, whereas 84 were represented by at least five individuals (Appendix Table S1~~Appendix Table 1~~). Species accumulation curves based on rarefaction suggested that pitfall traps had captured nearly all potential species at about 200 individuals at both Mountains, whereas the accumulation curves for light and Malaise traps were still very steep at about 400-500 individuals (~~Appendix 1~~Fig. 2).

The GLMM performed rather similarly in terms of the four measures of richness, but the three Methods produced partly different results (Table 2, Fig. 2; Supplementary materials, Table S1). The Mountains differed little in terms of light and Malaise trap data but remarkably in pitfall-trap data. The richness measures based on light traps declined with Altitude but only 1,800 m differed significantly from 500 m. The decline with Altitude occurred also in Malaise traps so that all higher Altitudes differed significantly from 500 m. Richness measures based on pitfall traps, on the other hand, peaked at 1,000 m (Table 2, Fig. 2).

Species turnover

The rank-abundance plots of twenty most abundant species (Fig. 4~~3~~) reflected relatively high similarity between light and Malaise traps, with nine of the 20 most numerous species being shared (grey columns), whereas only one of the 20 dominant species – *Pityogenes* sp1 – in pitfall-trap samples was shared with light- and Malaise-trap samples (white columns). For species identities in this graph, see Appendix Fig. S1.~~Appendix~~. Permutational multivariate ANOVA indicated that also light and Malaise traps were compositionally different; for light vs. Malaise traps, F = 3.21, R2 = 0.11, p = 0.0010; for light vs. pitfall traps F = 9.41, R2 = 0.15, p = 0.0010; and for Malaise vs. pitfall traps F = 10.48, R2 = 0.17, p = 0.0010. Moreover, the plots suggested more abundance or occurrence changes with Altitude in Malaise and light traps, whereas the plots varied less and more erratically in pitfall-trap samples (Fig. 4~~3~~). Another striking pattern in light-trap samples (Fig. 5~~3~~, left) was the lack of the 12 most abundant species at 1,800 m. ~~The three Methods are difficult to compare due to different sampling efforts, species pools, and perhaps also capturing efficiencies, but o~~Only 9% of light-trap species and 17% of Malaise-trap species were found in both 500 m and 1,800 m, whereas the percentage was 63 for pitfall-trap samples.

Permutational multivariate ANOVA confirmed compositional changes with Altitude, particularly at 1,800 m, but the three Methods showed different patterns in this respect (Table 3). In light traps, only 1,800 m differed significantly from the other Altitudes; however, all except one combination of 2–3 Altitudes also differed significantly from the rest of the Altitudes. The patterns were similar for Malaise traps, except that different combinations of 2–3 Altitudes differed less commonly from the rest of the Altitudes. Regarding pitfall traps, then, all Altitudes and their combinations (except for 1,000–1,500 m) had distinctive assemblages (Table 3).

Assemblage composition according to Method and Altitude

According to NMDS, samples in light and Malaise traps distinguished 1,800 m from the three lower Altitudes, whereas pitfall trap samples formed two sample clusters, of which only the other distinguished clearly between Altitudes (Fig. 65). Thus, in pitfall-trap samples, Altitude was reflected in Fraser's Hill but not in Genting Highland (on the left and right, respectively, in Fig. 5).

MRT for light-trap data consistently resulted in a one-dichotomy tree (Fig. 65). The only division, where 1,800 m was split from the rest of the Altitudes, explained 47% of the variation in light-trap data, the former being species poor according to branch-specific rank-abundance plots (Fig. 65). Malaise-trap data, on the other hand, suggested that the same dichotomy, based on Altitude, was apparent in Fraser's Hill but not in Genting Highland; again, 1,800 m had fewer species though this was based on only two samples. This two-dichotomy tree explained 81% of the variation in the Malaise trap data (Fig. 65). Also, pitfall trap data suggested that Altitudes could be distinguished at Fraser's Hill but not at Genting highland; here, 1,500 and 1,800 m diverged from the two lower Altitudes that, in turn, were split in the third dichotomy (Fig. 65).

Species associated with different combinations of Method and Altitude

We found 83 indicator species for different combinations of Method and Altitude (Table 4). Thirteen species were commonly caught with two sampling methods (12 for light and Malaise traps, and one with Malaise and pitfall traps). Moreover, eight species indicated pitfall traps across the full Altitudinal range (500-1,800 m), whereas we did not detect such altitude-independent indicators for the two other sampling methods. Light traps produced 12 indicators of low (500-1,000 m), six of the middle (1,000-1,500 m) and seven of high (1,500-1,800 m) Altitudes, whereas the respective numbers were 16, four, and six for Malaise and ten, eleven and five for pitfall traps (Table 4). Moreover, three species occurred in pitfall traps across a wider Altitudinal range: two species were found at 500-1,500 m and one at 1,000-1,800 m.

Discussion

Beetle species turnover between methods and across the altitudinal gradient

We found that light and Malaise trap samples shared about half of the abundant species, whereas pitfall trap samples were distinctive in this respect. Light and Malaise traps may have attracted a shared pool of species that flies actively, whereas pitfall traps capture mostly ground dwellers that seldom fall into the two other types of traps (e.g., Missa et al. 2009). This finding suggests that, at least in the study region, the variation in beetle communities caused by ~~elevation~~ altitude or climatic factors may be better captured if pitfall traps are used together with either light or Malaise traps, whereas a combination of light and Malaise traps may not be equally efficient.

At 1,800 m light (but not Malaise or pitfall) trap samples lost all 12 most abundant species of the total light-trap sample. This loss might have resulted from the captured species pool being more

sensitive to altitude-associated changes in abiotic or biotic conditions. Studies comparing climatic conditions associated with altitudinal gradients may provide cues on species responses to the global climate change (Chen et al. 2009; Ashton et al. 2015, 2016; Musthafa et al. 2018; Musthafa & Abdullah 2019); perhaps many of the lower-altitude species eventually become more common at 1,800 m following climate change. However, some of the 12 "lost" species were still captured at 1,800 m using Malaise traps, so another explanation may be trap functioning. Perhaps light or wind conditions – or some other factors related to, for example, vegetation or moisture – were different at the highest altitudes, which might, in turn, have impacted the visibility of light traps or flight activity of many species (Mathews & Mathews 1971; McGeachie 1989; Sheikh et al. 2016; Nowinszky & Puskás 2017; Skvarla & Dowling 2017). It would be important to continue beetle monitoring at these mountain tops to see whether the altitudinal distributions of species indeed change with climate and whether the currently dominant mountain-top species persist or disappear.

The relative similarity of rank-abundance plots at different altitudes, particularly for pitfall-trap samples, may have occurred because many dominant species are adapted to a wide range of altitudes (e.g., Nunes et al. 2016) and/or associated temperature or moisture conditions (Gilchrist, 1995; Saavedra et al., 2013). Pitfall traps capture mostly ground dwellers, and field-layer vegetation or soil conditions may thus have kept conditions relatively constant across the altitudinal gradient for these species. Also, climatic factors such as temperature, rainfall, relative humidity, wind velocity, and the amount of light may not have changed uniformly with increasing altitude in our study sites, but the possible altitudinal effect (changes in temperature, atmospheric oxygen, etc.) might have been compensated by, for example, habitat type or topography (Adam & Enning 1996; Adam et al. 2011). These factors might decrease variation in micro-climate (McCain & Grytnes 2001), perhaps through the sheltering effect of forest trees (Barnes et al. 1997).

Beetle community structure across the altitudinal gradient using different methods

Our community analyses suggest that the overall beetle community varied remarkably according to altitude, but the magnitude of this response depended on geographic location (the two mountains) and captured species subset (sampling method). Thus, according to NMDS and MRT, light traps distinguished 1,800 m from lower altitudes, whereas Malaise and pitfall traps reflected primarily differences between the two mountains and only secondarily altitudinal variation. Moreover, and only at Fraser’s Hill, Malaise traps distinguished 1,800 m from the lower altitudes, and pitfall traps distinguished 500, 1,000, and 1,500-1,800 m. However, altitude might partly be a proxy of other environmental variables, such that were not assessed in this study. For instance, the lower (500 and 1,000 m) and higher (1,500 and 1,800 m) altitudes represented different forest types, the former being mixed deciduous and the latter being mixed conifer-heath-myrtle forests.

Indicator species for sampling methods and altitudes

Pitfall traps produced eight indicator species that were common at all altitudes, whereas the other sampling methods did not produce such altitude generalists (for identities of these species, see Table 4). This finding is in line with our rank-abundance plots (Fig. 4). Moreover, all sampling methods produced at least some indicators of low (500–1,000 m), intermediate (1,000–1,500 m), and/or high altitudes (1,500–1,800 m), suggesting potential for each method to reflect altitude and thus potentially global changes in climate or land use. Important from a climate perspective, we found nine significant indicators of 1,800 m (four using light, four using Malaise, and one using pitfall traps). Species adapted to these highest mountain tops may face difficulty in survival with

predicted climate warming (e.g., Lenoir et al. 2008; Pickering et al. 2008; Dirnböck et al. 2011; Elsen et al., 2020) unless they can adapt or disperse elsewhere. Good dispersal ability of beetles allows them to rapidly move towards higher altitudes with warmer temperature (Bale et al., 2002; Williams & Liebhold, 2002). Moreover, beetles may lengthen their flight-activity period and increase the number of generations produced per year to compensate with changing climate, most notably temperature (Menéndez, 2007; Altermatt, 2010). Moreover, thermal adaptability of species could regulate the distributions and local adaptations of beetles across altitudinal gradients, since temperature intensely influences their flight activity (Calosi et al., 2010; Sunday et al., 2012; García-Robledo et al., 2016). For most Malaysian beetle species, the level of knowledge about such traits is low. Though *Onthophagus* beetles (Coleoptera: Scarabaeidae) in South Korea have been predicted to move to higher altitudes with increasing ambient temperature (Kwon et al. 2015). European studies suggest that the dispersal ability is high for Curculionidae (Bouget et al., 2015) and dung beetles (Gómez et al., 2020). Moreover, some of the species in the present data can be categorized as specialists of narrow temperature ranges, such as *Orphnebius* species (Staphylinidae), and *Rhadinomerus* species (Curculionidae), whereas *Brachypeplus* species may be considered a generalist in terms of temperature conditions of the studied mountain slopes (Musthafa, 2018). On the other hand, *Inopeplus* (Salpingidae), *Macrotomoderus* (Anthicidae), *Paederinae* (Staphylinidae) and *Pentagonica* species (Carabidae) are considered temperature specialists and only *Stenus* species (Staphylinidae) can be considered as a temperature-range generalist (Musthafa, 2018).

~~For most Malaysian beetle species, the level of knowledge about such traits is low.~~ The precautionary principle ~~nevertheless~~ calls for mitigation of climatic or land-use alterations at high altitudes (Gallou et al. 2017). In our study area, for instance, the two mountains hosted very

different beetle communities, which perhaps partly reflects different intensities of land use. The studied sampling methods probably differ in required labor, relative capturing efficiency, and captured species pool. The differences, reported here, nevertheless suggest that the use of more than one location and several sampling methods ~~is~~ are desirable in environmental assessments (Lamarre et al. 2012; Devigne & De Biseau 2014; Abdullah & Musthafa 2019).

Research caveats

Taxonomic constraints may have affected our results to some extent, as most taxa had been identified to family or genus levels only. Thus, two individuals of a given taxon being captured using two methods or at different altitudes might in reality be two different species. However, this possibility rather masks than exaggerates the true community responses. We are therefore confident that the sampling methods and altitudes truly differed in beetle communities, but the differences would have been more pronounced had we been able to identify everything to species.

~~Beetle community assemblages across the elevational gradient at both mountains differed significantly, a finding similar to other studies from different parts of the world (Escobar et al. 2005; Wardhaugh et al. 2018; Hiramatsu & Usio 2018; Şenyüz et al. 2019). One explanation for this difference is land use. We had had one near-pristine (Fraser's Hill) and one partly managed mountain (Genting Highland) to study, which is too little to assess a general human impact, warranting further research. Nevertheless, Gallou et al. (2017) showed for butterflies, and Assis et al. (2018) and Flores et al. (2018) for ants, that altitude, habitat features, and human land-use all shape the community structure.~~

Conclusions

The location of the present study, Malaysia, belongs to the global biodiversity hotspot of Sundaland, yet little is known about the invertebrate diversity of its mountains (Myers et al. 2000, Doll et al. 2014). Our data contribute to fulfilling this knowledge gap. However, our results are also applicable to tropical species conservation: they provide evidence for adaptations of many species to particular altitudes and, more importantly, differences in beetle samples between collecting methods. The three sampling methods reflected the altitudinal gradient in different ways. This, in turn, calls for caution in interpretations of data based on only one method and thus on a narrow range of species life histories. We, therefore, recommend the use of multiple collecting methods in environmental impact assessments on biodiversity, whenever possible. This is particularly important in land-use or political decision making, which should ideally be based on a holistic picture of biodiversity to avoid unwanted species losses or changes in ecosystem functioning.

Acknowledgments

We thank Mohd Shukri Mohd Sabri and Davindram A/L Rajendram for assistance in beetle sampling. We also acknowledge the Universiti Kebangsaan Malaysia and Forestry Department of Malaysia for supporting this study, and Marcelo Bruno Pessôa and an anonymous reviewer for constructive comments.

References

Abdullah F, Musthafa MM. Beetle abundance, diversity and species richness of Pulau Pangkor, Perak, Malaysia. Malaysia Forester 2019; 82: 247-270.

Adam JH, Enning FL. Altitudinal zonation of tropical rain forest at Danum Valley Research Centre, Sabah, Malaysia. Malaysian J Sci 1996; 17A: 25-35.

Adam JH, Ming LK, Juhari MAA, Jaluluddin MA, Idris WMR, Othman AR, Tarmiddzi SNA. Cluster Analysis of Submontane Forest along Western Slope of Frasers' Hill Research Centre in Raub District, Malaysia. Bangladesh J Bot 2011; 40: 121-132.

Altermatt F. Climatic warming increases voltinism in European butterflies and moths. Proc Biol Sci. 2010; 277(1685):1281-1287. doi:10.1098/rspb.2009.1910.

Anzaldo SS. Review of the genera of Conoderinae (Coleoptera, Curculionidae) from North America, Central America, and the Caribbean. ZooKeys 2017; 683: 51-138. https://doi.org/10.3897/zookeys.683.12080.

Ashton LA, Nakamura A, Basset Y, Burwell CJ, Cao M, Eastwood R, et al. Vertical stratification of moths across elevation and latitude. J Biogeogr 2016; 43: 59-69. https://doi.org/10.1111/jbi.12616

Ashton LA, Odell EH, Burwell CJ, Maunsell SC, Nakamura A, McDonald WJF, Kitching RL. Altitudinal patterns of moth diversity in tropical and subtropical Australian rainforests. *Austral Ecol* 2015; 41: 197-208. https://doi.org/10.1111/aec.12309.

Assing V. Four new species and additional records of Palaearctic *Sunius*, with two new synonymies (Coleoptera: Staphylinidae: Paederinae). *Beiträge zur èntomologie* 2008; 58: 455-470. https://doi.org/10.21248/contrib.entomol.58.2.455-470.

Bale JS, Masters GJ, Hodkinson ID, Caroline A, Martijn BT, Valerie KB, … John BW. Herbivory in global climate change research: Direct effects of rising temperature on insect herbivores. Global Change Biology 2002; 8: 1-16. https://doi.org/10.1046/j.1365-2486.2002.00451.x

Baniya CB, Solhøy T, Gauslaa Y, Palmer MW. The elevation gradient of lichen species richness in Nepal. The Lichenologist 2010; 42: 83-96. https://doi.org/10.1017/S0024282909008627

Barnes BV, Zak DR, Denton SR, Spurr SH. Forest ecology. 4th ed. John Wiley & Sons. 1999.

Barzani GM, Sahibin AR, Adam JH, Zulfahmi AR. The physical environment. Bukit Fraser crown of the Titiwangsa Range. In: Latiff, A., Maimon, A., Norhayati, A. & Adam, J.H., editors. Bukit Fraser Research Centre, Universiti Kebangsaan Malaysia & Fraser's Hill Development Corporation, Fraser's Hill Development Corporation, Raub, Malaysia; 2009. pp 9-16.

Bouget, C., Brin, A., Tellez, D. et al. Intraspecific variations in dispersal ability of saproxylic beetles in fragmented forest patches. Oecologia 2015; 177: 911–920. https://doi.org/10.1007/s00442-014-3162-9

Bouzan AM, Flinte V, Macedo MV, Monteiro RF. Elevation and temporal distributions of Chrysomelidae in southeast Brazil with emphasis on the Galerucinae. ZooKeys 2015; 547: 103-117. https://doi.org/10.3897/zookeys.547.9723

Brehm G, Colwell RK. Kluge J. () The role of environment and mid-domain effect on moth species richness along a tropical elevational gradient. Glob Ecol Biogeogr 2007; 16: 205-219. https://doi.org/10.1111/j.1466-8238.2006.00281.x

Bates D, Maechler M, Bolker B, Walker S. Fitting linear mixed effects models using lme4. J Stat Softw 2015; 67: 1-48. http://dx.doi.org/10.18637/jss.v067.i01

Borg I, Groenen P. Modern multidimensional scaling: theory and applications. 2nd ed. Springer-Verlag, New York. 2005

Breiman L, Friedman JH, Olshen RA, Stone CG. Classification and regression trees. CRC Press, Boca Raton, Florida. 358 pp. 1998.

Calosi P, Bilton DT, Spicer JI, Votier SC, Atfield A. What determines a species' geographical range? Thermal biology and latitudinal range size relationships in European diving beetles (Coleoptera: Dytiscidae). J Anim Ecol 2010; 79: 194–204. DOI: 10.1111/j.1365-2656.2009.01611.x.

Chao A, Jost, L. Coverage-based rarefaction and extrapolation: standardizing samples by completeness rather than size. Ecol 2012; 93(12): 2533–2547.

Chen IC, Shiu HJ, Benedick S, Holloway JD, Chey VK, Barlow HS, Hill JK, Thomas CD. Elevation increases in moth assemblages over 42 years on a tropical mountain. PNAS 2009; 106: 1479-1483. https://doi.org/10.1073/pnas.0809320106

Cheong C. Fraser's Hill - A Lush Highland Hideaway. WWF Malaysia, Petaling Jaya. 2013.

Cox CB, Moore PD, Ladle R. Biogeography: an ecological and evolutionary approach. Wiley Blackwell. 2016.

De Cáceres M, Legendre P, Moretti M. Improving indicator species analysis by combining groups of sites. Oikos 2010; 119: 1674-1684. DOI: 10.1111/j.1600-0706.2010.18334.x

De Cáceres M, Jansen F. Package Indicspecies. 2016. URL https://CRAN.R-project.org/package=indicspecies.

De'ath G. Multivariate regression trees: a new technique for modeling species–environment relationships. Ecol 2002; 83(4): 1105-1117. https://doi.org/10.2307/3071917

De'ath G, Therneau TM, Atkinson B, Ripley B, Oksanen J. mvpart: Multivariate partitioning. 2014. URL https://mran.microsoft.com/snapshot/2014-12-11/web/packages/mvpart/index.html

Devigne C, De Biseau J-C. Urban ecology: comparison of the effectiveness of five traps commonly used to study the biodiversity of flying insects. Biodivers J 2014; 5: 165-174.

Doll HM, Butod E, Harrison RD, Fletcher C, Kassim AR, Ibrahim S, Potts MD. Environmental and geographic factors driving dung beetle (Coleoptera: Scarabaeidae: Scarabaeinae) diversity in the dipterocarp forests of Peninsular Malaysia. Raffles Bull Zool 2014; 62: 549-560.

Dufrêne M, Legendre P. Species assemblages and indicator species: the need for a flexible asymmetrical approach. Ecol Monogr 1997; 67(3): 345-366. https://doi.org/10.1890/0012-9615(1997)067[0345:SAAIST]2.0.CO;2

Dirnböck T, Essl F, Rabitsch W. Disproportional risk for habitat loss of high-altitude endemic species under climate change. Glob Chang Biol 2011; 17: 990-996. https://doi.org/10.1111/j.1365-2486.2010.02266.x

Elsen PR, Tingley MW. Global mountain topography and the fate of montane species under climate change. Nat Clim Chang 2015; 5: 772-776. https://doi.org/10.1038/nclimate2656

Elsen, P.R., Monahan, W.B. & Merenlender, A.M. Topography and human pressure in mountain ranges alter expected species responses to climate change. Nat Commun 2020; 11: 1974. https://doi.org/10.1038/s41467-020-15881-x

Escobar F, Lobo JM, Halffter G. Altitudinal variation of dung beetle assemblages in the Colombian Andes. Glob Ecol Biogeogr 2005; 14: 337-347. https://doi.org/10.1111/j.1466-822X.2005.00161.x

Filippini V, Micó E, Galante E. Checklist and identification key of Anomalini (Coleoptera, Scarabaeidae, Rutelinae) of Costa Rica. ZooKeys 2016; 621: 63-136. https://doi.org/10.3897/zookeys.621.7565

Fox J, Weisberg S. An R companion to applied regression. 3rd edition. Sage Publications Inc., California. 2019.

Gallou A, Baillet Y, Ficetola GF, Despres L. Elevational gradient and human effects on butterfly species richness in the French Alps. Ecol Evol 2017; 7(11): 1-10. https://doi.org/10.1002/ece3.2803.

García-Robledo C, Kuprewicz EK, Staines CL, Erwin TL, Kress WJ. Limited tolerance by insects to high temperatures across tropical elevational gradients and the implications of global warming for extinction. PNAS 2016: 113; 680-685. doi: 10.1073/pnas.1507681113

Gilchrist GW. Specialists and generalists in changing environments. I. Fitness landscapes of thermal sensitivity. Am Nat 1995; 146: 252-270.

Gómez VCG, Verdú JR, Zurita GA. Thermal niche helps to explain the ability of dung beetles to exploit disturbed habitats. Sci Rep 2020; 10: 13364. https://doi.org/10.1038/s41598-020-70284-8

Hlaváč P, Newton AF, Maruyama M. World catalogue of the species of the tribe Lomechusini (Staphylinidae: Aleocharinae). Zootaxa 2011; 3075: 1-151.

Hooper DU, Chapin FS III, Ewel JJ, Hector A, Inchausti P, et al. Effects of biodiversity on ecosystem functioning: a consensus of current knowledge. Ecol Monogr 2005; 75: 3-35. https://doi.org/10.1890/04-0922.

Kessler M. Elevational gradients in species richness and endemism of selected plant groups in the central Bolivian Andes. Plant Ecol 2000; 149: 181-193.

Kiew R. The seed plant flora of Fraser's Hill, Peninsular Malaysia: With special reference to its conservation status. Research pamphlet No. 21. Forest Research Institute of Malaysia (FRIM), Kuala Lumpur, Malaysia. 1998.

Kohler T, Wehrli A, Jurek M. Mountains and climate change: A global concern. Sustainable Mountain Development Series. Bern, Switzerland, Centre for Development and Environment (CDE), Swiss Agency for Development and Cooperation (SDC) and Geographica Bernensia. 2014.

Koivula MJ, Chmaberlain DE, et al. Breeding bird species diversity across gradients of land use from forest to agriculture in Europe. Ecogeg 2018; 41: 1331-1344. https://doi.org/10.1111/ecog.03295

Körner C. Alpine plant life. Functional plant ecology of high mountain ecosystems. 2nd edition. Springer, Berlin. 2003.

Kumar A, Longino JT, Colwell RK, O'Donnell S. Elevational patterns of diversity and abundance of eusocial paper wasps (Vespidae) in Costa Rica. Biotropica 2009; 41, 338-346. https://doi.org/10.1111/j.1744-7429.2008.00483.x.

Kwon T-S, Lee CM, Kim S-S. Prediction of abundance of beetles according to climate warming in South Korea. J Asia Pac Biodivers 2015; 8: 7-30. https://doi.org/10.1016/j.japb.2015.01.001.

Laliberte E, Wells JA, DeClerck,F… Mayfield, MM. Land-use intensification reduces functional redundancy and response diversity in plant communities. Ecol Lett 2009; 13: 76-86. https://doi.org/10.1111/j.1461-0248.2009.01403.x

Lamarre GPA, Molto Q, Fine PVA, Baraloto C. A comparison of two common flight interception traps to survey tropical arthropods. ZooKeys 2012; 216: 43-55. https://doi.org/10.3897/zookeys.216.3332

Lenoir J, Gegout JC, Marquet PA, de Ruffray P, Brisse H. A significant upward shift in plant species optimum elevation during the 20th century. Science 2008; 320: 1768-1771. DOI: 10.1126/science.1156831.

Makranczy G. Review of the *Anotylus* cimicoides species group (Coleoptera: Staphylinidae: Oxytelinae). Acta Zoolog Academiae Scientiarum Hung 2017; 63: 143-262. DOI: 10.17109/AZH.63.2.143.2017

Mathews RW., Mathews JR. The Malaise Trap: Its Utility and Potential for Sampling Insect Populations. *The Great Lakes Entomologist* 1971; 4(4): Article 4.

McCain CM, Grytnes JA. Elevation gradients in species richness. In Encyclopedia of Life Sciences (ELS). John Wiley & Sons Ltd., Chichester. 2000.

McGeachie WJ.. The effects of moonlight illuminance, temperature and wind speed on light-trap catches of moths. Bull Entomol Res 1989; 79: 185-192.

Menéndez R. How are insects responding to global warming? Tijdschrift voor Entomologie 2007; 150: 355-365.

Mittermeier RA, Turner WR, Larsen FW, Brooks TM, Gascon C. Global Biodiversity Conservation: The Critical Role of Hotspots. In: Zachos, F.E. & Habel, J.C. editors, Biodiversity Hotspots. Springer-Verlag, Berlin-Heidelberg. 2011. Pp. 3-22.

Mohamedsaid MS. Catalogue of the Malaysian Chrysomelidae (Insecta: Coleoptera). Pensoft Publishers, Bulgaria. 2004.

Musthafa, MM. A comparative study of beetle diversity at different elevations at Fraser's hill and Genting Highland. Ph.D. Thesis. University of Malaya. 2018.

Musthafa MM, Abdullah F. Coleoptera of Genting Highland, Malaysia: Species richness and diversity along the elevations. Arx de Misc Zoology 2019; 17: 45-66. DOI: 10.32800/amz.2019.17.0045.

Musthafa MM, Abdullah F, Sánchez‐Reyes UJ. Comparative study of spatial patterns and ecological niches of beetles in two Malaysian mountains elevation gradients. J Insect Conserv 2018; 22(5-6): 757-769. https://doi.org/10.1007/s10841-018-0099-z

Myers N, Mittermeier RA, Mittermeier CG, da Fonseca GAB, Kent J. Biodiversity hotspots for conservation priorities. Nature 2000; 403: 853-858.

Naomi S. Taxonomic revision of the genus *Stenus* Latreille, 1797 (Coleoptera, Staphylinidae, Steninae) of Japan: Species group of S. indubius Sharp. Jpn J Syst Entomol 2006; 12: 39-120.

Newbold T, et al. Global effects of land use on local terrestrial biodiversity. Nature 2015; 520: 45-50. https://doi.org/10.1038/nature14324

Nowinszky L, Puskás J. Light-Trap Catch of Insects in Connection with Environmental Factors. In: Vonnie D.C. Shields, editors, *Biological Control of Pest and Vector Insects*, InTech Publishers. 2017. pp. 97-117.

Nunes CA, Braga RF, Figueira JEC, Neves FS, Fernandes GW. Dung beetles along a tropical altitudinal gradient: environmental filtering on taxonomic and functional diversity. PLoS ONE 2016; 11: e0157442. https://doi.org/10.1371/journal.pone.0157442

Oksanen J, Blanchet FG, Friendly M, Kindt R, Legendre P, McGlinn D, et al. Package vegan. Community Ecology Package. 2018. URL https://cran.r-project.org, https://github.com/vegandevs/vegan

Peh KS-H, Soh MCK, Sodhi NS, Laurance WF, Ong DF, Clements R. Up in the clouds: Is sustainable use of tropical montane cloud forests possible in Malaysia? Bioscience 2011; 61: 27–38. https://doi.org/10.1525/bio.2011.61.1.8

Perumal B, Lo FSJ. Recorded mountain trees of Peninsular Malaysia - a checklist. World Wildlife Fund Malaysia, Kuala Lumpur, Malaysia. 1998.

Pickering C, Hill W, Green K. Vascular plant diversity and climate change in the alpine zone of the Snowy Mountains, Australia. Biodivers Conserv 2008; 17: 1627-1644. https://doi.org/10.1007/s10531-008-9371-y

Piggott AG. The ferns of Gunung Ulu Kali. Garden Bulletin of Singapore 1977; 30: 31-43.

Prathapan KD, Viraktamath CA. A new species of *Longitarsus* Latreille, 1829 (Coleoptera, Chrysomelidae, Galerucinae) pupating inside stem aerenchyma of the hydrophyte host from the Oriental Region. ZooKeys 2011; 87: 1-10. doi: 10.3897/zookeys.87.1294

R Core Team. R: A language and environment for statistical computing. R Foundation for Statistical Computing, Vienna, Austria. 2019. URL https://www.R-project.org/

Rahbek C, Borregaard MK, Colwell RK, Dalsgaard B, Holt BG, Morueta-Holme N, ... Fjeldsa J. Humboldt's enigma: What causes global patterns of mountain biodiversity? Science 2019; 365(6458): 1108-1113. https://doi.org/10.1126/science.aax0149.

Rangwala I, Miller JR. Climate change in mountains: a review of elevation-dependent warming and its possible causes. Clim Change 2012; 114: 527-547. https://doi.org/10.1007/s10584-012-0419-3

Ryvkin AB. Contributions to the knowledge of *Stenus* (Nestus) species of the *Crassus* group (Insecta: Coleoptera: Staphylinidae: Steninae). Four new species from the Russian Far East with taxonomic notes. Balt J Coleopterol 2011; 11: 57-72.

Saavedra S, Rohr RP, Dakos V, Bascompte J. Estimating the tolerance of species to the effects of global environmental change. Nat Commun 2013; 4: 2350. https://doi.org/10.1038/ncomms3350

Saito M. A New Species of the genus *Tomoderus* (Coleoptera, Anthicidae) from the Ryukyu Islands, Southwest Japan. *Elytra* 2003; 31: 321-323.

Sasakawa K, Kim J-K, Kim J-K, Kubota K. Morphological phylogeny and biogeography of the *Pterostichus raptor* species group (Coleoptera: Carabidae) of ground beetles, endemic to the Korean Peninsula and adjacent islands. J Asia Pac Entomol 2017; 20: 7-12. https://doi.org/10.1016/j.aspen.2016.11.001

Schawaller W. Revision of the Oriental species of the genus Bradymerus Perroud, with descriptions of 29 new species (Coleoptera: Tenebrionidae). Stuttgarter Beiträge zur Naturkunde 2006; 4: 1-64.

Schawaller W. Two new species and new records of the genus *Spinolyprops* Pic, 1917 from the Oriental Region (Coleoptera, Tenebrionidae, Lupropini). ZooKeys 2012; 243: 83-94. DOI: 10.3897/zookeys.243.3879

Schimmel R, Tarnawski D. The species of the genus *Mulsanteus* Gozis, 1875 of Southeast Asia (Insecta: Coleoptera: Elateridae). Genus 2007; 14, 89-172.

Sheikh AH, Thomas M, Bhandari R, Bunkar K. Light trap and insect sampling: an overview. Int J Curr Res 2016; 8(11): 40868-40873.

Shi H, Zhou H, Liang H. Taxonomic synopsis of the subtribe *Physoderina* (Coleoptera, Carabidae, Lebiini), with species revisions of eight genera. Zookeys 2013; 284: 1-129. https://doi.org/10.3897/zookeys.284.3983

Skvarla MJ, Dowling APG. A comparison of trapping techniques (Coleoptera: Carabidae, Buprestidae, Cerambycidae, and Curculionoidea excluding Scolytinae). J Insect Sci 2017; 17(1): 7-20. https://doi.org/10.1093/jisesa/iew098

Staunton KM, Nakamura A, Burwell CJ, Robson SKA, Williams SE. Elevational distribution of flightless ground beetles in the tropical rainforests of North-Eastern Australia. PLoS ONE 2016; 11: e0155826. https://doi.org/10.1371/journal.pone.0155826

Stone BC. The summit flora of Gunung Ulu Kali (Pahang, Malaysia). Federation of Museum Journal 1981; 26(1): 157-162.

Sunday, JM, Bates AE, Dulvy NK. Thermal tolerance and the global redistribution of animals. Nature Clim Change 2002: 2(9); 686–690. https://doi.org/10.1038/nclimate1539.

~~Wardhaugh CW, Stone MJ, Stork NE. Seasonal variation in a diverse beetle assemblage along two elevational gradients in the Australian Wet Tropics. Sci Rep 2018; 8: 8559.~~ Williams DW, Liebhold AM. Climate change and the outbreak of two North American bark beetles. Agr Forest Entomol 2002; 4: 87-99.

Ya’cob Z, Takaoka H, Pramual P, Low VL, Sofian-Azirun M. Breeding habitat preference of preimaginal black flies (Diptera: Simuliidae) in Peninsular Malaysia. Acta Tropica 2016; 153: 57-63. doi: 10.1016/j.actatropica.2015.10.007.

Zuur AF, Ieno EN, Walker NJ, Saveliev AA, Smith GM. Mixed effects models and extensions in ecology with R. Springer Science. 2009.

Table 1 Sampling effort at Fraser's Hill (FH) and Genting Highland (GH) mountains. In both mountains, traps were set at four Altitudes: 500, 1,000, 1,500 and 1,800 m a.s.l., in ten stands in both mountains; stands are indicated with letters A-J (compare Fig. 1). The three right-hand columns show the number of traps (or groups of five pitfall traps) in each stand.

**Mountain**	**Altitude**	**Stand ID**	**Light**	**Malaise**	**Pitfall**
FH	500	A	-	-	1
FH	500	B	-	-	4
FH	500	C	1	-	-
FH	1,000	D	1	2	5
FH	1,000	E	1	-	-
FH	1,500	F	1	-	2
FH	1,500	G	-	1	1
FH	1,500	H	1	1	2
FH	1,800	I	1	2	1
FH	1,800	J	1	-	4
GH	500	A	-	1	-
GH	500	B	-	1	-
GH	500	C	2	-	5
GH	1,000	D	-	-	2
GH	1,000	E	2	2	3
GH	1,500	F	-	2	4
GH	1,500	G	-	-	1
GH	1,500	H	2	-	-
GH	1,800	I	2	1	4
GH	1,800	J	-	1	1

Table 2 GLMM summary for Altitude, using Mountain as a random factor, for the number of species and rarefaction standardized number of species for 5, 10 or 20 individuals. Results are shown for light, Malaise and pitfall trap data. Numbers in "%var" columns are percentages explained by a given variable; letters in "Effect" columns show whether a given Altitude differed significantly ($p < 0.05$) and positively (pos) or negatively (neg) from the lowest Altitude (500 m a.s.l.) (in parentheses if marginally significant, i.e., $p < 0.1$) or whether this comparison was non-significant (ns; $p > 0.1$). For full output, see Appendix, Table 1~~2~~.

	**LIGHT TRAPS**			**MALAISE TRAPS**			**PITFALL TRAPS**	
**Variable/Category**	**%var**	**Effect**		**%var**	**Effect**		**%var**	**Effect**
Number of species								
Mountain	7			4			53	
Altitude	41			78			11	
* 1,000 m a.s.l.		ns			neg			(pos)
* 1,500 m a.s.l.		ns			neg			ns
* 1,800 m a.s.l.		neg			neg			ns
Residuals	52			19			35	
Rarefied richness to 5 individuals								
Mountain	7			6			17	
Altitude	33			70			16	
* 1,000 m a.s.l.		ns			ns			pos
* 1,500 m a.s.l.		ns			ns			ns
* 1,800 m a.s.l.		(neg)			neg			ns
Residuals	60			24			67	
Rarefied richness to 10 individuals								
Mountain	15			7			26	
Altitude	45			72			11	
* 1,000 m a.s.l.		ns			ns			pos
* 1,500 m a.s.l.		ns			ns			ns
* 1,800 m a.s.l.		neg			neg			ns
Residuals	40			21			63	
Rarefied richness to 20 individuals								
Mountain	4			7			28	
Altitude	61			72			12	
* 1,000 m a.s.l.		ns			(neg)			pos
* 1,500 m a.s.l.		ns			neg			ns
* 1,800 m a.s.l.		neg			neg			ns
Residuals	35			21			59	

Table 3 Permutational multivariate ANOVA to assess the distinctiveness of beetle communities at different Altitudes (Alt 500, 1,000, 1,500 or 1,800 m a.s.l., or their logical combinations) as reflected by using three sampling methods (compare Fig. 2).

	LIGHT TRAPS							MALAISE							PITFALL					
**Variable**	**df**	**SS**	**MS**	**F**	**R^2**	**p**		**df**	**SS**	**MS**	**F**	**R^2**	**p**		**df**	**SS**	**MS**	**F**	**R^2**	**p**
Alt 500	1	0.47	0.47	1.28	0.09	0.0659		1	0.40	0.40	1.17	0.09	0.2717		1	0.48	0.48	1.58	0.04	0.0300
Residuals	13	4.75	0.37	0.91				12	4.12	0.34	0.91				38	11.55	0.30	0.96		
Alt 1000	1	0.47	0.47	1.29	0.09	0.1548		1	0.42	0.42	1.23	0.09	0.1528		1	0.97	0.97	3.34	0.08	0.0010
Residuals	13	4.75	0.37	0.91				12	4.10	0.34	0.91				38	11.06	0.29	0.92		
Alt 1500	1	0.53	0.53	1.47	0.10	0.0939		1	0.42	0.42	1.24	0.09	0.1299		1	0.46	0.46	1.51	0.04	0.0390
Residuals	13	4.69	0.36	0.90				12	4.10	0.34	0.91				38	11.57	0.30	0.96		
Total	14	5.22	1.00					13	4.52	1.00					39	12.03	1.00			
Alt 1800	1	1.36	1.36	4.56	0.26	0.0020		1	0.70	0.70	2.21	0.16	0.0030		1	0.76	0.76	2.56	0.06	0.0020
Residuals	13	3.87	0.30	0.74				12	3.82	0.32	0.84				38	11.27	0.30	0.94		
Alt 500-1000	1	0.67	0.67	1.93	0.13	0.0290		1	0.47	0.47	1.40	0.10	0.0699		1	0.97	0.97	3.33	0.08	0.0010
Residuals	13	4.55	0.35	0.87				12	4.05	0.34	0.90				38	11.06	0.29	0.92		
Alt 1000-1500	1	0.98	0.98	3.01	0.19	0.0010		1	0.68	0.68	2.13	0.15	0.0420		1	0.55	0.55	1.82	0.05	0.0070
Residuals	13	4.24	0.33	0.81				12	3.84	0.32	0.85				38	11.48	0.30	0.95		
Alt 1500-1800	1	0.67	0.67	1.93	0.13	0.0380		1	0.47	0.47	1.40	0.10	0.0619		1	0.97	0.97	3.33	0.08	0.0010
Residuals	13	4.55	0.35	0.87				12	4.05	0.34	0.90				38	11.06	0.29	0.92		
Alt 500-1500	1	1.36	1.36	4.56	0.26	0.0020		1	0.70	0.70	2.21	0.16	0.0050		1	0.76	0.76	2.56	0.06	0.0020
Residuals	13	3.87	0.30	0.74				12	3.82	0.32	0.84				38	11.27	0.30	0.94		
Alt 1000-1800	1	0.47	0.47	1.28	0.09	0.0699		1	0.40	0.40	1.17	0.09	0.2408		1	0.48	0.48	1.58	0.04	0.0210
Residuals	13	4.75	0.37	0.91				12	4.12	0.34	0.91				38	11.55	0.30	0.96		

Table 4 Significant ($p < 0.05$) indicators with $n > 4$ in the beetle data. LT = indicator of light traps; MA = indicator of Malaise traps; PT = indicator of pitfall traps; different Altitudes or ranges are shown with numbers (500, 1,000, 1,500, 1,800 m a. s. l.). Column IndVal shows indicator value for each taxon.

Category	Species	IndVal		Category	Species	IndVal
**Single-method indicators**				**Single-method indicators continued**		
LT 500	*Anomala* sp2	0.81		PT	*Harpalus* sp1	0.84
LT 500	*Anomala* sp4	0.76		PT	*Harpalus* sp2	0.91
LT 500	*Anomala* sp6	0.57		PT	*Hiletus* sp1	0.44
LT 500	*Apogonia* sp3	0.79		PT	*Inopeplus* sp1	0.63
LT 500	*Apogonia* sp5	0.79		PT	*Lebia* sp1	0.57
LT 500	*Cicindela* sp2	0.79		PT	*Omonadus* sp1	0.52
LT 500	Mordellidae A	0.80		PT	*Pentagonica* sp1	0.54
LT 500	Scarabeidae M	0.80		PT	Staphylinidae M	0.63
LT 500	Scarabeidae P	0.58		PT 500	*Pterostichus* sp2	0.87
LT 500-1000	*Epepeotes lateralis*	0.52		PT 500-1500	*Spinolyprops* A	0.53
LT 1000	*Apogonia* sp1	0.90		PT 500-1500	Staphylinidae C	0.61
LT 1000	*Cicindela* sp1	0.69		PT 1000	*Actiastes* sp1	0.71
LT 1000-1500	*Anomala* sp1	0.94		PT 1000	*Anotylus* sp2	0.86
LT 1000-1500	*Apogonia* sp2	0.96		PT 1000	*Bledius* sp1	0.78
LT 1500	Lampyridae E	0.70		PT 1000	*Lispinus* sp1	0.84
LT 1500	*Luciola* sp1	0.71		PT 1000	*Orphnebius* sp1	0.84
LT 1500-1800	*Altica* sp1	0.69		PT 1000	*Orphnebius* sp2	0.92
LT 1800	*Cleorina* sp	0.71		PT 1000	Passalidae A	0.44
LT 1800	*Hoplocerambyx spinicornis*	0.87		PT 1000	*Pityogenes* sp1	0.74
LT 1800	*Hydrovatus enigmaticus*	0.97		PT 1000	*Sunius* sp1	0.71
LT 1800	*Illeis* sp2	0.70		PT 1000-1800	*Oxylatus* sp1	0.57
MA 500	Aleocharinae sp1	0.84		PT 1500	*Hiletus* sp2	0.58
MA 500	*Anotylus* sp1	0.94		PT 1500	*Pterostichus* sp1	0.79
MA 500	*Aphthona* sp1	0.98		PT 1500-1800	*Paedarus* sp1	0.69
MA 500	*Brachypeplus* sp1	0.92		PT 1800	*Lebia* sp2	0.57
MA 500	*Brachypeplus* sp3	0.98		PT 1800	*Pterostichus* sp3	0.79
MA 500	*Bradymerus* sp2	0.99				
MA 500	*Epuraea* sp1	0.70		**Multiple-method indicators**		
MA 500	Galerucinae sp1	0.97		MA+LT 500	*Mulsanteus* sp1	0.60
MA 500	*Ischnosoma* sp1	0.95		MA+LT 500	Paederinae sp3	0.68
MA 500	*Lymantor* sp1	0.94		MA+LT 1000	*Sarmydus* sp1	0.71

MA 500	*Lymantor* sp2	0.91		MA+LT 1000-1500	*Strotocera* sp1	0.71
MA 500	*Lymantor* sp3	0.94		MA+LT 1500	Alticinae sp2	0.69
MA 500	*Sinoxylon* sp1	0.99		MA+LT 1500	*Anisandrus* sp1	0.68
MA 500	*Xyleborus* sp1	0.98		MA+LT 1500	*Brachypeplus* sp2	0.65
MA 500-1000	Aleocharinae sp2	0.58		MA+LT 1500	*Colaspoma* sp2	0.71
MA 1000	*Anomala* sp3	0.82		MA+LT 1500	Curculionidae A	0.70
MA 1000-1500	Alticinae sp1	0.70		MA+LT 1500	Meloidae A	0.71
MA 1500	Paederinae sp2	0.66		MA+LT 1500	*Nisotra* sp2	0.71
MA 1500-1800	*Xyleborus* sp2	0.71		MA+LT 1500	*Pityogenes* sp2	0.48
MA 1800	*Anomala* sp5	0.66		MA+PT 500	Aleocharinae sp3	0.62
MA 1800	*Apogonia* sp4	0.69				
MA 1800	*Lymantor* sp4	0.90				
MA 1800	*Xylothrips* sp1	0.68				

Appendix 12. Twenty most abundantly captured species in our data, sorted according to trap-type specific sample sizes; n indicates number of individuals in the data. Light gray: species shared between light and Malaise traps; dark gray: species shared with pitfall, light and Malaise traps.

Light	n	Malaise	n	Pitfall	n
Anomala_sp1	53	Aleocharinae_sp1	88	Orphnebius_sp1	121
Apogonia_sp2	48	Ischnosoma_sp1	62	Pterostichus_sp2	107
Apogonia_sp1	46	Lymantor_sp2	58	Harpalus_sp1	104
Lymantor_sp3	27	Sinoxylon_sp1	43	Paedarus_sp1	99
Apogonia_sp5	23	Bradymerus_sp2	40	Bledius_sp1	80
Apogonia_sp3	22	Lymantor_sp3	38	Harpalus_sp2	78
Lymantor_sp2	22	Apogonia_sp4	31	Pterostichus_sp1	74
Anomala_sp3	21	Brachypeplus_sp3	31	Pterostichus_sp3	73
Lymantor_sp4	21	Brachypeplus_sp1	30	Inopeplus_sp1	70
Colaspoma_sp2	19	Paederinae_sp3	30	Oxylatus_sp1	59
Anomala_sp2	18	Xyleborus_sp1	24	Orphnebius_sp2	56
Anomala_sp4	16	Galerucinae_sp1	22	Pityogenes_sp1	53
Altica_sp1	15	Lymantor_sp4	19	Anotylus_sp2	48
Apogonia_sp4	15	Anomala_sp3	16	Pentagonica_sp1	45
Illeis_sp2	14	Lymantor_sp1	15	Lispinus_sp1	42
Brachypeplus_sp3	13	Anomala_sp1	14	Staphy_C	41
Pityogenes_sp1	13	Apogonia_sp2	13	Aleocharinae_sp3	32
Brachypeplus_sp2	11	Apogonia_sp1	13	Brachypeplus_sp1	31
Cicindela_sp2	11	Alticinae_sp1	13	Lebia_sp2	30
Hydrovatus_enigmaticus	11	Pityogenes_sp1	11	Staphy_M	30

Figure captions

Fig. 1. Study location in Malaysia. Left: map of Malaysia, with major mountain regions shown with gray; the two squares show locations of Fraser's Hill (top right) and Genting Highland (bottom right) mountains. Right-hand graphs show locations of ten study stands in each study mountain (denoted with letters A-J; compare Table 1); black lines show major highways; Altitudes are indicated with numbers (500, 1,000, 1,500 or 1,800 m a.s.l.). In the bottom right-hand graph, the gray patch shows a forest reserve.

Fig. 2. Rarefaction curves for the three trap types and the pooled sample. The end point of each curve indicates the trap-type specific or pooled (total) number of individuals.

Fig. 3. Average samples + SE of four measures of species richness for three sampling methods according to Altitude. Number of species, and rarefaction standardized richness based on 5, 10 and 20 individuals are shown. Note that the X axis length for pitfall trap samples is different.

Fig. 4. Rank abundance (mean + SE) of twenty most abundant species in each trap type at different Altitudes (500, 1,000, 1,500 and 1,800 m a.s.l.). Note that species are mostly different for each trap type, each sorted according to the rank order of the total number of individuals in that trap type. Grey columns show species that were shared between light and Malaise traps, and white columns show the shared species between pitfall, light and pitfall traps (one species). For species identities, see Appendix.

Fig. 5. NMDS plots for Malaysian beetle data, separate runs for three sampling methods (light, Malaise or pitfall traps) at different Altitudes (500 m = black squares, 1,000 m = red circles, 1,500 m = green up-triangles and 1,800 m = blue down-triangles). For statistical comparisons between Altitudes, see Table 3.

Fig. 6. MRT for sampling-method specific beetle data, using mountain (Fraser's Hill or Genting Highland) and Altitude (500, 1,000, 1,500 or 1,800 m a.s.l.) as explanatory variables. The column plots show relative abundance of each species captured using a given trap type, sorted according to the rank order of abundance of the total trap-type specific sample. Numbers in parentheses below each end branch show the number of samples falling into that branch.
