# Supplementary material for: The functioning of different beetle (Coleoptera) sampling methods across altitudinal gradients in Peninsular Malaysia
Source: PLoS One. 2022 Mar 31;17(3):e0266076. doi: 10.1371/journal.pone.0266076 (PMC8970512; doi:10.1371/journal.pone.0266076)
Supplement: Attachment — Submitted filename: PONE-D-21-03456_R2_reviewer.pdf [file pone.0266076.s011.pdf]

PLOS ONE

The functioning of different beetle (Coleoptera) sampling methods across altitudinal gradients in Peninsular Malaysia

--Manuscript Draft--

**Manuscript Number:**	PONE-D-21-03456R2
**Article Type:**	Research Article
**Full Title:**	The functioning of different beetle (Coleoptera) sampling methods across altitudinal gradients in Peninsular Malaysia
**Short Title:**	Beetle sampling methods across altitudes in Malaysia
**Corresponding Author:**	Muneeb M. Musthafa, PhD<br>South Eastern University of Sri Lanka<br>Oluvil, Ampara SRI LANKA
**Keywords:**	Biodiversity; altitude; forest; light trap; Malaise trap; pitfall trap; tropical
**Abstract:**	Biodiversity research relies largely on knowledge about species responses to environmental gradients, assessed using some commonly applied sampling method. However, the consistency of detected responses using different sampling methods, and thus the generality of findings, has seldom been assessed in tropical ecosystems. Hence, we studied the response consistency and indicator functioning of beetle assemblages in altitudinal gradients from two mountains in Malaysia, using Malaise, light, and pitfall traps. The data were analyzed using generalized linear mixed-effects models (GLMM), non-metric multidimensional scaling (NMDS), multivariate regression trees (MRT), and indicator species analysis (IndVal). We collected 198 morpho-species of beetles representing 32 families, with a total number of 3,052 individual beetles. The richness measures generally declined with increasing Altitude. The Mountains differed little in terms of light and Malaise trap data but differed remarkably in pitfall-trap data. Only light traps (but not the other trap types) distinguished high from middle or low altitudes in terms of beetle richness and assemblage composition. The lower altitudes hosted about twice as many indicators as middle or high altitudes, and many species were trap-type specific in our data. These results suggest that the three sampling methods reflected the altitudinal gradient in different ways and the detection of community variation in the environment thus depends on the chosen sampling method. However, also the analytical approach appeared important, further underlining the need to use multiple methods in environmental assessments.
**Order of Authors:**	Muneeb M. No Middle Name Musthafa, PhD
	Fauziah Abdullah
	Matti J. Koivula
**Response to Reviewers:**	• If you want to keep this format to describe your results, I recommend rewrite something like this: "Our finds were..."<br>This sentence had been rewritten as suggested<br>• …. only light traps...<br>Corrected as suggested<br>• It seems a little vague, please be more specific describing your results in the abstract. It is your time to show a compilation of your paper. Which is considerable? Which is less so?<br>This sentence is sharpened as follows: “Only light traps (but not the other trap types) distinguished high from middle or<br>low altitudes in terms of beetle richness and assemblage composition.”<br>• Please, rewrite! It seems little confuse this sentence as well as I specified in the first review:<br>Community composition – species, their relative shares, and interactions (biotic responses) –largely determines the general biodiversity response to environmental variation. The Climate change sentence is lost in the text, and don’t add up to your objective, you can use it in the discussion and conclusion part, but it does not make any difference in the introduction.<br>This sentence was indeed unclearly written so we rewrote it as follows: “Community

composition is largely determined by
the relative abundances of, and interactions among, its species, which contribute to the general biodiversity response to
environmental variation." We also omitted most of the climate notes from the manuscript as the reviewer is right in that
we did not directly assess climatic issues.

• Please, rewrite this sentence.
I suggest: "The objective of the present study is evalute the consistency of light, Malaise and pitfall trap samples in
relation to the altitudinal gradient".
Following the reviewers advise, we modified the sentence as: "The objective of the present study is to evaluate the
consistency of light, Malaise and pitfall trap samples in reflecting an altitudinal gradient in tropical Malaysia."

• Exclude this comma: We removed the extra comma.

• Please, see the comment in the map:
Please, try to improve this map. Your map has several problems. I can't see which group of points represent each altitude
(maybe is the map resolution, but it is better represent this with some color in the legend). Some points are overlap
(there are many function in QGIS that you can correct this problem - for example see in points
proprieties/Labels/Callouts). You need to use a better layer or some shapefile, add maps coordinates, add scale, add the
north arrow and structure better the overview. The previous map was better than this map.

• I still don't understand how did you arrange 25 pitfall traps in four altitudes. The map show to me only 20 pitfall traps.
Please, check it.
Thank you for pointing out an error in the text! We had 40 pitfall traps so the text has been clarified as follows: "We
sampled beetles using two Malaise traps, two light traps, and 10 (5 groups of 2) pitfall traps at each altitude (500 m,
1,000 m, 1,500 m, and 1,800 m a.s.l.) at both mountain slopes."

• Why 40 pitfall traps if you describe above 25 pitfall traps in each mountain? Please, check it:
See previous point: we had 40 traps (5 sets of 2 locations at 4 elevations)

• I'm still not convinced with your analysis. You assumed Gaussian distribution, because you evaluated your data using only
Q-Q plots and transform your data. I don't recommend transform your data. It is better to find a better distribution that
fits your data. You should use some analysis which you can test the normality such as shapiro test. Quasi-poisson is not a
distribution. You use Quasi-poisson if you found overdispersion in Poisson model. Did you evaluate your model with
Poisson? There is overdispersion?
Did you perform some diagnostics after to run your model? Did you test the normality of residuals and homogeneity of
variance? Which R package did you perform your analysis?
Please, rewrite your data analysis and check all these things.
We followed the reviewer's advice by adding assessments of GLMM residual normality and heteroskedasticity. Moreover,
as these and the QQ plots sometimes suggested slight departures from normality, we reran the models using a novel
"robust LMM" approach, which is tailored for data with outliers while being technically quite similar to lme4. As these
reruns produced similar results to our initial GLMM, we conclude that the initial GLMM produced reliable results, so we
therefore maintain the initial GLMM results in the main document (but present the robust LMM results in the
Supplementary materials). We think it is also important to realize that the QQ plots in Supplementary files measure
residual normality, which is a commonly used way to assess the appropriateness of GLMM, and that none of the presented

Powered by Editorial Manager® and ProduXion Manager® from Aries Systems Corporation

	analyses are or were done with transformed data (we used transformed data in some trials in the supplementary files of<br>the previous manuscript version, which are now not presented or referred to). We were apparently not very clear with<br>these things earlier on. We thus removed the various GLMM trials from the previous version of Supplementary files and<br>now show the normality, heteroskedasticity and robust LMM results (for comparisons with the initial GLMM) in the<br>Supplementary files.<br>The rest of the information can be found in the manuscript.<br>• How did you analyze the species accumulation curves? Which package? Please, describe this in MM:<br>We restructured Material and Methods to clarify these. (They were initially in the end of Material and Methods.) We now<br>mention each package in text sections where the analysis in question is described.<br>• I didn't see any significant improvement in relation to the previous version. Please, detail more your finds.<br>We have now restructured and rewritten sections in Results to improve the text flow. We are unsure what the reviewer<br>means with detailing findings, but hope that the rewritten parts better fulfil this request too.<br>• I didn't find this Fig: This figure is referred to as Supplementary Table S6, as it is really a table not figure. Apologies for<br>the lack of clarity here!<br>• This seems very speculative. Please, remove: Removed as suggested<br>• Again, this seems very speculative. It is obvious that there are climact changes along the altitudinal gradient, but you<br>don't have this data. You don't test this data with climact factors. Many of your explanations are related with climatic<br>factors. So, why you didn't test the climatic factors? I suggest do less emphasis to climact factors, trying to explain your<br>data and focus in your finds:<br>We agree with the reviewer in that we did not really deal with climate, but many things that change in concert with<br>altitude. This part too was removed as suggested.<br>• So, you can't discuss a half of what you discussed in the previous topic: We agree with the reviewer in that we did not<br>really deal with climate, but many things that change in concert with altitude. This part too was removed as suggested.<br>• The reader will judge this. Please, remove: We see the point here and removed the section as suggested<br>• Whenever possible, we recommend the use of multiple collecting methods in environmental impact assessments on<br>biodiversity: We rewrote the recommendation as suggested by the reviewer.<br>• Figure 1 – Put the names of the mountains in the first image. Identify the images A, B e C: Figure 1 has been re-drawn with all the details.
**Additional Information:**	
**Question**	**Response**
**Financial Disclosure**<br><br>Enter a financial disclosure statement that describes the sources of funding for the work included in this submission. Review the submission guidelines for detailed requirements. View published research articles from *PLOS ONE* for specific examples.<br><br>This statement is required for submission and **will appear in the published article** if	We thank the University of Malaya for financing this study (grant numbers RP004E/13SUS and PG059/2014B).

the submission is accepted. Please make sure it is accurate.<br><br>**Unfunded studies**<br>Enter: *The author(s) received no specific funding for this work.*<br><br>**Funded studies**<br>Enter a statement with the following details:<br>• Initials of the authors who received each award<br>• Grant numbers awarded to each author<br>• The full name of each funder<br>• URL of each funder website<br>• Did the sponsors or funders play any role in the study design, data collection and analysis, decision to publish, or preparation of the manuscript?<br>• **NO** - Include this sentence at the end of your statement: *The funders had no role in study design, data collection and analysis, decision to publish, or preparation of the manuscript.*<br>• **YES** - Specify the role(s) played.<br><br>* typeset	
**Competing Interests**<br><br>Use the instructions below to enter a competing interest statement for this submission. On behalf of all authors, disclose any competing interests that could be perceived to bias this work—acknowledging all financial support and any other relevant financial or non-financial competing interests.<br><br>This statement is required for submission and **will appear in the published article** if the submission is accepted. Please make sure it is accurate and that any funding sources listed in your Funding Information later in the submission form are also declared in your Financial Disclosure statement.<br><br>View published research articles from *PLOS ONE* for specific examples.	NO authors have competing interests

**NO authors have competing interests**<br><br>Enter: *The authors have declared that no competing interests exist.*<br><br>**Authors with competing interests**<br><br>Enter competing interest details beginning with this statement:<br><br>*I have read the journal's policy and the authors of this manuscript have the following competing interests: [insert competing interests here]*<br><br>* typeset	
**Ethics Statement**<br><br>Enter an ethics statement for this submission. This statement is required if the study involved:<br><br>• Human participants<br>• Human specimens or tissue<br>• Vertebrate animals or cephalopods<br>• Vertebrate embryos or tissues<br>• Field research<br><br>Write "N/A" if the submission does not require an ethics statement.<br><br>General guidance is provided below. Consult the submission guidelines for detailed instructions. **Make sure that all information entered here is included in the Methods section of the manuscript.**	The collection permits for Fraser's Hill and Genting Highlands were obtained from Forest Department of Malaysia.

**Format for specific study types**<br><br>**Human Subject Research (involving human participants and/or tissue)**<br>• Give the name of the institutional review board or ethics committee that approved the study<br>• Include the approval number and/or a statement indicating approval of this research<br>• Indicate the form of consent obtained (written/oral) or the reason that consent was not obtained (e.g. the data were analyzed anonymously)<br><br>**Animal Research (involving vertebrate animals, embryos or tissues)**<br>• Provide the name of the Institutional Animal Care and Use Committee (IACUC) or other relevant ethics board that reviewed the study protocol, and indicate whether they approved this research or granted a formal waiver of ethical approval<br>• Include an approval number if one was obtained<br>• If the study involved *non-human primates*, add *additional details* about animal welfare and steps taken to ameliorate suffering<br>• If anesthesia, euthanasia, or any kind of animal sacrifice is part of the study, include briefly which substances and/or methods were applied<br><br>**Field Research**<br><br>Include the following details if this study involves the collection of plant, animal, or other materials from a natural setting:<br>• Field permit number<br>• Name of the institution or relevant body that granted permission	
**Data Availability**<br><br>Authors are required to make all data underlying the findings described fully available, without restriction, and from the time of publication. PLOS allows rare exceptions to address legal and ethical concerns. See the PLOS Data Policy and FAQ for detailed information.	Yes - all data are fully available without restriction

<table>
<tr><td>A Data Availability Statement describing where the data can be found is required at submission. Your answers to this question constitute the Data Availability Statement and will be published in the article, if accepted.

Important: Stating ‘data available on request from the author’ is not sufficient. If your data are only available upon request, select ‘No’ for the first question and explain your exceptional situation in the text box.

Do the authors confirm that all data underlying the findings described in their manuscript are fully available without restriction?</td><td></td></tr>
<tr><td>Describe where the data may be found in full sentences. If you are copying our sample text, replace any instances of XXX with the appropriate details.

• If the data are held or will be held in a public repository, include URLs, accession numbers or DOIs. If this information will only be available after acceptance, indicate this by ticking the box below. For example: All XXX files are available from the XXX database (accession number(s) XXX, XXX.).
• If the data are all contained within the manuscript and/or Supporting Information files, enter the following: All relevant data are within the manuscript and its Supporting Information files.
• If neither of these applies but you are able to provide details of access elsewhere, with or without limitations, please do so. For example:

Data cannot be shared publicly because of [XXX]. Data are available from the XXX Institutional Data Access / Ethics Committee (contact via XXX) for researchers who meet the criteria for access to confidential data.

The data underlying the results presented in the study are available from (include the name of the third party</td><td>Data is attached as supplementary information along with the manuscript.</td></tr>
</table>

and contact information or URL).<br>• This text is appropriate if the data are owned by a third party and authors do not have permission to share the data.<br><br>* typeset	
Additional data availability information:	Tick here if the URLs/accession numbers/DOIs will be available only after acceptance of the manuscript for publication so that we can ensure their inclusion before publication.

Powered by Editorial Manager® and ProduXion Manager® from Aries Systems Corporation

Manuscript Click here to access/download;Manuscript;Manuscript.docx

Full Title:

The functioning of different beetle (Coleoptera) sampling methods across altitudinal gradients in Peninsular Malaysia

Short Title: Beetle sampling methods across altitudes in Malaysia

Muneeb M. Musthafa[1,2,3 *], Fauziah Abdullah[2,3,4] and Matti J. Koivula[5]

[1]Department of Biosystems Technology, Faculty of Technology, South Eastern University of Sri Lanka, University Park, Oluvil, #32360, Sri Lanka.

[2]Institute of Biological Science, Faculty of Science, University of Malaya, 50603, Kuala Lumpur, Malaysia.

[3]B513, Toxicology Lab, Institute of Postgraduate Studies, University Malaya, 50603, Kuala Lumpur, Malaysia.

[4]Center of Biotechnology in Agriculture, University Malaya, 50603, Kuala Lumpur, Malaysia

[5] Natural Resources Institute Finland (LUKE), P. O. Box 2, FI-00791 Helsinki, Finland.

* Corresponding author: muneeb@seu.ac.lk

Author contributions

Muneeb M. Musthafa: Initial research idea, fieldwork, data curation, manuscript writing, and technical editing.

Fauziah Abdullah: Conceptualization of the research and methodological design, manuscript writing.

Matti J. Koivula: Statistical analysis, manuscript writing, and technical editing.

Abstract

Biodiversity research relies largely on knowledge about species responses to environmental gradients, assessed using some commonly applied sampling method. However, the consistency of detected responses using different sampling methods, and thus the generality of findings, has seldom been assessed in tropical ecosystems. Hence, we studied the response consistency and indicator functioning of beetle assemblages in altitudinal gradients from two mountains in Malaysia, using Malaise, light, and pitfall traps. The data were analyzed using generalized linear mixed-effects models (GLMM), non-metric multidimensional scaling (NMDS), multivariate regression trees (MRT), and indicator species analysis (IndVal). We collected 198 morpho-species of beetles representing 32 families, with a total number of 3,052 individual beetles. The richness measures generally declined with increasing Altitude. The Mountains differed little in terms of light and Malaise trap data but differed remarkably in pitfall-trap data. Only light traps (but not the other trap types) distinguished high from middle or low altitudes in terms of beetle richness and assemblage composition. The lower altitudes hosted about twice as many indicators as middle or high altitudes, and many species were trap-type specific in our data. These results suggest that the three sampling methods reflected the altitudinal gradient in different ways and the detection of community variation in the environment thus depends on the chosen sampling method. However, also the analytical approach appeared important, further underlining the need to use multiple methods in environmental assessments.

Keywords: biodiversity, altitude, forest, light trap, Malaise trap, pitfall trap, tropical.

Introduction

Most hotspots for the global biodiversity can be found in tropical regions (Mittermeier et al. 2011) which are thus of central importance for conservation. Tropical species often have limited distributions, particularly those species that occupy higher altitudes at mountain slopes (Cox et al. 2016). The majority of the world's vertebrates, for instance, occupy mountain regions that cover only one-fourth of terrestrial landmass (excluding Antarctica; Rahbek et al. 2019). Such mountain species are often endemic to these regions, and also sometimes such that may not be able to move elsewhere if conditions turn unfavorable for them due to, for example, habitat destruction or climate change (Escobar et al., 2005; Elsen & Tingley 2015). Hence, studies on mountain species communities provide crucial information for global conservation efforts, in addition to these environments being particularly vulnerable to habitat degradation and alterations in landscape structure (Körner 2003; Rangwala & Miller 2012).

Community composition is largely determined by the relative abundances of, and interactions among, its species, which contribute to the general biodiversity response to environmental variation. Communities vary according to, among other aspects, latitude and altitude (Hooper et al. 2005; Ashton et al. 2015; Bouzan et al. 2015). Our understanding regarding this variation relies on some commonly assessed taxonomic groups and associated sampling methods. Similarly, land-use planning or conservation decisions may be based on only a handful of well-known species or a single sampling method. Insects, for instance, are often sampled using light, Malaise, or pitfall traps (e.g., Grootaert et al. 2010). Environmental assessments, however, are often based on just one sampling method, which is assumed to produce a general biodiversity response, but this assumption has seldom been challenged.

Earlier research indicates that the three mentioned trap types differ in their efficiency in capturing specimens and species (e.g., Hansen 1988; Ulyshen et al. 2005; Devigne & De Biseau 2014)

though the efficiency could vary with the composition of the local species community determined by, for example, altitude or habitat type. As different methods are known to capture partly different species (e.g., Missa et al. 2009), their use may, at least theoretically, result in different conclusions and hence applications in land use or conservation.

The objective of the present study is to evaluate the consistency of light, Malaise and pitfall trap samples in reflecting an altitudinal gradient in tropical Malaysia. . More specifically, we compare the three sampling methods across the altitudinal gradient from four viewpoints:

(1) Assemblage species richness;

(2) Assemblage species-compositional turnover;

(3) Assemblage composition; and

(4) Species associated with different combinations of sampling method and altitude

Materials and Methods

Study sites

The Titiwangsa mountain range dominates the landscape of Peninsular Malaysia. Within this range, we sampled beetles at Fraser's Hill (3°43’ N, 101°45’ E) and Genting Highland (3°25' N, 101°47' E) that are 95 km apart (Fig. 1). Fraser’s Hill mountain tops peak at between 1,000 and 1,800 m a.s.l., whereas the Genting Highland peaks at about 1,800 m a.s.l. In this region, wet and dry seasons cannot be differentiated, as the annual rainfall of 1,800–3,500 mm is distributed throughout the year (Cheong 2013; Ya’cob et al. 2016). Collection permits for Fraser’s Hill and Genting Highlands were obtained from the Forest Department of Malaysia.

Fraser's Hill, locally known as Bukit Fraser, is a well-preserved permanently-protected nature reserve located at the Raub district of Pahang state. Fraser's Hill has been developed as a hill station dating back in 1919 (Freeman 1999), where 90% of 2,800-ha land area is covered by forests (Kiew 1998; Cheong 2013). Fraser's Hill comprises residential areas, commercial complexes, community services, and recreational facilities. The forests mainly consist of tropical montane cloud forests. Lower montane forests – at about 500–1,200 m a.s.l. – are dominated by Fagaceae and Lauraceae trees, whereas upper montane forests – from about 1,200 to 1,800 m a.s.l. – are dominated by Coniferae, Ericaceae, and Myrtaceae trees (Perumal & Lo 1998).

Genting Highland is perhaps the most disturbed cloud forest in the Malaysian mountains. The summit is covered by amusement parks, casinos, and hotels (Peh et al. 2011). The highest peaks at Genting Highland reach 1,800 m a.s.l, where 96% of the total of 3,965 ha of land is still covered with mostly primary forests (Cheong 2013). Before Genting Highland became an entertainment site, it was an undisturbed forest that could be reached only via jungle trekking (Piggott 1977; Stone 1981).

Sampling methods

We sampled beetles using two Malaise traps, two light traps, and 10 (5 groups of 2) pitfall traps at each altitude (500 m, 1,000 m, 1,500 m, and 1,800 m a.s.l.) at both mountain slopes. All the traps (or trap groups for pitfall traps) were set at least 150 m apart at a given altitude and were placed at least 150 m from the nearest main road. These roads and a river split the sampled forests into a total of twenty distinctive stands; each of these had at least one type of trap (Fig. 1, Table 1).

Malaise traps consisted of a nylon net connected to a collection jar, half filled with 70% ethanol and attached to a tree branch about one meter above the ground. Light traps had a mosquito net

with attached 160-watt mercury bulb connected to a portable Honda EU10i generator. Pitfall traps were plastic cups (diameter 65 mm, depth 95 mm) partly filled with 70% ethanol and dug into the ground with the rim flush with the soil surface. We placed large dry leaves above each pitfall trap to protect them from litter and rain.

We sampled beetles once per month in October 2014, and March, June, and September 2015. At each collecting date, Malaise and pitfall traps were set for 24 hours, starting at 08:00 AM, and light traps operated from 18:00 to 23:30. In the latter, beetles were obtained manually from the traps using collection bottles and aspirators. We occasionally continued to use the light traps until the next morning at 06:00 AM but did not capture additional beetles.

In statistical analyses, we pooled the four periods for each trap (or trap group for pitfall traps) and consider each trap (or trap group for pitfall traps) as a replicate (initially 72; 4 altitudes and 2 mountains, each with 2 light and 2 Malaise, and 5 groups of pitfall traps).

Identification of specimens

We sorted, counted, and cross-checked all beetle specimens using available keys (Saito 2003; Mohamedsaid 2004; Naomi 2006; Schawaller 2006, 2012; Schimmel & Tarnawski 2007; Assing 2008; Hlaváč et al. 2011; Prathapan & Viraktamath 2011; Ryvkin 2011; Shi et al. 2013; Filippini et al. 2016; Anzaldo 2017; Makranczy 2017; Sasakawa et al. 2017). We confirmed the identification of difficult specimens at collections of the Wildlife Department of Malaysia, University of Malaya, National University of Malaysia, and Forestry Department of Malaysia. The identification of beetles to species is hampered by the lack of experts and species compilations. In the present study, the samples contained at least five species new to science, which will be

described in later papers. Clearly, the often-broad taxonomic levels and the shortage of knowledge about the ecological traits of species must be acknowledged while interpreting results, as similar-looking species might be different in terms of, for example, life cycles, diets, abilities to disperse, and habitat requirements.

Statistical analysis

We had initially 72 samples (16 for light, 16 for Malaise, and 40 for pitfall traps (5 sets X 2 X 4 elevations)). Three samples, however, produced only 1-2 species and were excluded due to difficulty in calculating pair-wise dissimilarities and richness estimates (one light and two Malaise trap samples). Thus, we ran all analyses with 69 samples (Table 1). We ran the following analyses for the four viewpoints proposed above.

Assemblage richness:

To assess sampling-method dependent variation in species richness, we subjected the beetle data to a generalized linear mixed-effects model (GLMM; Zuur et al. 2009) to quantify variation according to altitude (500, 1,000, 1,500, or 1,800 m a.s.l.). We ran the model separately for each sampling method (light, Malaise, or pitfall traps; hereafter "Method" for brevity) to be able to compare their similarity in responses to altitude. We subjected the observed number of species and richness estimates based on rarefaction standardization to 5, 10, and 20 individuals, to a GLMM with Altitude as a fixed factor and Mountain (Fraser's Hill or Genting Highland; Fig. 1, Table 1) as a random factor (to account for spatial autocorrelation), using lme4 (Bates et al. 2015). For full model outputs, see Supplementary Table S1.

For linear models it is important to assess the normality and homoskedasticity of model residuals, which we did in two ways. Firstly, we inspected Q-Q plots of GLMM residuals visually (car

package; Fox & Weisberg 2018), confirmed by Wilk-Shapiro test for residual normality (Supplementary Figure S1 and Table S2). These did not indicate major departures from normality, except in the pitfall-trap data. Secondly, we ran Wilk-Shapiro test to check the normality, and Breusch-Pagan test to assess homoskedasticity, of ANOVA residuals (model Mountain + Altitude; Supplementary Table S3). These checks indicated the following issues: (i) heteroskedasticity in the number of species and non-normality in the rarefied richness to 5 individuals in the light-trap data; (ii) heteroskedasticity in the number of species in the Malaise-trap data; and (iii) non-normality in the rarefied richness standardized to 5 and 10 individuals in the pitfall-trap data (as for GLMM residuals above). Due to these deviations from normality, we reran the GLMM models (as in Table 2) using Robust LMM (robustlmm package; Koller 2016). Models run using biological data often contain outlier samples that may render residual distributions non-normal or heteroskedastic. Estimates from Robust LMM are little affected by such outliers, if the tuning parameter (k) is set at a low value; values approaching ∞ produce results similar to a normal GLMM. Here, we applied the smoothed Huber ($k = 1.345$, $s = 10$) function for fitting random effects (Mountain) and variance component (Altitude), as recommended by Koller (2016) (Supplementary Table S4). As the relative magnitudes and directions of estimates in Robust LMM were very similar to the initial GLMM, we conclude that our GLMM results shown in Table 2 are robust.

Assemblage turnover:

To examine possible species turnover according to altitude and Method, we calculated averages and standard errors for 20 most abundant species, separately for each Altitude and Method. We plotted these values according to Altitude, using the rank order of the abundances of species captured using each Method. We evaluated the community turnover, or distinctiveness of samples,

between Altitudes using permutational multivariate ANOVA (the adonis function in R package vegan, with Mountain [Fraser's Hill or Genting Highland] as strata; Oksanen et al. 2018). Here, we only considered single or combinations of subsequent Altitudes. Thus, for example, a combination 500 + 1000 m was considered but not 500 + 1800 m. As a simple measure of turnover between Methods, we indicated in these plots species that were unique for a given Method, and those species that were shared with 1–2 other Methods. We confirmed this comparison of Methods using permutational multivariate ANOVA, as described above.

Assemblage composition:

To examine beetle assemblage composition across Altitudes and Methods, we used two analyses. Firstly, we used non-metric multidimensional scaling (NMDS; Borg & Groenen 2005) to assess variation in community composition, using the vegan package (Oksanen et al. 2018). We used Method-specific data sets by applying a Bray-Curtis dissimilarity matrix. We used the above-described permutational multivariate ANOVA for Altitudes as a confirmation of the NMDS result. Secondly, we subjected the Method-specific beetle data sets to multivariate regression tree analysis (MRT; De'ath 2002) based on the Bray-Curtis dissimilarity matrix, using the mvpart package (De'ath et al. 2014). We used altitude and, as our earlier research has indicated differences in beetle faunas between Fraser's Hill and Genting Highland (Musthafa et al. 2018), Mountain as explanatory variables. MRT identifies groups of samples as defined by explanatory variables (Mountain and Altitude) and is not restricted by non-linearities (De'ath 2002). We present the result as a tree of dichotomies, where each dichotomy is based on minimizing the dissimilarity of samples within each tree branch. We report the tree with the lowest cross-validated relative error, following the 1-SE rule (Breiman et al. 2017). Cross-validated relative error provides a better estimate than relative error for the predictive accuracy of the MRT for a new dataset (De'ath 2002).

Species associations with Altitudes and Methods:

To detect species characteristic to particular combinations of Method and Altitudes, we calculated an indicator value (IndVal; Dufrêne & Legendre 1997, De Cáceres et al. 2010) for each species, based on all logical combinations of Method and Altitude. Here, we used the indicspecies package (De Cáceres & Jansen 2016) and allowed each Method to appear singly or jointly with 1–2 other Methods, whereas for Altitude we only considered single, or combinations of subsequent, Altitudes, as described above. We restricted the IndVal to species with a total sample of at least five individuals.

Results

Beetle richness according to Mountain and Altitude

We collected 198 morpho-species of beetles representing 32 families with a total number of 3052 individual beetles (Supplementary Table S5). Nine were identified to species, 143 to genus, and 43 to higher taxonomic levels. We refer to all these as “species” below for convenience. Regarding Method, we collected 107 species using light traps, 127 using Malaise, and 45 using pitfall traps. A total of 135 species were represented by only one Method, whereas 45 had been captured with two and 18 with all three sampling methods. Altogether 98 species were singletons or doubletons, whereas 84 were represented by at least five individuals (Supplementary Table S5). Species accumulation curves based on rarefaction suggested that pitfall traps had captured nearly all potential species at about 200 individuals at both Mountains, whereas the accumulation curves for light and Malaise traps were still very steep at about 400-500 individuals (Fig. 2).

The GLMM performed rather similarly in terms of the four measures of richness, but the three Methods produced partly different results (Table 2, Fig. 2; Supplementary Table S1). The Mountains differed little in terms of light and Malaise trap data but remarkably in pitfall-trap data. The richness measures based on light traps declined with Altitude but only 1,800 m differed significantly from 500 m. The decline with Altitude occurred also in Malaise traps so that all higher Altitudes differed significantly from 500 m. Richness measures based on pitfall traps, on the other hand, peaked at 1,000 m (Table 2, Fig. 2).

Species turnover

The rank-abundance plots of twenty most abundant species (Fig. 4) reflected relatively high similarity between light and Malaise traps, with nine of the 20 most numerous species being shared (grey columns), whereas only one of the 20 dominant species – *Pityogenes* sp1 – in pitfall-trap samples was shared with light- and Malaise-trap samples (white columns). For species identities in this graph, see Supplementary Table S6. Permutational multivariate ANOVA indicated that also light and Malaise traps were compositionally different; for light vs. Malaise traps, F = 3.21, R2 = 0.11, p = 0.0010; for light vs. pitfall traps F = 9.41, R2 = 0.15, p = 0.0010; and for Malaise vs. pitfall traps F = 10.48, R2 = 0.17, p = 0.0010. Moreover, the plots suggested more abundance or occurrence changes with Altitude in Malaise and light traps, whereas the plots varied less and more erratically in pitfall-trap samples (Fig. 4). Another striking pattern in light-trap samples (Fig. 5, left) was the lack of the 12 most abundant species at 1,800 m. Only 9% of light-trap species and 17% of Malaise-trap species were found in both 500 m and 1,800 m, whereas the percentage was 63 for pitfall-trap samples.

Permutational multivariate ANOVA confirmed compositional changes with Altitude, particularly at 1,800 m, but the three Methods showed different patterns in this respect (Table 3). In light traps,

only 1,800 m differed significantly from the other Altitudes; however, all except one combination of 2–3 Altitudes also differed significantly from the rest of the Altitudes. The patterns were similar for Malaise traps, except that different combinations of 2–3 Altitudes differed less commonly from the rest of the Altitudes. Regarding pitfall traps, then, all Altitudes and their combinations (except for 1,000–1,500 m) had distinctive assemblages (Table 3).

Assemblage composition according to Method and Altitude

According to NMDS, samples in light and Malaise traps distinguished 1,800 m from the three lower altitudes, whereas pitfall trap samples formed two sample clusters, of which only the other distinguished clearly between Altitudes (Fig. 6). Thus, in pitfall-trap samples, Altitude was reflected in Fraser's Hill but not in Genting Highland (on the left and right, respectively, in Fig. 5).

MRT for light-trap data consistently resulted in a one-dichotomy tree (Fig. 6). The only division, where 1,800 m was split from the rest of the Altitudes, explained 47% of the variation in light-trap data, the former being species poor according to branch-specific rank-abundance plots (Fig. 6). Malaise-trap data, on the other hand, suggested that the same dichotomy, based on Altitude, was apparent in Fraser's Hill but not in Genting Highland; again, 1,800 m had fewer species though this was based on only two samples. This two-dichotomy tree explained 81% of the variation in the Malaise trap data (Fig. 6). Also, pitfall trap data suggested that Altitudes could be distinguished at Fraser's Hill but not at Genting highland; here, 1,500 and 1,800 m diverged from the two lower Altitudes that, in turn, were split in the third dichotomy (Fig. 6).

Species associated with different combinations of Method and Altitude

We found 83 indicator species for different combinations of Method and Altitude (Table 4). Thirteen species were commonly caught with two sampling methods (12 for light and Malaise

traps, and one with Malaise and pitfall traps). Moreover, eight species indicated pitfall traps across the full altitudinal range (500-1,800 m), whereas we did not detect such altitude-independent indicators for the two other sampling methods. Light traps produced 12 indicators of low (500-1,000 m), six of the middle (1,000-1,500 m) and seven of high (1,500-1,800 m) Altitudes, whereas the respective numbers were 16, four, and six for Malaise and ten, eleven and five for pitfall traps (Table 4). Moreover, three species occurred in pitfall traps across a wider Altitudinal range: two species were found at 500-1,500 m and one at 1,000-1,800 m.

Discussion

Beetle species turnover between methods and across the Altitudinal gradient

We found that light and Malaise trap samples shared about half of the abundant species, whereas pitfall trap samples were distinctive in this respect. Light and Malaise traps may have attracted a shared pool of species that flies actively, whereas pitfall traps capture mostly ground dwellers that seldom fall into the two other types of traps (e.g., Missa et al. 2009). This finding suggests that, at least in the study region, the variation in beetle communities caused by altitude or climatic factors may be better captured if pitfall traps are used together with either light or Malaise traps, whereas a combination of light and Malaise traps may not be equally efficient.

At 1,800 m light (but not Malaise or pitfall) trap samples lost all 12 most abundant species of the total light-trap sample. This loss might have resulted from the captured species pool being more sensitive to altitude-associated changes in abiotic or biotic conditions. However, some of the 12 “lost” species were still captured at 1,800 m using Malaise traps, so another explanation may be trap functioning. Perhaps light or wind conditions – or some other factors related to, for example, vegetation or moisture – were different at the highest altitudes, which might, in turn, have impacted

the visibility of light traps or flight activity of many species (Mathews & Mathews 1971; McGeachie 1989; Sheikh et al. 2016; Nowinszky & Puskás 2017; Skvarla & Dowling 2017). It would be important to continue beetle monitoring at these mountain tops to see whether the altitudinal distributions of species indeed change with climate and whether the currently dominant mountain-top species persist or disappear.

The relative similarity of rank-abundance plots at different altitudes, particularly for pitfall-trap samples, may have occurred because many dominant species are adapted to a wide range of altitudes (e.g., Nunes et al. 2016) and/or temperature or moisture conditions (Gilchrist, 1995; Saavedra et al., 2013). Pitfall traps capture mostly ground dwellers, and field-layer vegetation or soil conditions may thus have kept conditions relatively constant across the altitudinal gradient for these species. These factors might decrease variation in micro-climate (McCain & Grytnes 2001), perhaps through the sheltering effect of forest trees (Barnes et al. 1997).

Beetle community structure across the altitudinal gradient using different methods

Our community analyses suggest that the overall beetle community varied remarkably according to altitude, but the magnitude of this response depended on geographic location (the two mountains) and captured species subset (sampling method). Thus, according to NMDS and MRT, light traps distinguished 1,800 m from lower altitudes, whereas Malaise and pitfall traps reflected primarily differences between the two mountains and only secondarily altitudinal variation. Moreover, and only at Fraser’s Hill, Malaise traps distinguished 1,800 m from the lower altitudes, and pitfall traps distinguished 500, 1,000, and 1,500-1,800 m.

Indicator species for sampling methods and altitude

Pitfall traps produced eight indicator species that were common at all altitudes, whereas the other sampling methods did not produce such altitude generalists (for identities of these species, see Table 4). This finding is in line with our rank-abundance plots (Fig. 4). Moreover, all sampling methods produced at least some indicators of low (500–1,000 m), intermediate (1,000–1,500 m), and/or high altitudes (1,500–1,800 m), suggesting potential for each method to reflect altitude and thus potentially global changes in climate and land use. Important from a climate perspective, we found nine significant indicators of 1,800 m (four using light, four using Malaise, and one using pitfall traps). Species adapted to these highest mountain tops may face difficulty in survival with predicted climate warming (e.g., Lenoir et al. 2008; Pickering et al. 2008; Dirnböck et al. 2011; Elsen et al., 2020) unless they can adapt or disperse elsewhere. Good dispersal ability of beetles allows them to rapidly move towards higher altitudes with warmer temperature (Bale et al., 2002; Williams & Liebhold, 2002). Moreover, beetles may lengthen their flight-activity period and increase the number of generations produced per year to compensate with changing climate, most notably temperature (Menéndez, 2007; Altermatt, 2010). Moreover, thermal adaptability of species could regulate the distributions and local adaptations of beetles across altitudinal gradients, since temperature intensely influences their flight activity (Calosi et al., 2010; Sunday et al., 2012; García-Robledo et al., 2016). In South Korea, for example, *Onthophagus* beetles (Coleoptera: Scarabaeidae) have been predicted to move to higher altitudes with increasing ambient temperature (Kwon et al. 2015). The dispersal ability is high also for many European Curculionidae (Bouget et al., 2015) and dung beetles (Gómez et al., 2020). For most Malaysian beetle species, however, the level of knowledge about such traits is low. Nevertheless, some of the species in the present data are apparently specialists of narrow temperature ranges, such as *Orphnebius* (Staphylinidae) and *Rhadinomerus* species (Curculionidae), whereas *Brachypeplus* species may be considered a

generalist in terms of temperature conditions in the studied mountain slopes (Musthafa, 2018). On the other hand, *Inopeplus* (Salpingidae), *Macrotomoderus* (Anthicidae), Paederinae (Staphylinidae) and *Pentagonica* species (Carabidae) are temperature specialists whereas *Stenus* species (Staphylinidae) can be considered a temperature-range generalist (Musthafa, 2018).

The precautionary principle calls for mitigation of climatic or land-use alterations at high altitudes (Gallou et al. 2017). In our study area, for instance, the two mountains hosted very different beetle communities, which perhaps partly reflects different intensities of land use. The studied sampling methods differ in costs, required labor, relative capturing efficiency, and captured species pool. The differences reported here suggest that the use of more than one location and several sampling methods are desirable in environmental assessments (Lamarre et al. 2012; Devigne & De Biseau 2014; Abdullah & Musthafa 2019).

Research caveats

Taxonomic constraints may have affected our results to some extent, as most taxa had been identified to family or genus levels only. Thus, two individuals of a given taxon being captured using two methods or at different altitudes might in reality be two different species. However, this possibility rather masks than exaggerates the true community responses. We are therefore confident that the sampling methods and altitudes truly differed in beetle communities, but the differences would have been more pronounced had we been able to identify everything to species.

Conclusions

The location of the present study, Malaysia, belongs to the global biodiversity hotspot of Sundaland, yet little is known about the invertebrate diversity of its mountains (Myers et al. 2000, Doll et al. 2014). Results of our study are applicable to tropical species conservation: they provide

evidence for adaptations of many species to particular altitudes and, more importantly, differences in beetle samples between collecting methods. The three sampling methods reflected the altitudinal gradient in different ways. This, in turn, calls for caution in interpretations of data based on only one method and thus on a narrow range of species life histories. Whenever possible, we strongly recommend multiple collecting methods in environmental impact assessments on biodiversity.. This is particularly important in land-use or political decision making, which should ideally be based on a holistic picture of biodiversity to avoid unwanted species losses or changes in ecosystem functioning.

Acknowledgments

We thank Mohd Shukri Mohd Sabri and Davindram A/L Rajendram for assistance in beetle sampling. We also acknowledge the Universiti Kebangsaan Malaysia and Forestry Department of Malaysia for supporting this study, and Marcelo Bruno Pessôa and an anonymous reviewer for constructive comments.

References

Abdullah F, Musthafa MM. Beetle abundance, diversity and species richness of Pulau Pangkor, Perak, Malaysia. Malaysia Forester 2019; 82: 247-270.

Altermatt F. Climatic warming increases voltinism in European butterflies and moths. Proc Biol Sci. 2010; 277(1685):1281-1287. doi:10.1098/rspb.2009.1910.

Anzaldo SS. Review of the genera of Conoderinae (Coleoptera, Curculionidae) from North America, Central America, and the Caribbean. ZooKeys 2017; 683: 51-138. https://doi.org/10.3897/zookeys.683.12080.

Ashton LA, Odell EH, Burwell CJ, Maunsell SC, Nakamura A, McDonald WJF, Kitching RL. Altitudinal patterns of moth diversity in tropical and subtropical Australian rainforests. *Austral Ecol* 2015; 41: 197-208. https://doi.org/10.1111/aec.12309.

Assing V. Four new species and additional records of Palaearctic *Sunius*, with two new synonymies (Coleoptera: Staphylinidae: Paederinae). *Beiträge zur èntomologie* 2008; 58: 455-470. https://doi.org/10.21248/contrib.entomol.58.2.455-470.

Bale JS, Masters GJ, Hodkinson ID, Caroline A, Martijn BT, Valerie KB, … John BW. Herbivory in global climate change research: Direct effects of rising temperature on insect herbivores. Global Change Biology 2002; 8: 1-16. https://doi.org/10.1046/j.1365-2486.2002.00451.x

Baniya CB, Solhøy T, Gauslaa Y, Palmer MW. The elevation gradient of lichen species richness in Nepal. The Lichenologist 2010; 42: 83-96. https://doi.org/10.1017/S0024282909008627

Barnes BV, Zak DR, Denton SR, Spurr SH. Forest ecology. 4th ed. John Wiley & Sons. 1999.

Barzani GM, Sahibin AR, Adam JH, Zulfahmi AR. The physical environment. Bukit Fraser crown of the Titiwangsa Range. In: Latiff, A., Maimon, A., Norhayati, A. & Adam, J.H., editors. Bukit Fraser Research Centre, Universiti Kebangsaan Malaysia & Fraser's Hill Development Corporation, Fraser's Hill Development Corporation, Raub, Malaysia; 2009. pp 9-16.

Bouget C, Brin A, Tellez D, et al. Intraspecific variations in dispersal ability of saproxylic beetles in fragmented forest patches. Oecologia 2015; 177: 911–920. https://doi.org/10.1007/s00442-014-3162-9

Bouzan AM, Flinte V, Macedo MV, Monteiro RF. Elevation and temporal distributions of Chrysomelidae in southeast Brazil with emphasis on the Galerucinae. ZooKeys 2015; 547: 103-117. https://doi.org/10.3897/zookeys.547.9723

Brehm G, Colwell RK. Kluge J. The role of environment and mid-domain effect on moth species richness along a tropical elevational gradient. Glob Ecol Biogeogr 2007; 16: 205-219. https://doi.org/10.1111/j.1466-8238.2006.00281.x

Bates D, Maechler M, Bolker B, Walker S. Fitting linear mixed effects models using lme4. J Stat Softw 2015; 67: 1-48. http://dx.doi.org/10.18637/jss.v067.i01

Borg I, Groenen P. Modern multidimensional scaling: theory and applications. 2nd ed. Springer-Verlag, New York. 2005

Breiman L, Friedman JH, Olshen RA, Stone CG. Classification and regression trees. CRC Press, Boca Raton, Florida. 358 pp. 1998.

Calosi P, Bilton DT, Spicer JI, Votier SC, Atfield A. What determines a species' geographical range? Thermal biology and latitudinal range size relationships in European diving beetles (Coleoptera: Dytiscidae). J Anim Ecol 2010; 79: 194–204. DOI: 10.1111/j.1365-2656.2009.01611.x.

Chao A, Jost, L. Coverage-based rarefaction and extrapolation: standardizing samples by completeness rather than size. Ecol 2012; 93(12): 2533–2547.

Cheong C. Fraser’s Hill - A Lush Highland Hideaway. WWF Malaysia, Petaling Jaya. 2013.

Cox CB, Moore PD, Ladle R. Biogeography: an ecological and evolutionary approach. Wiley Blackwell. 2016.

De Cáceres M, Legendre P, Moretti M. Improving indicator species analysis by combining groups of sites. Oikos 2010; 119: 1674-1684. DOI: 10.1111/j.1600-0706.2010.18334.x

De Cáceres M, Jansen F. Package Indicspecies. 2016. URL https://CRAN.R-project.org/package=indicspecies.

De'ath G. Multivariate regression trees: a new technique for modeling species–environment relationships. Ecol 2002; 83(4): 1105-1117. https://doi.org/10.2307/3071917

De'ath G, Therneau TM, Atkinson B, Ripley B, Oksanen J. mvpart: Multivariate partitioning. 2014. URL https://mran.microsoft.com/snapshot/2014-12-11/web/packages/mvpart/index.html

Devigne C, De Biseau J-C. Urban ecology: comparison of the effectiveness of five traps commonly used to study the biodiversity of flying insects. Biodivers J 2014; 5: 165-174.

Doll HM, Butod E, Harrison RD, Fletcher C, Kassim AR, Ibrahim S, Potts MD. Environmental and geographic factors driving dung beetle (Coleoptera: Scarabaeidae: Scarabaeinae) diversity in the dipterocarp forests of Peninsular Malaysia. Raffles Bull Zool 2014; 62: 549-560.

Dufrêne M, Legendre P. Species assemblages and indicator species: the need for a flexible asymmetrical approach. Ecol Monogr 1997; 67(3): 345-366. https://doi.org/10.1890/0012-9615(1997)067[0345:SAAIST]2.0.CO;2

Dirnböck T, Essl F, Rabitsch W. Disproportional risk for habitat loss of high-altitude endemic species under climate change. Glob Chang Biol 2011; 17: 990-996. https://doi.org/10.1111/j.1365-2486.2010.02266.x

Elsen PR, Tingley MW. Global mountain topography and the fate of montane species under climate change. Nat Clim Chang 2015; 5: 772-776. https://doi.org/10.1038/nclimate2656

Elsen, PR, Monahan, WB, Merenlender, AM. Topography and human pressure in mountain ranges alter expected species responses to climate change. Nat Commun 2020; 11: 1974. https://doi.org/10.1038/s41467-020-15881-x

Escobar F, Lobo JM, Halffter G. Altitudinal variation of dung beetle assemblages in the Colombian Andes. Glob Ecol Biogeogr 2005; 14: 337-347. https://doi.org/10.1111/j.1466-822X.2005.00161.x

Filippini V, Micó E, Galante E. Checklist and identification key of Anomalini (Coleoptera, Scarabaeidae, Rutelinae) of Costa Rica. ZooKeys 2016; 621: 63-136. https://doi.org/10.3897/zookeys.621.7565

Fox J, Weisberg S. An R companion to applied regression. 3rd edition. Sage Publications Inc., California. 2019.

Gallou A, Baillet Y, Ficetola GF, Despres L. Elevational gradient and human effects on butterfly species richness in the French Alps. Ecol Evol 2017; 7(11): 1-10. https://doi.org/10.1002/ece3.2803.

García-Robledo C, Kuprewicz EK, Staines CL, Erwin TL, Kress WJ. Limited tolerance by insects to high temperatures across tropical elevational gradients and the implications of global warming for extinction. PNAS 2016: 113; 680-685. doi: 10.1073/pnas.1507681113

Gilchrist GW. Specialists and generalists in changing environments. I. Fitness landscapes of thermal sensitivity. Am Nat 1995; 146: 252-270.

Gómez VCG, Verdú JR, Zurita GA. Thermal niche helps to explain the ability of dung beetles to exploit disturbed habitats. Sci Rep 2020; 10: 13364. https://doi.org/10.1038/s41598-020-70284-8

Hlaváč P, Newton AF, Maruyama M. World catalogue of the species of the tribe Lomechusini (Staphylinidae: Aleocharinae). Zootaxa 2011; 3075: 1-151.

Hooper DU, Chapin FS III, Ewel JJ, Hector A, Inchausti P, et al. Effects of biodiversity on ecosystem functioning: a consensus of current knowledge. Ecol Monogr 2005; 75: 3-35. https://doi.org/10.1890/04-0922.

Kessler M. Elevational gradients in species richness and endemism of selected plant groups in the central Bolivian Andes. Plant Ecol 2000; 149: 181-193.

Kiew R. The seed plant flora of Fraser's Hill, Peninsular Malaysia: With special reference to its conservation status. Research pamphlet No. 21. Forest Research Institute of Malaysia (FRIM), Kuala Lumpur, Malaysia. 1998.

Kohler T, Wehrli A, Jurek M. Mountains and climate change: A global concern. Sustainable Mountain Development Series. Bern, Switzerland, Centre for Development and

Environment (CDE), Swiss Agency for Development and Cooperation (SDC) and Geographica Bernensia. 2014.

Koivula MJ, Chmaberlain DE, et al. Breeding bird species diversity across gradients of land use from forest to agriculture in Europe. Ecography 2018; 41: 1331-1344. https://doi.org/10.1111/ecog.03295

Koller M. robustlmm: An R Package for Robust Estimation of Linear Mixed-Effects Models. Journal of Statistical Software 2016; 75: 1–24. https://doi: 10.18637/jss

Körner C. Alpine plant life. Functional plant ecology of high mountain ecosystems. 2nd edition. Springer, Berlin. 2003.

Kumar A, Longino JT, Colwell RK, O'Donnell S. Elevational patterns of diversity and abundance of eusocial paper wasps (Vespidae) in Costa Rica. Biotropica 2009; 41, 338-346. https://doi.org/10.1111/j.1744-7429.2008.00483.x.

Kwon T-S, Lee CM, Kim S-S. Prediction of abundance of beetles according to climate warming in South Korea. J Asia Pac Biodivers 2015; 8: 7-30. https://doi.org/10.1016/j.japb.2015.01.001.

Laliberte E, Wells JA, DeClerck F… Mayfield, MM. Land-use intensification reduces functional redundancy and response diversity in plant communities. Ecol Lett 2009; 13: 76-86. https://doi.org/10.1111/j.1461-0248.2009.01403.x

Lamarre GPA, Molto Q, Fine PVA, Baraloto C. A comparison of two common flight interception traps to survey tropical arthropods. ZooKeys 2012; 216: 43-55. https://doi.org/10.3897/zookeys.216.3332

Lenoir J, Gegout JC, Marquet PA, de Ruffray P, Brisse H. A significant upward shift in plant species optimum elevation during the 20th century. Science 2008; 320: 1768-1771. DOI: 10.1126/science.1156831.

Makranczy G. Review of the *Anotylus* cimicoides species group (Coleoptera: Staphylinidae: Oxytelinae). Acta Zoolog Academiae Scientiarum Hung 2017; 63: 143-262. DOI: 10.17109/AZH.63.2.143.2017

Mathews RW., Mathews JR. The Malaise Trap: Its Utility and Potential for Sampling Insect Populations. *The Great Lakes Entomologist* 1971; 4(4): Article 4.

McCain CM, Grytnes JA. Elevation gradients in species richness. In Encyclopedia of Life Sciences (ELS). John Wiley & Sons Ltd., Chichester. 2000.

McGeachie WJ. The effects of moonlight illuminance, temperature and wind speed on light-trap catches of moths. Bull Entomol Res 1989; 79: 185-192.

Menéndez R. How are insects responding to global warming? Tijdschrift voor Entomologie 2007; 150: 355-365.

Mittermeier RA, Turner WR, Larsen FW, Brooks TM, Gascon C. Global Biodiversity Conservation: The Critical Role of Hotspots. In: Zachos, F.E. & Habel, J.C. editors, Biodiversity Hotspots. Springer-Verlag, Berlin-Heidelberg. 2011. Pp. 3-22.

Mohamedsaid MS. Catalogue of the Malaysian Chrysomelidae (Insecta: Coleoptera). Pensoft Publishers, Bulgaria. 2004.

Musthafa, MM. A comparative study of beetle diversity at different elevations at Fraser's hill and Genting Highland. Ph.D. Thesis. University of Malaya. 2018.

Musthafa MM, Abdullah F, Sánchez‐Reyes UJ. Comparative study of spatial patterns and ecological niches of beetles in two Malaysian mountains elevation gradients. J Insect Conserv 2018; 22(5-6): 757-769. https://doi.org/10.1007/s10841-018-0099-z

Myers N, Mittermeier RA, Mittermeier CG, da Fonseca GAB, Kent J. Biodiversity hotspots for conservation priorities. Nature 2000; 403: 853-858.

Naomi S. Taxonomic revision of the genus *Stenus* Latreille, 1797 (Coleoptera, Staphylinidae, Steninae) of Japan: Species group of S. indubius Sharp. Jpn J Syst Entomol 2006; 12: 39-120.

Newbold T, et al. Global effects of land use on local terrestrial biodiversity. Nature 2015; 520: 45-50. https://doi.org/10.1038/nature14324

Nowinszky L, Puskás J. Light-Trap Catch of Insects in Connection with Environmental Factors. In: Vonnie D.C. Shields, editors, *Biological Control of Pest and Vector Insects*, InTech Publishers. 2017. pp. 97-117.

Nunes CA, Braga RF, Figueira JEC, Neves FS, Fernandes GW. Dung beetles along a tropical altitudinal gradient: environmental filtering on taxonomic and functional diversity. PLoS ONE 2016; 11: e0157442. https://doi.org/10.1371/journal.pone.0157442

Oksanen J, Blanchet FG, Friendly M, Kindt R, Legendre P, McGlinn D, et al. Package vegan. Community Ecology Package. 2018. URL https://cran.r-project.org, https://github.com/vegandevs/vegan

Peh KS-H, Soh MCK, Sodhi NS, Laurance WF, Ong DF, Clements R. Up in the clouds: Is sustainable use of tropical montane cloud forests possible in Malaysia? Bioscience 2011; 61: 27–38. https://doi.org/10.1525/bio.2011.61.1.8

Perumal B, Lo FSJ. Recorded mountain trees of Peninsular Malaysia - a checklist. World Wildlife Fund Malaysia, Kuala Lumpur, Malaysia. 1998.

Pickering C, Hill W, Green K. Vascular plant diversity and climate change in the alpine zone of the Snowy Mountains, Australia. Biodivers Conserv 2008; 17: 1627-1644. https://doi.org/10.1007/s10531-008-9371-y

Piggott AG. The ferns of Gunung Ulu Kali. Garden Bulletin of Singapore 1977; 30: 31-43.

Prathapan KD, Viraktamath CA. A new species of *Longitarsus* Latreille, 1829 (Coleoptera, Chrysomelidae, Galerucinae) pupating inside stem aerenchyma of the hydrophyte host from the Oriental Region. ZooKeys 2011; 87: 1-10. doi: 10.3897/zookeys.87.1294

R Core Team. R: A language and environment for statistical computing. R Foundation for Statistical Computing, Vienna, Austria. 2021. URL https://www.R-project.org/

Rahbek C, Borregaard MK, Colwell RK, Dalsgaard B, Holt BG, Morueta-Holme N, ... Fjeldsa J. Humboldt's enigma: What causes global patterns of mountain biodiversity? Science 2019; 365(6458): 1108-1113. https://doi.org/10.1126/science.aax0149.

Rangwala I, Miller JR. Climate change in mountains: a review of elevation-dependent warming and its possible causes. Clim Change 2012; 114: 527-547. https://doi.org/10.1007/s10584-012-0419-3

Ryvkin AB. Contributions to the knowledge of *Stenus* (Nestus) species of the *Crassus* group (Insecta: Coleoptera: Staphylinidae: Steninae). Four new species from the Russian Far East with taxonomic notes. Balt J Coleopterol 2011; 11: 57-72.

Saavedra S, Rohr RP, Dakos V, Bascompte J. Estimating the tolerance of species to the effects of global environmental change. Nat Commun 2013; 4: 2350. https://doi.org/10.1038/ncomms3350

Saito M. A New Species of the genus *Tomoderus* (Coleoptera, Anthicidae) from the Ryukyu Islands, Southwest Japan. *Elytra* 2003; 31: 321-323.

Sasakawa K, Kim J-K, Kim J-K, Kubota K. Morphological phylogeny and biogeography of the *Pterostichus raptor* species group (Coleoptera: Carabidae) of ground beetles, endemic to the Korean Peninsula and adjacent islands. J Asia Pac Entomol 2017; 20: 7-12. https://doi.org/10.1016/j.aspen.2016.11.001

Schawaller W. Revision of the Oriental species of the genus Bradymerus Perroud, with descriptions of 29 new species (Coleoptera: Tenebrionidae). Stuttgarter Beiträge zur Naturkunde 2006; 4: 1-64.

Schawaller W. Two new species and new records of the genus *Spinolyprops* Pic, 1917 from the Oriental Region (Coleoptera, Tenebrionidae, Lupropini). ZooKeys 2012; 243: 83-94. DOI: 10.3897/zookeys.243.3879

Schimmel R, Tarnawski D. The species of the genus *Mulsanteus* Gozis, 1875 of Southeast Asia (Insecta: Coleoptera: Elateridae). Genus 2007; 14, 89-172.

Sheikh AH, Thomas M, Bhandari R, Bunkar K. Light trap and insect sampling: an overview. Int J Curr Res 2016; 8(11): 40868-40873.

Shi H, Zhou H, Liang H. Taxonomic synopsis of the subtribe *Physoderina* (Coleoptera, Carabidae, Lebiini), with species revisions of eight genera. Zookeys 2013; 284: 1-129. https://doi.org/10.3897/zookeys.284.3983

Skvarla MJ, Dowling APG. A comparison of trapping techniques (Coleoptera: Carabidae, Buprestidae, Cerambycidae, and Curculionoidea excluding Scolytinae). J Insect Sci 2017; 17(1): 7-20. https://doi.org/10.1093/jisesa/iew098

Staunton KM, Nakamura A, Burwell CJ, Robson SKA, Williams SE. Elevational distribution of flightless ground beetles in the tropical rainforests of North-Eastern Australia. PLoS ONE 2016; 11: e0155826. https://doi.org/10.1371/journal.pone.0155826

Stone BC. The summit flora of Gunung Ulu Kali (Pahang, Malaysia). Federation of Museum Journal 1981; 26(1): 157-162.

Sunday, JM, Bates AE, Dulvy NK. Thermal tolerance and the global redistribution of animals. Nature Clim Change 2002: 2(9); 686–690. https://doi.org/10.1038/nclimate1539.

Williams DW, Liebhold AM. Climate change and the outbreak of two North American bark beetles. Agr Forest Entomol 2002; 4: 87-99.

Ya'cob Z, Takaoka H, Pramual P, Low VL, Sofian-Azirun M. Breeding habitat preference of preimaginal black flies (Diptera: Simuliidae) in Peninsular Malaysia. Acta Tropica 2016; 153: 57-63. doi: 10.1016/j.actatropica.2015.10.007.

Zuur AF, Ieno EN, Walker NJ, Saveliev AA, Smith GM. Mixed effects models and extensions in ecology with R. 2009; Springer-Verlag, New York.

Table 1 Sampling effort at Fraser's Hill (FH) and Genting Highland (GH) mountains. In both mountains, traps were set at four Altitudes: 500, 1,000, 1,500 and 1,800 m a.s.l., in ten stands in both mountains; stands are indicated with letters A-J (compare Fig. 1). The three right-hand columns show the number of traps (or groups of five pitfall traps) in each stand.

**Mountain**	**Altitude**	**Stand ID**	**Light**	**Malaise**	**Pitfall**
FH	500	A	-	-	1
FH	500	B	-	-	4
FH	500	C	1	-	-
FH	1,000	D	1	2	5
FH	1,000	E	1	-	-
FH	1,500	F	1	-	2
FH	1,500	G	-	1	1
FH	1,500	H	1	1	2
FH	1,800	I	1	2	1
FH	1,800	J	1	-	4
GH	500	A	-	1	-
GH	500	B	-	1	-
GH	500	C	2	-	5
GH	1,000	D	-	-	2
GH	1,000	E	2	2	3
GH	1,500	F	-	2	4
GH	1,500	G	-	-	1
GH	1,500	H	2	-	-
GH	1,800	I	2	1	4
GH	1,800	J	-	1	1

Table 2 GLMM summary for Altitude, using Mountain as a random factor, for the number of species and rarefaction standardized number of species for 5, 10 or 20 individuals. Results are shown for light, Malaise and pitfall trap data. Numbers in “%var” columns are percentages explained by a given variable; letters in ”Effect” columns show whether a given Altitude differed significantly ($p < 0.05$) and positively (pos) or negatively (neg) from the lowest Altitude (500 m a.s.l.) (in parentheses if marginally significant, i.e., $p < 0.1$) or whether this comparison was non-significant (ns; $p > 0.1$). For full output, see Appendix, Table 1.

	**LIGHT TRAPS**			**MALAISE TRAPS**			**PITFALL TRAPS**	
**Variable/Category**	**%var**	**Effect**		**%var**	**Effect**		**%var**	**Effect**
Number of species								
Mountain	7			4			53	
Altitude	41			78			11	
* 1,000 m a.s.l.		ns			neg			(pos)
* 1,500 m a.s.l.		ns			neg			ns
* 1,800 m a.s.l.		neg			neg			ns
Residuals	52			19			35	
Rarefied richness to 5 individuals								
Mountain	7			6			17	
Altitude	33			70			16	
* 1,000 m a.s.l.		ns			ns			pos
* 1,500 m a.s.l.		ns			ns			ns
* 1,800 m a.s.l.		(neg)			neg			ns
Residuals	60			24			67	
Rarefied richness to 10 individuals								
Mountain	15			7			26	
Altitude	45			72			11	
* 1,000 m a.s.l.		ns			ns			pos
* 1,500 m a.s.l.		ns			ns			ns
* 1,800 m a.s.l.		neg			neg			ns
Residuals	40			21			63	
Rarefied richness to 20 individuals								
Mountain	4			7			28	
Altitude	61			72			12	
* 1,000 m a.s.l.		ns			(neg)			pos
* 1,500 m a.s.l.		ns			neg			ns
* 1,800 m a.s.l.		neg			neg			ns
Residuals	35			21			59	

Table 3 Permutational multivariate ANOVA to assess the distinctiveness of beetle communities at different Altitudes (Alt 500, 1,000, 1,500 or 1,800 m a.s.l., or their logical combinations) as reflected by using three sampling methods (compare Fig. 2).

	**LIGHT TRAPS**							**MALAISE**							**PITFALL**					
**Variable**	**df**	**SS**	**MS**	**F**	**R^2**	**p**		**df**	**SS**	**MS**	**F**	**R^2**	**p**		**df**	**SS**	**MS**	**F**	**R^2**	**p**
Alt 500	1	0.47	0.47	1.28	0.09	0.0659		1	0.40	0.40	1.17	0.09	0.2717		1	0.48	0.48	1.58	0.04	0.0300
Residuals	13	4.75	0.37	0.91				12	4.12	0.34	0.91				38	11.55	0.30	0.96		
Alt 1000	1	0.47	0.47	1.29	0.09	0.1548		1	0.42	0.42	1.23	0.09	0.1528		1	0.97	0.97	3.34	0.08	0.0010
Residuals	13	4.75	0.37	0.91				12	4.10	0.34	0.91				38	11.06	0.29	0.92		
Alt 1500	1	0.53	0.53	1.47	0.10	0.0939		1	0.42	0.42	1.24	0.09	0.1299		1	0.46	0.46	1.51	0.04	0.0390
Residuals	13	4.69	0.36	0.90				12	4.10	0.34	0.91				38	11.57	0.30	0.96		
Total	14	5.22	1.00					13	4.52	1.00					39	12.03	1.00			
Alt 1800	1	1.36	1.36	4.56	0.26	0.0020		1	0.70	0.70	2.21	0.16	0.0030		1	0.76	0.76	2.56	0.06	0.0020
Residuals	13	3.87	0.30	0.74				12	3.82	0.32	0.84				38	11.27	0.30	0.94		
Alt 500-1000	1	0.67	0.67	1.93	0.13	0.0290		1	0.47	0.47	1.40	0.10	0.0699		1	0.97	0.97	3.33	0.08	0.0010
Residuals	13	4.55	0.35	0.87				12	4.05	0.34	0.90				38	11.06	0.29	0.92		
Alt 1000-1500	1	0.98	0.98	3.01	0.19	0.0010		1	0.68	0.68	2.13	0.15	0.0420		1	0.55	0.55	1.82	0.05	0.0070
Residuals	13	4.24	0.33	0.81				12	3.84	0.32	0.85				38	11.48	0.30	0.95		
Alt 1500-1800	1	0.67	0.67	1.93	0.13	0.0380		1	0.47	0.47	1.40	0.10	0.0619		1	0.97	0.97	3.33	0.08	0.0010
Residuals	13	4.55	0.35	0.87				12	4.05	0.34	0.90				38	11.06	0.29	0.92		
Alt 500-1500	1	1.36	1.36	4.56	0.26	0.0020		1	0.70	0.70	2.21	0.16	0.0050		1	0.76	0.76	2.56	0.06	0.0020
Residuals	13	3.87	0.30	0.74				12	3.82	0.32	0.84				38	11.27	0.30	0.94		
Alt 1000-1800	1	0.47	0.47	1.28	0.09	0.0699		1	0.40	0.40	1.17	0.09	0.2408		1	0.48	0.48	1.58	0.04	0.0210
Residuals	13	4.75	0.37	0.91				12	4.12	0.34	0.91				38	11.55	0.30	0.96		

Table 4 Significant ($p < 0.05$) indicators with $n > 4$ in the beetle data. LT = indicator of light traps; MA = indicator of Malaise traps; PT = indicator of pitfall traps; different Altitudes or ranges are shown with numbers (500, 1,000, 1,500, 1,800 m a. s. l.). Column IndVal shows indicator value for each taxon.

**Category**	**Species**	**IndVal**		**Category**	**Species**	**IndVal**
**Single-method indicators**				**Single-method indicators continued**		
LT 500	*Anomala* sp2	0.81		PT	*Harpalus* sp1	0.84
LT 500	*Anomala* sp4	0.76		PT	*Harpalus* sp2	0.91
LT 500	*Anomala* sp6	0.57		PT	*Hiletus* sp1	0.44
LT 500	*Apogonia* sp3	0.79		PT	*Inopeplus* sp1	0.63
LT 500	*Apogonia* sp5	0.79		PT	*Lebia* sp1	0.57
LT 500	*Cicindela* sp2	0.79		PT	*Omonadus* sp1	0.52
LT 500	Mordellidae A	0.80		PT	*Pentagonica* sp1	0.54
LT 500	Scarabeidae M	0.80		PT	Staphylinidae M	0.63
LT 500	Scarabeidae P	0.58		PT 500	*Pterostichus* sp2	0.87
LT 500-1000	*Epepeotes lateralis*	0.52		PT 500-1500	*Spinolyprops* A	0.53
LT 1000	*Apogonia* sp1	0.90		PT 500-1500	Staphylinidae C	0.61
LT 1000	*Cicindela* sp1	0.69		PT 1000	*Actiastes* sp1	0.71
LT 1000-1500	*Anomala* sp1	0.94		PT 1000	*Anotylus* sp2	0.86
LT 1000-1500	*Apogonia* sp2	0.96		PT 1000	*Bledius* sp1	0.78
LT 1500	Lampyridae E	0.70		PT 1000	*Lispinus* sp1	0.84
LT 1500	*Luciola* sp1	0.71		PT 1000	*Orphnebius* sp1	0.84
LT 1500-1800	*Altica* sp1	0.69		PT 1000	*Orphnebius* sp2	0.92
LT 1800	*Cleorina* sp	0.71		PT 1000	Passalidae A	0.44
LT 1800	*Hoplocerambyx spinicornis*	0.87		PT 1000	*Pityogenes* sp1	0.74
LT 1800	*Hydrovatus enigmaticus*	0.97		PT 1000	*Sunius* sp1	0.71
LT 1800	*Illeis* sp2	0.70		PT 1000-1800	*Oxylatus* sp1	0.57
MA 500	Aleocharinae sp1	0.84		PT 1500	*Hiletus* sp2	0.58
MA 500	*Anotylus* sp1	0.94		PT 1500	*Pterostichus* sp1	0.79
MA 500	*Aphthona* sp1	0.98		PT 1500-1800	*Paedarus* sp1	0.69
MA 500	*Brachypeplus* sp1	0.92		PT 1800	*Lebia* sp2	0.57

MA 500	*Brachypeplus* sp3	0.98		PT 1800	*Pterostichus* sp3	0.79
MA 500	*Bradymerus* sp2	0.99				
MA 500	*Epuraea* sp1	0.70		**Multiple-method indicators**		
MA 500	Galerucinae sp1	0.97		MA+LT 500	*Mulsanteus* sp1	0.60
MA 500	*Ischnosoma* sp1	0.95		MA+LT 500	Paederinae sp3	0.68
MA 500	*Lymantor* sp1	0.94		MA+LT 1000	*Sarmydus* sp1	0.71
MA 500	*Lymantor* sp2	0.91		MA+LT 1000-1500	*Strotocera* sp1	0.71
MA 500	*Lymantor* sp3	0.94		MA+LT 1500	Alticinae sp2	0.69
MA 500	*Sinoxylon* sp1	0.99		MA+LT 1500	*Anisandrus* sp1	0.68
MA 500	*Xyleborus* sp1	0.98		MA+LT 1500	*Brachypeplus* sp2	0.65
MA 500-1000	Aleocharinae sp2	0.58		MA+LT 1500	*Colaspoma* sp2	0.71
MA 1000	*Anomala* sp3	0.82		MA+LT 1500	Curculionidae A	0.70
MA 1000-1500	Alticinae sp1	0.70		MA+LT 1500	Meloidae A	0.71
MA 1500	Paederinae sp2	0.66		MA+LT 1500	*Nisotra* sp2	0.71
MA 1500-1800	*Xyleborus* sp2	0.71		MA+LT 1500	*Pityogenes* sp2	0.48
MA 1800	*Anomala* sp5	0.66		MA+PT 500	Aleocharinae sp3	0.62
MA 1800	*Apogonia* sp4	0.69				
MA 1800	*Lymantor* sp4	0.90				
MA 1800	*Xylothrips* sp1	0.68				

Figure captions

Fig. 1. Study location in Malaysia. Left: map of Malaysia, with major mountain regions shown with gray; the two squares show locations of Fraser's Hill (top right) and Genting Highland (bottom right) mountains. Right-hand graphs show locations of ten study stands in each study mountain (denoted with letters A-J; compare Table 1); black lines show major highways; Altitudes are indicated with numbers (500, 1,000, 1,500 or 1,800 m a.s.l.). In the bottom right-hand graph, the gray patch shows a forest reserve. (The figure is generated by us, which is similar but not identical to the original image and is therefore for illustrative purposes only).

Fig. 2. Rarefaction curves for the three trap types and the pooled sample. The end point of each curve indicates the trap-type specific or pooled (total) number of individuals.

Fig. 3. Average samples + SE of four measures of species richness for three sampling methods according to Altitude. Number of species, and rarefaction standardized richness based on 5, 10 and 20 individuals are shown. Note that the X axis length for pitfall trap samples is different.

Fig. 4. Rank abundance (mean + SE) of twenty most abundant species in each trap type at different Altitudes (500, 1,000, 1,500 and 1,800 m a.s.l.). Note that species are mostly different for each trap type, each sorted according to the rank order of the total number of individuals in that trap type. Grey columns show species that were shared between light and Malaise traps, and white columns show the shared species between pitfall, light and pitfall traps (one species). For species identities, see Appendix.

Fig. 5. NMDS plots for Malaysian beetle data, separate runs for three sampling methods (light, Malaise or pitfall traps) at different Altitudes (500 m = black squares, 1,000 m = red circles, 1,500

m = green up-triangles and 1,800 m = blue down-triangles). For statistical comparisons between Altitudes, see Table 3.

Fig. 6. MRT for sampling-method specific beetle data, using mountain (Fraser's Hill or Genting Highland) and Altitude (500, 1,000, 1,500 or 1,800 m a.s.l.) as explanatory variables. The column plots show relative abundance of each species captured using a given trap type, sorted according to the rank order of abundance of the total trap-type specific sample. Numbers in parentheses below each end branch show the number of samples falling into that branch.

Click here to access/download;Figure;Figure 2.jpg

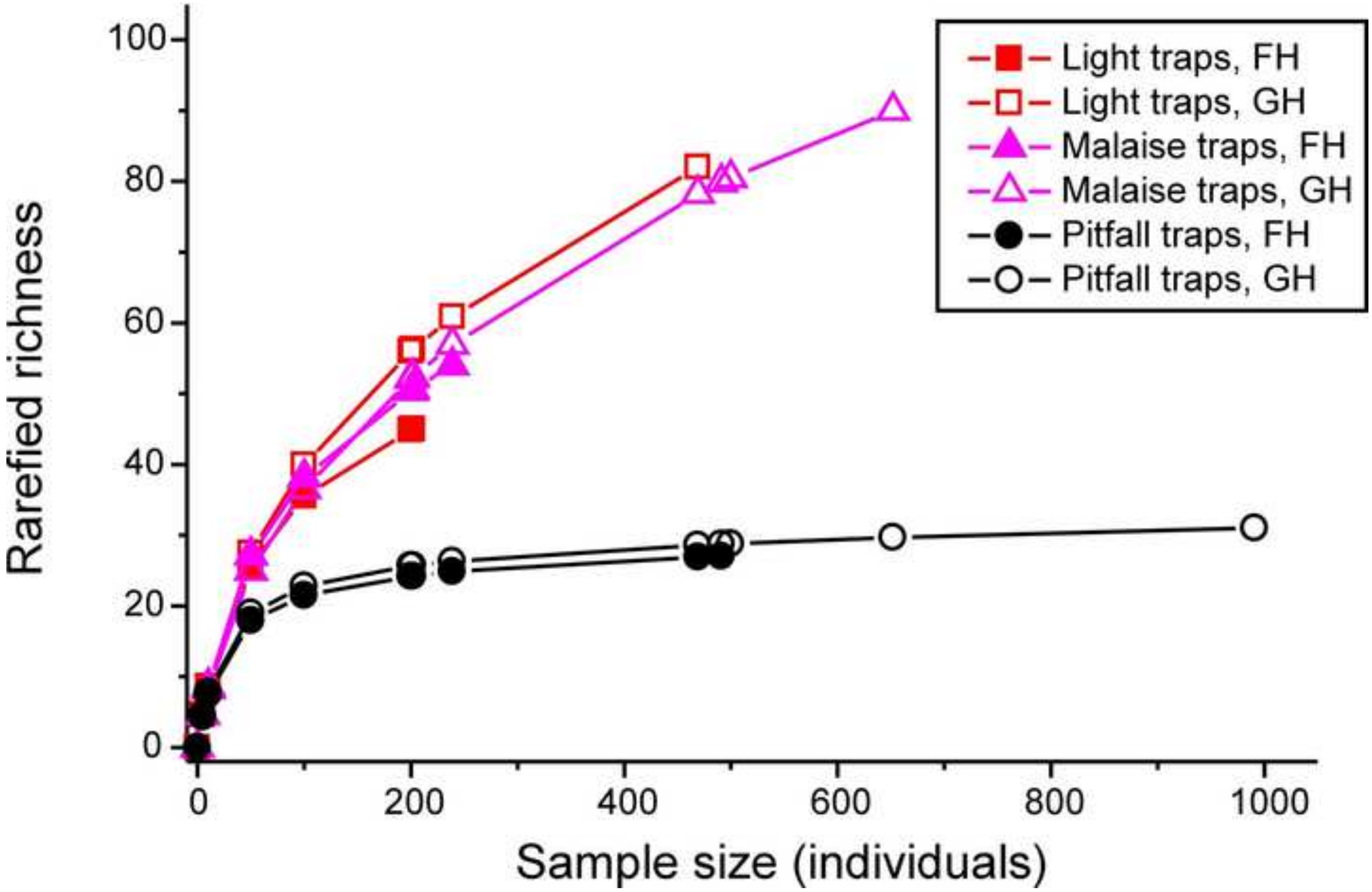

Figure 1

Click here to access/download;Figure;Fig 1.tif

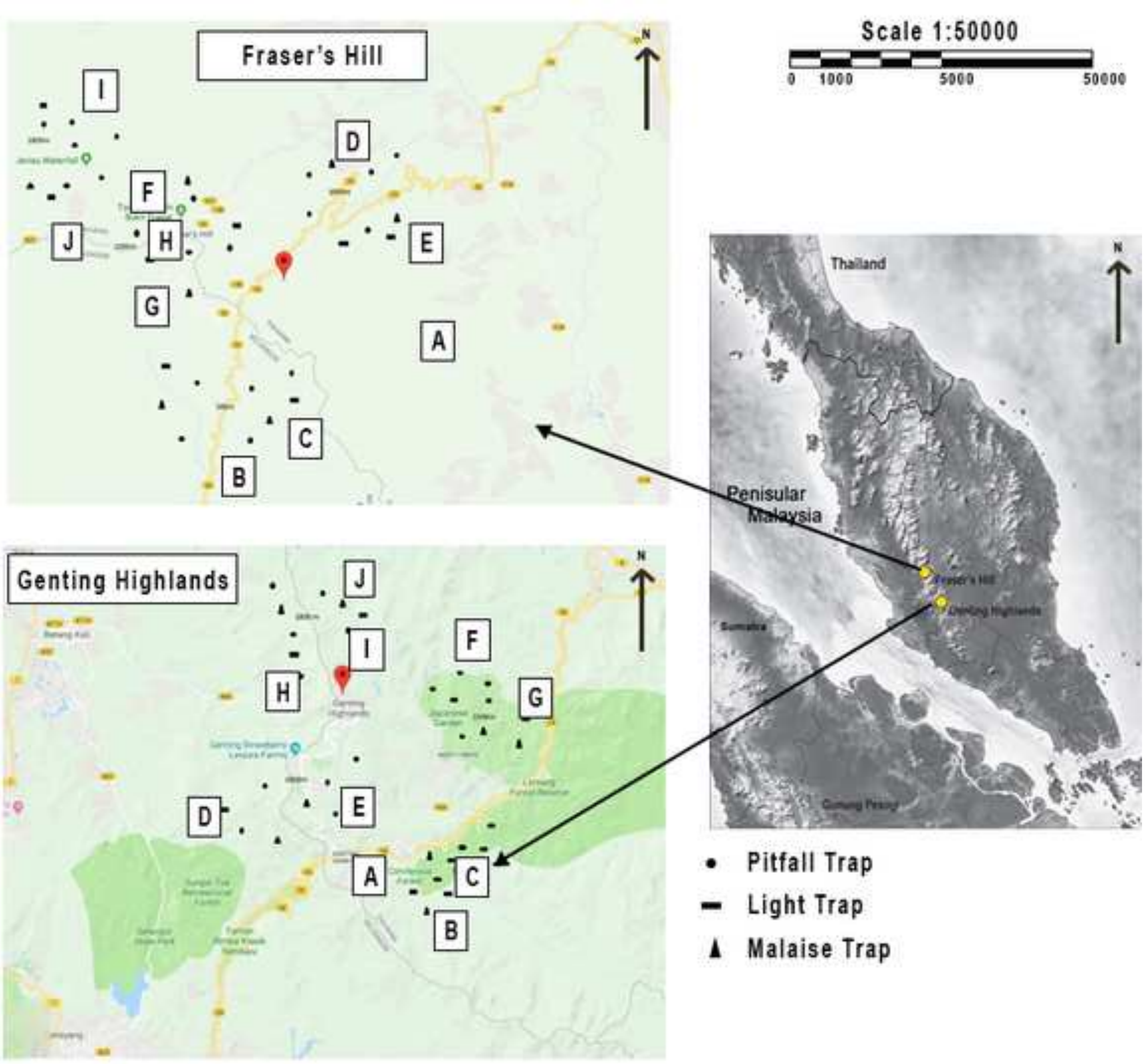

Click here to access/download
Supporting Information
Musthafa et al. Supplementary files.docx

Click here to access/download
Supporting Information - Compressed/ZIP File Archive
Supplementary Table S5 Musthafa et al..txt

Full Title:

The functioning of different beetle (Coleoptera) sampling methods across altitudinal gradients in Peninsular Malaysia

Short Title: Beetle sampling methods across altitudes in Malaysia

Muneeb M. Musthafa[1,2,3 *], Fauziah Abdullah[2,3,4] and Matti J. Koivula[5]

[1]Department of Biosystems Technology, Faculty of Technology, South Eastern University of Sri Lanka, University Park, Oluvil, #32360, Sri Lanka.

[2]Institute of Biological Science, Faculty of Science, University of Malaya, 50603, Kuala Lumpur, Malaysia.

[3]B513, Toxicology Lab, Institute of Postgraduate Studies, University Malaya, 50603, Kuala Lumpur, Malaysia.

[4]Center of Biotechnology in Agriculture, University Malaya, 50603, Kuala Lumpur, Malaysia

[5] Natural Resources Institute Finland (LUKE), P. O. Box 2, FI-00791 Helsinki, Finland.

* Corresponding author: muneeb@seu.ac.lk

Author contributions

Muneeb M. Musthafa: Initial research idea, fieldwork, data curation, manuscript writing, and technical editing.

Fauziah Abdullah: Conceptualization of the research and methodological design, manuscript writing.

Matti J. Koivula: Statistical analysis, manuscript writing, and technical editing.

Abstract

Biodiversity research relies largely on knowledge about species responses to environmental gradients, assessed using some commonly applied sampling method. However, the consistency of detected responses using different sampling methods, and thus the generality of findings, has seldom been assessed in tropical ecosystems. Hence, we studied the response consistency and indicator functioning of beetle assemblages in altitudinal gradients from two mountains in Malaysia, using Malaise, light, and pitfall traps. The data were analyzed using generalized linear mixed-effects models (GLMM), non-metric multidimensional scaling (NMDS), multivariate regression trees (MRT), and indicator species analysis (IndVal). We collected 198 morpho-species of beetles representing 32 families, with a total number of 3,052 individual beetles. The richness measures generally declined with increasing Altitude. The Mountains differed little in terms of light and Malaise trap data but differed remarkably in pitfall-trap data. Only light traps (but not the other trap types) distinguished high from middle or low altitudes in terms of beetle richness and assemblage composition~~, where t. he richness measures have declined with Altitude but only 1,800 m differed significantly from 500 m. We found (1) considerable differences in sample richness and composition between two mountains in pitfall but less so in light or Malaise traps; (2) light traps (but not the other trap types) distinguished high from middle or low altitudes in terms of beetle richness and assemblage composition; and (3)~~ The lower altitudes hosted about twice as many indicators as middle or high altitudes, and many species were trap-type specific in our data. These results suggest that the three sampling methods reflected the altitudinal gradient in different ways and the ~~that the~~ detection of community variation in the environment thus depends on the chosen sampling method~~, but and~~. However, also ~~on~~ the analytical approach appeared

important, ~~suggesting~~ further underlining the need to use multiple methods in environmental assessments.

Keywords: biodiversity, altitude, forest, light trap, Malaise trap, pitfall trap, tropical.

Introduction

Most hotspots for the global biodiversity can be found in tropical regions (Mittermeier et al. 2011) which are thus of central importance for conservation. Tropical species often have limited distributions, particularly those species that occupy higher altitudes at mountain slopes (Cox et al. 2016). The majority of the world's vertebrates, for instance, occupy mountain regions that cover only one-fourth of terrestrial landmass (excluding Antarctica; Rahbek et al. 2019). Such mountain species are often endemic to these regions, and also sometimes such that may not be able to move elsewhere if conditions turn unfavorable for them due to, for example, habitat destruction or climate change (Escobar et al., 2005; Elsen & Tingley 2015). Hence, studies on mountain species communities provide crucial information for global conservation efforts, in addition to these environments being particularly vulnerable to habitat degradation and alterations in landscape structure (Körner 2003; Rangwala & Miller 2012).

Community composition is largely determined by ~~the biotic and abiotic interactions of various species~~ the relative abundances of, and interactions among, its species, which contribute~~s~~ to ~~their relative shares, and interactions (biotic responses) largely determines~~ the general biodiversity response to environmental variation. Communities vary according to, among other aspects, latitude and altitude (Hooper et al. 2005; Ashton et al. 2015; Bouzan et al. 2015). Our understanding regarding this variation relies on some commonly assessed taxonomic groups and associated

sampling methods. Similarly, land-use planning or conservation decisions may be based on only a handful of well-known species or a single sampling method. Insects, for instance, are often sampled using light, Malaise, or pitfall traps (e.g., Grootaert et al. 2010). Environmental assessments, however, are often based on just one sampling method, which is assumed to produce a general biodiversity response, but this assumption has seldom been challenged.

Earlier research indicates that the three mentioned trap types differ in their efficiency in capturing specimens and species (e.g., Hansen 1988; Ulyshen et al. 2005; Devigne & De Biseau 2014) though the efficiency could vary with the composition of the local species community determined by, for example, altitude or habitat type. As different methods are known to capture partly different species (e.g., Missa et al. 2009), their use may, at least theoretically, result in different conclusions and hence applications in land use or conservation.

The objective of the present study is to evaluate the consistency of light, Malaise and pitfall trap samples in ~~relation to the~~reflecting an altitudinal gradient in tropical Malaysia. ~~The objective of the present study is methodological: we assess the consistency of light, Malaise, and pitfall trap samples in reflecting a simple environmental gradient, altitude~~. More specifically, we compare the three sampling methods across the altitudinal gradient from four viewpoints:

(1) Assemblage species richness;

(2) Assemblage species-compositional turnover;

(3) Assemblage composition; and

(4) Species associated with different combinations of sampling method and altitude

Materials and Methods

Study sites

The Titiwangsa mountain range dominates the landscape of Peninsular Malaysia. Within this range, we sampled beetles at Fraser's Hill (3°43’ N, 101°45’ E) and Genting Highland (3°25' N, 101°47' E) that are 95 km apart (Fig. 1). Fraser’s Hill mountain tops peak at between 1,000 and 1,800 m a.s.l., whereas the Genting Highland peaks at about 1,800 m a.s.l. In this region, wet and dry seasons cannot be differentiated, as the annual rainfall of 1,800–3,500 mm is distributed throughout the year (Cheong 2013; Ya’cob et al. 2016). ~~The c~~Collection permits for Fraser’s Hill and Genting Highlands were obtained from the Forest Department of Malaysia.

Fraser’s Hill, locally known as Bukit Fraser, is a well-preserved~~,~~ permanently~~-~~-protected nature reserve located at the Raub district of Pahang state. Fraser’s Hill has been developed as a hill station dating back in 1919 (Freeman 1999), where 90% of ~~the~~ 2,800-ha land area is covered by forests (Kiew 1998; Cheong 2013). Fraser’s Hill comprises residential areas, commercial complexes, community services, and recreational facilities. The forests mainly consist of tropical montane cloud forests. Lower montane forests – at about 500–1,200 m a.s.l. – are dominated by Fagaceae and Lauraceae trees, whereas upper montane forests – from about 1,200 to 1,800 m a.s.l. – are dominated by Coniferae, Ericaceae, and Myrtaceae trees (Perumal & Lo 1998).

Genting Highland is perhaps the most disturbed cloud forest in the Malaysian mountains. The summit is covered by amusement parks, casinos, and hotels (Peh et al. 2011). The highest peaks at Genting Highland reach 1,800 m a.s.l, where 96% of the total of 3,965 ha of land is still covered with mostly primary forests (Cheong 2013). Before Genting Highland became an entertainment site, it was an undisturbed forest that could be reached only via jungle trekking (Piggott 1977; Stone 1981).

Sampling methods

We sampled beetles using two Malaise traps, two light traps, and ~~25~~ 10 (5 groups of 2~~5~~) pitfall traps at each ~~altitudes~~altitude (500 m, 1,000 m, 1,500 m, and 1,800 m a.s.l.) at both mountain slopes. All the traps (or trap groups for pitfall traps) were set at least 150 m apart at a given altitude and were placed at least 150 m from the nearest main road. These roads and a river split the sampled forests into a total of twenty distinctive stands; each of these had at least one type of trap (Fig. 1, Table 1).

Malaise traps consisted of a nylon net connected to a collection jar, half filled with 70% ethanol and attached to a tree branch about one meter above the ground. Light traps had a mosquito net with attached 160-watt mercury bulb connected to a portable Honda EU10i generator. Pitfall traps were plastic cups (diameter 65 mm, depth 95 mm) partly filled with 70% ethanol and dug into the ground with the rim flush with the soil surface. We placed large dry leaves above each pitfall trap to protect them from litter and rain.

We sampled beetles once per month in October 2014, and March, June, and September 2015. At each collecting date, Malaise and pitfall traps were set for 24 hours, starting at 08:00 AM, and light traps operated from 18:00 to 23:30. In the latter, beetles were obtained manually from the traps using collection bottles and aspirators. We occasionally continued to use the light traps until the next morning at 06:00 AM but did not capture additional beetles.

In statistical analyses, we pooled the four periods for each trap (or trap group for pitfall traps) and consider each trap (or trap group for pitfall traps) as a replicate (initially 72; 4 altitudes and 2 mountains, each with 2 light and 2 Malaise, and 5 groups of pitfall traps).

Identification of specimens

We sorted, counted, and cross-checked all beetle specimens using available keys (Saito 2003; Mohamedsaid 2004; Naomi 2006; Schawaller 2006, 2012; Schimmel & Tarnawski 2007; Assing 2008; Hlaváč et al. 2011; Prathapan & Viraktamath 2011; Ryvkin 2011; Shi et al. 2013; Filippini et al. 2016; Anzaldo 2017; Makranczy 2017; Sasakawa et al. 2017). We confirmed the identification of difficult specimens at collections of the Wildlife Department of Malaysia, University of Malaya, National University of Malaysia, and Forestry Department of Malaysia. The identification of beetles to species is hampered by the lack of experts and species compilations. In the present study, the samples contained at least five species new to science, which will be described in later papers. Clearly, the often-broad taxonomic levels and the shortage of knowledge about the ecological traits of species must be acknowledged while interpreting results, as similar-looking species might be different in terms of, for example, life cycles, diets, abilities to disperse, and habitat requirements.

Statistical analysis

We had initially 72 samples (16 for light, 16 for Malaise, and 40 for pitfall traps (5 sets X 2 X 4 elevations)). Three samples, however, produced only 1-2 species and were excluded due to difficulty in calculating pair-wise dissimilarities and richness estimates (one light and two Malaise trap samples). Thus, we ran all analyses with 69 samples (Table 1). We ran the following analyses for the four viewpoints proposed above.

Assemblage richness:

To assess sampling-method dependent variation in species richness, we subjected the beetle data to a generalized linear mixed-effects model (GLMM; Zuur et al. 2009) to quantify variation

according to altitude (500, 1,000, 1,500, or 1,800 m a.s.l.). We ran the model separately for each sampling method (light, Malaise, or pitfall traps; hereafter "Method" for brevity) to be able to compare their similarity in responses to altitude. We subjected the observed number of species and richness estimates based on rarefaction standardization to 5, 10, and 20 individuals, to a GLMM with Altitude as a fixed factor and Mountain (Fraser's Hill or Genting Highland; Fig. 1, Table 1) as a random factor (to account for spatial autocorrelation), using lme4 (Bates et al. 2015). For full model outputs, see Supplementary Table S1.

For linear models it is important to assess the normality and homoskedasticity of model residuals, which we did in two ways. Firstly, we inspected Q-Q plots of GLMM residuals visually (car package; Fox & Weisberg 2018), confirmed by Wilk-Shapiro test for residual normality (Supplementary Figure S1 and Table S2). These did not indicate major departures from normality, except in the pitfall-trap data. Secondly, we ran Wilk-Shapiro test to check the normality, and Breusch-Pagan test to assess homoskedasticity, of ANOVA residuals (model Mountain + Altitude; Supplementary Table S3). These checks indicated the following issues: (i) heteroskedasticity in the number of species and non-normality in the rarefied richness to 5 individuals in the light-trap data; (ii) heteroskedasticity in the number of species in the Malaise-trap data; and (iii) non-normality in the rarefied richness standardized to 5 and 10 individuals in the pitfall-trap data (as for GLMM residuals above). Due to these deviations from normality, we reran the GLMM models (as in Table 2) using Robust LMM (robustlmm package; Koller 2016). Models run using biological data often contain outlier samples that may render residual distributions non-normal or heteroskedastic. Estimates from Robust LMM are little affected by such outliers, if the tuning parameter (k) is set at a low value; values approaching ∞ produce results similar to a normal GLMM. Here, we applied the smoothed Huber ($k = 1.345$, $s = 10$) function for fitting random

effects (Mountain) and variance component (Altitude), as recommended by Koller (2016) (Supplementary Table S4). As the relative magnitudes and directions of estimates in Robust LMM were very similar to the initial GLMM, we conclude that our GLMM results shown in Table 2 are robust.

~~To assess sampling-method dependent variation in species, we subjected the beetle data to a generalized linear mixed-effects model (GLMM; Zuur et al. 2009) to quantify variation according to altitude (500, 1,000, 1,500, or 1,800 m a.s.l.). We ran the model separately for each sampling method (light, Malaise, or pitfall traps; hereafter "Method" for brevity) to assess their similarity in responses to altitude. We subjected the observed species (number of species) and richness estimates based on rarefaction standardization to 5, 10, and 20 individuals, to a GLMM with altitude as a fixed factor and Mountain (Fraser's Hill or Genting Highland; Fig. 1, Table 1) as a random factor (to account for possible spatial autocorrelation) and assumed a Gaussian error distribution. We checked the normality of residuals using Q-Q plots (Supplementary Figure 1). These plots did not indicate major departures from normality, except in the pitfall trap data for the number of species and rarefied richness to 5 individuals. We attempted to solve these by square-root and log transforming the data, and by applying a quasi-Poisson error distribution on untransformed data (Supplementary Figure 2). The quasi-Poisson error distribution improved the model fit for the number of species but not for the rarefied richness. Nevertheless, as the rarefied richness to 10 and 20 individuals produced broadly similar results, we are confident that our initial analyses are robust.~~

Assemblage turnover:

To examine possible species turnover according to altitude and Method, we calculated averages and standard errors for 20 most abundant species, separately for each Altitude and Method. We

plotted these values according to Altitude, using the rank order of the abundances of species captured using each Method. We evaluated the community turnover, or distinctiveness of samples, between Altitudes using permutational multivariate ANOVA (the adonis function in R package vegan, with Mountain [Fraser's Hill or Genting Highland] as strata; Oksanen et al. 2018). Here, we only considered single, or combinations of subsequent, Altitudes. Thus, for example, a combination 500 + 1000 m was considered but not 500 + 1800 m. As a simple measure of turnover between Methods, we indicated in these plots species that were unique for a given Method, and those species that were shared with 1–2 other Methods. We confirmed this comparison of Methods using permutational multivariate ANOVA, as described above.

Assemblage composition:

To examine beetle assemblage composition across Altitudes and Methods, we used two analyses. Firstly, we used non-metric multidimensional scaling (NMDS; Borg & Groenen 2005) to assess variation in community composition, using the vegan package (Oksanen et al. 2018). We used Method-specific data sets by applying a Bray-Curtis dissimilarity matrix. We used the above-described permutational multivariate ANOVA for Altitudes as a confirmation of the NMDS result. Secondly, we subjected the Method-specific beetle data sets to multivariate regression tree analysis (MRT; De'ath 2002) based on the Bray-Curtis dissimilarity matrix, using the mvpart package (De'ath et al. 2014). We used altitude and, as our earlier research has indicated differences in beetle faunas between Fraser's Hill and Genting Highland (Musthafa et al. 2018), Mountain as explanatory variables. MRT identifies groups of samples as defined by explanatory variables (Mountain and Altitude) and is not restricted by non-linearities (De'ath 2002). We present the result as a tree of dichotomies, where each dichotomy is based on minimizing the dissimilarity of samples within each tree branch. We report the tree with the lowest cross-validated relative error, following

the 1-SE rule (Breiman et al. 2017). Cross-validated relative error provides a better estimate than relative error for the predictive accuracy of the MRT for a new dataset (De'ath 2002).

Species associations with Altitudes and Methods:

To detect species characteristic to particular combinations of Method and Altitudes, we calculated an indicator value (IndVal; Dufrêne & Legendre 1997, De Cáceres et al. 2010) for each species, based on all logical combinations of Method and Altitude. Here, we used the indicspecies package (De Cáceres & Jansen 2016) and allowed each Method to appear singly or jointly with 1–2 other Methods, whereas for Altitude we only considered single, or combinations of subsequent, Altitudes, as described above. We restricted the IndVal to species with a total sample of at least five individuals.

~~Analysis software:~~

~~We ran the analyses using R 3.6.1 software (R Core Team, 2019) with lme4 (Bates et al. 2015), vegan (Oksanen et al. 2018), mvpart (Therneau et al. 2014), indicspecies (De Cáceres & Jansen 2016), and car (Fox & Weisberg 2018) packages.~~

Results

Beetle richness according to Mountain and Altitude

We collected 198 morpho-species of beetles representing 32 families with a total number of 3052 individual beetles (Supplementary Table S5~~Supporting Information~~). Nine were identified to species, 143 to genus, and 43 to higher taxonomic levels. We refer to all these as “species” below for convenience. Regarding Method, we collected 107 species using light traps, 127 using Malaise,

and 45 using pitfall traps. A total of 135 species were represented by only one Method, whereas 45 had been captured with two and 18 with all three sampling methods. Altogether 98 species were singletons or doubletons, whereas 84 were represented by at least five individuals (Supplementary ~~materials~~ ~~Appendix~~ Table S~~1~~5). Species accumulation curves based on rarefaction suggested that pitfall traps had captured nearly all potential species at about 200 individuals at both Mountains, whereas the accumulation curves for light and Malaise traps were still very steep at about 400-500 individuals (Fig. 2).

The GLMM performed rather similarly in terms of the four measures of richness, but the three Methods produced partly different results (Table 2, Fig. 2; Supplementary ~~materials,~~ Table S1). The Mountains differed little in terms of light and Malaise trap data but remarkably in pitfall-trap data. The richness measures based on light traps declined with Altitude but only 1,800 m differed significantly from 500 m. The decline with Altitude occurred also in Malaise traps so that all higher Altitudes differed significantly from 500 m. Richness measures based on pitfall traps, on the other hand, peaked at 1,000 m (Table 2, Fig. 2).

Species turnover

The rank-abundance plots of twenty most abundant species (Fig. 4) reflected relatively high similarity between light and Malaise traps, with nine of the 20 most numerous species being shared (grey columns), whereas only one of the 20 dominant species – *Pityogenes* sp1 – in pitfall-trap samples was shared with light- and Malaise-trap samples (white columns). For species identities in this graph, see Supplementary Table S6~~Appendix~~ ~~Fig. S1~~. Permutational multivariate ANOVA indicated that also light and Malaise traps were compositionally different; for light vs. Malaise traps, F = 3.21, R2 = 0.11, p = 0.0010; for light vs. pitfall traps F = 9.41, R2 = 0.15, p = 0.0010; and for Malaise vs. pitfall traps F = 10.48, R2 = 0.17, p = 0.0010. Moreover, the plots suggested

more abundance or occurrence changes with Altitude in Malaise and light traps, whereas the plots varied less and more erratically in pitfall-trap samples (Fig. 4). Another striking pattern in light-trap samples (Fig. 5, left) was the lack of the 12 most abundant species at 1,800 m. Only 9% of light-trap species and 17% of Malaise-trap species were found in both 500 m and 1,800 m, whereas the percentage was 63 for pitfall-trap samples.

Permutational multivariate ANOVA confirmed compositional changes with Altitude, particularly at 1,800 m, but the three Methods showed different patterns in this respect (Table 3). In light traps, only 1,800 m differed significantly from the other Altitudes; however, all except one combination of 2–3 Altitudes also differed significantly from the rest of the Altitudes. The patterns were similar for Malaise traps, except that different combinations of 2–3 Altitudes differed less commonly from the rest of the Altitudes. Regarding pitfall traps, then, all Altitudes and their combinations (except for 1,000–1,500 m) had distinctive assemblages (Table 3).

Assemblage composition according to Method and Altitude

According to NMDS, samples in light and Malaise traps distinguished 1,800 m from the three lower altitudes, whereas pitfall trap samples formed two sample clusters, of which only the other distinguished clearly between Altitudes (Fig. 6). Thus, in pitfall-trap samples, Altitude was reflected in Fraser's Hill but not in Genting Highland (on the left and right, respectively, in Fig. 5).

MRT for light-trap data consistently resulted in a one-dichotomy tree (Fig. 6). The only division, where 1,800 m was split from the rest of the Altitudes, explained 47% of the variation in light-trap data, the former being species poor according to branch-specific rank-abundance plots (Fig. 6). Malaise-trap data, on the other hand, suggested that the same dichotomy, based on Altitude, was apparent in Fraser's Hill but not in Genting Highland; again, 1,800 m had fewer species though

this was based on only two samples. This two-dichotomy tree explained 81% of the variation in the Malaise trap data (Fig. 6). Also, pitfall trap data suggested that Altitudes could be distinguished at Fraser's Hill but not at Genting highland; here, 1,500 and 1,800 m diverged from the two lower Altitudes that, in turn, were split in the third dichotomy (Fig. 6).

Species associated with different combinations of Method and Altitude

We found 83 indicator species for different combinations of Method and Altitude (Table 4). Thirteen species were commonly caught with two sampling methods (12 for light and Malaise traps, and one with Malaise and pitfall traps). Moreover, eight species indicated pitfall traps across the full altitudinal range (500-1,800 m), whereas we did not detect such altitude-independent indicators for the two other sampling methods. Light traps produced 12 indicators of low (500-1,000 m), six of the middle (1,000-1,500 m) and seven of high (1,500-1,800 m) Altitudes, whereas the respective numbers were 16, four, and six for Malaise and ten, eleven and five for pitfall traps (Table 4). Moreover, three species occurred in pitfall traps across a wider Altitudinal range: two species were found at 500-1,500 m and one at 1,000-1,800 m.

Discussion

Beetle species turnover between methods and across the Altitudinal gradient

We found that light and Malaise trap samples shared about half of the abundant species, whereas pitfall trap samples were distinctive in this respect. Light and Malaise traps may have attracted a shared pool of species that flies actively, whereas pitfall traps capture mostly ground dwellers that seldom fall into the two other types of traps (e.g., Missa et al. 2009). This finding suggests that, at least in the study region, the variation in beetle communities caused by altitude or climatic factors

may be better captured if pitfall traps are used together with either light or Malaise traps, whereas a combination of light and Malaise traps may not be equally efficient.

At 1,800 m light (but not Malaise or pitfall) trap samples lost all 12 most abundant species of the total light-trap sample. This loss might have resulted from the captured species pool being more sensitive to altitude-associated changes in abiotic or biotic conditions. Studies comparing climatic conditions associated with altitudinal gradients may provide cues on species responses to the global climate change (Chen et al. 2009; Ashton et al. 2015, 2016; Musthafa et al. 2018; Musthafa & Abdullah 2019); perhaps many of the lower- altitudinal species eventually become more common at 1,800 m following climate change. However, some of the 12 “lost” species were still captured at 1,800 m using Malaise traps, so another explanation may be trap functioning. Perhaps light or wind conditions – or some other factors related to, for example, vegetation or moisture – were different at the highest altitudes, which might, in turn, have impacted the visibility of light traps or flight activity of many species (Mathews & Mathews 1971; McGeachie 1989; Sheikh et al. 2016; Nowinszky & Puskás 2017; Skvarla & Dowling 2017). It would be important to continue beetle monitoring at these mountain tops to see whether the altitudinal distributions of species indeed change with climate and whether the currently dominant mountain-top species persist or disappear.

The relative similarity of rank-abundance plots at different altitudes, particularly for pitfall-trap samples, may have occurred because many dominant species are adapted to a wide range of altitudes (e.g., Nunes et al. 2016) and/or associated temperature or moisture conditions (Gilchrist, 1995; Saavedra et al., 2013). Pitfall traps capture mostly ground dwellers, and field-layer vegetation or soil conditions may thus have kept conditions relatively constant across the altitudinal gradient for these species. Also, climatic factors such as temperature, rainfall, relative

~~humidity, wind velocity, and the amount of light may not have changed uniformly with increasing altitude in our study sites, but the possible altitudinal effect (changes in temperature, atmospheric oxygen, etc.) might have been compensated by, for example, habitat type or topography (Adam & Enning 1996; Adam et al. 2011).~~ These factors might decrease variation in micro-climate (McCain & Grytnes 2001), perhaps through the sheltering effect of forest trees (Barnes et al. 1997).

Beetle community structure across the altitudinal gradient using different methods

Our community analyses suggest that the overall beetle community varied remarkably according to altitude, but the magnitude of this response depended on geographic location (the two mountains) and captured species subset (sampling method). Thus, according to NMDS and MRT, light traps distinguished 1,800 m from lower altitudes, whereas Malaise and pitfall traps reflected primarily differences between the two mountains and only secondarily altitudinal variation. Moreover, and only at Fraser's Hill, Malaise traps distinguished 1,800 m from the lower altitudes, and pitfall traps distinguished 500, 1,000, and 1,500-1,800 m. ~~However, altitude might partly be a proxy of other environmental variables, such that were not assessed in this study. For instance, the lower (500 and 1,000 m) and higher (1,500 and 1,800 m) altitudes represented different forest types, the former being mixed deciduous and the latter being mixed conifer-heath-myrtle forests.~~

Indicator species for sampling methods and altitude

Pitfall traps produced eight indicator species that were common at all altitudes, whereas the other sampling methods did not produce such altitude generalists (for identities of these species, see Table 4). This finding is in line with our rank-abundance plots (Fig. 4). Moreover, all sampling methods produced at least some indicators of low (500–1,000 m), intermediate (1,000–1,500 m), and/or high altitudes (1,500–1,800 m), suggesting potential for each method to reflect altitude and

thus potentially global changes in climate or land use. Important from a climate perspective, we found nine significant indicators of 1,800 m (four using light, four using Malaise, and one using pitfall traps). Species adapted to these highest mountain tops may face difficulty in survival with predicted climate warming (e.g., Lenoir et al. 2008; Pickering et al. 2008; Dirnböck et al. 2011; Elsen et al., 2020) unless they can adapt or disperse elsewhere. Good dispersal ability of beetles allows them to rapidly move towards higher altitudes with warmer temperature (Bale et al., 2002; Williams & Liebhold, 2002). Moreover, beetles may lengthen their flight-activity period and increase the number of generations produced per year to compensate with changing climate, most notably temperature (Menéndez, 2007; Altermatt, 2010). Moreover, thermal adaptability of species could regulate the distributions and local adaptations of beetles across altitudinal gradients, since temperature intensely influences their flight activity (Calosi et al., 2010; Sunday et al., 2012; García-Robledo et al., 2016). ~~For most Malaysian beetle species, the level of knowledge about such traits is low.~~ In South Korea, for example, ~~Though~~ *Onthophagus* beetles (Coleoptera: Scarabaeidae) ~~in South Korea~~ have been predicted to move to higher altitudes with increasing ambient temperature (Kwon et al. 2015). ~~European studies suggest that t~~The dispersal ability is high also for many European Curculionidae (Bouget et al., 2015) and dung beetles (Gómez et al., 2020). For most Malaysian beetle species, however, the level of knowledge about such traits is low. ~~Moreover~~Nevertheless, some of the species in the present data ~~can be categorized as~~are apparently specialists of narrow temperature ranges, such as *Orphnebius* ~~species~~ (Staphylinidae)~~,~~ and *Rhadinomerus* species (Curculionidae), whereas *Brachypeplus* species may be considered a generalist in terms of temperature conditions ~~of~~ in the studied mountain slopes (Musthafa, 2018). On the other hand, *Inopeplus* (Salpingidae), *Macrotomoderus* (Anthicidae), Paederinae (Staphylinidae) and *Pentagonica* species (Carabidae) are ~~considered~~ temperature specialists ~~and~~

~~only~~whereas *Stenus* species (Staphylinidae) can be considered ~~as~~ a temperature-range generalist (Musthafa, 2018).

The precautionary principle calls for mitigation of climatic or land-use alterations at high altitudes (Gallou et al. 2017). In our study area, for instance, the two mountains hosted very different beetle communities, which perhaps partly reflects different intensities of land use. The studied sampling methods ~~probably~~ differ in costs, required labor, relative capturing efficiency, and captured species pool. The differences~~,~~ reported here~~,~~ ~~nevertheless~~ suggest that the use of more than one location and several sampling methods are desirable in environmental assessments (Lamarre et al. 2012; Devigne & De Biseau 2014; Abdullah & Musthafa 2019).

Research caveats

Taxonomic constraints may have affected our results to some extent, as most taxa had been identified to family or genus levels only. Thus, two individuals of a given taxon being captured using two methods or at different altitudes might in reality be two different species. However, this possibility rather masks than exaggerates the true community responses. We are therefore confident that the sampling methods and altitudes truly differed in beetle communities, but the differences would have been more pronounced had we been able to identify everything to species.

Conclusions

The location of the present study, Malaysia, belongs to the global biodiversity hotspot of Sundaland, yet little is known about the invertebrate diversity of its mountains (Myers et al. 2000, Doll et al. 2014). ~~Our data contribute to fulfilling this knowledge gap. However, our~~~~This study~~ ~~r~~Results of our study are ~~also~~ applicable to tropical species conservation: they provide evidence for adaptations of many species to particular altitudes and, more importantly, differences in beetle

samples between collecting methods. The three sampling methods reflected the altitudinal gradient in different ways. This, in turn, calls for caution in interpretations of data based on only one method and thus on a narrow range of species life histories. Whenever possible, ~~we recommend the use of~~ we strongly recommend multiple collecting methods ~~should be used~~ in environmental impact assessments on biodiversity.~~We, therefore, recommend the use of multiple collecting methods in environmental impact assessments on biodiversity, whenever possible~~. This is particularly important in land-use or political decision making, which should ideally be based on a holistic picture of biodiversity to avoid unwanted species losses or changes in ecosystem functioning.

Acknowledgments

We thank Mohd Shukri Mohd Sabri and Davindram A/L Rajendram for assistance in beetle sampling. We also acknowledge the Universiti Kebangsaan Malaysia and Forestry Department of Malaysia for supporting this study, and Marcelo Bruno Pessôa and an anonymous reviewer for constructive comments.

References

Abdullah F, Musthafa MM. Beetle abundance, diversity and species richness of Pulau Pangkor, Perak, Malaysia. Malaysia Forester 2019; 82: 247-270.

~~Adam JH, Enning FL. Altitudinal zonation of tropical rain forest at Danum Valley Research Centre, Sabah, Malaysia. Malaysian J Sci 1996; 17A: 25-35.~~

Adam JH, Ming LK, Juhari MAA, Jaluluddin MA, Idris WMR, Othman AR, Tarmiddzi SNA. Cluster Analysis of Submontane Forest along Western Slope of Frasers' Hill Research Centre in Raub District, Malaysia. Bangladesh J Bot 2011; 40: 121-132.

Altermatt F. Climatic warming increases voltinism in European butterflies and moths. Proc Biol Sci. 2010; 277(1685):1281-1287. doi:10.1098/rspb.2009.1910.

Anzaldo SS. Review of the genera of Conoderinae (Coleoptera, Curculionidae) from North America, Central America, and the Caribbean. ZooKeys 2017; 683: 51-138. https://doi.org/10.3897/zookeys.683.12080.

Ashton LA, Nakamura A, Basset Y, Burwell CJ, Cao M, Eastwood R, et al. Vertical stratification of moths across elevation and latitude. J Biogeogr 2016; 43: 59-69. https://doi.org/10.1111/jbi.12616

Ashton LA, Odell EH, Burwell CJ, Maunsell SC, Nakamura A, McDonald WJF, Kitching RL. Altitudinal patterns of moth diversity in tropical and subtropical Australian rainforests. *Austral Ecol* 2015; 41: 197-208. https://doi.org/10.1111/aec.12309.

Assing V. Four new species and additional records of Palaearctic *Sunius*, with two new synonymies (Coleoptera: Staphylinidae: Paederinae). *Beiträge zur èntomologie* 2008; 58: 455-470. https://doi.org/10.21248/contrib.entomol.58.2.455-470.

Bale JS, Masters GJ, Hodkinson ID, Caroline A, Martijn BT, Valerie KB, … John BW. Herbivory in global climate change research: Direct effects of rising temperature on insect herbivores. Global Change Biology 2002; 8: 1-16. https://doi.org/10.1046/j.1365-2486.2002.00451.x

Baniya CB, Solhøy T, Gauslaa Y, Palmer MW. The elevation gradient of lichen species richness in Nepal. The Lichenologist 2010; 42: 83-96. https://doi.org/10.1017/S0024282909008627

Barnes BV, Zak DR, Denton SR, Spurr SH. Forest ecology. 4th ed. John Wiley & Sons. 1999.

Barzani GM, Sahibin AR, Adam JH, Zulfahmi AR. The physical environment. Bukit Fraser crown of the Titiwangsa Range. In: Latiff, A., Maimon, A., Norhayati, A. & Adam, J.H., editors. Bukit Fraser Research Centre, Universiti Kebangsaan Malaysia & Fraser's Hill Development Corporation, Fraser's Hill Development Corporation, Raub, Malaysia; 2009. pp 9-16.

Bouget C, Brin A, Tellez D, et al. Intraspecific variations in dispersal ability of saproxylic beetles in fragmented forest patches. Oecologia 2015; 177: 911–920. https://doi.org/10.1007/s00442-014-3162-9

Bouzan AM, Flinte V, Macedo MV, Monteiro RF. Elevation and temporal distributions of Chrysomelidae in southeast Brazil with emphasis on the Galerucinae. ZooKeys 2015; 547: 103-117. https://doi.org/10.3897/zookeys.547.9723

Brehm G, Colwell RK. Kluge J. The role of environment and mid-domain effect on moth species richness along a tropical elevational gradient. Glob Ecol Biogeogr 2007; 16: 205-219. https://doi.org/10.1111/j.1466-8238.2006.00281.x

Bates D, Maechler M, Bolker B, Walker S. Fitting linear mixed effects models using lme4. J Stat Softw 2015; 67: 1-48. http://dx.doi.org/10.18637/jss.v067.i01

Borg I, Groenen P. Modern multidimensional scaling: theory and applications. 2nd ed. Springer-Verlag, New York. 2005

Breiman L, Friedman JH, Olshen RA, Stone CG. Classification and regression trees. CRC Press, Boca Raton, Florida. 358 pp. 1998.

Calosi P, Bilton DT, Spicer JI, Votier SC, Atfield A. What determines a species' geographical range? Thermal biology and latitudinal range size relationships in European diving beetles (Coleoptera: Dytiscidae). J Anim Ecol 2010; 79: 194–204. DOI: 10.1111/j.1365-2656.2009.01611.x.

Chao A, Jost, L. Coverage-based rarefaction and extrapolation: standardizing samples by completeness rather than size. Ecol 2012; 93(12): 2533–2547.

~~Chen IC, Shiu HJ, Benedick S, Holloway JD, Chey VK, Barlow HS, Hill JK, Thomas CD. Elevation increases in moth assemblages over 42 years on a tropical mountain. PNAS 2009; 106: 1479-1483. https://doi.org/10.1073/pnas.0809320106~~

Cheong C. Fraser's Hill - A Lush Highland Hideaway. WWF Malaysia, Petaling Jaya. 2013.

Cox CB, Moore PD, Ladle R. Biogeography: an ecological and evolutionary approach. Wiley Blackwell. 2016.

De Cáceres M, Legendre P, Moretti M. Improving indicator species analysis by combining groups of sites. Oikos 2010; 119: 1674-1684. DOI: 10.1111/j.1600-0706.2010.18334.x

De Cáceres M, Jansen F. Package Indicspecies. 2016. URL https://CRAN.R-project.org/package=indicspecies.

De'ath G. Multivariate regression trees: a new technique for modeling species–environment relationships. Ecol 2002; 83(4): 1105-1117. https://doi.org/10.2307/3071917

De'ath G, Therneau TM, Atkinson B, Ripley B, Oksanen J. mvpart: Multivariate partitioning. 2014. URL https://mran.microsoft.com/snapshot/2014-12-11/web/packages/mvpart/index.html

Devigne C, De Biseau J-C. Urban ecology: comparison of the effectiveness of five traps commonly used to study the biodiversity of flying insects. Biodivers J 2014; 5: 165-174.

Doll HM, Butod E, Harrison RD, Fletcher C, Kassim AR, Ibrahim S, Potts MD. Environmental and geographic factors driving dung beetle (Coleoptera: Scarabaeidae: Scarabaeinae) diversity in the dipterocarp forests of Peninsular Malaysia. Raffles Bull Zool 2014; 62: 549-560.

Dufrêne M, Legendre P. Species assemblages and indicator species: the need for a flexible asymmetrical approach. Ecol Monogr 1997; 67(3): 345-366. https://doi.org/10.1890/0012-9615(1997)067[0345:SAAIST]2.0.CO;2

Dirnböck T, Essl F, Rabitsch W. Disproportional risk for habitat loss of high-altitude endemic species under climate change. Glob Chang Biol 2011; 17: 990-996. https://doi.org/10.1111/j.1365-2486.2010.02266.x

Elsen PR, Tingley MW. Global mountain topography and the fate of montane species under climate change. Nat Clim Chang 2015; 5: 772-776. https://doi.org/10.1038/nclimate2656

Elsen, P.R., Monahan, W.B. &, Merenlender, A.M. Topography and human pressure in mountain ranges alter expected species responses to climate change. Nat Commun 2020; 11: 1974. https://doi.org/10.1038/s41467-020-15881-x

Escobar F, Lobo JM, Halffter G. Altitudinal variation of dung beetle assemblages in the Colombian Andes. Glob Ecol Biogeogr 2005; 14: 337-347. https://doi.org/10.1111/j.1466-822X.2005.00161.x

Filippini V, Micó E, Galante E. Checklist and identification key of Anomalini (Coleoptera, Scarabaeidae, Rutelinae) of Costa Rica. ZooKeys 2016; 621: 63-136. https://doi.org/10.3897/zookeys.621.7565

Fox J, Weisberg S. An R companion to applied regression. 3rd edition. Sage Publications Inc., California. 2019.

Gallou A, Baillet Y, Ficetola GF, Despres L. Elevational gradient and human effects on butterfly species richness in the French Alps. Ecol Evol 2017; 7(11): 1-10. https://doi.org/10.1002/ece3.2803.

García-Robledo C, Kuprewicz EK, Staines CL, Erwin TL, Kress WJ. Limited tolerance by insects to high temperatures across tropical elevational gradients and the implications of global warming for extinction. PNAS 2016: 113; 680-685. doi: 10.1073/pnas.1507681113

Gilchrist GW. -Specialists and generalists in changing environments. I. Fitness landscapes of thermal sensitivity. Am Nat 1995; 146: 252-270.

Gómez VCG, Verdú JR, Zurita GA. Thermal niche helps to explain the ability of dung beetles to exploit disturbed habitats. Sci Rep 2020; 10: 13364. https://doi.org/10.1038/s41598-020-70284-8

Hlaváč P, Newton AF, Maruyama M. World catalogue of the species of the tribe Lomechusini (Staphylinidae: Aleocharinae). Zootaxa 2011; 3075: 1-151.

Hooper DU, Chapin FS III, Ewel JJ, Hector A, Inchausti P, et al. Effects of biodiversity on ecosystem functioning: a consensus of current knowledge. Ecol Monogr 2005; 75: 3-35. https://doi.org/10.1890/04-0922.

Kessler M. Elevational gradients in species richness and endemism of selected plant groups in the central Bolivian Andes. Plant Ecol 2000; 149: 181-193.

Kiew R. The seed plant flora of Fraser's Hill, Peninsular Malaysia: With special reference to its conservation status. Research pamphlet No. 21. Forest Research Institute of Malaysia (FRIM), Kuala Lumpur, Malaysia. 1998.

Kohler T, Wehrli A, Jurek M. Mountains and climate change: A global concern. Sustainable Mountain Development Series. Bern, Switzerland, Centre for Development and Environment (CDE), Swiss Agency for Development and Cooperation (SDC) and Geographica Bernensia. 2014.

Koivula MJ, Chmaberlain DE, et al. Breeding bird species diversity across gradients of land use from forest to agriculture in Europe. Ecog~~eg~~graphy 2018; 41: 1331-1344. https://doi.org/10.1111/ecog.03295

Koller M. robustlmm: An R Package for Robust Estimation of Linear Mixed-Effects Models. Journal of Statistical Software 2016; 75: 1–24. https://doi: 10.18637/jss

Körner C. Alpine plant life. Functional plant ecology of high mountain ecosystems. 2nd edition. Springer, Berlin. 2003.

Kumar A, Longino JT, Colwell RK, O'Donnell S. Elevational patterns of diversity and abundance of eusocial paper wasps (Vespidae) in Costa Rica. Biotropica 2009; 41, 338-346. https://doi.org/10.1111/j.1744-7429.2008.00483.x.

Kwon T-S, Lee CM, Kim S-S. Prediction of abundance of beetles according to climate warming in South Korea. J Asia Pac Biodivers 2015; 8: 7-30. https://doi.org/10.1016/j.japb.2015.01.001.

Laliberte E, Wells JA, DeClerck F… Mayfield, MM. Land-use intensification reduces functional redundancy and response diversity in plant communities. Ecol Lett 2009; 13: 76-86. https://doi.org/10.1111/j.1461-0248.2009.01403.x

Lamarre GPA, Molto Q, Fine PVA, Baraloto C. A comparison of two common flight interception traps to survey tropical arthropods. ZooKeys 2012; 216: 43-55. https://doi.org/10.3897/zookeys.216.3332

Lenoir J, Gegout JC, Marquet PA, de Ruffray P, Brisse H. A significant upward shift in plant species optimum elevation during the 20th century. Science 2008; 320: 1768-1771. DOI: 10.1126/science.1156831.

Makranczy G. Review of the *Anotylus* cimicoides species group (Coleoptera: Staphylinidae: Oxytelinae). Acta Zoolog Academiae Scientiarum Hung 2017; 63: 143-262. DOI: 10.17109/AZH.63.2.143.2017

Mathews RW., Mathews JR. The Malaise Trap: Its Utility and Potential for Sampling Insect Populations. *The Great Lakes Entomologist* 1971; 4(4): Article 4.

McCain CM, Grytnes JA. Elevation gradients in species richness. In Encyclopedia of Life Sciences (ELS). John Wiley & Sons Ltd., Chichester. 2000.

McGeachie WJ.. The effects of moonlight illuminance, temperature and wind speed on light-trap catches of moths. Bull Entomol Res 1989; 79: 185-192.

Menéndez R. How are insects responding to global warming? Tijdschrift voor Entomologie 2007; 150: 355-365.

Mittermeier RA, Turner WR, Larsen FW, Brooks TM, Gascon C. Global Biodiversity Conservation: The Critical Role of Hotspots. In: Zachos, F.E. & Habel, J.C. editors, Biodiversity Hotspots. Springer-Verlag, Berlin-Heidelberg. 2011. Pp. 3-22.

Mohamedsaid MS. Catalogue of the Malaysian Chrysomelidae (Insecta: Coleoptera). Pensoft Publishers, Bulgaria. 2004.

Musthafa, MM. A comparative study of beetle diversity at different elevations at Fraser's hill and Genting Highland. Ph.D. Thesis. University of Malaya. 2018.

~~Musthafa MM, Abdullah F. Coleoptera of Genting Highland, Malaysia: Species richness and diversity along the elevations. Arx de Misc Zoology 2019; 17: 45-66. DOI: 10.32800/amz.2019.17.0045.~~

Musthafa MM, Abdullah F, Sánchez‐Reyes UJ. Comparative study of spatial patterns and ecological niches of beetles in two Malaysian mountains elevation gradients. J Insect Conserv 2018; 22(5-6): 757-769. https://doi.org/10.1007/s10841-018-0099-z

Myers N, Mittermeier RA, Mittermeier CG, da Fonseca GAB, Kent J. Biodiversity hotspots for conservation priorities. Nature 2000; 403: 853-858.

Naomi S. Taxonomic revision of the genus *Stenus* Latreille, 1797 (Coleoptera, Staphylinidae, Steninae) of Japan: Species group of S. indubius Sharp. Jpn J Syst Entomol 2006; 12: 39-120.

Newbold T, et al. Global effects of land use on local terrestrial biodiversity. Nature 2015; 520: 45-50. https://doi.org/10.1038/nature14324

Nowinszky L, Puskás J. Light-Trap Catch of Insects in Connection with Environmental Factors. In: Vonnie D.C. Shields, editors, *Biological Control of Pest and Vector Insects*, InTech Publishers. 2017. pp. 97-117.

Nunes CA, Braga RF, Figueira JEC, Neves FS, Fernandes GW. Dung beetles along a tropical altitudinal gradient: environmental filtering on taxonomic and functional diversity. PLoS ONE 2016; 11: e0157442. https://doi.org/10.1371/journal.pone.0157442

Oksanen J, Blanchet FG, Friendly M, Kindt R, Legendre P, McGlinn D, et al. Package vegan. Community Ecology Package. 2018. URL https://cran.r-project.org, https://github.com/vegandevs/vegan

Peh KS-H, Soh MCK, Sodhi NS, Laurance WF, Ong DF, Clements R. Up in the clouds: Is sustainable use of tropical montane cloud forests possible in Malaysia? Bioscience 2011; 61: 27–38. https://doi.org/10.1525/bio.2011.61.1.8

Perumal B, Lo FSJ. Recorded mountain trees of Peninsular Malaysia - a checklist. World Wildlife Fund Malaysia, Kuala Lumpur, Malaysia. 1998.

Pickering C, Hill W, Green K. Vascular plant diversity and climate change in the alpine zone of the Snowy Mountains, Australia. Biodivers Conserv 2008; 17: 1627-1644. https://doi.org/10.1007/s10531-008-9371-y

Piggott AG. The ferns of Gunung Ulu Kali. Garden Bulletin of Singapore 1977; 30: 31-43.

Prathapan KD, Viraktamath CA. A new species of *Longitarsus* Latreille, 1829 (Coleoptera, Chrysomelidae, Galerucinae) pupating inside stem aerenchyma of the hydrophyte host from the Oriental Region. ZooKeys 2011; 87: 1-10. doi: 10.3897/zookeys.87.1294

R Core Team. R: A language and environment for statistical computing. R Foundation for Statistical Computing, Vienna, Austria. 2021. URL https://www.R-project.org/

Rahbek C, Borregaard MK, Colwell RK, Dalsgaard B, Holt BG, Morueta-Holme N, ... Fjeldsa J. Humboldt's enigma: What causes global patterns of mountain biodiversity? Science 2019; 365(6458): 1108-1113. https://doi.org/10.1126/science.aax0149.

Rangwala I, Miller JR. Climate change in mountains: a review of elevation-dependent warming and its possible causes. Clim Change 2012; 114: 527-547. https://doi.org/10.1007/s10584-012-0419-3

Ryvkin AB. Contributions to the knowledge of *Stenus* (Nestus) species of the *Crassus* group (Insecta: Coleoptera: Staphylinidae: Steninae). Four new species from the Russian Far East with taxonomic notes. Balt J Coleopterol 2011; 11: 57-72.

Saavedra S, Rohr RP, Dakos V, Bascompte J. Estimating the tolerance of species to the effects of global environmental change. Nat Commun 2013; 4: 2350. https://doi.org/10.1038/ncomms3350

Saito M. A New Species of the genus *Tomoderus* (Coleoptera, Anthicidae) from the Ryukyu Islands, Southwest Japan. *Elytra* 2003; 31: 321-323.

Sasakawa K, Kim J-K, Kim J-K, Kubota K. Morphological phylogeny and biogeography of the *Pterostichus raptor* species group (Coleoptera: Carabidae) of ground beetles, endemic to the Korean Peninsula and adjacent islands. J Asia Pac Entomol 2017; 20: 7-12. https://doi.org/10.1016/j.aspen.2016.11.001

Schawaller W. Revision of the Oriental species of the genus Bradymerus Perroud, with descriptions of 29 new species (Coleoptera: Tenebrionidae). Stuttgarter Beiträge zur Naturkunde 2006; 4: 1-64.

Schawaller W. Two new species and new records of the genus *Spinolyprops* Pic, 1917 from the Oriental Region (Coleoptera, Tenebrionidae, Lupropini). ZooKeys 2012; 243: 83-94. DOI: 10.3897/zookeys.243.3879

Schimmel R, Tarnawski D. The species of the genus *Mulsanteus* Gozis, 1875 of Southeast Asia (Insecta: Coleoptera: Elateridae). Genus 2007; 14, 89-172.

Sheikh AH, Thomas M, Bhandari R, Bunkar K. Light trap and insect sampling: an overview. Int J Curr Res 2016; 8(11): 40868-40873.

Shi H, Zhou H, Liang H. Taxonomic synopsis of the subtribe *Physoderina* (Coleoptera, Carabidae, Lebiini), with species revisions of eight genera. Zookeys 2013; 284: 1-129. https://doi.org/10.3897/zookeys.284.3983

Skvarla MJ, Dowling APG. A comparison of trapping techniques (Coleoptera: Carabidae, Buprestidae, Cerambycidae, and Curculionoidea excluding Scolytinae). J Insect Sci 2017; 17(1): 7-20. https://doi.org/10.1093/jisesa/iew098

Staunton KM, Nakamura A, Burwell CJ, Robson SKA, Williams SE. Elevational distribution of flightless ground beetles in the tropical rainforests of North-Eastern Australia. PLoS ONE 2016; 11: e0155826. https://doi.org/10.1371/journal.pone.0155826

Stone BC. The summit flora of Gunung Ulu Kali (Pahang, Malaysia). Federation of Museum Journal 1981; 26(1): 157-162.

Sunday, JM, Bates AE, Dulvy NK. Thermal tolerance and the global redistribution of animals. Nature Clim Change 2002: 2(9); 686–690. https://doi.org/10.1038/nclimate1539.

Williams DW, Liebhold AM. Climate change and the outbreak of two North American bark beetles. Agr Forest Entomol 2002; 4: 87-99.

Ya'cob Z, Takaoka H, Pramual P, Low VL, Sofian-Azirun M. Breeding habitat preference of preimaginal black flies (Diptera: Simuliidae) in Peninsular Malaysia. Acta Tropica 2016; 153: 57-63. doi: 10.1016/j.actatropica.2015.10.007.

Zuur AF, Ieno EN, Walker NJ, Saveliev AA, Smith GM. Mixed effects models and extensions in ecology with R. 2009; Springer-Verlag, New York.

Table 1 Sampling effort at Fraser's Hill (FH) and Genting Highland (GH) mountains. In both mountains, traps were set at four Altitudes: 500, 1,000, 1,500 and 1,800 m a.s.l., in ten stands in both mountains; stands are indicated with letters A-J (compare Fig. 1). The three right-hand columns show the number of traps (or groups of five pitfall traps) in each stand.

**Mountain**	**Altitude**	**Stand ID**	**Light**	**Malaise**	**Pitfall**
FH	500	A	-	-	1
FH	500	B	-	-	4
FH	500	C	1	-	-
FH	1,000	D	1	2	5
FH	1,000	E	1	-	-
FH	1,500	F	1	-	2
FH	1,500	G	-	1	1
FH	1,500	H	1	1	2
FH	1,800	I	1	2	1
FH	1,800	J	1	-	4
GH	500	A	-	1	-
GH	500	B	-	1	-
GH	500	C	2	-	5
GH	1,000	D	-	-	2
GH	1,000	E	2	2	3
GH	1,500	F	-	2	4
GH	1,500	G	-	-	1
GH	1,500	H	2	-	-
GH	1,800	I	2	1	4
GH	1,800	J	-	1	1

Table 2 GLMM summary for Altitude, using Mountain as a random factor, for the number of species and rarefaction standardized number of species for 5, 10 or 20 individuals. Results are shown for light, Malaise and pitfall trap data. Numbers in "%var" columns are percentages explained by a given variable; letters in "Effect" columns show whether a given Altitude differed significantly ($p < 0.05$) and positively (pos) or negatively (neg) from the lowest Altitude (500 m a.s.l.) (in parentheses if marginally significant, i.e., $p < 0.1$) or whether this comparison was non-significant (ns; $p > 0.1$). For full output, see Appendix, Table 1.

	**LIGHT TRAPS**			**MALAISE TRAPS**			**PITFALL TRAPS**	
**Variable/Category**	**%var**	**Effect**		**%var**	**Effect**		**%var**	**Effect**
Number of species								
Mountain	7			4			53	
Altitude	41			78			11	
* 1,000 m a.s.l.		ns			neg			(pos)
* 1,500 m a.s.l.		ns			neg			ns
* 1,800 m a.s.l.		neg			neg			ns
Residuals	52			19			35	
Rarefied richness to 5 individuals								
Mountain	7			6			17	
Altitude	33			70			16	
* 1,000 m a.s.l.		ns			ns			pos
* 1,500 m a.s.l.		ns			ns			ns
* 1,800 m a.s.l.		(neg)			neg			ns
Residuals	60			24			67	
Rarefied richness to 10 individuals								
Mountain	15			7			26	
Altitude	45			72			11	
* 1,000 m a.s.l.		ns			ns			pos
* 1,500 m a.s.l.		ns			ns			ns
* 1,800 m a.s.l.		neg			neg			ns
Residuals	40			21			63	
Rarefied richness to 20 individuals								
Mountain	4			7			28	
Altitude	61			72			12	
* 1,000 m a.s.l.		ns			(neg)			pos
* 1,500 m a.s.l.		ns			neg			ns
* 1,800 m a.s.l.		neg			neg			ns
Residuals	35			21			59	

Table 3 Permutational multivariate ANOVA to assess the distinctiveness of beetle communities at different Altitudes (Alt 500, 1,000, 1,500 or 1,800 m a.s.l., or their logical combinations) as reflected by using three sampling methods (compare Fig. 2).

	**LIGHT TRAPS**							**MALAISE**							**PITFALL**					
**Variable**	**df**	**SS**	**MS**	**F**	**R^2**	**p**		**df**	**SS**	**MS**	**F**	**R^2**	**p**		**df**	**SS**	**MS**	**F**	**R^2**	**p**
Alt 500	1	0.47	0.47	1.28	0.09	0.0659		1	0.40	0.40	1.17	0.09	0.2717		1	0.48	0.48	1.58	0.04	0.0300
Residuals	13	4.75	0.37	0.91				12	4.12	0.34	0.91				38	11.55	0.30	0.96		
Alt 1000	1	0.47	0.47	1.29	0.09	0.1548		1	0.42	0.42	1.23	0.09	0.1528		1	0.97	0.97	3.34	0.08	0.0010
Residuals	13	4.75	0.37	0.91				12	4.10	0.34	0.91				38	11.06	0.29	0.92		
Alt 1500	1	0.53	0.53	1.47	0.10	0.0939		1	0.42	0.42	1.24	0.09	0.1299		1	0.46	0.46	1.51	0.04	0.0390
Residuals	13	4.69	0.36	0.90				12	4.10	0.34	0.91				38	11.57	0.30	0.96		
Total	14	5.22	1.00					13	4.52	1.00					39	12.03	1.00			
Alt 1800	1	1.36	1.36	4.56	0.26	0.0020		1	0.70	0.70	2.21	0.16	0.0030		1	0.76	0.76	2.56	0.06	0.0020
Residuals	13	3.87	0.30	0.74				12	3.82	0.32	0.84				38	11.27	0.30	0.94		
Alt 500-1000	1	0.67	0.67	1.93	0.13	0.0290		1	0.47	0.47	1.40	0.10	0.0699		1	0.97	0.97	3.33	0.08	0.0010
Residuals	13	4.55	0.35	0.87				12	4.05	0.34	0.90				38	11.06	0.29	0.92		
Alt 1000-1500	1	0.98	0.98	3.01	0.19	0.0010		1	0.68	0.68	2.13	0.15	0.0420		1	0.55	0.55	1.82	0.05	0.0070
Residuals	13	4.24	0.33	0.81				12	3.84	0.32	0.85				38	11.48	0.30	0.95		
Alt 1500-1800	1	0.67	0.67	1.93	0.13	0.0380		1	0.47	0.47	1.40	0.10	0.0619		1	0.97	0.97	3.33	0.08	0.0010
Residuals	13	4.55	0.35	0.87				12	4.05	0.34	0.90				38	11.06	0.29	0.92		
Alt 500-1500	1	1.36	1.36	4.56	0.26	0.0020		1	0.70	0.70	2.21	0.16	0.0050		1	0.76	0.76	2.56	0.06	0.0020
Residuals	13	3.87	0.30	0.74				12	3.82	0.32	0.84				38	11.27	0.30	0.94		
Alt 1000-1800	1	0.47	0.47	1.28	0.09	0.0699		1	0.40	0.40	1.17	0.09	0.2408		1	0.48	0.48	1.58	0.04	0.0210
Residuals	13	4.75	0.37	0.91				12	4.12	0.34	0.91				38	11.55	0.30	0.96		

Table 4 Significant ($p < 0.05$) indicators with $n > 4$ in the beetle data. LT = indicator of light traps; MA = indicator of Malaise traps; PT = indicator of pitfall traps; different Altitudes or ranges are shown with numbers (500, 1,000, 1,500, 1,800 m a. s. l.). Column IndVal shows indicator value for each taxon.

**Category**	**Species**	**IndVal**		**Category**	**Species**	**IndVal**
**Single-method indicators**				**Single-method indicators continued**		
LT 500	*Anomala* sp2	0.81		PT	*Harpalus* sp1	0.84
LT 500	*Anomala* sp4	0.76		PT	*Harpalus* sp2	0.91
LT 500	*Anomala* sp6	0.57		PT	*Hiletus* sp1	0.44
LT 500	*Apogonia* sp3	0.79		PT	*Inopeplus* sp1	0.63
LT 500	*Apogonia* sp5	0.79		PT	*Lebia* sp1	0.57
LT 500	*Cicindela* sp2	0.79		PT	*Omonadus* sp1	0.52
LT 500	Mordellidae A	0.80		PT	*Pentagonica* sp1	0.54
LT 500	Scarabeidae M	0.80		PT	Staphylinidae M	0.63
LT 500	Scarabeidae P	0.58		PT 500	*Pterostichus* sp2	0.87
LT 500-1000	*Epepeotes lateralis*	0.52		PT 500-1500	*Spinolyprops* A	0.53
LT 1000	*Apogonia* sp1	0.90		PT 500-1500	Staphylinidae C	0.61
LT 1000	*Cicindela* sp1	0.69		PT 1000	*Actiastes* sp1	0.71
LT 1000-1500	*Anomala* sp1	0.94		PT 1000	*Anotylus* sp2	0.86
LT 1000-1500	*Apogonia* sp2	0.96		PT 1000	*Bledius* sp1	0.78
LT 1500	Lampyridae E	0.70		PT 1000	*Lispinus* sp1	0.84
LT 1500	*Luciola* sp1	0.71		PT 1000	*Orphnebius* sp1	0.84
LT 1500-1800	*Altica* sp1	0.69		PT 1000	*Orphnebius* sp2	0.92
LT 1800	*Cleorina* sp	0.71		PT 1000	Passalidae A	0.44
LT 1800	*Hoplocerambyx spinicornis*	0.87		PT 1000	*Pityogenes* sp1	0.74
LT 1800	*Hydrovatus enigmaticus*	0.97		PT 1000	*Sunius* sp1	0.71
LT 1800	*Illeis* sp2	0.70		PT 1000-1800	*Oxylatus* sp1	0.57
MA 500	Aleocharinae sp1	0.84		PT 1500	*Hiletus* sp2	0.58
MA 500	*Anotylus* sp1	0.94		PT 1500	*Pterostichus* sp1	0.79
MA 500	*Aphthona* sp1	0.98		PT 1500-1800	*Paedarus* sp1	0.69
MA 500	*Brachypeplus* sp1	0.92		PT 1800	*Lebia* sp2	0.57

MA 500	*Brachypeplus* sp3	0.98		PT 1800	*Pterostichus* sp3	0.79
MA 500	*Bradymerus* sp2	0.99				
MA 500	*Epuraea* sp1	0.70		**Multiple-method indicators**		
MA 500	Galerucinae sp1	0.97		MA+LT 500	*Mulsanteus* sp1	0.60
MA 500	*Ischnosoma* sp1	0.95		MA+LT 500	Paederinae sp3	0.68
MA 500	*Lymantor* sp1	0.94		MA+LT 1000	*Sarmydus* sp1	0.71
MA 500	*Lymantor* sp2	0.91		MA+LT 1000-1500	*Strotocera* sp1	0.71
MA 500	*Lymantor* sp3	0.94		MA+LT 1500	Alticinae sp2	0.69
MA 500	*Sinoxylon* sp1	0.99		MA+LT 1500	*Anisandrus* sp1	0.68
MA 500	*Xyleborus* sp1	0.98		MA+LT 1500	*Brachypeplus* sp2	0.65
MA 500-1000	Aleocharinae sp2	0.58		MA+LT 1500	*Colaspoma* sp2	0.71
MA 1000	*Anomala* sp3	0.82		MA+LT 1500	Curculionidae A	0.70
MA 1000-1500	Alticinae sp1	0.70		MA+LT 1500	Meloidae A	0.71
MA 1500	Paederinae sp2	0.66		MA+LT 1500	*Nisotra* sp2	0.71
MA 1500-1800	*Xyleborus* sp2	0.71		MA+LT 1500	*Pityogenes* sp2	0.48
MA 1800	*Anomala* sp5	0.66		MA+PT 500	Aleocharinae sp3	0.62
MA 1800	*Apogonia* sp4	0.69				
MA 1800	*Lymantor* sp4	0.90				
MA 1800	*Xylothrips* sp1	0.68				

Table 4 Significant ($p < 0.05$) indicators with $n > 4$ in the beetle data. LT = indicator of light traps; MA = indicator of Malaise traps; PT = indicator of pitfall traps; different Altitudes or ranges are shown with numbers (500, 1,000, 1,500, 1,800 m a. s. l.). Column IndVal shows indicator value for each taxon.

**Category**	**Species**	**IndVal**	**Category**	**Species**	**IndVa**
**Single-method indicators**			**Single-method indicators continued**		
LT 500	*Anomala* sp2	0.81	PT	*Harpalus* sp1	0.84
LT 500	*Anomala* sp4	0.76	PT	*Harpalus* sp2	0.91
LT 500	*Anomala* sp6	0.57	PT	*Hiletus* sp1	0.44
LT 500	*Apogonia* sp3	0.79	PT	*Inopeplus* sp1	0.63
LT 500	*Apogonia* sp5	0.79	PT	*Lebia* sp1	0.57
LT 500	*Cicindela* sp2	0.79	PT	*Omonadus* sp1	0.52
LT 500	Mordellidae A	0.80	PT	*Pentagonica* sp1	0.54
LT 500	Scarabeidae M	0.80	PT	Staphylinidae M	0.63
LT 500	Scarabeidae P	0.58	PT 500	*Pterostichus* sp2	0.87
LT 500-1000	*Epepeotes lateralis*	0.52	PT 500-1500	*Spinolyprops* A	0.53
LT 1000	*Apogonia* sp1	0.90	PT 500-1500	Staphylinidae C	0.61
LT 1000	*Cicindela* sp1	0.69	PT 1000	*Actiastes* sp1	0.71
LT 1000-1500	*Anomala* sp1	0.94	PT 1000	*Anotylus* sp2	0.86
LT 1000-1500	*Apogonia* sp2	0.96	PT 1000	*Bledius* sp1	0.78
LT 1500	Lampyridae E	0.70	PT 1000	*Lispinus* sp1	0.84
LT 1500	*Luciola* sp1	0.71	PT 1000	*Orphnebius* sp1	0.84
LT 1500-1800	*Altica* sp1	0.69	PT 1000	*Orphnebius* sp2	0.92
LT 1800	*Cleorina* sp	0.71	PT 1000	Passalidae A	0.44
LT 1800	*Hoplocerambyx spinicornis*	0.87	PT 1000	*Pityogenes* sp1	0.74
LT 1800	*Hydrovatus enigmaticus*	0.97	PT 1000	*Sunius* sp1	0.71
LT 1800	*Illeis* sp2	0.70	PT 1000-1800	*Oxylatus* sp1	0.57
MA 500	Aleocharinae sp1	0.84	PT 1500	*Hiletus* sp2	0.58
MA 500	*Anotylus* sp1	0.94	PT 1500	*Pterostichus* sp1	0.79
MA 500	*Aphthona* sp1	0.98	PT 1500-1800	*Paedarus* sp1	0.69
MA 500	*Brachypeplus* sp1	0.92	PT 1800	*Lebia* sp2	0.57
MA 500	*Brachypeplus* sp3	0.98	PT 1800	*Pterostichus* sp3	0.79
MA 500	*Bradymerus* sp2	0.99			
MA 500	*Epuraea* sp1	0.70	**Multiple-method indicators**		
MA 500	Galerucinae sp1	0.97	MA+LT 500	*Mulsanteus* sp1	0.60
MA 500	*Ischnosoma* sp1	0.95	MA+LT 500	Paederinae sp3	0.68
MA 500	*Lymantor* sp1	0.94	MA+LT 1000	*Sarmydus* sp1	0.71
MA 500	*Lymantor* sp2	0.91	MA+LT 1000-1500	*Strotocera* sp1	0.71
MA 500	*Lymantor* sp3	0.94	MA+LT 1500	Alticinae sp2	0.69
MA 500	*Sinoxylon* sp1	0.99	MA+LT 1500	*Anisandrus* sp1	0.68
MA 500	*Xyleborus* sp1	0.98	MA+LT 1500	*Brachypeplus* sp2	0.65
MA 500-1000	Aleocharinae sp2	0.58	MA+LT 1500	*Colaspoma* sp2	0.71

~~MA 1000~~	~~*Anomala* sp3~~	~~0.82~~		~~MA+LT 1500~~	~~Curculionidae A~~	~~0.70~~
~~MA 1000-1500~~	~~Alticinae sp1~~	~~0.70~~		~~MA+LT 1500~~	~~Meloidae A~~	~~0.71~~
~~MA 1500~~	~~Paederinae sp2~~	~~0.66~~		~~MA+LT 1500~~	~~*Nisotra* sp2~~	~~0.71~~
~~MA 1500-1800~~	~~*Xyleborus* sp2~~	~~0.71~~		~~MA+LT 1500~~	~~*Pityogenes* sp2~~	~~0.48~~
~~MA 1800~~	~~*Anomala* sp5~~	~~0.66~~		~~MA+PT 500~~	~~Aleocharinae sp3~~	~~0.62~~
~~MA 1800~~	~~*Apogonia* sp4~~	~~0.69~~				
~~MA 1800~~	~~*Lymantor* sp4~~	~~0.90~~				
~~MA 1800~~	~~*Xylothrips* sp1~~	~~0.68~~				

Supplementary Table SAppendix 1. Twenty most abundantly captured species in our data, sorted according to trap-type specific sample sizes; n indicates number of individuals in the data. Light gray: species shared between light and Malaise traps; dark gray: species shared with pitfall, light and Malaise traps.

Light	n	Malaise	n	Pitfall	n
Anomala_sp1	53	Aleocharinae_sp1	88	Orphnebius_sp1	121
Apogonia_sp2	48	Ischnosoma_sp1	62	Pterostichus_sp2	107
Apogonia_sp1	46	Lymantor_sp2	58	Harpalus_sp1	104
Lymantor_sp3	27	Sinoxylon_sp1	43	Paedarus_sp1	99
Apogonia_sp5	23	Bradymerus_sp2	40	Bledius_sp1	80
Apogonia_sp3	22	Lymantor_sp3	38	Harpalus_sp2	78
Lymantor_sp2	22	Apogonia_sp4	31	Pterostichus_sp1	74
Anomala_sp3	21	Brachypeplus_sp3	31	Pterostichus_sp3	73
Lymantor_sp4	21	Brachypeplus_sp1	30	Inopeplus_sp1	70
Colaspoma_sp2	19	Paederinae_sp3	30	Oxylatus_sp1	59
Anomala_sp2	18	Xyleborus_sp1	24	Orphnebius_sp2	56
Anomala_sp4	16	Galerucinae_sp1	22	Pityogenes_sp1	53
Altica_sp1	15	Lymantor_sp4	19	Anotylus_sp2	48
Apogonia_sp4	15	Anomala_sp3	16	Pentagonica_sp1	45
Illeis_sp2	14	Lymantor_sp1	15	Lispinus_sp1	42
Brachypeplus_sp3	13	Anomala_sp1	14	Staphy_C	41
Pityogenes_sp1	13	Apogonia_sp2	13	Aleocharinae_sp3	32
Brachypeplus_sp2	11	Apogonia_sp1	13	Brachypeplus_sp1	31
Cicindela_sp2	11	Alticinae_sp1	13	Lebia_sp2	30
Hydrovatus_enigmaticus	11	Pityogenes_sp1	11	Staphy_M	30

Figure captions

Fig. 1. Study location in Malaysia. Left: map of Malaysia, with major mountain regions shown with gray; the two squares show locations of Fraser's Hill (top right) and Genting Highland (bottom right) mountains. Right-hand graphs show locations of ten study stands in each study mountain (denoted with letters A-J; compare Table 1); black lines show major highways; Altitudes are indicated with numbers (500, 1,000, 1,500 or 1,800 m a.s.l.). In the bottom right-hand graph, the gray patch shows a forest reserve. (The figure is similar but not identical to the original image and is therefore for illustrative purposes only).

Fig. 2. Rarefaction curves for the three trap types and the pooled sample. The end point of each curve indicates the trap-type specific or pooled (total) number of individuals.

Fig. 3. Average samples + SE of four measures of species richness for three sampling methods according to Altitude. Number of species, and rarefaction standardized richness based on 5, 10 and 20 individuals are shown. Note that the X axis length for pitfall trap samples is different.

Fig. 4. Rank abundance (mean + SE) of twenty most abundant species in each trap type at different Altitudes (500, 1,000, 1,500 and 1,800 m a.s.l.). Note that species are mostly different for each trap type, each sorted according to the rank order of the total number of individuals in that trap type. Grey columns show species that were shared between light and Malaise traps, and white columns show the shared species between pitfall, light and pitfall traps (one species). For species identities, see Appendix.

Fig. 5. NMDS plots for Malaysian beetle data, separate runs for three sampling methods (light, Malaise or pitfall traps) at different Altitudes (500 m = black squares, 1,000 m = red circles, 1,500

m = green up-triangles and 1,800 m = blue down-triangles). For statistical comparisons between Altitudes, see Table 3.

Fig. 6. MRT for sampling-method specific beetle data, using mountain (Fraser's Hill or Genting Highland) and Altitude (500, 1,000, 1,500 or 1,800 m a.s.l.) as explanatory variables. The column plots show relative abundance of each species captured using a given trap type, sorted according to the rank order of abundance of the total trap-type specific sample. Numbers in parentheses below each end branch show the number of samples falling into that branch.

To the Editors of PLOS ONE

We thank you for a possibility to resubmit a revised version of our manuscript "The functioning of different beetle (Coleoptera) sampling methods across altitudinal gradients in Peninsular Malaysia". We have tried our best to account for all referee comments. Below, we provide detailed responses to the comments by the two reviewers. We think that these comments greatly improved the manuscript and were therefore happy to include a thank you to Acknowledgements.

On behalf of all authors,

Muneeb M. Musthafa

Reviewer Comments	Authors Responses
**Reviewer #1**	
**ABSTRACT**	
• If you want to keep this format to describe your results, I recommend rewrite something like this: "Our finds were..."	This sentence had been rewritten as suggested
• …. only light traps...	Corrected as suggested
• It seems a little vague, please be more specific describing your results in the abstract. It is your time to show a compilation of your paper. Which is considerable? Which is less so?	This sentence is sharpened as follows: "Only light traps (but not the other trap types) distinguished high from middle or low altitudes in terms of beetle richness and assemblage composition."
**INTRODUCTION**	
• Please, rewrite! It seems little confuse this sentence as well as I specified in the first review:<br>Community composition – species, their relative shares, and interactions (biotic responses) –largely determines the general biodiversity response to environmental variation. The Climate change sentence is lost in the text, and don't add up to your objective, you can use it in the discussion and conclusion part, but it does not make any difference in the introduction.	This sentence was indeed unclearly written so we rewrote it as follows: "Community composition is largely determined by the relative abundances of, and interactions among, its species, which contribute to the general biodiversity response to environmental variation." We also omitted most of the climate notes from the manuscript as the reviewer is right in that we did not directly assess climatic issues.
• Please, rewrite this sentence.<br>I suggest: "The objective of the present study is evalute the consistency of light, Malaise and	Following the reviewers advise, we modified the sentence as: "The objective of the present study is to evaluate the consistency of light,

pitfall trap samples in relation to the altitudinal gradient".	Malaise and pitfall trap samples in reflecting an altitudinal gradient in tropical Malaysia."
**METHODS**	
• Exclude this comma	We removed the extra comma.
• Please, see the comment in the map:<br>Please, try to improve this map. Your map has several problems. I can't see which group of points represent each altitude (maybe is the map resolution, but it is better represent this with some color in the legend). Some points are overlap (there are many function in QGIS that you can correct this problem - for example see in points proprieties/Labels/Callouts). You need to use a better layer or some shapefile, add maps coordinates, add scale, add the north arrow and structure better the overview. The previous map was better than this map.	We attempted to clarify and complement the map by showing precise locations of each trap at different sites, referred to as A-J according to Table 1. We avoided overlapping symbols and text as much as possible. We applied Google Maps (open access data) as a baseline. We added compass lines and scale bars to the maps.
• I still don't understand how did you arrange 25 pitfall traps in four altitudes. The map show to me only 20 pitfall traps. Please, check it.	Thank you for pointing out an error in the text! We had 40 pitfall traps so the text has been clarified as follows: "We sampled beetles using two Malaise traps, two light traps, and 10 (5 groups of 2) pitfall traps at each altitude (500 m, 1,000 m, 1,500 m, and 1,800 m a.s.l.) at both mountain slopes."
• Why 40 pitfall traps if you describe above 25 pitfall traps in each mountain? Please, check it.	See previous point: we had 40 traps (5 sets of 2 locations at 4 elevations)
• I'm still not convinced with your analysis. You assumed Gaussian distribution, because you evaluated your data using only Q-Q plots and transform your data. I don't recommend transform your data. It is better to find a better distribution that fits your data. You should use some analysis which you can test the normality such as shapiro test. Quasi-poisson is not a distribution. You use Quasi-poisson if you found overdispersion in Poisson model. Did you evaluate your model with Poisson? There is overdispersion?<br>• Did you perform some diagnostics after to run your model? Did you test the normality of residuals and homogeneity of variance? Which R package did you perform your analysis? Please, rewrite your data analysis and check all these things.	We followed the reviewer's advice by adding assessments of GLMM residual normality and heteroskedasticity. Moreover, as these and the QQ plots sometimes suggested slight departures from normality, we reran the models using a novel "robust LMM" approach, which is tailored for data with outliers while being technically quite similar to lme4. As these reruns produced similar results to our initial GLMM, we conclude that the initial GLMM produced reliable results, so we therefore maintain the initial GLMM results in the main document (but present the robust LMM results in the Supplementary materials). We think it is also important to realize that the QQ plots in Supplementary files measure residual normality, which is a commonly used way to assess the appropriateness of GLMM, and that none of the presented analyses are or were done with transformed data (we used transformed

	data in some trials in the supplementary files of the previous manuscript version, which are now not presented or referred to). We were apparently not very clear with these things earlier on. We thus removed the various GLMM trials from the previous version of Supplementary files and now show the normality, heteroskedasticity and robust LMM results (for comparisons with the initial GLMM) in the Supplementary files.<br>The rest of the information can be found in the manuscript.
• How did you analyze the species accumulation curves? Which package? Please, describe this in MM.	We restructured Material and Methods to clarify these. (They were initially in the end of Material and Methods.) We now mention each package in text sections where the analysis in question is described.
**RESULTS**	
• I didn't see any significant improvement in relation to the previous version. Please, detail more your finds.	We have now restructured and rewritten sections in Results to improve the text flow. We are unsure what the reviewer means with detailing findings, but hope that the rewritten parts better fulfil this request too.
• I didn't find this Fig.	This figure is referred to as Supplementary Table S6, as it is really a table not figure. Apologies for the lack of clarity here!
• This seems very speculative. Please, remove.	Removed as suggested
• Again, this seems very speculative. It is obvious that there are climact changes along the altitudinal gradient, but you don't have this data. You don't test this data with climact factors. Many of your explanations are related with climatic factors. So, why you didn't test the climatic factors? I suggest do less emphasis to climact factors, trying to explain your data and focus in your finds.	We agree with the reviewer in that we did not really deal with climate, but many things that change in concert with altitude. This part too was removed as suggested.
• So, you can't discuss a half of what you discussed in the previous topic.	We agree with the reviewer in that we did not really deal with climate, but many things that change in concert with altitude. This part too was removed as suggested.
• The reader will judge this. Please, remove.	We see the point here and removed the section as suggested
**CONCLUSION**	

• Whenever possible, we recommend the use of multiple collecting methods in environmental impact assessments on biodiversity.	We rewrote the recommendation as suggested by the reviewer.
**FIGURES**	
• Figure 1 – Put the names of the mountains in the first image. Identify the images A, B e C.	Figure 1 has been re-drawn with all the details.
