# Supplementary material for: The functioning of different beetle (Coleoptera) sampling methods across altitudinal gradients in Peninsular Malaysia
Source: PLoS One. 2022 Mar 31;17(3):e0266076. doi: 10.1371/journal.pone.0266076 (PMC8970512; doi:10.1371/journal.pone.0266076)
Supplement: Attachment — Submitted filename: Response to Reviewers PLOS ONE.docx [file pone.0266076.s010.docx]

To the Editors of PLOS ONE

We thank you for a possibility to resubmit a revised version of our manuscript “The functioning of different beetle (Coleoptera) sampling methods across altitudinal gradients in Peninsular Malaysia”. We have tried our best to account for all referee comments. Below, we provide detailed responses to the comments by the two reviewers. We think that these comments greatly improved the manuscript and were therefore happy to include a thank you to Acknowledgements.

On behalf of all authors,

Muneeb M. Musthafa

| Reviewer Comments | Authors Responses |
| --- | --- |
| **Reviewer #1** | |
| **ABSTRACT** | |
| - If you want to keep this format to describe your results, I recommend rewrite something like this: "Our finds were..." | This sentence had been rewritten as suggested |
| - …. only light traps... | Corrected as suggested |
| - It seems a little vague, please be more specific describing your results in the abstract. It is your time to show a compilation of your paper. Which is considerable? Which is less so? | This sentence is sharpened as follows: “Only light traps (but not the other trap types) distinguished high from middle or low altitudes in terms of beetle richness and assemblage composition.” |
| **INTRODUCTION** | |
| - Please, rewrite! It seems little confuse this sentence as well as I specified in the first review:   Community composition – species, their relative shares, and interactions (biotic responses) –largely determines the general biodiversity response to environmental variation. The Climate change sentence is lost in the text, and don’t add up to your objective, you can use it in the discussion and conclusion part, but it does not make any difference in the introduction. | This sentence was indeed unclearly written so we rewrote it as follows: “Community composition is largely determined by the relative abundances of, and interactions among, its species, which contribute to the general biodiversity response to environmental variation.” We also omitted most of the climate notes from the manuscript as the reviewer is right in that we did not directly assess climatic issues. |
| - Please, rewrite this sentence.   I suggest: "The objective of the present study is evalute the consistency of light, Malaise and pitfall trap samples in relation to the altitudinal gradient". | Following the reviewers advise, we modified the sentence as: “The objective of the present study is to evaluate the consistency of light, Malaise and pitfall trap samples in reflecting an altitudinal gradient in tropical Malaysia.” |
| **METHODS** | |
| - Exclude this comma | We removed the extra comma. |
| - Please, see the comment in the map:   Please, try to improve this map. Your map has several problems. I can't see which group of points represent each altitude (maybe is the map resolution, but it is better represent this with some color in the legend). Some points are overlap (there are many function in QGIS that you can correct this problem - for example see in points proprieties/Labels/Callouts). You need to use a better layer or some shapefile, add maps coordinates, add scale, add the north arrow and structure better the overview. The previous map was better than this map. | We attempted to clarify and complement the map by showing precise locations of each trap at different sites, referred to as A-J according to Table 1. We avoided overlapping symbols and text as much as possible. We applied Google Maps (open access data) as a baseline. We added compass lines and scale bars to the maps. |
| - I still don't understand how did you arrange 25 pitfall traps in four altitudes. The map show to me only 20 pitfall traps. Please, check it. | Thank you for pointing out an error in the text! We had 40 pitfall traps so the text has been clarified as follows: “We sampled beetles using two Malaise traps, two light traps, and 10 (5 groups of 2) pitfall traps at each altitude (500 m, 1,000 m, 1,500 m, and 1,800 m a.s.l.) at both mountain slopes.” |
| - Why 40 pitfall traps if you describe above 25 pitfall traps in each mountain? Please, check it. | See previous point: we had 40 traps (5 sets of 2 locations at 4 elevations) |
| - I'm still not convinced with your analysis. You assumed Gaussian distribution, because you evaluated your data using only Q-Q plots and transform your data. I don't recommend transform your data. It is better to find a better distribution that fits your data. You should use some analysis which you can test the normality such as shapiro test. Quasi-poisson is not a distribution. You use Quasi-poisson if you found overdispersion in Poisson model. Did you evaluate your model with Poisson? There is overdispersion? - Did you perform some diagnostics after to run your model? Did you test the normality of residuals and homogeneity of variance? Which R package did you perform your analysis?   Please, rewrite your data analysis and check all these things. | We followed the reviewer's advice by adding assessments of GLMM residual normality and heteroskedasticity. Moreover, as these and the QQ plots sometimes suggested slight departures from normality, we reran the models using a novel “robust LMM” approach, which is tailored for data with outliers while being technically quite similar to lme4. As these reruns produced similar results to our initial GLMM, we conclude that the initial GLMM produced reliable results, so we therefore maintain the initial GLMM results in the main document (but present the robust LMM results in the Supplementary materials). We think it is also important to realize that the QQ plots in Supplementary files measure residual normality, which is a commonly used way to assess the appropriateness of GLMM, and that none of the presented analyses are or were done with transformed data (we used transformed data in some trials in the supplementary files of the previous manuscript version, which are now not presented or referred to). We were apparently not very clear with these things earlier on. We thus removed the various GLMM trials from the previous version of Supplementary files and now show the normality, heteroskedasticity and robust LMM results (for comparisons with the initial GLMM) in the Supplementary files.  The rest of the information can be found in the manuscript. |
| - How did you analyze the species accumulation curves? Which package? Please, describe this in MM. | We restructured Material and Methods to clarify these. (They were initially in the end of Material and Methods.) We now mention each package in text sections where the analysis in question is described. |
| **RESULTS** | |
| - I didn't see any significant improvement in relation to the previous version. Please, detail more your finds. | We have now restructured and rewritten sections in Results to improve the text flow. We are unsure what the reviewer means with detailing findings, but hope that the rewritten parts better fulfil this request too. |
| - I didn't find this Fig. | This figure is referred to as Supplementary Table S6, as it is really a table not figure. Apologies for the lack of clarity here! |
| - This seems very speculative. Please, remove. | Removed as suggested |
| - Again, this seems very speculative. It is obvious that there are climact changes along the altitudinal gradient, but you don't have this data. You don't test this data with climact factors. Many of your explanations are related with climatic factors. So, why you didn't test the climatic factors? I suggest do less emphasis to climact factors, trying to explain your data and focus in your finds. | We agree with the reviewer in that we did not really deal with climate, but many things that change in concert with altitude. This part too was removed as suggested. |
| - So, you can't discuss a half of what you discussed in the previous topic. | We agree with the reviewer in that we did not really deal with climate, but many things that change in concert with altitude. This part too was removed as suggested. |
| - The reader will judge this. Please, remove. | We see the point here and removed the section as suggested |
| **CONCLUSION** | |
| - Whenever possible, we recommend the use of multiple collecting methods in environmental impact assessments on biodiversity. | We rewrote the recommendation as suggested by the reviewer. |
| **FIGURES** | |
| - Figure 1 – Put the names of the mountains in the first image. Identify the images A, B e C. | Figure 1 has been re-drawn with all the details. |
