# Supplementary material for: The functioning of different beetle (Coleoptera) sampling methods across altitudinal gradients in Peninsular Malaysia
Source: PLoS One. 2022 Mar 31;17(3):e0266076. doi: 10.1371/journal.pone.0266076 (PMC8970512; doi:10.1371/journal.pone.0266076)
Supplement: Attachment — Submitted filename: Musthafa et al PLOS ONE R3 responses for reviewers.docx [file pone.0266076.s012.docx]

Editors of PLOS ONE

Dear Sir/Madam,

We thank you for a chance for letting us to improve our manuscript “The functioning of different beetle (Coleoptera) sampling methods across altitudinal gradients in Peninsular Malaysia” and submit a revised version to be considered for publication.

We have now made the following changes according to the reviewers’ advice. If no changes were made, we justify this decision below. We hope that the manuscript now satisfies the PLOS ONE editors.

On behalf of all authors,

Muneeb M. Musthafa

Reviewer 2:

**1. The Figure 1 still not good.**

We have now used a freely-available terrain map as a basis for this figure, and added scale bars and compass arrows. On these maps we have plotted the locations of the ten sites per mountain (referred to as letters A-J). Additions of precise locations of every trap would have made the map unreadable (realistically, this would be a maximum of 10-15 cm wide in a journal page); however, should anyone be interested in these, coordinates are given in Supplementary materials.

**2. I did not find Figures 3, 4, 5 and 6 in this version of the manuscript.**

Apologies for this, we hope this was not an error of ours. We nevertheless pay attention in including all figures (1-6), tables and supplementary materials. The data are added as another supplementary file in text format.

**3. In the previous review I asked to avoid talk about climate change in the discussion. This subject in your manuscript it seems speculative. You can talk about conservation issues and in the final sentence, use as example land use and global climate changes. Remember, your manuscript tested the differences among sample methods along a altitude gradient and did not test any hypothesis about climate change.**

We agree with this statement and have omitted climate discussions, except for one point in Discussion where additional follow-ups are recommended (for climate reasons).

Reviewer 4:

**1. I highlight the importance of the work as it was carried out in a geographic area with few studies on Coleoptera biodiversity in altitudinal gradients.**

We agree with this note and highlighted this on lines 53-54.

**2. One of my most important criticisms is the lack of an analysis of the efficiency of the sampling by the sample coverage estimator (Chao & Jost 2012: Ecology 93(12):2533–2547) that would have allowed first to check the quality of the samplings and secondly a comparison of the richness of species between types of sampling methods much more valid. These analyses could be complementary to the GLMM since the corrected Species Richness values would be used in the latter (if this were the case).**

Thank you for this note, and pointing out this richness approach. We applied the Chao & Jost paper, used iNEXT package to calculate the coverage estimator asymptotes for each sample, and subjected these to the same GLMM approaches as for the rest of the richness estimates. The model performances are added to respective Tables and supplementary materials. Regrettably the pitfall data performed poorly due to a few apparent outliers – these could have been collected on richer than average soils, but we do not have data on this – and the robust LMM did not shed much light on this, except for confirming that pitfall data indeed largely reflected differences between mountains and altitudinal effect was much smaller.

**3. I also consider it important to add information on the temperature and humidity gradient (at least) in the Material and methods section since part of the results and the discussion focus on the importance of the environmental gradient in a climate change scenario.**

While we agree in that these measures are of importance or interest, the author MMM is currently preparing another manuscript on these variables. Therefore, we would not like to include these here. However, we refer to the general patterns of temperature, humidity and luminosity according to altitude (lines 87-90). Also, as the altitudinal effects generally reflected this to be quite small, we point out these generalities once again in Discussion (line 317).

**4. Lastly, I have observed some minor mistakes that need to be considered**

Thank you for pointing these out – these are all corrected now.
